# CO_2_ reduction to CO on an iron-porphyrin complex with crown-ether appended cation-binding site†

**DOI:** 10.1039/d5dt00119f

**Published:** 2025-02-19

**Authors:** Chengxu Zhu, Adarsh Koovakattil Surendran, Carmine D'Agostino, Jana Roithová, Sam P. de Visser

**Affiliations:** a Manchester Institute of Biotechnology, The University of Manchester 131 Princess Street Manchester M1 7DN UK sam.devisser@manchester.ac.uk; b Department of Chemical Engineering, The University of Manchester Oxford Road Manchester M13 9PL UK; c Department of Spectroscopy and Catalysis, Institute for Molecules and Materials, Radboud University Heyendaalseweg 135 6525 AJ Nijmegen The Netherlands j.roithova@science.ru.nl; d Dipartimento di Ingegneria Civile, Chimica, Ambientale e dei Materiali (DICAM), Alma Mater Studiorum – Università di Bologna Via Terracini 28 40131 Bologna Italy

## Abstract

With increasing carbon dioxide concentrations in the atmosphere, the utilization and conversion of CO_2_ into valuable materials is an important goal. In recent years, evidence has emerged of low-valent iron-porphyrin complexes able to bind CO_2_ and reduce it to carbon monoxide and water. To find out how the porphyrin scaffold and second coordination sphere influence the CO_2_ reduction on iron-porphyrin complexes, we study the structure, electronic and redox properties of a novel crown-ether appended porphyrin complex with cation (K^+^) binding site. Cyclic voltammetry studies show that the K^+^ binding site does not change the Fe^0/I^ and Fe^I/II^ redox potentials of the complexes. Subsequently, density functional theory calculations were performed on the catalytic cycle of CO_2_ reduction on the K^+^-bound crown-ether appended iron-porphyrin complex. The work shows that proton-donors such as acetic acid bind the K^+^ strongly and can assist with efficient and fast proton transfer that leads to the conversion of CO_2_ to CO and water. In agreement with experiment, the calculations show little perturbations of the redox potentials upon binding K^+^ to the crown-ether scaffold.

## Abstract 2

In this work a novel CO_2_ reduction catalyst is discussed that contains an iron-porphyrin complex with a K^+^ ion bound in the second coordination sphere, which assists with efficient proton delivery.

## Graphical abstract

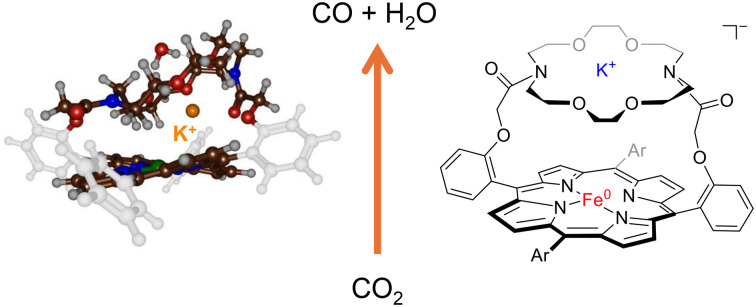

## Introduction

Since the industrial revolution, the concentration of CO_2_ in the Earth atmosphere has been steadily increasing, which is causing environmental problems for life on Earth. Important solutions are being sought to slow down or reverse the CO_2_ increase. One solution would be to store CO_2_, while another one is to focus on utilization of CO_2_ as a source for the synthesis of valuable materials using Fisher-Tropsch type processes.^1^ Unfortunately, most Fisher-Tropsch processes are energetically demanding and often require high pressure and high temperature as well as toxic and expensive heavy elements.^2^ In recent years, however, several iron-porphyrin complexes have been identified with the potential to catalyse the CO_2_ reduction reaction in a water solution.^3^ Thus, electrochemical studies showed that iron(0)-porphyrin complexes can trigger the conversion of CO_2_ to CO efficiently.^4^ Several studies investigated the effect of the structure and local environment on the CO_2_ to CO conversion reaction. In particular, the work of Chang *et al.* highlighted that a hydrogen bonding donor group in the second coordination sphere could assist with positioning and binding CO_2_ to the metal centre and assist with the proton relay.^5^

**Scheme 1:**
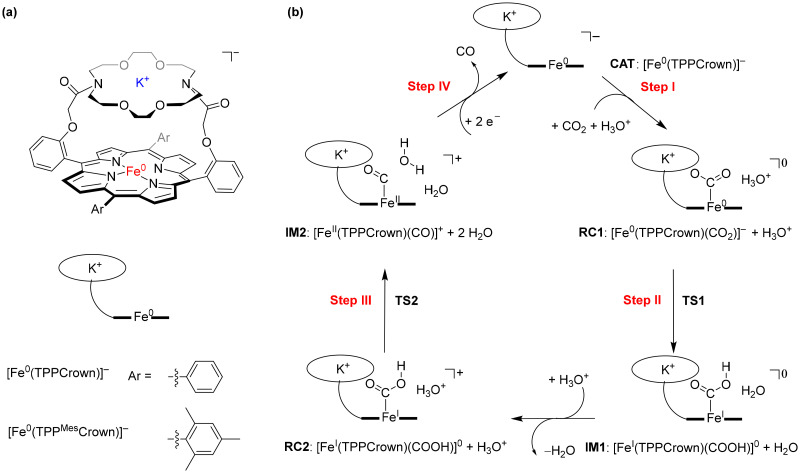
(a) Models studied in this work. (b) Catalytic cycle for CO_2_ reduction to CO studied in this work.

Our extensive studies on enzymes and biomimetic model complexes have shown that second-coordination sphere effects can play a major role in catalysis.^6^ In particular, a local dipole moment or electric field effect may weaken or strengthen bonds and direct catalysis into a specific reaction channel. We, therefore, decided to explore second-coordination sphere effects on CO_2_ to CO reduction on an iron-porphyrin complex. Previously, we calculated the catalytic cycle of CO_2_ to CO reduction on an [Fe(TPP)] system, with TPP as *meso*-tetraphenylporphyrin.^7^ In subsequent studies, a modified iron-porphyrin complex was investigated with an *ortho*-amide substituent attached to one of the TPP ligands. This complex was shown to fix CO_2_ tightly on the iron complex through hydrogen bonding interactions and enabled reactions with proton donors efficiently. A recent study demonstrated that the presence of K^+^ ions enhances CO_2_ reduction under highly acidic conditions,^8^ prompting an investigation into the influence of these ions on the reaction mechanism and catalysis. We have shown that binding K^+^ ions to a porphyrin cage with crown-like binding sites on the side above the porphyrin plane substantially accelerated the CO_2_ reduction reaction. Here, we designed and synthesised a novel iron-porphyrin complex with a crown-ether analogue linked horizontally above the porphyrin plane, see Scheme 1.

The crown-ether functionality can be used to bind different alkali metals. With cyclic voltammetry, we compared the effects of binding Na^+^ and K^+^ and benchmarked it against tetrabutylammonium (TBA) that does not fit into the crown-type macrocycle.^8^ To find out, whether potassium ions bound to the crown-ether will influence the CO_2_ reduction reaction we performed a joint experimental and computational study on the reaction cycle shown in Scheme 1b. In our work the *meso*-tetramesitylporphryin (TPP^Mes^) ligand was used appended with the aza-crown ether containing a K^+^ ion, designated TPP^Mes^Crown (for simplicity DFT calcualtions used a truncated model of TPPCrown instead of TPP^Mes^Crown). Thus, the catalytic cycle was hypothesized as shown in Scheme 1, starting from the [Fe^0^(TPPCrown)]^−^ complex (designated CAT). The reaction is expected to start with the addition of CO_2_ and a proton donor, highlighted as H_3_O^+^ in Scheme 1, to form the proton-transfer reactant complex RC1, *i.e.* [Fe^0^(CO_2_)(TPPCrown)]^−^. Subsequently, the proton donor group relays its proton to the bound CO_2_ group to form the iron(i)-COOH complex IM1. A second proton donor molecule is picked up from the solvent and binds to the periphery of the complex (structure RC2) and relays its proton to the OH group of the iron(i)-COOH complex to form water, CO and iron(ii) intermediate IM2. The cycle is expected to return to structure CAT by the expulsion of CO and the reduction of the complex with two electrons. In this work, the catalytic cycle of Scheme 1 is calculated with DFT methods and redox measurements to confirm the cycle. The combined experimental and computational studies show that the aza-crown-ether with K^+^ in the second coordination sphere does not influence the redox potentials in the reaction cycle but influences proton transfer steps and improves catalysis.

## Methods

### Experimental

All chemicals were obtained from commercial sources. The crown ether appended porphyrin ligand (H_2_(TPP^Mes^Crown)) was prepared based on a previously published procedure.^9^ The iron complex [Fe^III^(TPP^Mes^Crown)OAc] was obtained by refluxing (H_2_(TPP^Mes^Crown)) with [Fe(OAc)_2_] in dimethylformamide (DMF). The synthesis and characterization details are provided in the ESI.†

Cyclic voltammetry (CV) experiments were performed using a standard three-electrode assembly in a solution of 0.25 mM catalyst, 0.1 M of a supporting electrolyte (tetrabutylammonium tetrafluoroborate (TBABF_4_), sodium tetrafluoroborate (NaBF_4_) and potassium tetrafluoroborate (KBF_4_)) and 0.5 M H_2_O in dimethylformamide (10 mL). We used a glassy carbon working electrode, a double junction non-aqueous Ag/AgCl reference electrode filled with 2 M LiCl in ethanol as the inner electrolyte, and a platinum mesh of 2 cm^2^ area as the counter electrode. The reference electrode was calibrated against the ferrocene/ferrocenium (Fc/Fc^+^) redox couple. Prior to the measurements, the solution was bubbled through with N_2_ or CO_2_ gas for 30 minutes, and during the measurements, the corresponding gas flow was maintained through the head space.

### Computation

Density functional theory calculations were performed with the Gaussian-09 software package^10^ for the structures and catalytic cycle shown in Scheme 1. The crown-based model structure [Fe(TPPCrown)(CO_2_)] was manually created from the optimised CO_2_-bound geometry of [Fe(TPP)(CO_2_)] from our previous research on CO_2_ reduction catalysis.^11^ To account for potential variations in spin-state ordering and relative energies, three different density functional methods were assessed, namely we tested the unrestricted B3LYP-GD3BJ,^12,13^ B3LYP,^12^ and PBE0 density functionals.^14^ In general, the change of the DFT method did not influence the results dramatically and gave the same patterns and conclusions, see ESI.† All calculations were performed in the lowest energy triplet and quintet spin states. For the proton transfer steps in the catalytic cycle, three different potential proton donors were tested with different p*K*_a_ values in water at room temperature, namely H_3_O^+^, acetate (p*K*_a_ = 4.76) and phenol (p*K*_a_ = 10). A def2-SVP basis set was employed for geometry optimisations, analytical frequency calculations and constrained geometry scans (referred to as basis set BS1).^15^ Single-point energy calculations were conducted using the def2-TZVP basis set (referred to as basis set BS2) to improve the energetics. All computations, including geometry optimisations and frequency calculations, were performed utilising a solvent model (continuously polarised conductor model, CPCM) with a dielectric constant simulating water.^16^ No symmetry or geometrical constraints were applied during the calculations. The models and method applied here were previously validated against experimental work and shown to reproduce experimentally determined free energies of activation to within a few kcal mol^−1^ and predict the correct product distributions.^17^

## Results and discussion

**Fig. 1:**
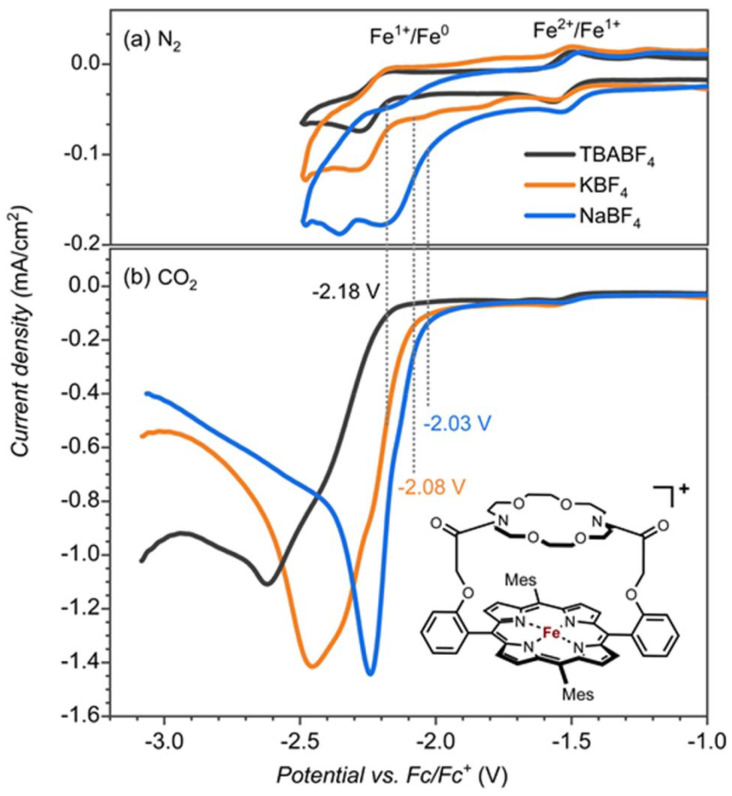
Cyclic voltammetry (CV) of [Fe^III^(TPPcrown)]^+^ (0.25 mM) measured in electrolytes containing TBABF_4_ (black), NaBF_4_ (blue) and KBF_4_ (orange) (0.1 M in DMF + 0.5 M H_2_O; scan rate 100 mV s^−1^) under (a) N_2_ and (b) CO_2_.

The iron-porphyrin complexes with the aza-crown ether aligned above the porphyrin ring offer the opportunity to study the effect of a positive charge in the second coordination sphere on the catalytic activity of the iron complex. The binding site of the attached aza-crown is analogous to that of the 18-crown-6, which is ideal for binding potassium ions. For testing the effect of potassium cations, we compared the catalytic activity of [Fe^III^(TPP^Mes^Crown)OAc] in the presence of sodium, potassium and tetrabutylammonium (TBA^+^) electrolytes with the BF_4_^−^ counter ions (Fig. 1). Contrary to the large binding affinity of Na^+^ and K^+^ to the crown ether, the TBA^+^ cations do not fit into the aza-crown ether ring because of their bulkiness. Therefore, it can serve as a reference for the system without the permanent ion above the porphyrin ring.

Cyclic voltammetry experiments with 0.25 mM [Fe^III^(TPP^Mes^Crown)OAc] in DMF under nitrogen gas showed the Fe^II^ → Fe^I^ reduction potentials at −1.53 V regardless of the presence of Na^+^, K^+^ or TBA^+^ cations in the solution (Fig. 1). However the Fe^I^ → Fe^0^ is influenced by the identity of the cations, especially the addition of Na^+^ ions to the solution shows the largest shift of ∼150 mV. The voltammogram in the presence of K^+^ electrolyte showed two minor peaks at −1.75 V and −2 V in addition to the peak at −2.18 V. The peak at −2.18 V matches with TBA^+^ electrolyte can correspond to the complex without bound to K^+^. The additional minor peaks observed in the K^+^ electrolyte are the result of the of the binding of K^+^ ions.

Subsequently, we investigated the CO_2_ reduction reaction and measured the redox potentials of the [Fe^III^(TPP^Mes^Crown)OAc] complex in the presence of TBA^+^, K^+^ and Na^+^ ions. Under a CO_2_ atmosphere, we observe a catalytic current for the CO_2_ reduction reaction at the Fe^I^ → Fe^0^ potential (Fig. 1b). The catalysis is more efficient in the presence of KBF_4_ and NaBF_4_ electrolytes than with the TBABF_4_ electrolyte. In particular, the onset of CO_2_ reduction reaction is shifted to a lower potential (by ∼100 mV in KBF_4_ and ∼150 mV In NaBF_4_), and a larger catalytic current is observed. The onset shift to the lower potential suggests the assistance of K^+^ and Na^+^ ions in the CO_2_ reduction reaction mechanism.

**Fig. 2:**
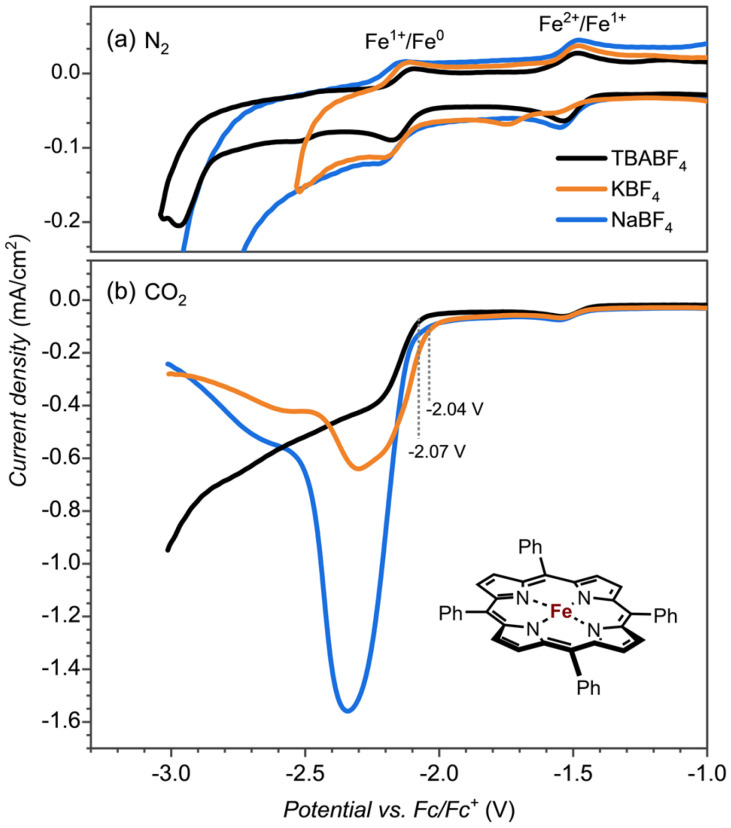
Cyclic voltammetry (CV) of [Fe^III^(TPP)]^+^ (0.25 mM) measured in electrolytes containing TBABF_4_ (black), NaBF_4_ (blue) and KBF_4_ (orange) (0.1 M in DMF + 0.5M H_2_O; scan rate 100 mV s^−1^) under (a) N_2_ and (b) CO_2_.

To test whether the redox potential changes and the CO_2_ reduction reaction mechanism are determined and influenced by the aza-crown ether appendage or the bound cation, we did a series of control experiments with the bare [Fe(TPP)] complex, see Fig. 2. The redox potentials for the Fe^II^ → Fe^I^ and Fe^I^ → Fe^0^ couples (Fig. 2a) give an onset that is slightly shifted to more negative potentials for the aza-crown appended complex than the one for the bare [Fe(TPP)] complex for which values of −1.51 V and −2.14 V are obtained. As such, the second coordination sphere and binding of a K^+^ cation to the complex does not lead to a major shift in the two redox potentials of the complex.

To test whether the assistance could be ascribed to a specific interaction of K^+^ or Na^+^ bound to the crown ether functionality with CO_2_ reduction reaction intermediates or non-specific interactions of K^+^ ions from the solution, we performed control experiments with the bare [Fe(TPP)] complex, see Fig. 2b. The CO_2_ reduction reaction as catalyzed by the [Fe(TPP)] complex showed no shift in the onset potential in NaBF_4_ electrolyte and only a minor shift by ∼30 mV in KBF_4_. However, the catalytic current is largely influenced, especially in the Na^+^ electrolyte. Consequently, Na^+^ and K^+^ ions do affect the CO_2_ reduction reaction in general, but the magnitude of the effect, as observed by the shift in redox potential, is much larger if we offer a binding site in the vicinity of the iron active site. This indicates a specific interaction between the complexed Na^+^ and K^+^ ion and a CO_2_ reduction reaction intermediate. The increase in the catalytic current is also observed with the [Fe(TPP)] complex, indicating that this is connected with the general electrolyte effect of the interaction of cations, not necessarily specific to the interaction of Na^+^ and K^+^ from the aza-crown group.^18^

### Computation

**Fig. 3:**
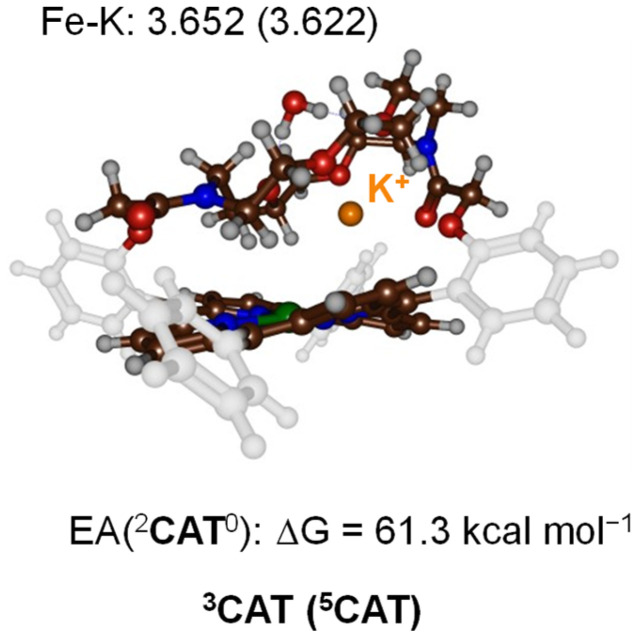
UB3LYP-GD3BJ/BS1 optimised geometries of ^3,5^CAT with bond lengths in Å. Also given is the electron affinity of ^2^CAT^0^ in kcal mol^−1^.

To test what parts of the catalytic cycle for CO_2_ reduction are influenced by the binding of K^+^ ions, we decided to do a computational study and compare the CO_2_ reduction catalytic cycle for [Fe^0^(TPP)]^2−^*versus* [Fe^0^(TPPCrown)]^−^. Let us start with a description of the reactant structure in the triplet and quintet spin states (^3,5^CAT) that contains the bare iron(0)-porphyrinate system [Fe^0^(TPPCrown)]^−^ hosting a K^+^ ion and one water molecule. The optimised geometries of ^3,5^CAT are shown in Fig. 3. The triplet spin state is the ground state by Δ*G* = 5.1 kcal mol^−1^ at B3LYP-GD3BJ level of theory. The same spin state ordering is found when the calculations are performed without dispersion corrections included in the density functional method, *i.e.*, with B3LYP the triplet is lower than the quintet spin state by Δ*G* = 1.2 kcal mol^−1^. The aza-crown ether forms a tight cavity above the distal porphyrin site with the K^+^ ion located at a distance of 3.652 Å in ^3^CAT and at 3.622 Å in ^5^CAT. Therefore, the cavity appears to have sufficient space to bind small molecules like CO_2_, CO, or O_2_. The formation free energy of ^3^CAT from a precursor complex through electron transfer, *i.e.*^2^CAT^0^ + e^−^ → ^3^CAT^−^, is calculated as the adiabatic electron affinity (EA) of ^2^CAT^0^ as Δ*G* = 61.3 kcal mol^−1^. This value would correspond to a reduction potential of 2.6 V, which compares well with the experimental number reported above. By comparison the same redox potential was obtained for the [Fe^0^(TPP)] complex.^7^ Therefore, in agreement with experimental observation the calculations predict minimal changes in the redox potential upon adding a cation to the second coordination sphere of an iron-porphyrin complex. The electron transfer and formation of ^3^CAT^−^ from ^2^CAT^0^ is exergonic and consequently will happen rapidly.

Next, we added a CO_2_ molecule to ^3^CAT and optimized its geometry and extracted the CO_2_ binding energy of the complex. Thus, without K^+^ bound inside the aza-crown ether scaffold a CO_2_ binding free energy of Δ*G* = 17.5 kcal mol^−1^ is found, while the addition of K^+^ into the aza-crown raises the binding free energy to Δ*G* = 23.5 kcal mol^−1^. Upon binding of CO_2_ to the metal centre the O–C–O angle considerably distorts from linearity. Specifically, in the presence of K^+^, the O–C–O bond angle is 128°, whereas in the absence of K^+^, the angle increases to 133°. In addition, the Fe–C bond is 1.92 Å in the complex containing K^+^, while it is elongated to over 2.0 Å in the complex without K^+^. Therefore, the presence of a K^+^ cation to the second coordination sphere facilitates the binding of CO_2_ to the iron centre leading to a stronger metal-carbon bond that will lead to more efficient CO_2_ reduction reactions.

**Fig. 4:**
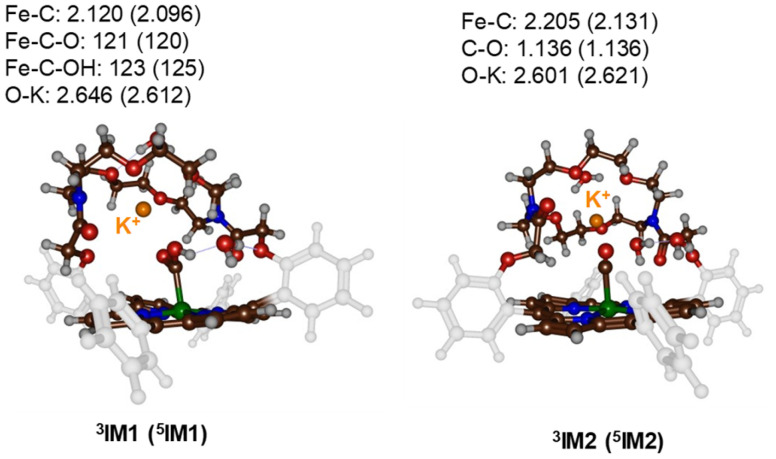
UB3LYP-GD3BJ/BS1 optimised geometries of the proton-transfer intermediates ^3,5^IM1 and ^3,5^IM2. Bond lengths are in Å and angles in degrees.

Subsequently, a proton donor molecule was added to the complex, namely a H_3_O^+^ ion was inserted into the binding cavity nearby the iron atom. A geometry optimisation of the proton transfer reactant complex RC1 was performed with DFT methods. During the geometry optimisation the proton spontaneously moves to the CO_2_ group and formed IM1 in the triplet and quintet spin states. Therefore, the p*K*_a_ value of the bound-CO_2_ complex is well higher than that of a water molecule resulting in a spontaneous and exergonic proton transfer from H_3_O^+^ to Fe–CO_2_. To estimate the barrier for proton transfer, we ran a constraint geometry scan in the reverse direction from ^3,5^IM1 to RC1. The geometry scan led to a continuous rise in energy and never stabilized to another local minimum. Therefore, the RC1 structures will be unstable in the presence of a strong proton donor and react fast and spontaneously through proton transfer from H_3_O^+^ to form hydroxycarbonyl iron complex and water without encountering a transition state for this step. The obtained optimised geometries of ^3,5^IM1 are shown in Fig. 4. The Fe–C bond is relatively long at 2.120 Å in ^3^IM1 and 2.096 Å in ^5^IM1. These distances match typical bond lengths between iron and a first-row element and for instance is seen for a histidine bound ligand in nonheme iron enzymes.^19^ The two Fe–C–O angles are just over 120° in size and cause a considerable bent into the CO_2_ structure from the original linear orientation in the gas phase. The K^+^ ion is at a distance of 2.646 Å in ^3^IM1.

Thereafter, another H_3_O^+^ molecule was added to the structures of ^3,5^IM1 to form the RC2 complexes. Also, during the geometry optimisations of the RC2 complexes a fast and facile proton transfer took place and the structures optimised to a complex containing water and an iron(ii)-CO product. As such the RC2 structures in the presence of a strong proton donor are unstable and collapse to form the ^3,5^IM2 products with large exergonicity. Again, a reverse scan was calculated for the relay the proton back to the water molecule to form H_3_O^+^. These scans did not give evidence of a transition state for proton transfer. Consequently, H_3_O^+^ will be a very efficient proton source for CO_2_ reduction reactions on an iron(0)-porphyrin complex. The optimised geometries of the ^3,5^IM2 structures are shown in Fig. 4. The Fe–C bond has elongated somewhat as compared to the IM1 structures to values of 2.205 Å for ^3^IM2 and 2.131 Å for ^5^IM2. These distances compare well for CO bound heme complexes calculated previously.^20^ The distance of the nearest water molecule to K^+^ is at 2.601 Å in ^3^IM1.

To explore the mechanism in the presence of an alternative proton donor, we also studied reactions with neutral phenol and acetic acid as well as systems with several water molecules included. The use of phenol as a proton donor and several explicit water molecules added to the model, however, did not lead to a feasible proton transfer pathway. In particular, constraint geometry scans for O–H bond formation led to a continuous rise in the energy and never led to a barrier and the creation of the Fe(i)C(O)(OH) product. This is most likely because of the crossing of different-spin potential energy surfaces (PES), and the constrained scan does not capture the overall PES well. Nevertheless, the size of a phenol molecule makes its positioning nearby CO_2_ difficult with the appended crown-ether in place and therefore, phenol is not suitable as proton donor for this system.

**Fig. 5:**
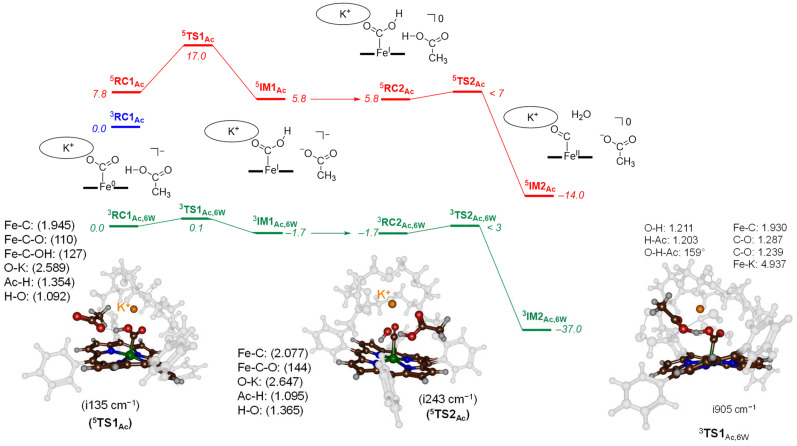
UB3LYP-GD3BJ calculated proton transfer free energy landscape using acetic acid as proton source. Values are BS2 energies corrected with BS1 calculated solvent, zero-point energy, thermal and entropic contributions at 298 K in kcal mol^−1^. Optimised transition state structures give bond lengths in Å, bond angles in degrees and the imaginary frequency in cm^−1^.

To characterize transition states for the proton transfer steps during the CO_2_ reduction reaction, we finally tested acetic acid as the proton donor. With acetic acid as the proton source added to ^3,5^CAT we were able to locate several transition state structures and local minima for the overall CO_2_ reduction reaction as shown in Fig. 5. The calculated free energy landscape and transition state structures for CO_2_ reduction on the iron-porphyrin complex with acetic acid as a proton donor are shown in Fig. 5. On the triplet spin state, we did not manage to locate a viable proton transfer channel, and all attempts to locate an intermediate ^3^IM1 resulted in proton transfer back to the acetate group. Several starting structures were used with the acid in different positions and orientations with respect to the iron-CO_2_ complex, including a model with acetate pointing toward K^+^. However, none of those models led to viable proton transfer pathways on the triplet spin state. The same applies to the geometry search for ^3^IM2, which always collapsed back to ^3^RC2. We then performed constraint geometry scans for these steps on the triplet spin state, and the energetics of proton transfer continuously increases and never stabilises or forms a local minimum. Therefore, on the triplet spin state, the protonation of the CO_2_-bound complex cannot happen. Interestingly, we did locate transition state structures on the quintet spin state surface. In particular, the first proton transfer is endergonic by 5.8 kcal mol^−1^ with respect to ^3^RC1_Ac_, while free energy of activation *via*^5^TS1_Ac_ of Δ*G*^‡^ = 17.0 kcal mol^−1^ is obtained. These barriers are relatively low in free energy and, therefore, the reaction should proceed under room temperature conditions quickly.

The optimised transition state structure ^5^TS1_Ac_ is shown on the left-hand-side of Fig. 5. The structure is product-like with an O–H distance to acetate of 1.354 Å, while the accepting oxygen atom has a distance to the proton of 1.02 Å. The imaginary frequency is small, namely i135 cm^−1^; however, its visualisation gives a clear O–H–O stretch vibration. Typical imaginary frequencies for hydrogen atom abstraction are well over i1000 cm^−1^,^21^ although hydride transfer is often accompanied by a transition state with much smaller imaginary frequencies.^22^

For the second proton transfer step, we added a new proton to the acetate group and increased the total charge of the system by one unit, making the reaction complexes neutral. We then searched for a proton transfer transition state and located ^5^TS2_Ac_. Its energy, however, is of similar energy as ^5^RC2_Ac_ and therefore, the proton transfer will be fast and lead to CO and H_2_O efficiently. Indeed, the overall reaction energy is exergonic by Δ*G* = −14.0 kcal mol^−1^. The optimized geometry of ^5^TS2_Ac_ is shown in Fig. 5. The imaginary frequency in ^5^TS2_Ac_ is again small (i243 cm^−1^) and includes simultaneous C–O cleavage and proton transfer. As such, the C–O bond breaking and proton transfer are synchronous and not stepwise. The transferring proton is closest to the acetate group in ^5^TS2_Ac_ with a distance of 1.095 Å, while the accepting H–O distance is 1.365 Å. The transition state shows little unpaired spin density on the carbon atom of the O=C–OH group. Consequently, the step corresponds to a proton transfer coupled with C–O bond cleavage and formation of neutral iron(ii)-carbonyl complex.

**Fig. 6:**
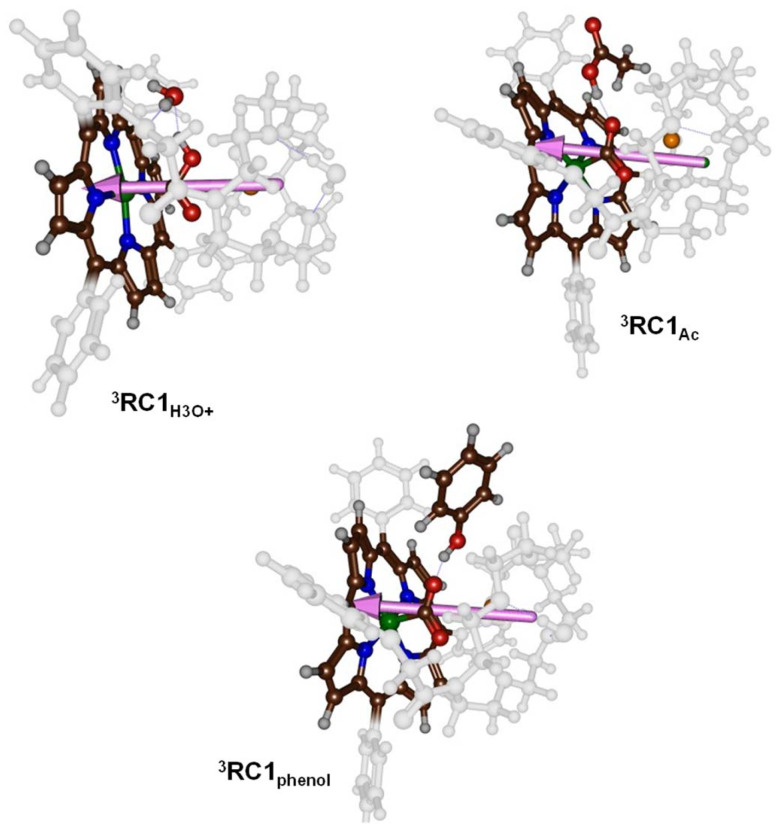
Dipole moment vectors in ^3^RC1_H_3_O^+^_, ^3^RC1_Ac_ and ^3^RC1_phenol_.

Finally, models were tested with additional explicit water molecules included. Thus, the landscape in green in Fig. 5 represents the [Fe(TPPCrown)] system with acetate and six explicit water molecules with all structures assigned with the subscript Ac,6 W to specify this model. In ^3^RC1_Ac,6W_ the acetate is positioned with its carboxylate at a distance of 2.699 Å from K^+^, while the acetate proton forms a hydrogen bond to bound CO_2_ at a distance of 1.503 Å. The six water molecules are positioned around the acetate, CO_2_ and aza-crown ether groups and form hydrogen bonding interactions that keep them in position. The proton transfer *via*^3^TS1_Ac,6W_ encounters a small barrier of about 0.1 kcal mol^−1^. Despite the low barrier, the ^3^TS1_Ac,6W_ structure was fully characterized and its geometry is displayed in Fig. 6. The transition state has a large imaginary frequency of i905 cm^−1^ for the O–H–O stretch vibration. This large imaginary frequency implies that the barrier is sharp and will incur a large kinetic isotope effect. The transferring proton is midway in between the donor and acceptor groups with distances of 1.203 and 1.211 Å, respectively. The two C–O bonds in the CO_2_-bound iron complex are very similar (1.239 and 1.287 Å). Hence, in the transition state, the two bonds have equal strength. After the transition state, the system relaxes to ^3^IM1_Ac,6W_, which is 1.7 kcal mol^−1^ in free energy more stable than the reactant complex. These two structures, therefore, will be in equilibrium until another proton source initiates the next reaction step. Clearly, hydrogen bonding interactions of water molecules facilitate low-energy, fast proton transfer from acetic acid to bound CO_2_. We finally calculated a second proton transfer step by adding another proton to acetate and studied the pathway to form CO and water. A proton transfer is barrierless and relays the system to the products rapidly.

To understand the differences in reactivity with different proton sources, we analysed our structures in more detail. Thus, in our previous work on CO_2_ reduction by iron-porphyrin complexes, we predicted that a dipole moment aligned with a proton transfer arrangement would lower the proton transfer barriers.^11^ The dipole moment would then create an electric field effect and influence the charge distributions in the system. In particular, these electric field effects have been shown to weaken and strengthen chemical bonds and direct regio- and chemoselectivity patterns in catalysis.^6*b*,23^ The dipole moment vectors in ^3^RC1_H_3_O^+^_, ^3^RC1_Ac_ and ^3^RC1_phenol_ are shown in Fig. 6. In ^3^RC1_H_3_O^+^_ and ^3^RC1_Ac_, the electric dipole moment is nicely aligned with the proton transfer from the donor to the CO_2_ group and consequently lowers the proton transfer barriers. Indeed, small proton transfer barriers are seen for these systems. With phenol as a proton donor, however, the phenol group cannot align itself with the electric dipole vector of the catalyst due to steric interactions of the aromatic ring of the phenol and the crown ether system. Consequently, for ^3^RC1_phenol_ the phenol OH group points against the dipole vector of the catalyst rather than along the dipole vector, as is seen for ^3^RC1_H_3_O^+^_ and ^3^RC1_Ac_. The dipole moment vectors in Fig. 6, therefore, highlight important electrostatic interactions between donor and acceptor groups and emphasise on an ideal orientation of the two groups for fast and efficient proton transfer.

## Conclusions

Our study demonstrates that incorporating potassium ions into the aza-crown ether scaffold appended above an iron-porphyrin catalyst significantly enhances the efficiency of CO_2_ reduction. This improvement is primarily due to the stabilizing role of K^+^ in the reaction intermediates and its ability to facilitate critical proton transfer steps during the catalytic cycle. As such the secondary coordination sphere plays a major role in the CO_2_ reduction cycle and assists with positioning the various components in an ideal orientation for catalysis. Accordingly, filling the crown ether scaffold with K^+^ does not significantly alter the redox potentials of the Fe(ii)/Fe(i) and Fe(i)/Fe(0) couples. These values remain comparable to those of the unmodified iron-porphyrin complex. Our findings highlight the importance of second-coordination sphere effects in modulating catalytic performance.

## Data availability

The data supporting this article have been included as part of the ESI.†

## Conflicts of interest

There are no conflicts to declare.

## Supplementary Material

DT-054-D5DT00119F-s001
