# Supplementary material for: CO2 reduction to CO on an iron-porphyrin complex with crown-ether appended cation-binding site
Source: Dalton Trans. 2025 Feb 19;54(12):4918–26. doi: 10.1039/d5dt00119f (PMC11851269; doi:10.1039/d5dt00119f)
Supplement: DT-054-D5DT00119F-s001 [file DT-054-D5DT00119F-s001.pdf]

Supplementary Information (SI) for Dalton Transactions.
This journal is © The Royal Society of Chemistry 2025

Electronic Supporting Information

CO_2 Reduction to CO on an Iron-Porphyrin Complex with Crown-Ether Appended Cation-Binding Site

Chengxu Zhu,[ab] Adarsh Koovakattil Surendran,[c] Carmine D'Agostino,[bd] Jana Roithová,*[c] and Sam P. de Visser*[ab]

[a] *Manchester Institute of Biotechnology, The University of Manchester, 131 Princess Street, Manchester M1 7DN, United Kingdom.*
[b] *Department of Chemical Engineering, The University of Manchester, Oxford Road, Manchester M13 9PL, United Kingdom.*
[c] *Department of Spectroscopy and Catalysis, Institute for Molecules and Materials, Radboud University, Heyendaalseweg 135, 6525 AJ Nijmegen, The Netherlands.*
[d] *Dipartimento di Ingegneria Civile, Chimica, Ambientale e dei Materiali (DICAM), Alma Mater Studiorum – Università di Bologna, Via Terracini, 28, 40131 Bologna, Italy.*

Corresponding author emails:
j.roithova@science.ru.nl (JR) and sam.devisser@manchester.ac.uk (SPdV)

Experimental Details

1. Cyclic voltammetry

Homogeneous cyclic voltammetry (CV) experiments were performed using a standard three-electrode cell in a solution containing 0.25 M of the [(TPP^{Mes}Crown)Fe^{III}(OAc)] and 0.1 M of the corresponding supporting electrolytes (*n*-Bu_4BF_4, $NaBF_4$, KBF_4) in 10 mL dimethylformamide (DMF) with the water concentration at 0.5 M. A glassy carbon working electrode (WE) was used and polished between each measurement. A double junction non-aqueous Ag/AgCl reference electrode (RE) filled with 2 M LiCl in ethanol as the inner electrolyte and the DMF solution as the outer electrolyte was used. The counter electrode (CE) was a platinum mesh of 2 cm^2 area. The reference electrode was calibrated against the ferrocene/ferrocenium redox couple. Prior to the measurements, the solution was deoxygenated with argon or saturated with CO_2 for 30 minutes, and during the measurements, the corresponding gas was kept flowing over the head space.

2. Synthesis of [(TPP^{Mes}Crown)Fe^{III}(OAc)] complex

2.1 Synthesis of mesityldipyrromethane

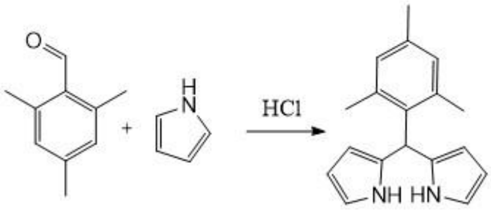

A Schenk flask flushed with nitrogen was filled with 200 mL of water containing 3 mL of concentrated HCl, 2,4,6-trimethylbenzaldehyde (4.00 g, 1 eq, 27.0 mmol), and 1H-pyrrole (5.43 g, 3 eq, 81.0 mmol). A white emulsion was formed and stirred overnight at room temperature under nitrogen shielded from light (covered with Al foil). A black solid was formed, filtered, and washed with 3 × 50 mL water followed by 3 × 50 mL heptane. The solid was then dissolved in dichloromethane (giving a dark green solution) and purified by silica column chromatography using eluent dichloromethane/heptane 60/40. The fraction was evaporated, and a white solid was obtained, followed by washing it with heptane and then pentane. The yield of white solid 2,2’-(mesitylmethylene)bis(1H-pyrrole) was 2.678 g, 37.5%.

^{1}H NMR (Fig. S1) (400 MHz, $CDCl_3$) δ 7.93 (s, 2H), 6.86 (s, 2H), 6.68 – 6.63 (m, 2H), 6.20 – 6.14 (m, 2H), 6.00 (s, 2H), 5.92 (s, 1H), 2.28 (s, 3H), 2.06 (s, 6H).

2.2 Synthesis of *N*,*N*’-bisformylphenoxyacetyl-4,13-diaza-18-crown-6-ether

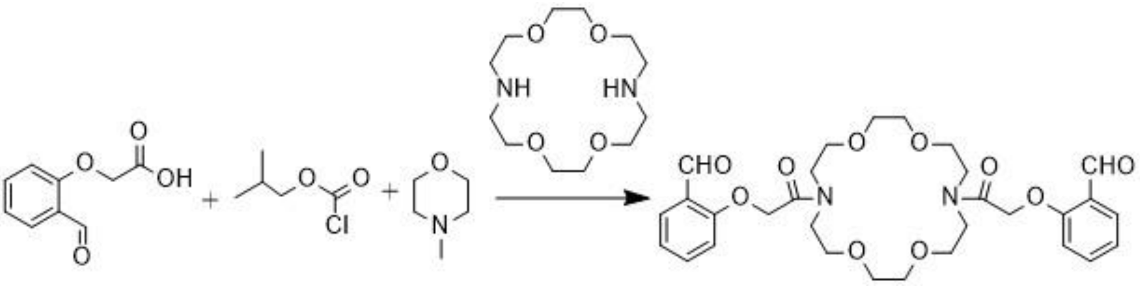

A Schlenk round-bottom flask of 100 mL with a stirring bar was filled with 50 mL of dry THF and 2-(2-formylphenoxy)acetic acid (369.1 mg, 2.15 eq, 2.049 mmol). The reaction mixture was cooled to −40 °C (in a dry ice bath), and then isobutyl chloroformate (312.4 mg, 2.4 eq, 2.287 mmol) and 4-methylmorpholine (231.3 mg, 2.4 eq, 2.287 mmol) were added slowly. The reaction mixture was stirred at −40 °C for two hours. A white solid appeared, and 1,4,10,13-tetraoxa-7,16-diazacyclooctadecane (250.0 mg, 1 eq, 952.9 μmol) was added, and stirring at −40 °C was continued for 15 min. After that, the reaction mixture was gradually heated up by not adding dry ice to the cooling bath and allowed to react overnight. The reaction mixture analysed using electrospray ionisation-mass spectrometry (ESI-MS) showed the mass disubstituted product (*N*,*N*'-bisformylphenoxyacetyl-4,13-diaza-18-crown-6-ether). The reaction mixture was evaporated to dryness and used directly for the next step.

ESI-MS (Fig. S2): *m/z* $[M+Na]^+$, $[C_{30}H_{38}N_2NaO_{10}]^+$ Calculated: 609.24, Observed: 609.40

2.3 Synthesis of crown-capped dimesityldiphenylporphyrin ($H_2(TPP^{Mes}Crown)$)

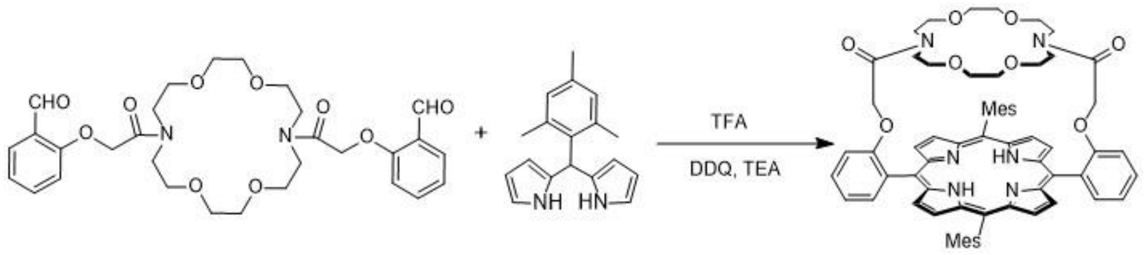

The *N*,*N*'-bisformylphenoxyacetyl 4,13-diaza-18-crown-6-ether reside from the previous step, and the mesityldipyrromethane (504 mg, 2 eq, 1.91 mmol) was dissolved in dichloromethane. The solution was sparged with nitrogen, and trifluoroacetic acid (65.2 mg, 0.6 eq, 572 μmol) was added. The solution was shielded from light and stirred overnight under nitrogen. Further, 2,3-dichloro-5,6-dicyano-1,4-benzoquinone (DDQ) (541 mg, 2.5 eq, 2.38 mmol) was added to the solution and left under stirring. After 1.5 hours, 3 mL triethylamine was added, and the solution was stirred for another 30 minutes. The reaction mixture was concentrated and purified over silica column chromatography. Two pre-fractions were collected using 1% methanol in dichloromethane as the eluent, and then methanol was increased to 2.5%, and a purple fraction containing the product was collected. The fraction was concentrated and further purified on a 60H silica column using 1.5% methanol in dichloromethane as the eluent. Finally, the purified fraction was concentrated and then precipitated with heptane. The solid was centrifuged and washed with pentane (four times) and dried to the yield crown-capped dimesityldiphenylporphyrin (290 mg, 270 μmol, 28% yield).

^1H NMR (Fig. S3) (400 MHz, $CDCl_3$) δ 8.76-8.69 (m, 8H), 8.18-8.16 (dd, J = 7.5, 1.7 Hz, 2H), 7.82-7.78 (m, 2H), 7.48-7.44 (td, J = 7.5, 1.0 Hz, 2H), 7.41-7.39 (d, J = 8.3 Hz, 2H), 7.32 (s, 2H), 7.24 (s, 2H), 4.65 (s, 4H), 2.92 (s, 3H), 2.69-2.59 (m, 14H), 2.02 (s, 6H), 1.94 (t, J = 4.8 Hz 4H), 1.66 (s, 6H), 1.53 (s, 2H), -0.04 (t, J = 4.7 Hz, 4H), -0.48 (s, 3H), -2.59 (s, 2H).

2.4 Synthesis of Fe(III)(crown-capped dimesityldiphenylporphyrin)OAc ([(TPP^{Mes}Crown)Fe^{III}(OAc)])

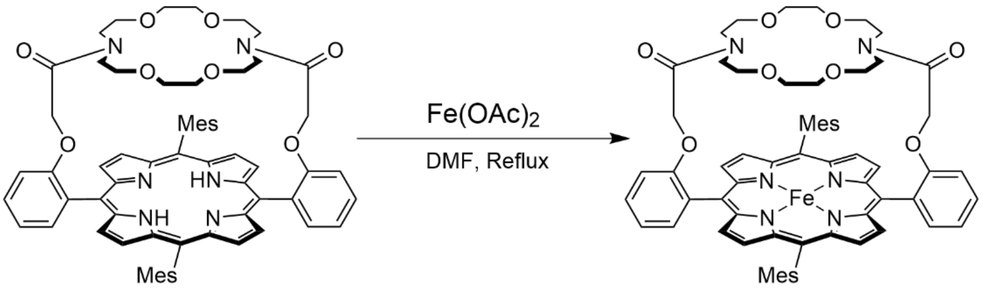

A round bottom flask was filled with 25 mL of deoxygenated dimethylformamide (DMF) containing H_2(TPP^{Mes}Crown) (100 mg, 1 eq, 93.2 µmol) and diacetoxyiron (89.1 mg, 5.5 eq, 512 µmol). The reaction mixture was shielded from light and brought to reflux (160 °C) under nitrogen for 3 hours. After cooling down, acetic acid was added and stirred for another one hour in the open air. Water (50 mL) was added to the reaction mixture and extracted with dichloromethane (5 × 35 mL). The combined dichloromethane layers were washed with water (5 × 50 mL). The dichloromethane layer was concentrated, and heptane was added. The dichloromethane was evaporated to yield the solid product. The solid was isolated by centrifuge and washed with pentane (4 × pentane/centrifuge), and dried to obtain [(TPP^{Mes}Crown)Fe^{III}(OAc)] (59.5 mg, 50.2 µmol, 53.8 % yield).

ESI-MS (TOF) (Fig. S4): [M+(Na$^+$)]$^+$, [(TPP^{Mes}Crown)Fe^{III}(OAc)(Na)]$^+$ m/z calculated 1208.4317, m/z observed 1208.4306; [M-(OAc)]$^+$, [(TPP^{Mes}Crown)Fe^{III}]$^+$ m/z caculated 1126.4292, m/z observed 1126.4299.

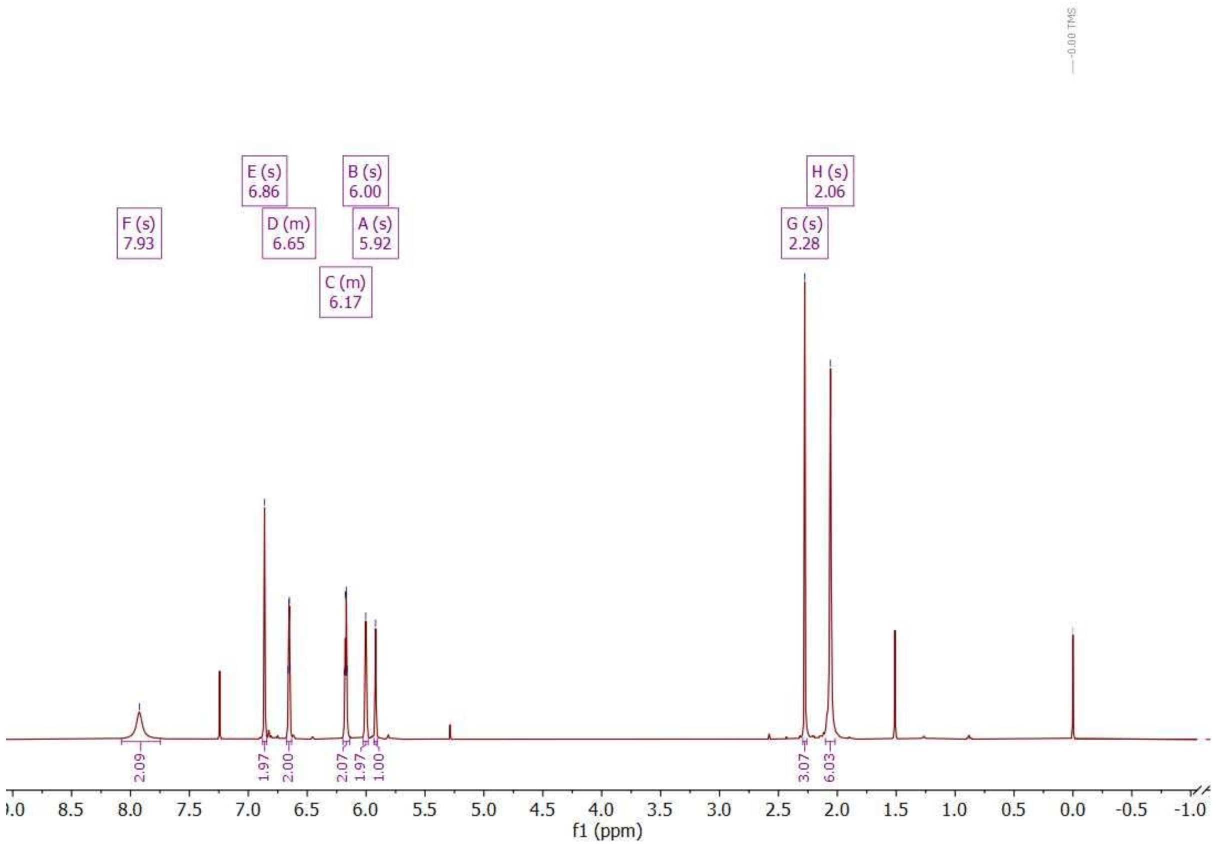

Fig. S1. ^{1}H NMR spectrum of mesityldipyrromethane (400 MHz, $CDCl_3$).

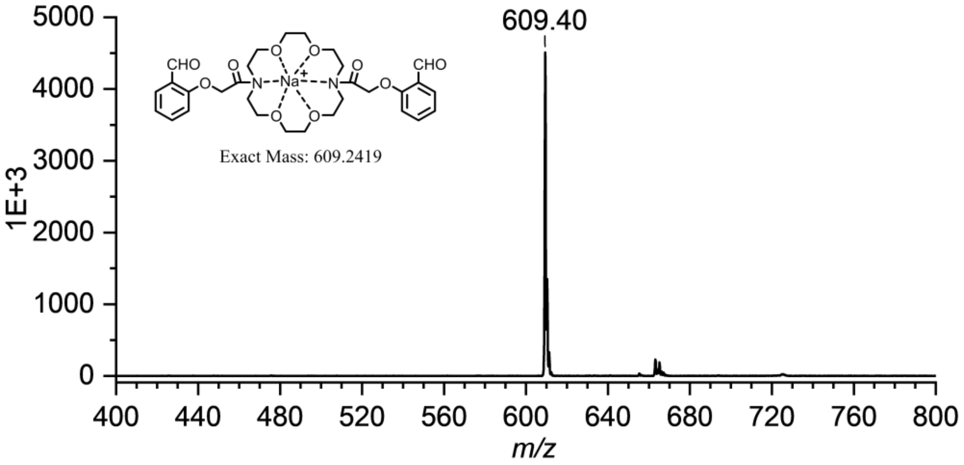

Fig. S2. ESI-MS (Thermo Finnigan LCQ Deca) mass spectrum of *N*,*N*'-bisformylphenoxyacetyl-4,13-diaza-18-crown-6-ether $[M+Na]^+$ = m/z 609.40.

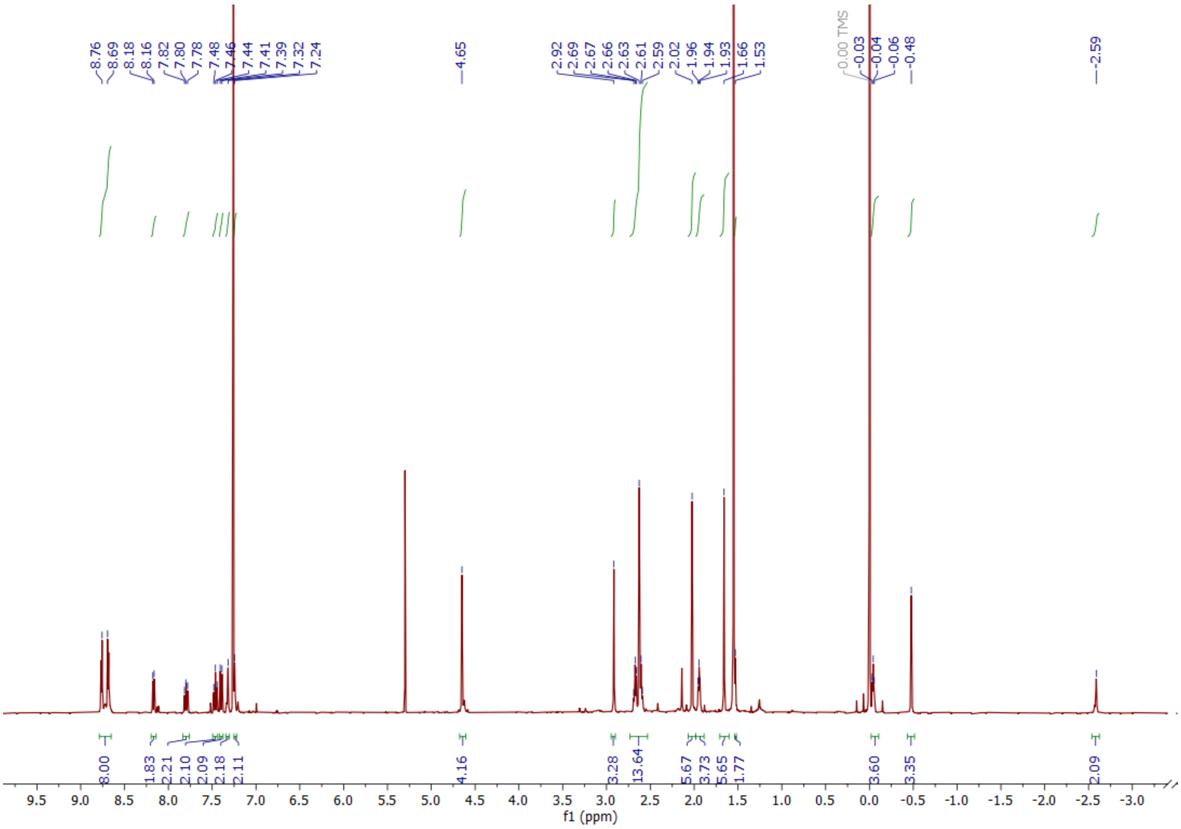

Fig. S3. ^{1}H NMR spectrum of H_2(TPP^{Mes}Crown) (400 MHz, $CDCl_3$).

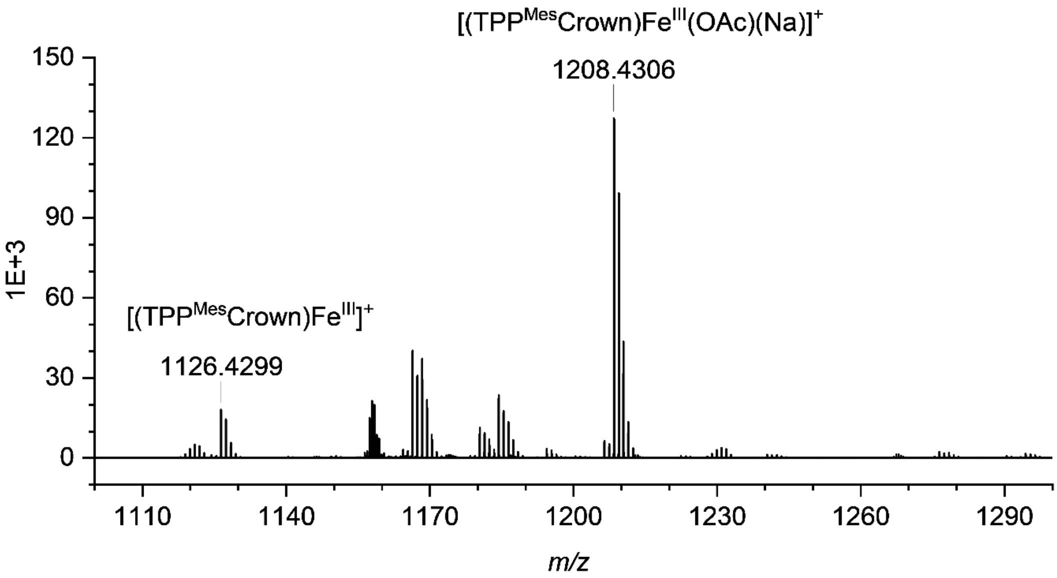

Fig. S4. ESI-MS (Bruker, timsTOF) mass spectrum of [(TPP^{Mes}Crown)Fe^{III}(OAc)] in the presence of $NaBF_4$ salt, detected as [(TPP^{Mes}Crown)Fe^{III}(OAc)(Na)]$^+$: *m/z* 1208.4306 and [(TPP^{Mes}Crown)Fe^{III}]$^+$: *m/z* 1126.4299.

Table S1: Absolute energies and free energies (in au) for UB3LYP-GD3BJ/BS1 optimized geometries for CO_2 reduction on an iron-porphyrin center with/without nearby K^+ using H_3O^+, phenol or acetate as a proton source.

	E [au, BS1]	ZPE	G	E [au,BS2]
**H_3O^+**				
^{2}**CAT**$_{H3O+}$	-4587.404689	1.066504	-4586.435419	-4591.188919
^{3}**CAT**$_{H3O+}$	-4587.490629	1.062391	-4586.525911	-4591.278140
^{3}**RC1**$_{H3O+}$	-4852.772171	1.114180	-4851.760795	-4856.868489
^{3}**IM1**$_{H3O+}$	-4852.800085	1.115876	-4851.789252	-4856.903592
^{3}**RC2**$_{H3O+}$	-4853.223192	1.126182	-4852.202849	-4857.320692
^{3}**IM2**$_{H3O+}$	-4853.290896	1.125608	-4852.274494	-4857.396762
^{4}**CAT**$_{H3O+}$	-4587.391863	1.066022	-4586.423664	-4591.176653
^{5}**CAT**$_{H3O+}$	-4587.471954	1.063349	-4586.506096	-4591.259860
^{5}**RC1**$_{H3O+}$	-4852.764580	1.112011	-4851.755962	-4856.857424
^{5}**IM1**$_{H3O+}$	-4852.788577	1.114669	-4851.781143	-4856.889837
^{5}**RC2**$_{H3O+}$	-4853.225846	1.124544	-4852.209623	-4857.323323
^{5}**IM2**$_{H3O+}$	-4853.283947	1.123434	-4852.270961	-4857.386909
^{2}**CAT**$_{H3O+,K+}$	-5187.265604	1.067524	-5186.298284	-5191.110762
^{3}**CAT**$_{H3O+,K+}$	-5187.354082	1.065355	-5186.391690	-5191.203501
^{3}**RC1**$_{H3O+,K+}$	-5452.626611	1.116194	-5451.614907	-5456.782012
^{3}**IM1**$_{H3O+,K+}$	-5452.648184	1.116736	-5451.639015	-5456.810510
^{3}**RC2**$_{H3O+,K+}$	-5453.068123	1.126790	-5452.049449	-5457.225416
^{3}**TS2**$_{H3O+,K+}$	-5453.076019	1.126001	-5452.057176	-5457.231028
^{3}**IM2**$_{H3O+,K+}$	-5453.140882	1.126912	-5452.123957	-5457.308372
^{4}**CAT**$_{H3O+,K+}$	-5187.261996	1.067565	-5186.294064	-5191.10681
^{5}**CAT**$_{H3O+,K+}$	-5187.350117	1.064721	-5186.385416	-5191.197633
^{5}**RC1**$_{H3O+,K+}$	-5452.613576	1.113642	-5451.606889	-5456.768711
^{5}**IM1**$_{H3O+,K+}$	-5452.651480	1.115433	-5451.643982	-5456.812878
^{5}**RC2**$_{H3O+,K+}$	-5453.069007	1.125570	-5452.050611	-5457.225974
^{5}**TS2**$_{H3O+,K+}$	-5453.086164	1.124552	-5452.067475	-5457.241761
^{5}**IM2**$_{H3O+,K+}$	-5453.137093	1.125293	-5452.122943	-5457.302426
**phenol**				
^{3}**RC1**$_{phenol,K+}$	-5683.105971	1.185644	-5682.031202	-5687.507107
^{5}**RC1**$_{phenol,K+}$	-5683.092219	1.183377	-5682.023243	-5687.492791
^{3}**RC1**$_{phenol, 6W, K+}$	-6065.094394	1.313519	-6063.903983	-6069.958867
^{5}**RC1**$_{phenol, 6W, K+}$	-6065.037907	1.310238	-6063.853744	-6069.902873
**acetate**				
^{3}**RC1**$_{acetate,K+}$	-5604.752623	1.142108	-5603.720928	-5609.087443
^{5}**RC1**$_{acetate,K+}$	-5604.739781	1.140133	-5603.710740	-5609.072379
^{5}**TS1**$_{acetate,K+}$	-5604.718308	1.135460	-5603.692740	-5609.054274
^{5}**IM1**$_{acetate,K+}$	-5604.735224	1.140103	-5603.705841	-5609.075897
^{5}**RC2**$_{acetate,K+}$	-5605.217797	1.153260	-5604.177196	-5609.549445
^{5}**TS2**$_{acetate,K+}$	-5605.221445	1.147683	-5604.188925	-5609.553966
^{5}**IM2**$_{acetate,K+}$	-5605.241660	1.149963	-5604.206575	-5609.575401
^{3}**RC1**$_{Ac, 6W, K+}$	-5986.705409	1.267449	-5985.558433	-5991.501598
^{3}**TS1**$_{acetate, 6W, K+}$	-5986.703255	1.264086	-5985.558928	-5991.498899
^{3}**IM1**$_{acetate, 6W, K+}$	-5986.707065	1.268557	-5985.558978	-5991.505482
^{3}**RC2**$_{acetate, 6W, K+}$	-5987.170947	1.282746	-5986.010089	-5991.962601
^{3}**IM2**$_{acetate, 6W, K+}$	-5987.211491	1.280719	-5986.054804	-5992.014555
^{5}**RC1**$_{acetate, 6W, K+}$	-5986.684029	1.266321	-5985.538967	-5991.482361
^{5}**TS1**$_{acetate, 6W, K+}$	-5986.649671	1.262808	-5985.507400	-5991.445074
^{5}**IM1**$_{acetate, 6W, K+}$	-5986.652342	1.267019	-5985.506514	-5991.450036
^{5}**RC2**$_{acetate, 6W, K+}$	-5987.116616	1.279820	-5985.959074	-5991.909516

Table S2: Relative (free) energies (in kcal mol^{-1}) of the CO_2 reduction on an iron-porphyrin center with/without nearby K^+ using H_3O^+, phenol or acetate as a proton source as obtained with UB3LYP-GD3BJ in Gaussian-09.

	ΔE(BS1)	ΔE+ZPE(BS1)	ΔG(BS1)	ΔE(BS2)	ΔE+ZPE(BS2)	ΔG(BS2)
**H_3O^+**						
^{2}**CAT**$_{H3O+}$	53.93	56.51	56.78	55.99	58.57	58.84
^{3}**CAT**$_{H3O+}$	0.00	0.00	0.00	0.00	0.00	0.00
^{3}**RC1**$_{H3O+}$	0.00	0.00	0.00	0.00	0.00	0.00
^{3}**IM1**$_{H3O+}$	-17.52	-16.45	-17.86	-22.03	-20.96	-22.37
^{3}**RC2**$_{H3O+}$	-17.52	-16.45	-17.86	-22.03	-20.96	-22.37
^{3}**IM2**$_{H3O+}$	-60.00	-59.30	-62.82	-69.76	-69.06	-72.58
^{4}**CAT**$_{H3O+}$	55.02	55.34	54.76	59.16	59.47	58.89
^{5}**CAT**$_{H3O+}$	4.76	3.40	3.03	6.94	5.58	5.21
^{5}**RC1**$_{H3O+}$	4.76	3.40	3.03	6.94	5.58	5.21
^{5}**IM1**$_{H3O+}$	-10.30	-9.99	-12.77	-13.40	-13.09	-15.87
^{5}**RC2**$_{H3O+}$	-10.30	-9.99	-12.77	-13.40	-13.09	-15.87
^{5}**IM2**$_{H3O+}$	-46.75	-47.14	-51.26	-53.30	-53.69	-57.80
^{2}**CAT**$_{H3O+,K+}$	55.52	56.88	58.61	58.19	59.56	61.29
^{3}**CAT**$_{H3O+,K+}$	0.00	0.00	0.00	0.00	0.00	0.00
^{3}**RC1**$_{H3O+,K+}$	0.00	0.00	0.00	0.00	0.00	0.00
^{3}**IM1**$_{H3O+,K+}$	-13.54	-13.20	-15.13	-17.88	-17.54	-19.47
^{3}**RC2**$_{H3O+,K+}$	-13.54	-13.20	-15.13	-17.88	-17.54	-19.47
^{3}**TS2**$_{H3O+,K+}$	-18.49	-18.65	-19.98	-21.40	-21.56	-22.89
^{3}**IM2**$_{H3O+,K+}$	-59.19	-58.78	-61.88	-69.94	-69.52	-72.63
^{4}**CAT**$_{H3O+,K+}$	63.48	63.66	62.36	65.34	65.52	64.22
^{5}**CAT**$_{H3O+,K+}$	8.18	6.58	5.03	8.35	6.75	5.20
^{5}**RC1**$_{H3O+,K+}$	8.18	6.58	5.03	8.35	6.75	5.20
^{5}**IM1**$_{H3O+,K+}$	-15.61	-16.08	-18.24	-19.37	-19.85	-22.01
^{5}**RC2**$_{H3O+,K+}$	-15.61	-16.08	-18.24	-19.37	-19.85	-22.01
^{5}**TS2**$_{H3O+,K+}$	-26.37	-27.49	-28.83	-29.28	-30.39	-31.73
^{5}**IM2**$_{H3O+,K+}$	-58.33	-58.98	-63.63	-67.34	-68.00	-72.65
**phenol**						
^{3}**RC1**$_{phenol,K+}$	0.00	0.00	0.00	0.00	0.00	0.00
^{5}**RC1**$_{phenol,K+}$	8.63	7.21	4.99	8.98	7.56	5.35
^{3}**RC1**$_{phenol, 6W, K+}$	0.00	0.00	0.00	0.00	0.00	0.00
^{5}**RC1**$_{phenol, 6W, K+}$	35.45	33.39	31.53	35.14	33.08	31.22
**acetate**						
^{3}**RC1**$_{acetate,K+}$	0.00	0.00	0.00	0.00	0.00	0.00
^{5}**RC1**$_{acetate,K+}$	8.06	6.82	6.39	9.45	8.21	7.79
^{5}**TS1**$_{acetate,K+}$	21.53	17.36	17.69	20.81	16.64	16.97
^{5}**IM1**$_{acetate,K+}$	10.92	9.66	9.47	7.24	5.99	5.79
^{5}**RC2**$_{acetate,K+}$	10.92	9.66	9.47	7.24	5.99	5.79
^{5}**TS2**$_{acetate,K+}$	8.63	3.87	2.11	4.41	-0.35	-2.11
^{5}**IM2**$_{acetate,K+}$	-4.06	-7.38	-8.97	-9.04	-12.37	-13.96
^{3}**RC1**$_{acetate, 6W, K+}$	0.00	0.00	0.00	0.00	0.00	0.00
^{3}**TS1**$_{acetate, 6W, K+}$	1.35	-0.76	-0.31	1.69	-0.42	0.03
^{3}**IM1**$_{acetate, 6W, K+}$	-1.04	-0.34	-0.34	-2.44	-1.74	-1.74
^{3}**RC2**$_{acetate, 6W, K+}$	-1.04	-0.34	-0.34	-2.44	-1.74	-1.74
^{3}**IM2**$_{acetate, 6W, K+}$	-26.48	-27.06	-28.40	-35.04	-35.62	-36.96
^{5}**RC1**$_{acetate, 6W, K+}$	13.42	12.71	12.22	12.07	11.36	10.87
^{5}**TS1**$_{acetate, 6W, K+}$	34.98	32.06	32.02	35.47	32.56	32.52
^{5}**IM1**$_{acetate, 6W, K+}$	33.30	33.03	32.58	32.36	32.09	31.64
^{5}**RC2**$_{acetate, 6W, K+}$	33.30	33.03	32.58	32.36	32.09	31.64

Table S3: Group spin densities (a) and charges (b) of UB3LYP-GD3BJ/BS1 optimized geometries for the CO_2 reduction on an iron-porphyrin center with/without nearby K^+ using H_3O^+, phenol or acetate as a proton source as obtained in Gaussian-09.

a Spin densities

	H_3O^+	CO_2(H)	Fe	Lig	total
**H_3O^+**					
^{2}**CAT**$_{H3O+}$			1.99	-0.99	1.00
^{3}**CAT**$_{H3O+}$			2.00	0.00	2.00
^{3}**RC1**$_{H3O+}$	0.00	-0.18	1.26	0.92	2.00
^{3}**IM1**$_{H3O+}$	0.00	0.15	2.27	-0.42	2.00
^{3}**RC2**$_{H3O+}$	0.00	0.15	2.31	-0.47	2.00
^{3}**IM2**$_{H3O+}$	0.00	0.03	2.07	-0.10	2.00
^{4}**CAT**$_{H3O+}$			2.17	0.83	3.00
^{5}**CAT**$_{H3O+}$			2.15	1.85	4.00
^{5}**RC1**$_{H3O+}$	0.00	-0.51	3.40	1.11	4.00
^{5}**IM1**$_{H3O+}$	0.00	0.35	2.64	1.01	4.00
^{5}**RC2**$_{H3O+}$	0.00	0.08	3.87	0.04	4.00
^{5}**IM2**$_{H3O+}$	0.00	0.00	3.81	0.19	4.00
^{2}**CAT**$_{H3O+,K+}$			1.98	-0.98	1.00
^{3}**CAT**$_{H3O+,K+}$			1.99	0.01	2.00
^{3}**RC1**$_{H3O+,K+}$	0.01	-0.16	1.27	0.89	2.00
^{3}**IM1**$_{H3O+,K+}$	0.00	0.12	2.09	-0.21	2.00
^{3}**RC2**$_{H3O+,K+}$	0.00	0.13	2.20	-0.33	2.00
^{3}**TS2**$_{H3O+,K+}$	0.01	-0.06	1.53	0.52	2.00
^{3}**IM2**$_{H3O+,K+}$	0.00	0.05	2.06	-0.11	2.00
^{4}**CAT**$_{H3O+,K+}$			2.04	0.96	3.00
^{5}**CAT**$_{H3O+,K+}$			2.05	1.95	4.00
^{5}**RC1**$_{H3O+,K+}$	0.01	-0.49	3.40	1.08	4.00
^{5}**IM1**$_{H3O+,K+}$	0.00	0.06	3.83	0.11	4.00
^{5}**RC2**$_{H3O+,K+}$	0.00	0.06	3.85	0.09	4.00
^{5}**TS2**$_{H3O+,K+}$	0.00	0.02	3.83	0.15	4.00
^{5}**IM2**$_{H3O+,K+}$	0.00	-0.02	3.81	0.21	4.00
**acetate**					
^{5}**RC1**$_{acetate,K+}$	0.00	-0.44	3.31	1.13	4.00
^{5}**TS1**$_{acetate,K+}$	0.00	-0.34	3.21	1.13	4.00
^{5}**IM1**$_{acetate,K+}$	0.00	0.06	3.85	0.09	4.00
^{5}**RC2**$_{acetate,K+}$	0.00	0.05	3.84	0.11	4.00
^{5}**TS2**$_{acetate,K+}$	0.00	0.02	3.80	0.18	4.00
^{5}**IM2**$_{acetate,K+}$	0.00	0.00	3.80	0.20	4.00
^{3}**RC1**$_{acetate, 6W, K+}$	0.00	-0.17	1.25	0.91	2.00
^{3}**TS1**$_{acetate, 6W, K+}$	0.00	-0.14	1.21	0.93	2.00
^{3}**IM1**$_{acetate, 6W, K+}$	0.00	-0.12	1.18	0.94	2.00
^{3}**RC2**$_{acetate, 6W, K+}$	0.00	-0.15	1.24	0.91	2.00
^{3}**IM2**$_{acetate, 6W, K+}$	0.00	0.04	2.06	-0.10	2.00
^{5}**RC1**$_{acetate, 6W, K+}$	0.00	0.41	2.56	1.03	4.00
^{5}**TS1**$_{acetate, 6W, K+}$	0.00	-0.21	1.40	2.80	4.00
^{5}**IM1**$_{acetate, 6W, K+}$	0.00	-0.16	1.29	2.86	4.00

b Charges

	H_3O^+	CO_2(H)	Fe	Lig	total
**H_3O^+:**					
^{2}**CAT**$_{H3O+}$			0.55	-1.55	-1.00
^{3}**CAT**$_{H3O+}$			0.48	-2.48	-2.00
^{3}**RC1**$_{H3O+}$	0.72	-0.53	0.45	-1.65	-1.00
^{3}**IM1**$_{H3O+}$	0.22	-0.56	0.56	-1.22	-1.00
^{3}**RC2**$_{H3O+}$	0.74	-0.32	0.57	-0.99	0.00
^{3}**IM2**$_{H3O+}$	0.22	-0.09	0.60	-0.73	0.00
^{4}**CAT**$_{H3O+}$			0.70	-1.70	-1.00
^{5}**CAT**$_{H3O+}$			0.65	-2.65	-2.00

**^{5}RC1 $_{H3O+}$**	0.72	-0.50	0.61	-1.82	-1.00
**^{5}IM1 $_{H3O+}$**	0.25	-0.47	0.62	-1.39	-1.00
**^{5}RC2 $_{H3O+}$**	0.74	-0.39	0.67	-1.03	0.00
**^{5}IM2 $_{H3O+}$**	0.27	-0.07	0.72	-0.92	0.00
**^{2}CAT$_{H3O+,K+}$**			0.55	-0.55	0.00
**^{3}CAT$_{H3O+,K+}$**			0.48	-1.48	-1.00
**^{3}RC1$_{H3O+,K+}$**	0.65	-0.51	0.47	-0.61	0.00
**^{3}IM1$_{H3O+,K+}$**	0.27	-0.55	0.55	-0.26	0.00
**^{3}RC2$_{H3O+,K+}$**	0.78	-0.31	0.59	-0.05	1.00
**^{3}TS2$_{H3O+,K+}$**	0.69	-0.11	0.49	-0.07	1.00
**^{3}IM2$_{H3O+,K+}$**	0.26	-0.07	0.61	0.20	1.00
**^{4}CAT$_{H3O+,K+}$**			0.58	-0.58	0.00
**^{5}CAT$_{H3O+,K+}$**			0.52	-1.52	-1.00
**^{5}RC1$_{H3O+,K+}$**	0.67	-0.48	0.62	-0.81	0.00
**^{5}IM1$_{H3O+,K+}$**	0.28	-0.58	0.66	-0.36	0.00
**^{5}RC2$_{H3O+,K+}$**	0.77	-0.32	0.68	-0.13	1.00
**^{5}TS2$_{H3O+,K+}$**	0.65	-0.28	0.68	-0.05	1.00
**^{5}IM2$_{H3O+,K+}$**	0.26	-0.05	0.72	0.07	1.00
**Acetate:**					
**^{5}RC1$_{acetate,K+}$**	-0.10	-0.46	0.57	-1.02	-1.00
**^{5}TS1$_{acetate,K+}$**	-0.38	-0.33	0.57	-0.86	-1.00
**^{5}IM1$_{acetate,K+}$**	-0.49	-0.64	0.65	-0.52	-1.00
**^{5}RC2$_{acetate,K+}$**	-0.01	-0.32	0.66	-0.32	0.00
**^{5}TS2$_{acetate,K+}$**	-0.07	-0.30	0.67	-0.30	0.00
**^{5}IM2$_{acetate,K+}$**	-0.36	-0.06	0.70	-0.28	0.00
**^{3}RC1$_{acetate, 6W, K+}$**	-0.03	-0.50	0.44	-0.91	-1.00
**^{3}TS1$_{acetate, 6W, K+}$**	-0.15	-0.43	0.44	-0.87	-1.00
**^{3}IM1$_{acetate, 6W, K+}$**	-0.34	-0.29	0.44	-0.82	-1.00
**^{3}RC2$_{acetate, 6W, K+}$**	0.05	-0.02	0.46	-0.49	0.00
**^{3}IM2$_{acetate, 6W, K+}$**	-0.47	-0.11	0.58	-0.01	0.00
**^{5}RC1$_{acetate, 6W, K+}$**	-0.05	-0.66	0.58	-0.87	-1.00
**^{5}TS1$_{acetate, 6W, K+}$**	-0.16	-0.39	0.46	-0.92	-1.00
**^{5}IM1$_{acetate, 6W, K+}$**	-0.33	-0.27	0.46	-0.86	-1.00

Table S4: Absolute energies and free energies (in au) for UB3LYP/BS1 optimized geometries for CO_2 reduction on an iron-porphyrin center with nearby K^+ using H_3O^+ as a proton source.

	E [au, BS1]	ZPE	G	E [au,BS2]
^{3}**CAT**$_{H3O+,K+}$	-5186.942526	1.057999	-5185.991308	-5190.801361
^{3}**RC1**$_{H3O+,K+}$	-5452.216713	1.111464	-5451.211828	-5456.379711
^{3}**IM1** $_{H3O+,K+}$	-5452.246950	1.112348	-5451.246724	-5456.416558
^{3}**RC2** $_{H3O+,K+}$	-5452.646236	1.122733	-5451.635105	-5456.810513
^{3}**TS2** $_{H3O+,K+}$	-5452.657027	1.121953	-5451.646329	-5456.818193
^{3}**IM2** $_{H3O+,K+}$	-5452.715820	1.122009	-5451.709326	-5456.887664
^{5}**CAT**$_{H3O+,K+}$	-5186.934014	1.059161	-5185.981996	-5190.792326
^{5}**RC1** $_{H3O+,K+}$	-5452.210413	1.109122	-5451.210337	-5456.372956
^{5}**IM1** $_{H3O+,K+}$	-5452.253399	1.110935	-5451.254863	-5456.421439
^{5}**RC2** $_{H3O+,K+}$	-5452.650026	1.120547	-5451.644121	-5456.813508
^{5}**TS2** $_{H3O+,K+}$	-5452.665182	1.119849	-5451.657255	-5456.826934
^{5}**IM2** $_{H3O+,K+}$	-5452.712124	1.120077	-5451.708877	-5456.881115

Table S5: Relative (free) energies (in kcal mol^{-1}) of the CO_2 reduction on an iron-porphyrin center with nearby K^+ using H_3O^+ as a proton source as obtained with UB3LYP in Gaussian-09.

	ΔE(BS1)	ΔE+ZPE	ΔG(BS1)	ΔE(BS2)	ΔE+ZPE	ΔG(BS2)
^{3}**RC1** $_{H3O+,K+}$	0.00	0.00	0.00	0.00	0.00	0.00
^{3}**IM1** $_{H3O+,K+}$	-18.97	-18.42	-21.90	-23.12	-22.57	-26.05
^{3}**RC2** $_{H3O+,K+}$	-18.97	-18.42	-21.90	-23.12	-22.57	-26.05
^{3}**TS2** $_{H3O+,K+}$	-25.75	-25.68	-28.94	-27.94	-27.88	-31.14
^{3}**IM2** $_{H3O+,K+}$	-62.64	-62.54	-68.47	-71.54	-71.43	-77.37
^{5}**RC1** $_{H3O+,K+}$	3.95	2.48	0.94	4.24	2.77	1.22
^{5}**IM1** $_{H3O+,K+}$	-23.02	-23.35	-27.00	-26.18	-26.52	-30.17
^{5}**RC2** $_{H3O+,K+}$	-23.02	-23.35	-27.00	-26.18	-26.52	-30.17
^{5}**TS2** $_{H3O+,K+}$	-32.53	-33.30	-35.25	-34.61	-35.38	-37.32
^{5}**IM2** $_{H3O+,K+}$	-61.99	-62.61	-67.64	-68.61	-69.24	-74.26

Table S6: Group spin densities (a) and charges (b) of UB3LYP/BS1 optimized geometries for the CO_2 reduction on an iron-porphyrin center with nearby K^+ using H_3O^+ as a proton source as obtained in Gaussian-09.

a Spin densities

	H_3O^+	CO_2(H)	Fe	Lig	total
^{3}**CAT**$_{H3O+,K+}$			2.00	0.00	2.00
^{3}**RC1**$_{H3O+,K+}$	0.00	-0.21	1.31	0.89	2.00
^{3}**IM1**$_{H3O+,K+}$	0.00	0.10	2.14	-0.24	2.00
^{3}**RC2**$_{H3O+,K+}$	0.00	0.12	2.17	-0.28	2.00
^{3}**TS2**$_{H3O+,K+}$	0.00	-0.08	1.46	0.62	2.00
^{3}**IM2**$_{H3O+,K+}$	0.00	0.04	2.08	-0.12	2.00
^{5}**CAT**$_{H3O+,K+}$			2.05	1.95	4.00
^{5}**RC1**$_{H3O+,K+}$	0.00	-0.54	3.47	1.07	4.00
^{5}**IM1**$_{H3O+,K+}$	0.00	0.04	3.85	0.11	4.00
^{5}**RC2**$_{H3O+,K+}$	0.00	0.05	3.85	0.10	4.00
^{5}**TS2**$_{H3O+,K+}$	0.00	0.01	3.83	0.16	4.00
^{5}**IM2**$_{H3O+,K+}$	0.00	-0.01	3.82	0.20	4.00

b Charges

	H_3O^+	CO_2(H)	Fe	Lig	total
^{3}**CAT**$_{H3O+,K+}$			0.51	-1.51	-1.00
^{3}**RC1**$_{H3O+,K+}$	0.67	-0.53	0.48	-0.62	0.00
^{3}**IM1**$_{H3O+,K+}$	0.27	-0.60	0.58	-0.25	0.00
^{3}**RC2**$_{H3O+,K+}$	0.76	-0.34	0.60	-0.02	1.00
^{3}**TS2**$_{H3O+,K+}$	0.70	-0.09	0.50	-0.11	1.00
^{3}**IM2**$_{H3O+,K+}$	0.24	-0.09	0.66	0.19	1.00
^{5}**CAT**$_{H3O+,K+}$			0.53	-1.53	-1.00
^{5}**RC1**$_{H3O+,K+}$	0.68	-0.52	0.65	-0.81	0.00
^{5}**IM1**$_{H3O+,K+}$	0.27	-0.62	0.69	-0.34	0.00
^{5}**RC2**$_{H3O+,K+}$	0.76	-0.35	0.70	-0.12	1.00
^{5}**TS2**$_{H3O+,K+}$	0.64	-0.31	0.70	-0.03	1.00
^{5}**IM2**$_{H3O+,K+}$	0.25	-0.05	0.73	0.06	1.00

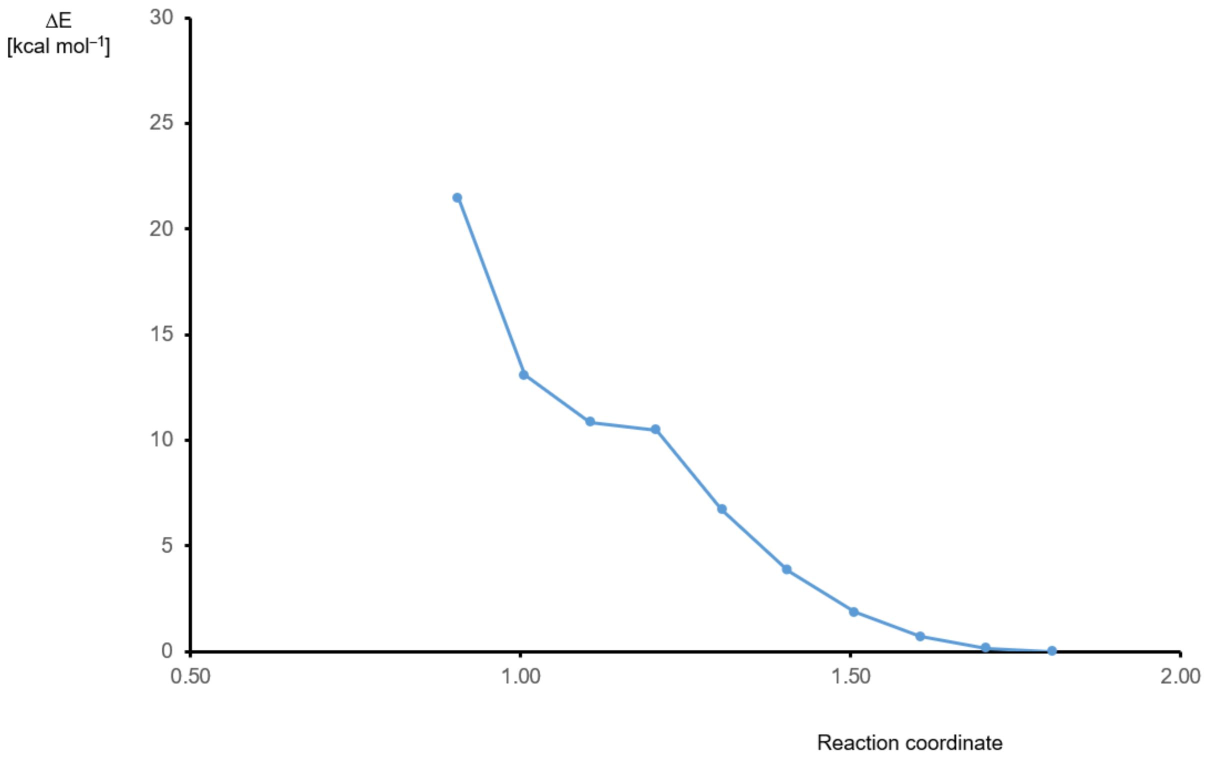

Fig. S5: UB3LYP-GD3BJ /BS1 calculated geometry scan for the O–H distance starting from ^{3}IM1$_{H3O+}$ on the right-hand-side to ^{3}RC1$_{H3O+}$. As can be seen no proton transfer barrier is encountered for the pathway from ^{3}RC1$_{H3O+}$ to ^{3}IM1$_{H3O+}$.

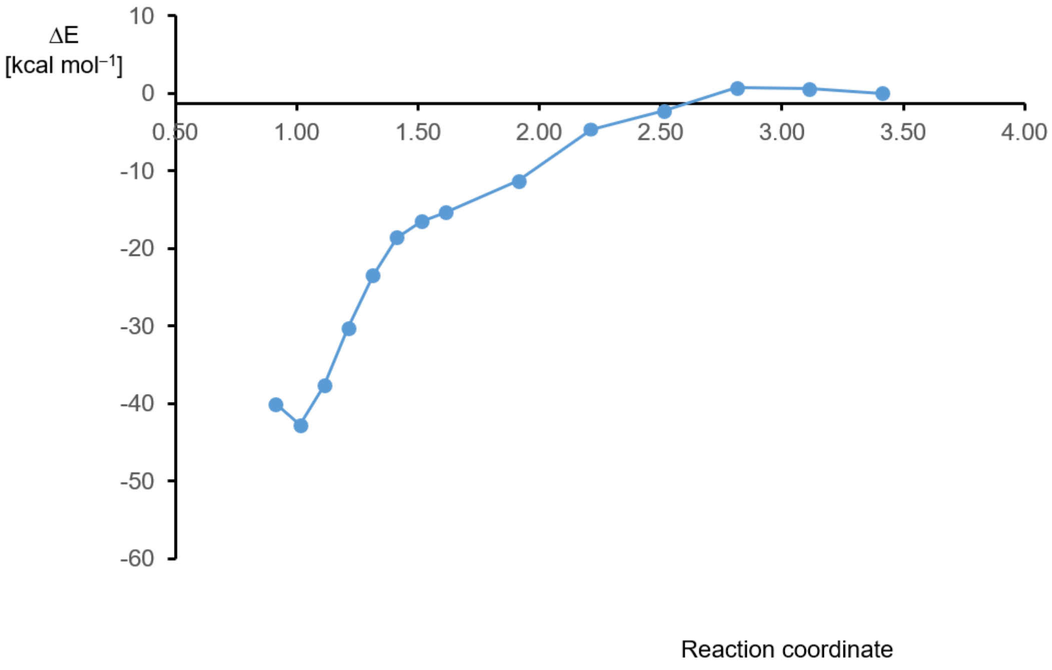

Fig. S6: UB3LYP-GD3BJ /BS1 calculated geometry scan for the O–H distance starting from ^{3}RC2$_{H3O+}$ on the right-hand-side to ^{3}IM2$_{H3O+}$. As can be seen a negligible proton transfer barrier is encountered for the pathway from ^{3}RC2$_{H3O+}$ to ^{3}IM2$_{H3O+}$.

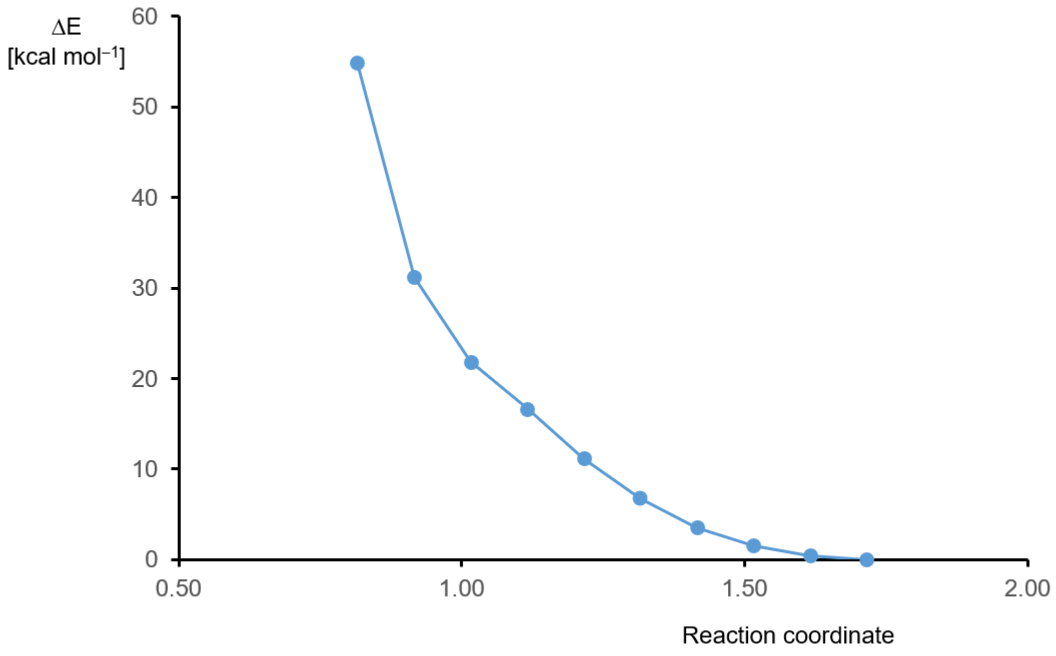

Fig. S7: UB3LYP-GD3BJ /BS1 calculated geometry scan for the O–H distance starting from $^5IM1_{H3O+}$ on the right-hand-side to $^5RC1_{H3O+}$. As can be seen no proton transfer barrier is encountered for the pathway from $^5RC1_{H3O+}$ to $^5IM1_{H3O+}$.

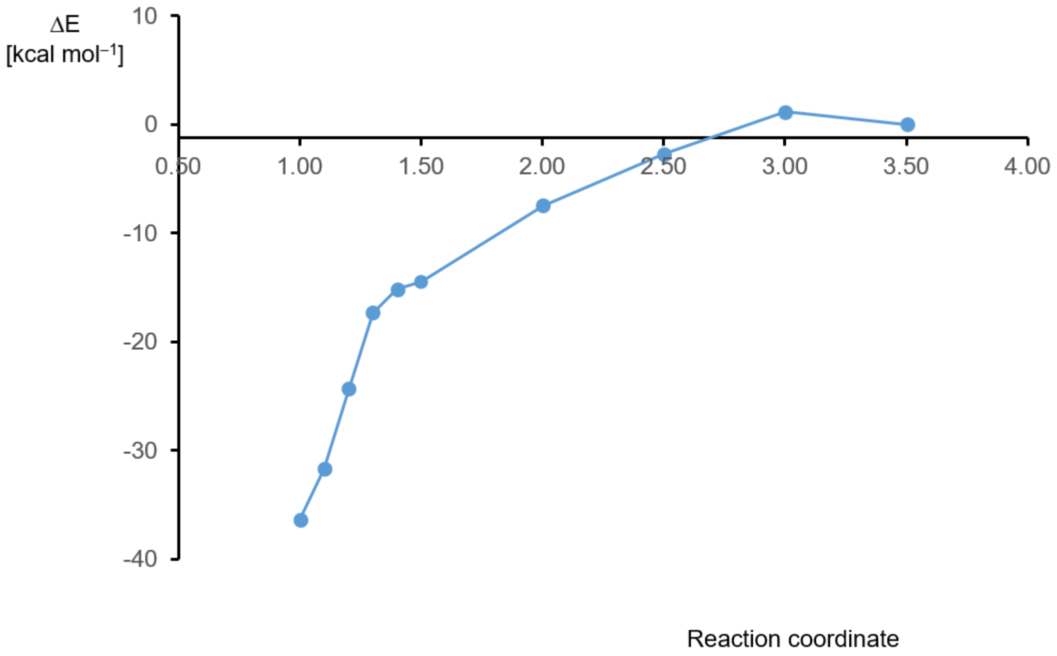

Fig. S8: UB3LYP-GD3BJ /BS1 calculated geometry scan for the O−H distance starting from $^5RC2_{H3O+}$ on the right-hand-side to $^5IM2_{H3O+}$. As can be seen a negligible proton transfer barrier is encountered for the pathway from $^5RC2_{H3O+}$ to $^5IM2_{H3O+}$.

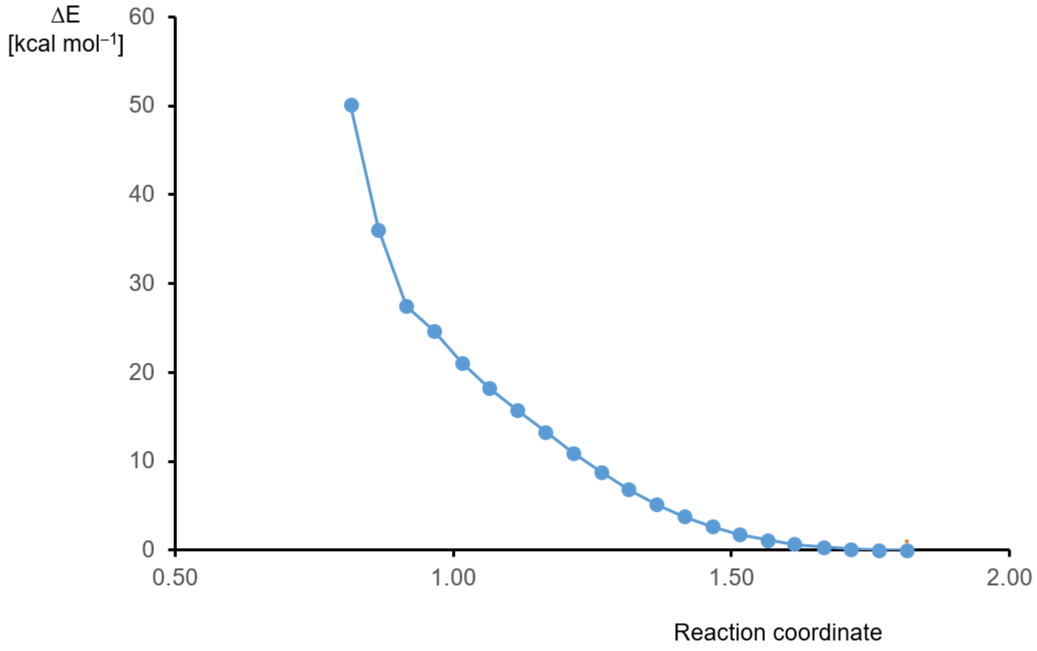

Fig. S9: UB3LYP-GD3BJ /BS1 calculated geometry scan for the O–H distance starting from $^{3}IM1_{H3O+,K+}$ on the right-hand-side to $^{3}RC1_{H3O+,K+}$. As can be seen no proton transfer barrier is encountered for the pathway from $^{3}RC1_{H3O+,K+}$ to $^{3}IM1_{H3O+,K+}$. (with nearby K^{+}).

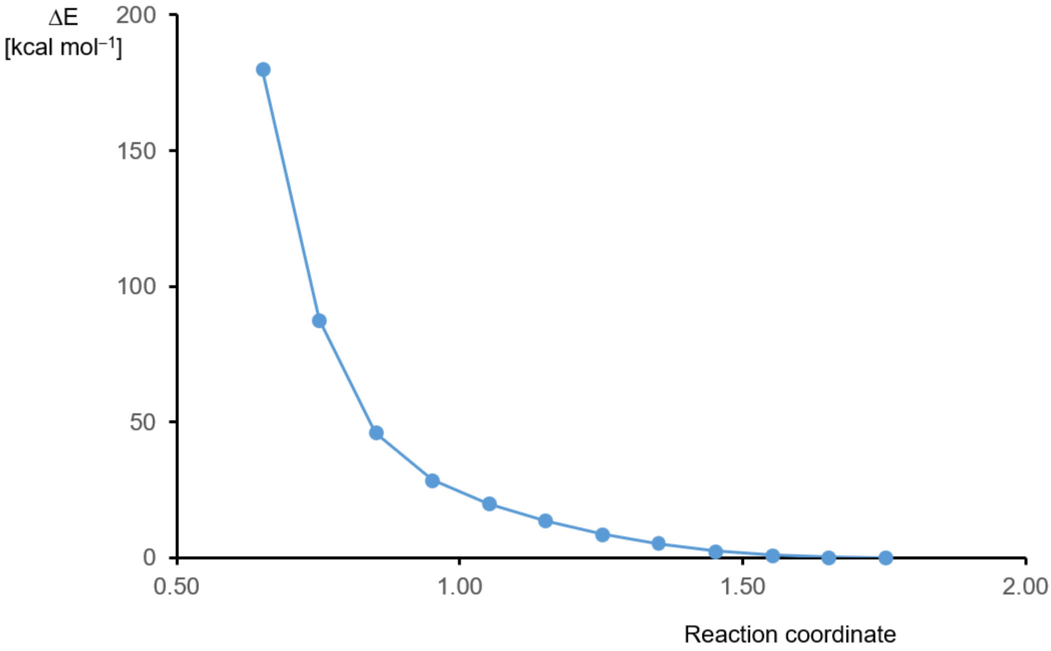

Fig. S10: UB3LYP-GD3BJ /BS1 calculated geometry scan for the O–H distance starting from $^{5}IM1_{H3O+,K+}$ on the right-hand-side to $^{5}RC1_{H3O+,K+}$. As can be seen no proton transfer barrier is encountered for the pathway from $^{5}RC1_{H3O+,K+}$ to $^{5}IM1_{H3O+,K+}$. (with nearby K^{+}).

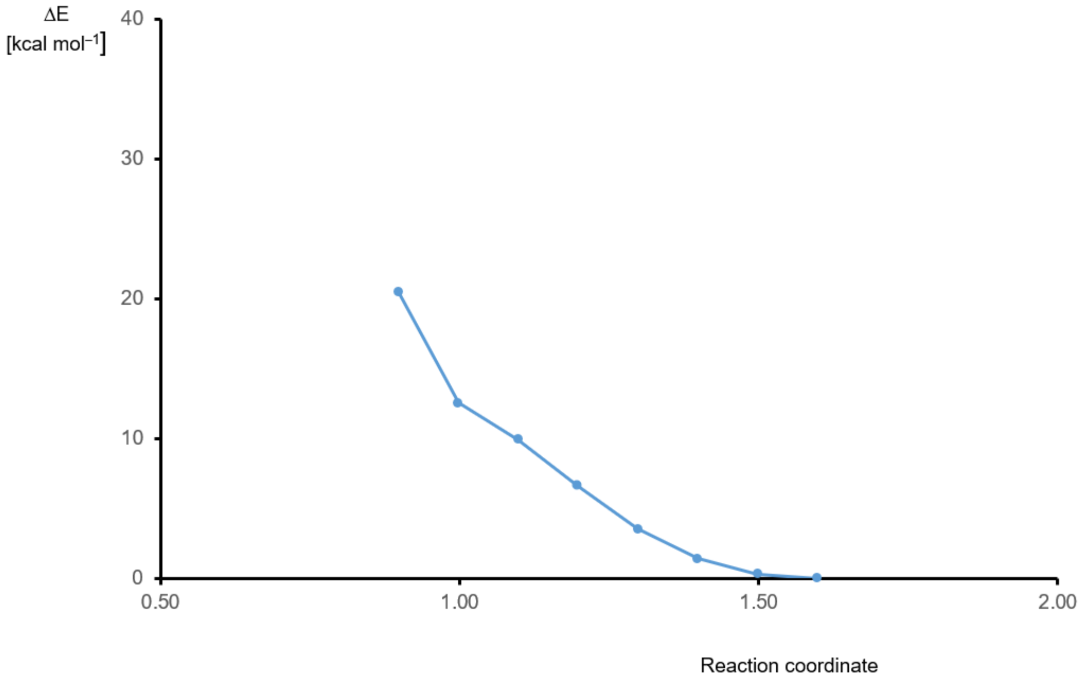

Fig. S11: UB3LYP-GD3BJ /BS1 calculated geometry scan for the O–H distance starting from ^{3}IM1$_{phenol,K+}$ on the right-hand-side to ^{3}RC1$_{phenol,K+}$. As can be seen no proton transfer barrier is encountered for the pathway from ^{3}RC1$_{phenol,K+}$ to ^{3}IM1$_{phenol,K+}$. (with nearby K^+).

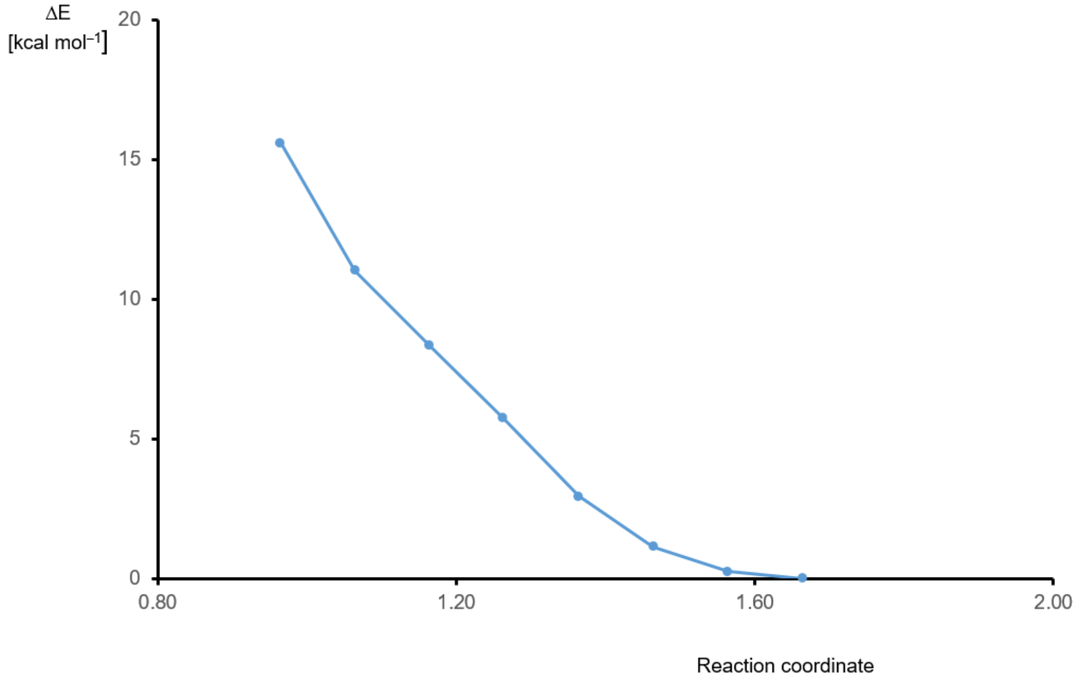

Fig. S12: UB3LYP-GD3BJ /BS1 calculated geometry scan for the O–H distance starting from ^{5}IM1$_{phenol,K+}$ on the right-hand-side to ^{5}RC1$_{phenol,K+}$. As can be seen no proton transfer barrier is encountered for the pathway from ^{5}RC1$_{phenol,K+}$ to ^{5}IM1$_{phenol,K+}$. (with nearby K^+).

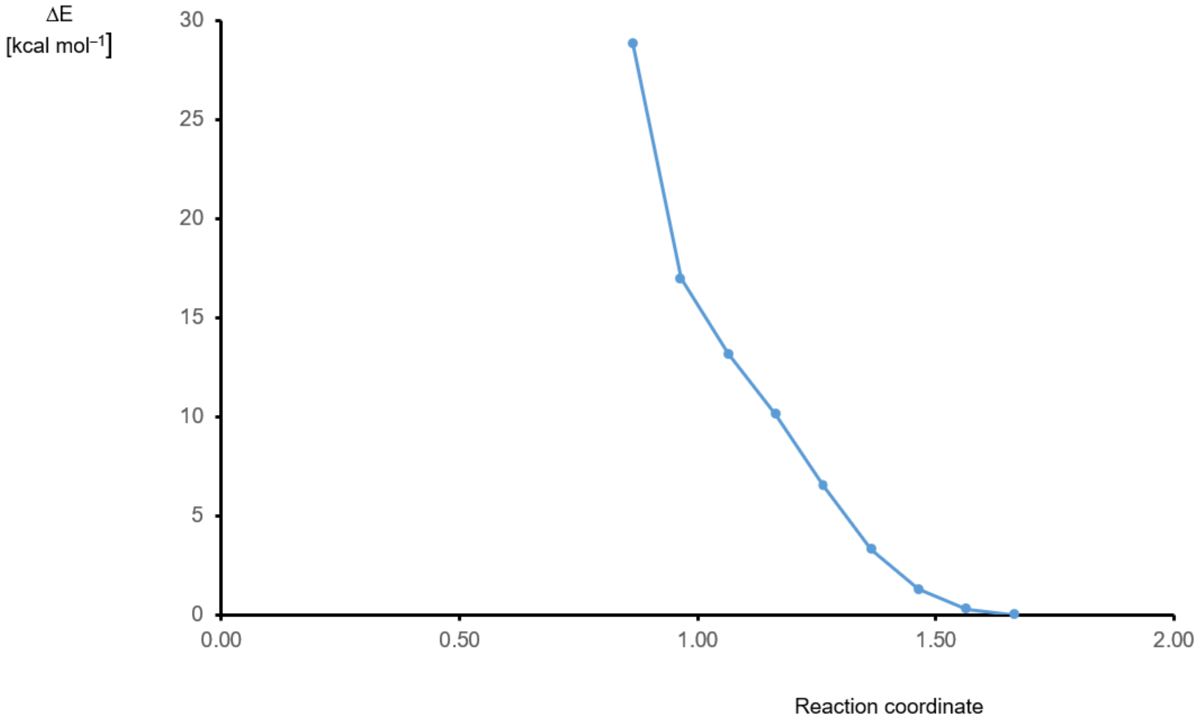

Fig. S13: UB3LYP-GD3BJ /BS1 calculated geometry scan for the O–H distance starting from ^{3}IM1$_{phenol, 6W, K+}$ on the right-hand-side to ^{3}RC1$_{phenol, 6W, K+}$. As can be seen no proton transfer barrier is encountered for the pathway from ^{3}RC1$_{phenol, 6W, K+}$ to ^{3}IM1$_{phenol, 6W, K+}$. (with nearby K^+).

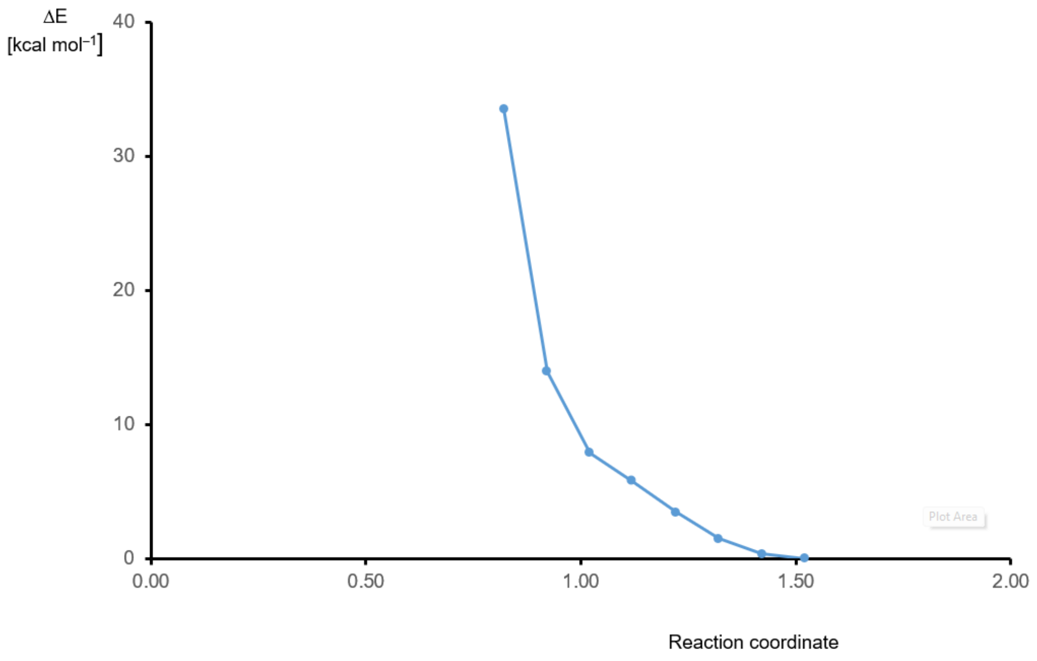

Fig. S14: UB3LYP-GD3BJ /BS1 calculated geometry scan for the O–H distance starting from ^{3}IM1$_{acetate,K+}$ on the right-hand-side to ^{3}RC1$_{acetate,K+}$. As can be seen no proton transfer barrier is encountered for the pathway from ^{3}RC1$_{acetate,K+}$ to ^{3}IM1$_{acetate,K+}$. (with nearby K^{+}).

Cartesian coordinates of optimized geometries:

CO_2 reduction on an iron-porphyrin center with/without nearby K^+

UB3LYP/GD3BJ:

$^{2}CAT_{H3O+}$:

6	3.911110000	3.755722000	-1.074495000
6	2.843461000	3.770836000	-1.993041000
6	2.782592000	4.813368000	-2.932994000
6	3.755427000	5.817567000	-2.954943000
6	4.808882000	5.794680000	-2.035983000
6	4.881900000	4.759681000	-1.096332000
6	1.803229000	2.703837000	-1.944282000
6	2.228640000	1.364380000	-2.122131000
7	1.471141000	0.239654000	-1.824249000
6	2.319411000	-0.838164000	-1.979478000
6	3.588465000	-0.402267000	-2.460627000
6	3.532041000	0.977261000	-2.547151000
26	-0.517416000	0.171098000	-1.573527000
7	-0.586416000	2.173897000	-1.552937000
6	-1.670258000	2.950524000	-1.164387000
6	-1.249554000	4.325728000	-0.966489000
6	0.073562000	4.387478000	-1.277316000
6	0.494630000	3.048594000	-1.640032000
6	-2.972434000	2.504931000	-0.998884000
6	-4.014752000	3.415805000	-0.450703000
6	-4.557059000	3.139452000	0.828753000
6	-5.561836000	3.947393000	1.372152000
6	-6.036702000	5.047523000	0.648620000
6	-5.508000000	5.343933000	-0.608955000
6	-4.505757000	4.527900000	-1.146328000
6	2.001345000	-2.182062000	-1.647565000
6	3.110285000	-3.174211000	-1.628432000
6	3.092312000	-4.286394000	-2.488583000
6	4.132953000	-5.216760000	-2.521588000
6	5.235909000	-5.041954000	-1.683423000
6	5.287762000	-3.944947000	-0.819593000
6	4.236116000	-3.020839000	-0.783823000
8	4.240647000	-1.962273000	0.088325000
6	5.475595000	-1.296267000	0.305761000
6	5.273024000	-0.054219000	1.175201000
7	4.790899000	-0.224046000	2.440082000
8	-4.017933000	2.081608000	1.498248000
6	-4.810441000	1.323123000	2.397928000
6	-4.470771000	-0.161328000	2.224598000
8	-5.356984000	-0.927202000	1.844945000
7	-0.444993000	-1.818254000	-1.516772000
6	0.697217000	-2.601840000	-1.405449000
6	0.325426000	-3.964825000	-1.073080000
6	-1.034460000	-4.021327000	-1.073687000
6	-1.523130000	-2.691359000	-1.374711000
6	-2.870355000	-2.361882000	-1.482976000
6	-3.878598000	-3.460913000	-1.466804000
6	-4.902982000	-3.505378000	-0.500486000
6	-5.823933000	-4.555953000	-0.486913000
6	-5.748281000	-5.580096000	-1.437329000
6	-4.742129000	-5.543009000	-2.407369000
6	-3.819650000	-4.493496000	-2.421011000
7	-2.512579000	0.094157000	-1.394383000
6	-3.326865000	-1.022146000	-1.544248000
6	-4.694161000	-0.621588000	-1.598835000
6	-4.719996000	0.746453000	-1.401243000
6	-3.365221000	1.167370000	-1.267124000
8	5.602847000	1.040309000	0.727608000
1	-4.977090000	-2.712982000	0.246027000
1	1.024033000	-4.771121000	-0.868120000
1	-1.662453000	-4.883425000	-0.866047000
1	4.426644000	-1.051563000	-2.700947000
1	4.319349000	1.657830000	-2.860535000
1	0.725167000	5.256397000	-1.243103000
1	-1.897141000	5.133514000	-0.636420000
1	-5.587303000	1.400824000	-1.353163000
1	-5.541816000	-1.286150000	-1.737621000
1	2.240009000	-4.402517000	-3.160819000
1	4.087410000	-6.064722000	-3.208477000
1	6.062626000	-5.756008000	-1.697117000
1	6.147235000	-3.819344000	-0.160585000
1	1.963800000	4.830152000	-3.655847000
1	3.692124000	6.618016000	-3.696452000
1	5.570065000	6.578593000	-2.052184000
1	5.699504000	4.734879000	-0.371164000
1	3.975116000	2.955749000	-0.333886000
1	-4.099832000	4.741198000	-2.137770000
1	-5.878048000	6.201212000	-1.175567000
1	-6.819790000	5.675671000	1.079973000
1	-5.960572000	3.733625000	2.365076000
1	-6.605700000	-4.576471000	0.276936000
1	-6.470594000	-6.400015000	-1.423562000
1	-4.676967000	-6.331780000	-3.161137000
1	-3.039873000	-4.464875000	-3.185023000
1	6.218582000	-1.956895000	0.784942000
1	5.887943000	-0.943277000	-0.649547000
1	-4.657250000	1.663327000	3.437081000
1	-5.877073000	1.413144000	2.153412000
7	-3.202684000	-0.581931000	2.485750000
8	-0.119706000	1.480204000	2.470522000
8	-0.531129000	-2.236572000	2.396358000
8	2.982375000	2.128397000	2.105455000
8	2.190970000	-1.479387000	2.932426000
6	-1.784032000	-2.662722000	2.886552000
1	-1.853975000	-2.503711000	3.980778000
6	-2.916889000	-1.983323000	2.144139000
1	-1.905328000	-3.747382000	2.707867000
1	-3.837195000	-2.547145000	2.338736000
1	-2.714814000	-2.066985000	1.064476000
6	0.511210000	-3.174785000	2.544051000
1	0.614616000	-3.490600000	3.601375000
1	0.303273000	-4.077712000	1.937403000
6	1.795887000	-2.537683000	2.065824000
1	2.585046000	-3.302034000	1.989768000
1	1.649402000	-2.116912000	1.061542000
6	3.296350000	-1.725578000	3.778159000
1	3.191115000	-1.033431000	4.625746000
1	3.266927000	-2.749480000	4.193272000
6	4.634658000	-1.531654000	3.071505000
1	5.435716000	-1.681313000	3.819472000
1	4.745914000	-2.311982000	2.314062000
6	-2.133636000	0.286802000	2.967935000
6	-1.178085000	0.736905000	1.870031000
1	-2.569136000	1.172158000	3.446801000
1	-1.562559000	-0.242680000	3.741152000
1	-1.718102000	1.364052000	1.147095000
1	-0.769714000	-0.135417000	1.337286000
6	0.599446000	2.226797000	1.497714000
1	-0.073609000	2.985323000	1.064939000
6	1.818540000	2.932138000	2.053680000
1	0.919603000	1.567683000	0.673727000
1	2.041800000	3.762732000	1.363548000
1	1.590412000	3.371219000	3.042922000
6	4.702137000	0.956096000	3.300258000

1	5.000911000	0.663588000	4.319292000
1	5.427049000	1.691760000	2.927893000
6	3.332018000	1.616959000	3.373341000
1	3.405974000	2.440603000	4.112960000
1	2.565573000	0.921656000	3.744188000
8	0.621457000	-0.020463000	4.704375000
1	0.369615000	0.701524000	4.097976000
1	0.978830000	-0.659969000	4.056284000

$^3CAT_{H3O+}$:

6	3.978963000	3.760699000	-1.143584000
6	2.878700000	3.779629000	-2.026067000
6	2.794460000	4.840075000	-2.947689000
6	3.764933000	5.846305000	-2.987189000
6	4.849475000	5.813016000	-2.104967000
6	4.950250000	4.763569000	-1.182917000
6	1.837415000	2.718757000	-1.960231000
6	2.250487000	1.370403000	-2.121313000
7	1.480774000	0.249842000	-1.807329000
6	2.325795000	-0.843625000	-1.949291000
6	3.592750000	-0.420155000	-2.430452000
6	3.546320000	0.965343000	-2.542099000
26	-0.515351000	0.192861000	-1.581138000
7	-0.565078000	2.213232000	-1.555522000
6	-1.641342000	2.994470000	-1.136206000
6	-1.205510000	4.349638000	-0.919237000
6	0.125888000	4.406913000	-1.250101000
6	0.526550000	3.079882000	-1.640049000
6	-2.951737000	2.546365000	-0.965727000
6	-3.976499000	3.449196000	-0.379290000
6	-4.532371000	3.143479000	0.890878000
6	-5.520461000	3.953398000	1.461562000
6	-5.969815000	5.092204000	0.780863000
6	-5.428446000	5.422146000	-0.462569000
6	-4.444447000	4.601596000	-1.027589000
6	1.991616000	-2.188171000	-1.625981000
6	3.088409000	-3.188340000	-1.598217000
6	3.063585000	-4.309954000	-2.450679000
6	4.091713000	-5.254116000	-2.479787000
6	5.199638000	-5.090825000	-1.644992000
6	5.261712000	-3.988476000	-0.787613000
6	4.221004000	-3.051982000	-0.755578000
8	4.243059000	-1.997503000	0.124350000
6	5.481996000	-1.340661000	0.328608000
6	5.298319000	-0.102646000	1.206801000
7	4.797016000	-0.271900000	2.464668000
8	-4.017790000	2.055052000	1.531198000
6	-4.812475000	1.313650000	2.438744000
6	-4.488625000	-0.175022000	2.272415000
8	-5.396428000	-0.940499000	1.944735000
7	-0.467594000	-1.814583000	-1.557989000
6	0.670956000	-2.610188000	-1.422111000
6	0.280862000	-3.958904000	-1.107013000
6	-1.091980000	-4.005282000	-1.132197000
6	-1.559852000	-2.679038000	-1.434694000
6	-2.910993000	-2.326747000	-1.555016000
6	-3.930505000	-3.410824000	-1.541248000
6	-4.968403000	-3.439995000	-0.585374000
6	-5.902774000	-4.478610000	-0.568238000
6	-5.831124000	-5.517245000	-1.504149000
6	-4.811752000	-5.501950000	-2.461766000
6	-3.879003000	-4.461460000	-2.479426000
7	-2.516152000	0.129656000	-1.415360000
6	-3.347400000	-0.976422000	-1.594901000
6	-4.704345000	-0.553519000	-1.657619000
6	-4.711433000	0.817291000	-1.425845000
6	-3.359527000	1.218699000	-1.268589000
8	5.656470000	0.988783000	0.772529000
1	-5.037630000	-2.642081000	0.155532000
1	0.965051000	-4.776372000	-0.894262000
1	-1.728276000	-4.865766000	-0.942038000
1	4.428773000	-1.077103000	-2.661548000
1	4.341286000	1.633565000	-2.864601000
1	0.784484000	5.270548000	-1.205298000
1	-1.837789000	5.160267000	-0.564966000
1	-5.572110000	1.481744000	-1.372511000
1	-5.562032000	-1.201805000	-1.815919000
1	2.210598000	-4.419809000	-3.122945000
1	4.034505000	-6.103887000	-3.164323000
1	6.018320000	-5.814312000	-1.656112000
1	6.121439000	-3.869325000	-0.127107000
1	1.952850000	4.868212000	-3.643681000
1	3.676651000	6.656709000	-3.715895000
1	5.610118000	6.597285000	-2.135035000
1	5.789501000	4.729802000	-0.482596000
1	4.064418000	2.953588000	-0.413399000
1	-4.031862000	4.843097000	-2.009863000
1	-5.776355000	6.308328000	-0.998715000
1	-6.740181000	5.720843000	1.234260000
1	-5.925346000	3.713139000	2.446095000
1	-6.691076000	-4.480944000	0.189857000
1	-6.563069000	-6.328773000	-1.488053000
1	-4.746356000	-6.301017000	-3.205309000
1	-3.090332000	-4.453321000	-3.234928000
1	6.228856000	-2.005644000	0.797697000
1	5.886134000	-0.985177000	-0.629482000
1	-4.653300000	1.656878000	3.477007000
1	-5.880223000	1.411869000	2.200448000
7	-3.208642000	-0.593913000	2.470828000
8	-0.114853000	1.455241000	2.444298000
8	-0.538975000	-2.261964000	2.394378000
8	2.994495000	2.061654000	2.100719000
8	2.183103000	-1.526610000	2.933190000
6	-1.809870000	-2.693108000	2.831766000
1	-1.912834000	-2.568341000	3.928207000
6	-2.916812000	-1.980650000	2.079557000
1	-1.931328000	-3.770911000	2.614525000
1	-3.845154000	-2.544957000	2.225736000
1	-2.684883000	-2.022994000	1.002373000
6	0.492005000	-3.211825000	2.545827000
1	0.584740000	-3.531466000	3.603468000
1	0.277779000	-4.110407000	1.934842000
6	1.787229000	-2.591527000	2.073397000
1	2.569899000	-3.364548000	2.017745000
1	1.657600000	-2.185718000	1.060278000
6	3.282173000	-1.769777000	3.787110000
1	3.171955000	-1.074820000	4.631924000
1	3.249320000	-2.792387000	4.205595000
6	4.626768000	-1.580102000	3.091327000
1	5.420441000	-1.735488000	3.846285000
1	4.740080000	-2.358710000	2.332507000
6	-2.129587000	0.268846000	2.942386000
6	-1.176118000	0.715589000	1.841790000
1	-2.556369000	1.156363000	3.424558000
1	-1.557385000	-0.265674000	3.711798000
1	-1.713124000	1.341768000	1.115645000
1	-0.768596000	-0.157234000	1.309766000
6	0.616875000	2.189388000	1.467985000
1	-0.044706000	2.953161000	1.028098000
6	1.841452000	2.881478000	2.026045000
1	0.927336000	1.524392000	0.645366000
1	2.082891000	3.697851000	1.325369000
1	1.612904000	3.337120000	3.007994000
6	4.703929000	0.907490000	3.324981000

1	4.989362000	0.614327000	4.347743000
1	5.435947000	1.639802000	2.960160000
6	3.334487000	1.572580000	3.378999000
1	3.406071000	2.408339000	4.105585000
1	2.564619000	0.885285000	3.757820000
8	0.616327000	-0.037517000	4.683247000
1	0.368211000	0.674949000	4.062914000
1	0.970698000	-0.689694000	4.046108000

$^{3}RC1_{H3O+}$:

6	-1.484208000	6.027793000	1.553980000
6	-1.818002000	5.643679000	0.243051000
6	-2.533394000	6.553327000	-0.554122000
6	-2.910469000	7.804495000	-0.056478000
6	-2.572521000	8.172139000	1.249487000
6	-1.856423000	7.278108000	2.053573000
6	-1.425346000	4.302053000	-0.279408000
6	-0.043760000	4.043114000	-0.473164000
7	0.488405000	2.867071000	-0.963777000
6	1.853089000	3.080991000	-1.051112000
6	2.184266000	4.390683000	-0.586845000
6	0.993209000	5.000317000	-0.238835000
26	-0.487206000	1.119254000	-1.289033000
7	-2.234759000	2.056727000	-0.946645000
6	-3.473619000	1.442861000	-0.838678000
6	-4.466741000	2.405313000	-0.405152000
6	-3.820741000	3.587289000	-0.217524000
6	-2.418030000	3.361868000	-0.516152000
6	-3.751974000	0.105422000	-1.065219000
6	-5.141863000	-0.375444000	-0.803430000
6	-5.505666000	-0.899758000	0.445927000
6	-6.812901000	-1.310202000	0.716289000
6	-7.780821000	-1.227481000	-0.285557000
6	-7.443309000	-0.723513000	-1.545539000
6	-6.136270000	-0.298393000	-1.790876000
6	2.795874000	2.157813000	-1.559093000
6	4.235657000	2.566738000	-1.556949000
6	4.676903000	3.471844000	-2.540435000
6	6.007779000	3.872724000	-2.646146000
6	6.946653000	3.361864000	-1.742034000
6	6.538191000	2.477409000	-0.748362000
6	5.193395000	2.078978000	-0.641352000
8	4.950228000	1.187028000	0.360241000
6	3.666333000	1.081735000	0.974020000
6	3.804735000	0.482708000	2.367729000
7	4.389056000	-0.742716000	2.507378000
8	-4.534302000	-0.932568000	1.446708000
6	-4.351644000	-2.131859000	2.225327000
6	-3.646861000	-3.169733000	1.345793000
8	-4.252281000	-3.586658000	0.361753000
7	1.179540000	0.392005000	-2.199483000
6	2.456717000	0.924452000	-2.089490000
6	3.430118000	-0.010160000	-2.613631000
6	2.741106000	-1.104184000	-3.040393000
6	1.333878000	-0.856258000	-2.774220000
6	0.332514000	-1.799088000	-3.008980000
6	0.676597000	-3.016422000	-3.798792000
6	0.474672000	-4.319350000	-3.304688000
6	0.813429000	-5.442866000	-4.061648000
6	1.361734000	-5.294371000	-5.340048000
6	1.561950000	-4.008762000	-5.852288000
6	1.219602000	-2.887540000	-5.092399000
7	-1.479999000	-0.542275000	-1.842688000
6	-1.000075000	-1.638009000	-2.543838000
6	-2.052256000	-2.587608000	-2.752052000
6	-3.181690000	-2.079107000	-2.143000000
6	-2.819913000	-0.808958000	-1.607804000
8	3.364687000	1.117388000	3.327077000
1	0.050537000	-4.453604000	-2.311074000
1	4.504013000	0.158484000	-2.641442000
1	3.143657000	-2.015077000	-3.474551000
1	3.186201000	4.810273000	-0.553094000
1	0.844507000	6.011968000	0.128823000
1	-4.240996000	4.530048000	0.122372000
1	-5.520732000	2.185618000	-0.252886000
1	-4.166567000	-2.528581000	-2.070609000
1	-1.961367000	-3.522949000	-3.295641000
1	3.935392000	3.853268000	-3.246360000
1	6.311749000	4.570414000	-3.429511000
1	7.996603000	3.657789000	-1.805783000
1	7.246851000	2.072677000	-0.022911000
1	-2.794653000	6.270703000	-1.576602000
1	-3.466996000	8.496514000	-0.693699000
1	-2.865360000	9.149967000	1.639559000
1	-1.591853000	7.554133000	3.077553000
1	-0.930643000	5.328979000	2.185344000
1	-5.863043000	0.105655000	-2.768107000
1	-8.198074000	-0.656128000	-2.332024000
1	-8.802526000	-1.551308000	-0.075166000
1	-7.077993000	-1.689773000	1.703811000
1	0.652567000	-6.440994000	-3.646393000
1	1.627520000	-6.173071000	-5.932590000
1	1.980517000	-3.876444000	-6.853292000
1	1.368573000	-1.887418000	-5.504742000
1	3.222811000	2.075391000	1.110862000
1	2.966224000	0.482506000	0.378194000
1	-3.802433000	-1.820581000	3.116186000
1	-5.322850000	-2.535591000	2.538461000
7	-2.370808000	-3.536517000	1.635726000
8	-0.633807000	-1.556670000	4.210111000
8	0.664572000	-4.068685000	1.215885000
8	2.159136000	-1.794798000	4.035255000
8	3.379983000	-3.389884000	1.379278000
6	-0.392415000	-4.983778000	0.981239000
1	-0.503175000	-5.579973000	1.899568000
6	-1.707317000	-4.336715000	0.590634000
1	-0.114044000	-5.683193000	0.172439000
1	-2.400136000	-5.135196000	0.283814000
1	-1.572069000	-3.704227000	-0.302506000
6	1.389798000	-3.663141000	0.066096000
1	1.785343000	-4.557116000	-0.452067000
1	0.738351000	-3.115335000	-0.636202000
6	2.520302000	-2.741778000	0.456102000
1	3.052339000	-2.477222000	-0.476535000
1	2.114726000	-1.814657000	0.884969000
6	4.726745000	-2.984682000	1.359665000
1	5.205547000	-3.486630000	2.214050000
1	5.235299000	-3.354092000	0.444951000
6	4.990165000	-1.478046000	1.397471000
1	6.088837000	-1.351625000	1.413280000
1	4.651049000	-1.031348000	0.459850000
6	-1.717979000	-3.193945000	2.900237000
6	-1.031684000	-1.836692000	2.882914000
1	-2.456657000	-3.218750000	3.713676000
1	-0.969720000	-3.951615000	3.154604000
1	-1.720290000	-1.060777000	2.507256000
1	-0.173934000	-1.852729000	2.196092000
6	0.235248000	-0.442758000	4.360266000
1	-0.226808000	0.285389000	5.052565000
6	1.571011000	-0.868986000	4.933473000
1	0.381553000	0.063638000	3.393870000
1	2.206913000	0.023940000	5.044174000
1	1.435722000	-1.344627000	5.925267000
6	4.564327000	-1.243600000	3.865755000

1	5.539197000	-1.752414000	3.940351000
1	4.591271000	-0.371939000	4.531336000
6	3.475084000	-2.223324000	4.321656000
1	3.602382000	-2.406325000	5.406117000
1	3.574616000	-3.183634000	3.803614000
8	1.249096000	-4.436494000	3.873349000
1	1.321526000	-3.483601000	4.076514000
1	1.289498000	-4.398032000	2.897839000
6	-0.151441000	0.688699000	0.577246000
8	0.826834000	-0.051520000	0.698680000
8	-0.864713000	1.174784000	1.500735000
8	-3.187402000	1.129074000	2.121277000
1	-3.716958000	0.309134000	1.803147000
1	-2.275019000	1.167361000	1.765620000
1	-3.156438000	1.146649000	3.092957000

^{3}IM1 $_{H3O+}$:

6	2.184849000	5.755515000	-1.690813000
6	2.424049000	5.418245000	-0.348398000
6	3.173118000	6.301975000	0.445098000
6	3.667511000	7.495460000	-0.088472000
6	3.420490000	7.822822000	-1.425600000
6	2.677260000	6.948854000	-2.225683000
6	1.906757000	4.134169000	0.212737000
6	0.534327000	3.988472000	0.399911000
7	-0.123289000	2.879954000	0.913711000
6	-1.459467000	3.237983000	0.994941000
6	-1.646983000	4.583027000	0.482776000
6	-0.420489000	5.049227000	0.129051000
26	0.634906000	1.082162000	1.261202000
7	2.522625000	1.846486000	0.902051000
6	3.681782000	1.116070000	0.845163000
6	4.778611000	1.953252000	0.441944000
6	4.253226000	3.206349000	0.215125000
6	2.841929000	3.114766000	0.481916000
6	3.786440000	-0.264355000	1.099832000
6	5.102650000	-0.911789000	0.812888000
6	5.358776000	-1.436127000	-0.466739000
6	6.591785000	-2.023436000	-0.767147000
6	7.578636000	-2.105669000	0.216398000
6	7.342077000	-1.593974000	1.496279000
6	6.112055000	-0.996564000	1.781460000
6	-2.496673000	2.468601000	1.513484000
6	-3.879021000	3.047483000	1.526609000
6	-4.182282000	4.005209000	2.512940000
6	-5.451668000	4.563556000	2.648709000
6	-6.469087000	4.158754000	1.776919000
6	-6.196931000	3.221583000	0.785449000
6	-4.913814000	2.661773000	0.645839000
8	-4.813002000	1.740866000	-0.351847000
6	-3.567118000	1.457264000	-0.986632000
6	-3.826531000	0.774830000	-2.324210000
7	-4.501117000	-0.411836000	-2.331540000
8	4.377815000	-1.278720000	-1.423143000
6	4.010344000	-2.374884000	-2.262132000
6	3.239445000	-3.391542000	-1.413183000
8	3.790396000	-3.833824000	-0.407153000
7	-1.112365000	0.538229000	2.203228000
6	-2.312712000	1.200003000	2.092182000
6	-3.370769000	0.408689000	2.657848000
6	-2.791582000	-0.747417000	3.121555000
6	-1.384473000	-0.654271000	2.825914000
6	-0.446314000	-1.681762000	3.077886000
6	-0.889888000	-2.822614000	3.934463000
6	-0.876127000	-4.156513000	3.490302000
6	-1.305526000	-5.196547000	4.318761000
6	-1.757941000	-4.925878000	5.613387000
6	-1.774045000	-3.604828000	6.072503000
6	-1.345131000	-2.567400000	5.242013000
7	1.448486000	-0.625710000	1.859513000
6	0.860164000	-1.653143000	2.586791000
6	1.821783000	-2.726029000	2.788892000
6	2.967354000	-2.364546000	2.156695000
6	2.752989000	-1.034732000	1.620165000
8	-3.420109000	1.310975000	-3.354972000
1	-0.531432000	-4.384617000	2.483037000
1	-4.418673000	0.695770000	2.696597000
1	-3.280901000	-1.591197000	3.600067000
1	-2.601294000	5.099898000	0.431052000
1	-0.166104000	6.028198000	-0.267533000
1	4.776328000	4.098995000	-0.117450000
1	5.810223000	1.628898000	0.329832000
1	3.895317000	-2.919303000	2.069689000
1	1.634137000	-3.636995000	3.348657000
1	-3.381586000	4.301436000	3.194623000
1	-5.647114000	5.301628000	3.429393000
1	-7.473877000	4.579124000	1.863516000
1	-6.968469000	2.897967000	0.083998000
1	3.364781000	6.048594000	1.490263000
1	4.245504000	8.173834000	0.543883000
1	3.806746000	8.755732000	-1.843075000
1	2.483396000	7.194867000	-3.272594000
1	1.610355000	5.068554000	-2.316485000
1	5.915551000	-0.589263000	2.775857000
1	8.112995000	-1.656852000	2.267219000
1	8.539166000	-2.567930000	-0.022931000
1	6.781265000	-2.417193000	-1.766737000
1	-1.290156000	-6.223537000	3.945932000
1	-2.094843000	-5.738827000	6.260886000
1	-2.118715000	-3.380117000	7.084862000
1	-1.355363000	-1.538869000	5.608371000
1	-3.031178000	2.387064000	-1.214172000
1	-2.913625000	0.830385000	-0.368936000
1	3.434357000	-1.931635000	-3.078339000
1	4.894809000	-2.870010000	-2.691027000
7	1.968114000	-3.735012000	-1.748535000
8	0.239905000	-1.814836000	-4.390795000
8	-1.058568000	-4.026899000	-1.190914000
8	-2.534187000	-1.766232000	-4.003564000
8	-3.687175000	-3.097683000	-1.125826000
6	-0.096397000	-5.063079000	-1.091453000
1	-0.113146000	-5.596441000	-2.052869000
6	1.300676000	-4.587675000	-0.747809000
1	-0.397425000	-5.787084000	-0.313039000
1	1.925174000	-5.479265000	-0.572859000
1	1.292016000	-4.044031000	0.210445000
6	-1.660773000	-3.635411000	0.030617000
1	-2.101521000	-4.522873000	0.523367000
1	-0.911102000	-3.197495000	0.712554000
6	-2.723181000	-2.590757000	-0.216898000
1	-3.181366000	-2.355764000	0.762492000
1	-2.258684000	-1.668590000	-0.598757000
6	-4.973386000	-2.538671000	-1.028023000
1	-5.572423000	-3.016527000	-1.817683000
1	-5.444982000	-2.807814000	-0.060269000
6	-5.063491000	-1.014991000	-1.124605000
1	-6.134685000	-0.755342000	-1.034890000
1	-4.571004000	-0.574787000	-0.252824000
6	1.363028000	-3.395127000	-3.039631000
6	0.694460000	-2.027562000	-3.065174000
1	2.126086000	-3.448271000	-3.830091000
1	0.604542000	-4.141626000	-3.299345000
1	1.401199000	-1.235178000	-2.761785000
1	-0.136911000	-2.004477000	-2.347200000

6	-0.529906000	-0.634153000	-4.574926000
1	-0.058370000	-0.015346000	-5.361159000
6	-1.946545000	-0.958417000	-5.008032000
1	-0.549784000	-0.042320000	-3.647892000
1	-2.501750000	-0.013298000	-5.125802000
1	-1.948823000	-1.502209000	-5.973982000
6	-4.847183000	-0.969423000	-3.635116000
1	-5.868697000	-1.381458000	-3.592105000
1	-4.855146000	-0.136272000	-4.348674000
6	-3.905916000	-2.075579000	-4.129495000
1	-4.163971000	-2.305539000	-5.181503000
1	-4.040957000	-2.990967000	-3.542309000
8	-1.817017000	-4.454531000	-3.788951000
1	-1.812883000	-3.514707000	-4.054128000
1	-1.811207000	-4.346468000	-2.818054000
6	0.094719000	0.550603000	-0.724711000
8	-0.993734000	0.023539000	-0.955660000
8	0.734657000	1.014860000	-1.841007000
8	3.400017000	1.258615000	-2.231453000
1	3.751441000	0.398776000	-1.925743000
1	1.680801000	1.234667000	-1.680716000
1	3.885608000	1.898039000	-1.684954000

^{3}RC2 $_{H3O+}$:

6	2.018457000	5.795741000	-1.627566000
6	2.250974000	5.462780000	-0.283286000
6	2.974195000	6.358063000	0.520552000
6	3.450035000	7.562059000	-0.006275000
6	3.210078000	7.886287000	-1.345332000
6	2.492812000	6.999550000	-2.154981000
6	1.754506000	4.167453000	0.271031000
6	0.388940000	3.999680000	0.474174000
7	-0.256120000	2.877077000	0.982502000
6	-1.600028000	3.208993000	1.039091000
6	-1.804257000	4.553172000	0.530306000
6	-0.582666000	5.044220000	0.198882000
26	0.518943000	1.094062000	1.344472000
7	2.406159000	1.874315000	0.918690000
6	3.575685000	1.160840000	0.885908000
6	4.662936000	2.016279000	0.489415000
6	4.113250000	3.268232000	0.253749000
6	2.709993000	3.157228000	0.515226000
6	3.697366000	-0.218987000	1.155053000
6	5.040245000	-0.830919000	0.913367000
6	5.412557000	-1.273894000	-0.363828000
6	6.677244000	-1.796793000	-0.626449000
6	7.598395000	-1.909543000	0.415643000
6	7.252816000	-1.487558000	1.703366000
6	5.988114000	-0.946194000	1.941314000
6	-2.634508000	2.410457000	1.517703000
6	-4.031568000	2.945564000	1.466172000
6	-4.414339000	3.910475000	2.416416000
6	-5.707445000	4.426526000	2.474286000
6	-6.663799000	3.971380000	1.559339000
6	-6.310654000	3.024865000	0.602450000
6	-5.004847000	2.506765000	0.542242000
8	-4.819206000	1.562542000	-0.421606000
6	-3.547331000	1.327076000	-1.024141000
6	-3.756287000	0.670342000	-2.385276000
7	-4.411742000	-0.525888000	-2.437972000
8	4.485858000	-1.118920000	-1.404883000
6	4.130401000	-2.242954000	-2.240632000
6	3.367789000	-3.255376000	-1.382927000
8	3.912075000	-3.656976000	-0.357023000
7	-1.227189000	0.505692000	2.226945000
6	-2.439274000	1.141514000	2.090979000
6	-3.491791000	0.322387000	2.626480000
6	-2.897043000	-0.821641000	3.099764000
6	-1.486306000	-0.695666000	2.838019000
6	-0.529978000	-1.701539000	3.105936000
6	-0.967105000	-2.856046000	3.947523000
6	-0.937906000	-4.183753000	3.487344000
6	-1.363626000	-5.236313000	4.301717000
6	-1.826797000	-4.983119000	5.596037000
6	-1.859182000	-3.667493000	6.069500000
6	-1.434791000	-2.617000000	5.253241000
7	1.362263000	-0.601907000	1.921740000
6	0.782443000	-1.643316000	2.637079000
6	1.756786000	-2.704555000	2.837643000
6	2.900067000	-2.327266000	2.212686000
6	2.673174000	-0.995760000	1.681465000
8	-3.333250000	1.239068000	-3.390767000
1	-0.585756000	-4.396844000	2.479359000
1	-4.546407000	0.585449000	2.641585000
1	-3.377154000	-1.678237000	3.564548000
1	-2.767313000	5.051105000	0.462761000
1	-0.340838000	6.028661000	-0.191559000
1	4.628574000	4.169932000	-0.066041000
1	5.711755000	1.729921000	0.444989000
1	3.832374000	-2.874039000	2.122415000
1	1.575704000	-3.622395000	3.388257000
1	-3.660643000	4.246968000	3.132055000
1	-5.968308000	5.171678000	3.228593000
1	-7.684763000	4.359469000	1.585533000
1	-7.033882000	2.661578000	-0.130240000
1	3.159674000	6.106667000	1.567308000
1	4.007846000	8.250690000	0.632902000
1	3.581838000	8.827466000	-1.757227000
1	2.304680000	7.244023000	-3.203198000
1	1.463300000	5.098866000	-2.259673000
1	5.715053000	-0.604036000	2.941665000
1	7.971556000	-1.573259000	2.520884000
1	8.589461000	-2.322887000	0.217328000
1	6.939219000	-2.117900000	-1.635500000
1	-1.336812000	-6.259042000	3.918207000
1	-2.160427000	-5.806034000	6.232480000
1	-2.213726000	-3.457200000	7.081448000
1	-1.458462000	-1.592222000	5.629504000
1	-3.027324000	2.273460000	-1.215516000
1	-2.899863000	0.700253000	-0.398983000
1	3.563112000	-1.810005000	-3.067234000
1	5.036641000	-2.717493000	-2.642368000
7	2.124669000	-3.651291000	-1.750705000
8	0.396307000	-1.727509000	-4.387389000
8	-0.894057000	-4.031800000	-1.244285000
8	-2.394855000	-1.819662000	-4.106047000
8	-3.534621000	-3.183206000	-1.250133000
6	0.092255000	-5.043097000	-1.133021000
1	0.107203000	-5.571981000	-2.096961000
6	1.467894000	-4.528299000	-0.762876000
1	-0.201292000	-5.778451000	-0.362687000
1	2.118195000	-5.399717000	-0.581035000
1	1.425532000	-3.987452000	0.195462000
6	-1.533621000	-3.666097000	-0.034346000
1	-1.961748000	-4.568558000	0.441637000
1	-0.812612000	-3.212416000	0.668344000
6	-2.620375000	-2.651994000	-0.305461000
1	-3.117231000	-2.439640000	0.660203000
1	-2.172612000	-1.713429000	-0.668281000
6	-4.847925000	-2.685997000	-1.183152000
1	-5.399738000	-3.178163000	-1.997908000
1	-5.335855000	-2.990931000	-0.234447000
6	-4.999761000	-1.166824000	-1.263486000
1	-6.083576000	-0.951858000	-1.218662000

1	-4.565890000	-0.718890000	-0.365447000
6	1.527788000	-3.314251000	-3.047112000
6	0.827398000	-1.962372000	-3.059442000
1	2.300415000	-3.343403000	-3.829462000
1	0.789141000	-4.074987000	-3.321768000
1	1.507768000	-1.159530000	-2.719913000
1	-0.018020000	-1.978824000	-2.359277000
6	-0.426848000	-0.579771000	-4.550254000
1	0.045436000	0.105593000	-5.278477000
6	-1.802751000	-0.956749000	-5.061651000
1	-0.534722000	-0.046543000	-3.593817000
1	-2.395359000	-0.034948000	-5.177321000
1	-1.735283000	-1.470901000	-6.041108000
6	-4.722768000	-1.055794000	-3.762090000
1	-5.740105000	-1.480163000	-3.749330000
1	-4.726552000	-0.205877000	-4.455747000
6	-3.760190000	-2.139740000	-4.265135000
1	-3.997409000	-2.346794000	-5.326735000
1	-3.895105000	-3.071114000	-3.703259000
8	-1.604667000	-4.471593000	-3.856976000
1	-1.644587000	-3.532090000	-4.122135000
1	-1.622012000	-4.365307000	-2.886255000
6	0.111308000	0.580523000	-0.667225000
8	-0.855318000	-0.023342000	-1.110161000
8	0.941862000	1.131869000	-1.626416000
8	4.066593000	1.187134000	-2.168871000
1	4.254887000	0.170893000	-1.889304000
1	1.649745000	1.579590000	-1.137352000
1	4.288138000	1.752896000	-1.361897000
1	4.640746000	1.455261000	-2.910484000

^{3}IM2 $_{H3O+}$:

6	5.159833000	3.385879000	-1.575424000
6	5.347568000	2.856752000	-0.287560000
6	6.576596000	3.043535000	0.359921000
6	7.602382000	3.757708000	-0.267341000
6	7.408865000	4.286237000	-1.547385000
6	6.186016000	4.097428000	-2.201021000
6	4.237372000	2.085350000	0.348460000
6	3.121227000	2.790593000	0.810298000
7	1.947639000	2.226719000	1.271472000
6	1.059011000	3.262765000	1.425144000
6	1.690950000	4.517451000	1.079086000
6	2.974990000	4.229368000	0.724039000
26	1.547972000	0.281854000	1.560313000
7	3.321381000	-0.180175000	0.740328000
6	3.703977000	-1.437483000	0.341161000
6	4.965878000	-1.373542000	-0.352669000
6	5.346586000	-0.056509000	-0.359209000
6	4.296670000	0.685748000	0.296378000
6	3.012171000	-2.627770000	0.604512000
6	3.415489000	-3.856412000	-0.139322000
6	2.894370000	-4.046810000	-1.434970000
6	3.199949000	-5.198193000	-2.163919000
6	4.029268000	-6.172912000	-1.601654000
6	4.560886000	-5.996836000	-0.321774000
6	4.252443000	-4.839448000	0.399680000
6	-0.262981000	3.152368000	1.878551000
6	-1.164727000	4.321930000	1.680070000
6	-1.739403000	5.016353000	2.756454000
6	-2.596575000	6.098933000	2.551058000
6	-2.891773000	6.510927000	1.247899000
6	-2.327015000	5.840491000	0.162069000
6	-1.469228000	4.754982000	0.368673000
8	-0.885809000	4.193842000	-0.736918000
6	-1.115405000	2.820298000	-1.070609000
6	-2.282520000	2.730457000	-2.055747000
7	-3.560223000	2.634330000	-1.582828000
8	2.135545000	-3.022658000	-1.960129000
6	0.962121000	-3.295950000	-2.704130000
6	-0.143376000	-3.806150000	-1.771298000
8	0.173247000	-4.295662000	-0.693798000
7	-0.114176000	0.775968000	2.555957000
6	-0.773245000	1.987712000	2.473413000
6	-2.049728000	1.892189000	3.138339000
6	-2.131919000	0.631379000	3.663674000
6	-0.932543000	-0.068296000	3.274222000
6	-0.721046000	-1.440761000	3.493331000
6	-1.710467000	-2.169142000	4.341440000
6	-2.559919000	-3.142742000	3.789139000
6	-3.500448000	-3.801877000	4.584159000
6	-3.605752000	-3.498364000	5.945323000
6	-2.765992000	-2.530546000	6.505393000
6	-1.827089000	-1.869909000	5.708956000
7	1.261874000	-1.630720000	2.030506000
6	0.309057000	-2.158979000	2.875901000
6	0.449666000	-3.602418000	2.931164000
6	1.423113000	-3.940063000	2.041505000
6	1.942127000	-2.704718000	1.497465000
8	-2.035110000	2.819328000	-3.257366000
1	-2.487302000	-3.375566000	2.725728000
1	-2.789406000	2.683973000	3.206280000
1	-2.951790000	0.201487000	4.231822000
1	1.209381000	5.490440000	1.129728000
1	3.757134000	4.917285000	0.415012000
1	6.249116000	0.377962000	-0.780771000
1	5.496860000	-2.229462000	-0.761913000
1	1.781770000	-4.930484000	1.778650000
1	-0.145816000	-4.265656000	3.551583000
1	-1.489920000	4.704427000	3.772455000
1	-3.025888000	6.623556000	3.407018000
1	-3.561621000	7.356219000	1.074836000
1	-2.540349000	6.140580000	-0.865414000
1	6.725992000	2.629125000	1.359564000
1	8.555720000	3.902027000	0.246578000
1	8.211343000	4.843429000	-2.036575000
1	6.032145000	4.503247000	-3.203819000
1	4.202443000	3.217721000	-2.075079000
1	4.656017000	-4.692105000	1.403814000
1	5.212356000	-6.756495000	0.114850000
1	4.264621000	-7.071805000	-2.176002000
1	2.797477000	-5.337982000	-3.167974000
1	-4.156781000	-4.551713000	4.136324000
1	-4.341413000	-4.013470000	6.567400000
1	-2.840407000	-2.288920000	7.568304000
1	-1.172122000	-1.114109000	6.147931000
1	-0.220576000	2.484011000	-1.606759000
1	-1.234735000	2.201574000	-0.173776000
1	0.694621000	-2.351437000	-3.190046000
1	1.140145000	-4.036580000	-3.501375000
7	-1.434015000	-3.725067000	-2.201057000
8	-2.372933000	-1.330246000	-4.872619000
8	-3.691821000	-2.230105000	-1.000000000
8	-4.119763000	0.561893000	-3.578149000
8	-4.570803000	0.199340000	-0.152502000
6	-3.780158000	-3.614637000	-1.264995000
1	-4.321966000	-3.673837000	-2.218912000
6	-2.443831000	-4.338872000	-1.333010000
1	-4.398094000	-4.124733000	-0.501835000
1	-2.650448000	-5.373864000	-1.664371000
1	-1.989218000	-4.413109000	-0.338645000
6	-3.348031000	-1.802889000	0.299358000
1	-4.088033000	-2.172809000	1.034405000
1	-2.349843000	-2.171587000	0.599387000

6	-3.321010000	-0.288480000	0.293213000
1	-3.089987000	0.062487000	1.313303000
1	-2.514918000	0.055328000	-0.378869000
6	-4.906873000	1.509115000	0.240416000
1	-5.898633000	1.704278000	-0.193054000
1	-5.013955000	1.576817000	1.341372000
6	-3.918411000	2.605457000	-0.167570000
1	-4.364011000	3.571586000	0.129478000
1	-3.007605000	2.500469000	0.427201000
6	-1.804683000	-3.212695000	-3.521919000
6	-2.004384000	-1.696154000	-3.555112000
1	-1.050463000	-3.499963000	-4.269423000
1	-2.736527000	-3.704746000	-3.833986000
1	-1.065478000	-1.186635000	-3.271802000
1	-2.766469000	-1.401453000	-2.824636000
6	-2.350902000	0.064447000	-5.121713000
1	-2.058639000	0.207279000	-6.175888000
6	-3.685921000	0.758295000	-4.914286000
1	-1.594635000	0.559393000	-4.487826000
1	-3.543687000	1.830615000	-5.127690000
1	-4.446135000	0.356598000	-5.612278000
6	-4.635068000	2.754236000	-2.566190000
1	-5.481423000	3.285073000	-2.102008000
1	-4.259832000	3.377522000	-3.387702000
6	-5.146617000	1.418058000	-3.107490000
1	-5.885111000	1.620643000	-3.905585000
1	-5.653727000	0.849439000	-2.320114000
8	-5.644349000	-1.649618000	-2.860108000
1	-5.066952000	-1.032204000	-3.346650000
1	-5.183306000	-1.662773000	-2.000673000
6	0.503859000	-0.077176000	-0.329354000
8	-0.012922000	-0.849375000	-0.984052000
8	1.875067000	1.941209000	-2.084527000
8	3.027331000	-0.461725000	-2.661905000
1	2.734046000	-1.341555000	-2.350203000
1	2.029069000	2.031080000	-1.133666000
1	2.244527000	1.052104000	-2.295734000
1	3.923969000	-0.386349000	-2.299345000

^{4}CAT$_{H3O+}$:

6	3.931600000	3.743626000	-1.091027000
6	2.861290000	3.764628000	-2.006809000
6	2.805956000	4.807783000	-2.946751000
6	3.784841000	5.806314000	-2.970687000
6	4.840216000	5.777143000	-2.054286000
6	4.908620000	4.741282000	-1.114844000
6	1.813021000	2.706036000	-1.954275000
6	2.232754000	1.360325000	-2.126054000
7	1.471305000	0.238810000	-1.818471000
6	2.312665000	-0.846715000	-1.969525000
6	3.579705000	-0.419475000	-2.455050000
6	3.530029000	0.963631000	-2.550867000
26	-0.521092000	0.180819000	-1.573555000
7	-0.578131000	2.199731000	-1.556982000
6	-1.652289000	2.978342000	-1.159042000
6	-1.222137000	4.354302000	-0.964900000
6	0.097726000	4.408631000	-1.286709000
6	0.507559000	3.063514000	-1.652003000
6	-2.953648000	2.533837000	-0.977263000
6	-3.987709000	3.443571000	-0.414503000
6	-4.533418000	3.153008000	0.860984000
6	-5.531581000	3.960317000	1.416927000
6	-5.997341000	5.076294000	0.711469000
6	-5.465282000	5.387878000	-0.540951000
6	-4.470424000	4.571455000	-1.091473000
6	1.984680000	-2.191862000	-1.635554000
6	3.089725000	-3.187149000	-1.613662000
6	3.068276000	-4.303220000	-2.469793000
6	4.104502000	-5.238664000	-2.499761000
6	5.209253000	-5.065981000	-1.663569000
6	5.266030000	-3.965156000	-0.804556000
6	4.218519000	-3.036504000	-0.771439000
8	4.229668000	-1.975749000	0.098523000
6	5.466963000	-1.312816000	0.308718000
6	5.272020000	-0.070841000	1.179594000
7	4.788925000	-0.239433000	2.444373000
8	-4.002931000	2.081412000	1.515998000
6	-4.804174000	1.316611000	2.401497000
6	-4.469438000	-0.168141000	2.221925000
8	-5.363331000	-0.932749000	1.858366000
7	-0.459070000	-1.824183000	-1.520952000
6	0.676268000	-2.610185000	-1.407468000
6	0.294717000	-3.977034000	-1.087594000
6	-1.064315000	-4.026721000	-1.098818000
6	-1.542813000	-2.687701000	-1.392037000
6	-2.886550000	-2.343658000	-1.493977000
6	-3.904318000	-3.433586000	-1.487514000
6	-4.931001000	-3.477719000	-0.523193000
6	-5.859277000	-4.521786000	-0.517474000
6	-5.789718000	-5.540812000	-1.474141000
6	-4.781938000	-5.504306000	-2.442396000
6	-3.852505000	-4.460698000	-2.448402000
7	-2.513440000	0.114703000	-1.380903000
6	-3.335097000	-0.997029000	-1.540754000
6	-4.695888000	-0.585586000	-1.593752000
6	-4.711619000	0.785389000	-1.387973000
6	-3.357693000	1.196256000	-1.249806000
8	5.607042000	1.022625000	0.733119000
1	-5.000300000	-2.690099000	0.228765000
1	0.987470000	-4.788529000	-0.882862000
1	-1.698808000	-4.886568000	-0.901747000
1	4.415175000	-1.073176000	-2.693298000
1	4.321365000	1.637134000	-2.869540000
1	0.754803000	5.273687000	-1.258698000
1	-1.862147000	5.166049000	-0.629484000
1	-5.575178000	1.444766000	-1.338121000
1	-5.549369000	-1.241915000	-1.736166000
1	2.216164000	-4.417873000	-3.142457000
1	4.054491000	-6.088741000	-3.183852000
1	6.032940000	-5.783586000	-1.675137000
1	6.126390000	-3.840417000	-0.146435000
1	1.985867000	4.829711000	-3.668012000
1	3.724684000	6.607168000	-3.712106000
1	5.606252000	6.556312000	-2.072054000
1	5.727386000	4.711550000	-0.391089000
1	3.992018000	2.943460000	-0.350410000
1	-4.064236000	4.796464000	-2.080207000
1	-5.827906000	6.257031000	-1.094263000
1	-6.775343000	5.703940000	1.152677000
1	-5.931816000	3.734099000	2.406537000
1	-6.641994000	-4.541788000	0.245507000
1	-6.517809000	-6.355719000	-1.466487000
1	-4.721049000	-6.288689000	-3.201174000
1	-3.071811000	-4.432583000	-3.211500000
1	6.211482000	-1.974752000	0.784007000
1	5.874971000	-0.960630000	-0.648773000
1	-4.659101000	1.645814000	3.445719000
1	-5.868697000	1.412142000	2.149742000
7	-3.198213000	-0.590474000	2.465101000
8	-0.103717000	1.451956000	2.439367000
8	-0.531639000	-2.254919000	2.388108000
8	2.984922000	2.113046000	2.103273000
8	2.188642000	-1.504345000	2.936860000
6	-1.789868000	-2.676333000	2.869638000

1	-1.866183000	-2.517706000	3.963529000
6	-2.915453000	-1.991603000	2.120814000
1	-1.913825000	-3.760427000	2.689333000
1	-3.839099000	-2.551965000	2.308696000
1	-2.707342000	-2.073572000	1.042137000
6	0.506045000	-3.196467000	2.547892000
1	0.600008000	-3.507155000	3.607622000
1	0.298469000	-4.101686000	1.944400000
6	1.797743000	-2.569086000	2.075912000
1	2.583800000	-3.338274000	2.017090000
1	1.664933000	-2.159141000	1.065138000
6	3.294946000	-1.741296000	3.784020000
1	3.186768000	-1.045011000	4.627855000
1	3.269252000	-2.763106000	4.204593000
6	4.632976000	-1.546436000	3.077284000
1	5.433855000	-1.694233000	3.825855000
1	4.745397000	-2.327647000	2.320942000
6	-2.124105000	0.275870000	2.938189000
6	-1.165170000	0.715541000	1.838053000
1	-2.553154000	1.167510000	3.410519000
1	-1.553790000	-0.250899000	3.713866000
1	-1.700530000	1.343800000	1.112464000
1	-0.763135000	-0.167729000	1.315584000
6	0.608230000	2.220573000	1.479035000
1	-0.068597000	2.983330000	1.059854000
6	1.824034000	2.920644000	2.048066000
1	0.935118000	1.579655000	0.643436000
1	2.052750000	3.755353000	1.364593000
1	1.589486000	3.353409000	3.038490000
6	4.701256000	0.941443000	3.303504000
1	4.997602000	0.649453000	4.323379000
1	5.427967000	1.675643000	2.931753000
6	3.331820000	1.603986000	3.372942000
1	3.405069000	2.428887000	4.111165000
1	2.564158000	0.909796000	3.743497000
8	0.617813000	-0.033003000	4.695222000
1	0.370244000	0.688505000	4.087072000
1	0.976587000	-0.674615000	4.049753000

$^{5}CAT_{H3O+}$:

6	3.975947000	3.808493000	-1.173903000
6	2.856082000	3.810687000	-2.033342000
6	2.742182000	4.872171000	-2.952853000
6	3.701417000	5.886832000	-3.015965000
6	4.807214000	5.866249000	-2.158802000
6	4.937494000	4.819808000	-1.237510000
6	1.827341000	2.741385000	-1.946298000
6	2.248341000	1.400291000	-2.081697000
7	1.484627000	0.272785000	-1.781466000
6	2.336045000	-0.820386000	-1.911864000
6	3.611407000	-0.382566000	-2.376571000
6	3.556610000	0.996437000	-2.488171000
26	-0.518031000	0.205674000	-1.573366000
7	-0.578121000	2.240278000	-1.537518000
6	-1.655266000	3.012082000	-1.110955000
6	-1.233416000	4.367142000	-0.899008000
6	0.102706000	4.430399000	-1.237411000
6	0.504799000	3.108122000	-1.626892000
6	-2.967612000	2.542464000	-0.939042000
6	-3.996096000	3.437757000	-0.346613000
6	-4.542574000	3.125779000	0.925914000
6	-5.533870000	3.927321000	1.503431000
6	-5.994723000	5.064211000	0.827844000
6	-5.461251000	5.401148000	-0.417519000
6	-4.473887000	4.589524000	-0.988932000
6	2.009609000	-2.161361000	-1.612686000
6	3.110211000	-3.158250000	-1.604181000
6	3.086813000	-4.266198000	-2.474835000
6	4.117717000	-5.206026000	-2.523685000
6	5.227305000	-5.055203000	-1.687911000
6	5.287465000	-3.968487000	-0.811085000
6	4.244015000	-3.035359000	-0.760605000
8	4.267124000	-1.995471000	0.137033000
6	5.507368000	-1.346967000	0.356884000
6	5.317737000	-0.109439000	1.234283000
7	4.800484000	-0.279369000	2.485629000
8	-4.018381000	2.039611000	1.563881000
6	-4.815918000	1.274389000	2.448762000
6	-4.487690000	-0.211621000	2.263389000
8	-5.397633000	-0.978086000	1.944717000
7	-0.457163000	-1.818052000	-1.558595000
6	0.683434000	-2.606066000	-1.417979000
6	0.304199000	-3.957110000	-1.127911000
6	-1.075942000	-4.008003000	-1.162422000
6	-1.542642000	-2.683310000	-1.450892000
6	-2.905533000	-2.321735000	-1.566447000
6	-3.918844000	-3.408873000	-1.573003000
6	-4.967559000	-3.456588000	-0.627664000
6	-5.899705000	-4.497626000	-0.633751000
6	-5.815062000	-5.525955000	-1.579584000
6	-4.782237000	-5.496639000	-2.523822000
6	-3.853247000	-4.453764000	-2.519184000
7	-2.518851000	0.133417000	-1.405051000
6	-3.342482000	-0.978217000	-1.573797000
6	-4.707713000	-0.555681000	-1.614295000
6	-4.720790000	0.806445000	-1.368336000
6	-3.364483000	1.221333000	-1.234242000
8	5.682933000	0.981847000	0.805624000
1	-5.045294000	-2.671297000	0.125954000
1	0.991127000	-4.775307000	-0.927453000
1	-1.709963000	-4.873681000	-0.988356000
1	4.453483000	-1.032995000	-2.603028000
1	4.349761000	1.667386000	-2.809155000
1	0.757457000	5.297152000	-1.194526000
1	-1.870493000	5.175459000	-0.548248000
1	-5.585154000	1.464306000	-1.298810000
1	-5.564324000	-1.205081000	-1.772634000
1	2.231174000	-4.368266000	-3.144863000
1	4.061759000	-6.043104000	-3.223899000
1	6.048023000	-5.776024000	-1.713772000
1	6.148075000	-3.858476000	-0.150088000
1	1.885067000	4.892046000	-3.629890000
1	3.588952000	6.694599000	-3.744439000
1	5.559343000	6.657755000	-2.207425000
1	5.791680000	4.795843000	-0.554920000
1	4.083517000	3.006746000	-0.440603000
1	-4.066565000	4.837140000	-1.971853000
1	-5.818322000	6.286080000	-0.949759000
1	-6.767675000	5.686133000	1.286114000
1	-5.932450000	3.680798000	2.489089000
1	-6.695921000	-4.510743000	0.116156000
1	-6.544491000	-6.339908000	-1.581205000
1	-4.704959000	-6.287315000	-3.275398000
1	-3.055167000	-4.436788000	-3.264574000
1	6.244900000	-2.017187000	0.833581000
1	5.925920000	-0.991973000	-0.595269000
1	-4.666572000	1.596986000	3.495292000
1	-5.882265000	1.373011000	2.204696000
7	-3.204332000	-0.627720000	2.445735000
8	-0.109275000	1.416356000	2.409391000
8	-0.533248000	-2.286870000	2.387930000
8	2.985942000	2.034041000	2.098064000
8	2.184124000	-1.542233000	2.937028000
6	-1.807596000	-2.725864000	2.808063000

1	-1.922963000	-2.608567000	3.904178000
6	-2.909431000	-2.012132000	2.049110000
1	-1.922183000	-3.802649000	2.582203000
1	-3.838593000	-2.577087000	2.186918000
1	-2.670955000	-2.049745000	0.973267000
6	0.499827000	-3.233923000	2.543700000
1	0.587654000	-3.554265000	3.601616000
1	0.291611000	-4.131957000	1.930010000
6	1.796014000	-2.610730000	2.077725000
1	2.581061000	-3.381960000	2.029782000
1	1.672139000	-2.209150000	1.062151000
6	3.278741000	-1.780336000	3.798006000
1	3.161412000	-1.084501000	4.641157000
1	3.246568000	-2.802402000	4.217897000
6	4.627001000	-1.588138000	3.110346000
1	5.416259000	-1.743758000	3.869957000
1	4.745226000	-2.365987000	2.351421000
6	-2.123517000	0.236244000	2.908321000
6	-1.171032000	0.681264000	1.804907000
1	-2.546939000	1.126828000	3.387163000
1	-1.547816000	-0.294935000	3.677284000
1	-1.708272000	1.309795000	1.080848000
1	-0.766015000	-0.197626000	1.277289000
6	0.614919000	2.172214000	1.444312000
1	-0.050165000	2.941201000	1.018848000
6	1.836945000	2.859038000	2.013271000
1	0.929732000	1.523960000	0.609938000
1	2.085543000	3.675493000	1.315151000
1	1.601427000	3.313910000	2.993810000
6	4.695412000	0.900545000	3.343543000
1	4.972157000	0.610422000	4.369602000
1	5.428338000	1.634521000	2.984104000
6	3.322906000	1.560380000	3.383186000
1	3.387840000	2.404084000	4.101055000
1	2.554121000	0.874085000	3.765865000
8	0.604682000	-0.052627000	4.674211000
1	0.357948000	0.656874000	4.050437000
1	0.965772000	-0.704881000	4.040700000

^{5}RC1 $_{H_3O^+}$:

6	0.008850000	6.316442000	1.160954000
6	-0.576345000	5.933159000	-0.059672000
6	-1.226497000	6.920374000	-0.820875000
6	-1.295744000	8.244322000	-0.376770000
6	-0.709948000	8.609285000	0.839181000
6	-0.056506000	7.638461000	1.606986000
6	-0.517115000	4.516550000	-0.526878000
6	0.779433000	3.977187000	-0.799520000
7	1.027317000	2.686425000	-1.211515000
6	2.386547000	2.561190000	-1.385122000
6	3.024539000	3.801561000	-1.055753000
6	2.018472000	4.691875000	-0.709145000
26	-0.334670000	1.106066000	-1.257318000
7	-1.886827000	2.499164000	-1.014143000
6	-3.189432000	2.139193000	-0.725622000
6	-3.895429000	3.294089000	-0.190833000
6	-3.001017000	4.319812000	-0.129738000
6	-1.721603000	3.810304000	-0.608855000
6	-3.748662000	0.869879000	-0.871067000
6	-5.171475000	0.667083000	-0.461426000
6	-5.495122000	0.044756000	0.756385000
6	-6.817793000	-0.182706000	1.138195000
6	-7.854043000	0.203472000	0.285165000
6	-7.565097000	0.829784000	-0.930308000
6	-6.234955000	1.057234000	-1.290189000
6	3.052028000	1.387944000	-1.857277000
6	4.548564000	1.438403000	-1.896982000
6	5.173364000	2.178808000	-2.918214000
6	6.559111000	2.247267000	-3.056605000
6	7.369372000	1.558390000	-2.146902000
6	6.783058000	0.829342000	-1.116159000
6	5.385232000	0.766194000	-0.976708000
8	4.957765000	-0.021109000	0.053566000
6	3.742928000	0.248105000	0.754497000
6	3.890813000	0.031106000	2.261107000
7	4.428098000	-1.127995000	2.741128000
8	-4.433637000	-0.241599000	1.616681000
6	-4.421742000	-1.428033000	2.429339000
6	-3.997821000	-2.620048000	1.565759000
8	-4.763390000	-2.978775000	0.675475000
7	1.070463000	-0.021669000	-2.357841000
6	2.428575000	0.226941000	-2.318108000
6	3.137601000	-0.924266000	-2.854395000
6	2.198784000	-1.849105000	-3.204456000
6	0.894010000	-1.290312000	-2.871043000
6	-0.319926000	-1.987797000	-2.963314000
6	-0.314775000	-3.313709000	-3.642351000
6	-0.794797000	-4.467611000	-2.992747000
6	-0.782678000	-5.712189000	-3.624421000
6	-0.292105000	-5.836015000	-4.929455000
6	0.181076000	-4.699927000	-5.593346000
6	0.165428000	-3.454933000	-4.958222000
7	-1.759360000	-0.347064000	-1.747349000
6	-1.569733000	-1.525085000	-2.441011000
6	-2.830337000	-2.199088000	-2.578742000
6	-3.780310000	-1.419862000	-1.942496000
6	-3.100212000	-0.261922000	-1.450588000
8	3.504436000	0.933444000	3.006762000
1	-1.168096000	-4.373204000	-1.974158000
1	4.218538000	-1.008566000	-2.943035000
1	2.372274000	-2.839993000	-3.615736000
1	4.093042000	3.996705000	-1.103705000
1	2.130587000	5.736321000	-0.429249000
1	-3.176404000	5.327254000	0.239159000
1	-4.938546000	3.301791000	0.117705000
1	-4.842612000	-1.619430000	-1.836608000
1	-2.998136000	-3.136286000	-3.101642000
1	4.529528000	2.702028000	-3.629070000
1	7.002808000	2.825807000	-3.869903000
1	8.457982000	1.593805000	-2.235010000
1	7.391273000	0.292431000	-0.385317000
1	-1.680876000	6.639839000	-1.773921000
1	-1.805907000	8.994752000	-0.986159000
1	-0.762062000	9.643689000	1.187555000
1	0.399010000	7.911739000	2.562306000
1	0.512412000	5.559850000	1.766701000
1	-5.996788000	1.539260000	-2.240858000
1	-8.373378000	1.136980000	-1.597458000
1	-8.889407000	0.021731000	0.581663000
1	-7.048375000	-0.649085000	2.096077000
1	-1.151610000	-6.592216000	-3.091230000
1	-0.281572000	-6.809659000	-5.425336000
1	0.558176000	-4.780620000	-6.616137000
1	0.524013000	-2.570390000	-5.488746000
1	3.457212000	1.298274000	0.643263000
1	2.918911000	-0.363623000	0.371947000
1	-3.758733000	-1.203306000	3.267072000
1	-5.422247000	-1.634890000	2.828629000
7	-2.795060000	-3.213062000	1.798537000
8	-0.462086000	-1.513194000	4.099058000
8	-0.201456000	-3.713242000	0.372631000
8	2.264139000	-1.886784000	4.531882000
8	2.396666000	-2.749423000	1.381387000
6	-1.015881000	-4.768033000	0.838240000

1	-0.637997000	-5.130739000	1.810793000
6	-2.469161000	-4.337325000	0.905223000
1	-0.987079000	-5.625197000	0.138476000
1	-3.062688000	-5.222920000	1.198245000
1	-2.808729000	-4.051001000	-0.099088000
6	1.070821000	-4.109941000	-0.115604000
1	1.453095000	-4.977962000	0.454073000
1	0.992867000	-4.412086000	-1.174969000
6	2.032393000	-2.948525000	0.021647000
1	2.923363000	-3.119088000	-0.603755000
1	1.542851000	-2.028290000	-0.323247000
6	3.613506000	-3.323009000	1.822655000
1	3.410883000	-3.778873000	2.799827000
1	3.940957000	-4.134640000	1.148096000
6	4.733270000	-2.285046000	1.904552000
1	5.634280000	-2.776089000	2.308549000
1	4.985628000	-1.935756000	0.898338000
6	-1.995312000	-2.938178000	2.995471000
6	-1.120354000	-1.698027000	2.863209000
1	-2.644605000	-2.846455000	3.879345000
1	-1.329513000	-3.786261000	3.186886000
1	-1.729536000	-0.810868000	2.610673000
1	-0.404092000	-1.833069000	2.039225000
6	0.441037000	-0.420546000	4.124490000
1	-0.066527000	0.496571000	4.482939000
6	1.607021000	-0.733989000	5.034609000
1	0.819045000	-0.207901000	3.113794000
1	2.281868000	0.136901000	5.027995000
1	1.268257000	-0.919278000	6.072752000
6	4.626105000	-1.234489000	4.182995000
1	5.624667000	-1.661922000	4.375477000
1	4.614534000	-0.212007000	4.576973000
6	3.597217000	-2.099624000	4.933449000
1	3.724372000	-1.924517000	6.018955000
1	3.795875000	-3.166363000	4.755308000
8	1.011415000	-4.139394000	3.413600000
1	1.175311000	-3.403784000	4.034309000
1	1.213298000	-3.680320000	2.575989000
6	-0.140339000	0.850836000	0.704149000
8	0.734197000	-0.011953000	0.726513000
8	-0.713893000	1.476391000	1.621596000
8	-3.016764000	1.703776000	2.355211000
1	-3.598178000	0.905776000	2.042122000
1	-2.114205000	1.654601000	1.976569000
1	-2.960471000	1.717116000	3.326205000

^{5}IM1$_{H_3O^+}$:

6	2.417624000	5.685050000	-1.681199000
6	2.617282000	5.350408000	-0.331523000
6	3.384657000	6.213720000	0.467622000
6	3.931684000	7.384147000	-0.065449000
6	3.720145000	7.710022000	-1.409344000
6	2.960693000	6.856704000	-2.216088000
6	2.041997000	4.093227000	0.231925000
6	0.674098000	4.001816000	0.425526000
7	-0.023325000	2.925619000	0.961170000
6	-1.360657000	3.306405000	0.990650000
6	-1.497253000	4.642317000	0.449422000
6	-0.250594000	5.074418000	0.117817000
26	0.678121000	1.075883000	1.281720000
7	2.556381000	1.749097000	0.885562000
6	3.701520000	0.977532000	0.836665000
6	4.817155000	1.772651000	0.453042000
6	4.332939000	3.055633000	0.225013000
6	2.931673000	3.017595000	0.480819000
6	3.768009000	-0.412406000	1.094918000
6	5.063031000	-1.092239000	0.795399000
6	5.316868000	-1.584089000	-0.497497000
6	6.537630000	-2.189678000	-0.809784000
6	7.516389000	-2.326402000	0.176119000
6	7.281880000	-1.851317000	1.470264000
6	6.064605000	-1.232717000	1.766148000
6	-2.423725000	2.564092000	1.472053000
6	-3.789439000	3.178145000	1.475577000
6	-4.063837000	4.161102000	2.446508000
6	-5.315388000	4.759973000	2.576371000
6	-6.347458000	4.371078000	1.714931000
6	-6.105657000	3.409651000	0.738715000
6	-4.840157000	2.810148000	0.605256000
8	-4.770380000	1.874918000	-0.382331000
6	-3.532945000	1.537891000	-1.004510000
6	-3.810212000	0.848387000	-2.334908000
7	-4.523057000	-0.315393000	-2.329977000
8	4.344891000	-1.388035000	-1.457980000
6	3.930898000	-2.479960000	-2.281062000
6	3.138187000	-3.465883000	-1.415379000
8	3.683144000	-3.913313000	-0.408567000
7	-1.087519000	0.575903000	2.161508000
6	-2.275636000	1.276217000	2.042071000
6	-3.347190000	0.528746000	2.605715000
6	-2.801182000	-0.647402000	3.092468000
6	-1.403666000	-0.603879000	2.809917000
6	-0.498450000	-1.660776000	3.103525000
6	-0.992123000	-2.771639000	3.969192000
6	-1.023772000	-4.111115000	3.541986000
6	-1.491907000	-5.126307000	4.381210000
6	-1.943204000	-4.824649000	5.668917000
6	-1.919020000	-3.496931000	6.110326000
6	-1.449904000	-2.485632000	5.270183000
7	1.438400000	-0.672029000	1.905059000
6	0.806336000	-1.678271000	2.628250000
6	1.727268000	-2.783052000	2.819677000
6	2.880386000	-2.470371000	2.170673000
6	2.722359000	-1.134644000	1.639695000
8	-3.384217000	1.359015000	-3.370591000
1	-0.683389000	-4.363100000	2.538710000
1	-4.384034000	0.852836000	2.646032000
1	-3.318096000	-1.468389000	3.581007000
1	-2.438199000	5.177610000	0.354756000
1	0.034073000	6.036272000	-0.300031000
1	4.889789000	3.932582000	-0.093429000
1	5.837275000	1.413096000	0.346676000
1	3.779984000	-3.067568000	2.066459000
1	1.507254000	-3.688242000	3.377327000
1	-3.253038000	4.444083000	3.121805000
1	-5.485853000	5.516593000	3.345150000
1	-7.339122000	4.822465000	1.796838000
1	-6.887970000	3.098528000	0.043514000
1	3.548623000	5.961803000	1.517957000
1	4.522370000	8.046593000	0.572193000
1	4.146628000	8.625504000	-1.826383000
1	2.793798000	7.101445000	-3.268023000
1	1.830835000	5.013488000	-2.312358000
1	5.871866000	-0.850741000	2.771313000
1	8.045341000	-1.957554000	2.243975000
1	8.467625000	-2.803111000	-0.071955000
1	6.722172000	-2.557157000	-1.820464000
1	-1.508532000	-6.157945000	4.021157000
1	-2.311222000	-5.617370000	6.324577000
1	-2.262934000	-3.247766000	7.117329000
1	-1.430086000	-1.452336000	5.622691000
1	-2.959272000	2.442832000	-1.239479000
1	-2.913178000	0.895855000	-0.368919000
1	3.359985000	-2.026648000	-3.095338000

1	4.794537000	-3.006867000	-2.715208000
7	1.855986000	-3.779482000	-1.739219000
8	0.181211000	-1.841454000	-4.400722000
8	-1.172679000	-3.985430000	-1.160484000
8	-2.587235000	-1.726589000	-3.992868000
8	-3.784585000	-3.004384000	-1.092955000
6	-0.238156000	-5.046247000	-1.054587000
1	-0.275690000	-5.590357000	-2.009369000
6	1.173378000	-4.604685000	-0.725759000
1	-0.553349000	-5.752499000	-0.265652000
1	1.775230000	-5.510876000	-0.546408000
1	1.185836000	-4.051783000	0.226846000
6	-1.762476000	-3.567203000	0.058760000
1	-2.222564000	-4.438709000	0.561827000
1	-1.002222000	-3.138947000	0.735369000
6	-2.803686000	-2.504083000	-0.199449000
1	-3.247689000	-2.238848000	0.778411000
1	-2.323058000	-1.600420000	-0.603432000
6	-5.056198000	-2.411180000	-1.000890000
1	-5.667755000	-2.883819000	-1.784068000
1	-5.533374000	-2.655696000	-0.029443000
6	-5.106978000	-0.886989000	-1.117820000
1	-6.171619000	-0.598878000	-1.038257000
1	-4.608700000	-0.447107000	-0.249089000
6	1.254926000	-3.441038000	-3.031886000
6	0.618126000	-2.058457000	-3.070765000
1	2.013883000	-3.519294000	-3.824065000
1	0.478747000	-4.172594000	-3.281563000
1	1.343711000	-1.283415000	-2.766135000
1	-0.218715000	-2.015974000	-2.359883000
6	-0.564587000	-0.648448000	-4.600058000
1	-0.085027000	-0.054329000	-5.399806000
6	-1.991169000	-0.949327000	-5.016273000
1	-0.567177000	-0.038675000	-3.684427000
1	-2.526908000	0.005159000	-5.146935000
1	-2.011637000	-1.510151000	-5.972044000
6	-4.881989000	-0.878288000	-3.627788000
1	-5.912601000	-1.266152000	-3.580078000
1	-4.870841000	-0.053319000	-4.350893000
6	-3.966043000	-2.011115000	-4.107697000
1	-4.232615000	-2.253626000	-5.154629000
1	-4.116580000	-2.913181000	-3.504095000
8	-1.940063000	-4.430986000	-3.753925000
1	-1.912780000	-3.495576000	-4.032311000
1	-1.932924000	-4.309497000	-2.784656000
6	0.122722000	0.568795000	-0.779578000
8	-0.976739000	0.070586000	-0.966561000
8	0.776230000	1.024737000	-1.865126000
8	3.393116000	1.174267000	-2.203442000
1	3.739686000	0.302817000	-1.922954000
1	1.745699000	1.190626000	-1.724980000
1	3.884194000	1.795998000	-1.638397000

^{5}RC2 $_{H3O+}$:

6	1.944366000	5.820038000	-1.535638000
6	2.286894000	5.448892000	-0.224973000
6	3.110919000	6.301705000	0.526640000
6	3.579711000	7.500098000	-0.018760000
6	3.231784000	7.861469000	-1.324169000
6	2.412626000	7.017839000	-2.081513000
6	1.786619000	4.161238000	0.345378000
6	0.413608000	4.061159000	0.627256000
7	-0.232525000	2.953764000	1.119928000
6	-1.561460000	3.265726000	1.225678000
6	-1.773306000	4.639254000	0.785893000
6	-0.553980000	5.133300000	0.428673000
26	0.547451000	0.999535000	1.248538000
7	2.458404000	1.843417000	0.936763000
6	3.616347000	1.117200000	0.893793000
6	4.696791000	1.976789000	0.454270000
6	4.143585000	3.220397000	0.211861000
6	2.736813000	3.122952000	0.519280000
6	3.759258000	-0.256771000	1.209424000
6	5.096914000	-0.859574000	0.916912000
6	5.416602000	-1.288130000	-0.380668000
6	6.664786000	-1.819827000	-0.696978000
6	7.622716000	-1.957488000	0.309107000
6	7.329832000	-1.550093000	1.614017000
6	6.080252000	-0.999470000	1.906897000
6	-2.580916000	2.395839000	1.656984000
6	-3.982959000	2.924227000	1.634379000
6	-4.366510000	3.833277000	2.638515000
6	-5.657231000	4.350900000	2.723063000
6	-6.611523000	3.956230000	1.778369000
6	-6.257831000	3.067766000	0.768061000
6	-4.954014000	2.547082000	0.680379000
8	-4.776828000	1.664588000	-0.341803000
6	-3.507054000	1.419210000	-0.944253000
6	-3.726192000	0.810223000	-2.327752000
7	-4.405003000	-0.372401000	-2.417390000
8	4.454788000	-1.109435000	-1.386999000
6	4.058650000	-2.214615000	-2.228509000
6	3.265113000	-3.207974000	-1.378119000
8	3.775000000	-3.595943000	-0.330191000
7	-1.196695000	0.418514000	2.250740000
6	-2.394749000	1.080536000	2.136043000
6	-3.460355000	0.223209000	2.610321000
6	-2.876834000	-0.946474000	3.020535000
6	-1.449788000	-0.814187000	2.792301000
6	-0.486933000	-1.822924000	3.064912000
6	-0.952725000	-3.013704000	3.836961000
6	-0.826178000	-4.322847000	3.339313000
6	-1.272374000	-5.420141000	4.079479000
6	-1.852228000	-5.232128000	5.337611000
6	-1.978649000	-3.937012000	5.849983000
6	-1.533630000	-2.841331000	5.107494000
7	1.456315000	-0.739332000	1.981967000
6	0.869325000	-1.763801000	2.682260000
6	1.870558000	-2.791136000	2.952447000
6	3.031379000	-2.383631000	2.367928000
6	2.775199000	-1.073793000	1.787641000
8	-3.294795000	1.401074000	-3.316087000
1	-0.380181000	-4.484108000	2.358975000
1	-4.515402000	0.484334000	2.635877000
1	-3.373490000	-1.822934000	3.427428000
1	-2.730451000	5.153520000	0.767663000
1	-0.322658000	6.130688000	0.064015000
1	4.659562000	4.114720000	-0.127611000
1	5.745230000	1.694694000	0.381159000
1	3.984048000	-2.902820000	2.339264000
1	1.704182000	-3.704328000	3.516446000
1	-3.614333000	4.123330000	3.375747000
1	-5.917608000	5.050276000	3.520130000
1	-7.631040000	4.346362000	1.823463000
1	-6.979340000	2.752884000	0.011659000
1	3.379719000	6.022579000	1.547923000
1	4.216681000	8.155473000	0.580079000
1	3.598362000	8.798255000	-1.750426000
1	2.139543000	7.291326000	-3.103526000
1	1.309857000	5.156170000	-2.127267000
1	5.848549000	-0.669083000	2.921485000
1	8.077671000	-1.653870000	2.402806000
1	8.601396000	-2.377488000	0.068056000
1	6.887251000	-2.126457000	-1.720025000

1	-1.169510000	-6.426523000	3.666792000
1	-2.202003000	-6.090061000	5.916516000
1	-2.422043000	-3.777971000	6.835883000
1	-1.626786000	-1.833648000	5.517210000
1	-2.961859000	2.358241000	-1.100498000
1	-2.879869000	0.755157000	-0.338327000
1	3.502623000	-1.759722000	-3.051258000
1	4.946555000	-2.716222000	-2.638422000
7	2.036978000	-3.613901000	-1.785125000
8	0.338772000	-1.687989000	-4.444566000
8	-0.987545000	-3.986429000	-1.312361000
8	-2.447773000	-1.702072000	-4.161943000
8	-3.608272000	-3.082298000	-1.305467000
6	-0.009230000	-5.006103000	-1.216756000
1	0.011419000	-5.511910000	-2.192717000
6	1.364656000	-4.505493000	-0.821515000
1	-0.313841000	-5.758735000	-0.467532000
1	2.009160000	-5.382666000	-0.645733000
1	1.312947000	-3.981358000	0.145806000
6	-1.620851000	-3.633083000	-0.095385000
1	-2.066328000	-4.535939000	0.363733000
1	-0.892421000	-3.207754000	0.616985000
6	-2.683979000	-2.590887000	-0.348747000
1	-3.178970000	-2.386956000	0.618953000
1	-2.213699000	-1.655742000	-0.692926000
6	-4.908541000	-2.553565000	-1.227314000
1	-5.471332000	-3.007431000	-2.056614000
1	-5.406806000	-2.872052000	-0.288418000
6	-5.017545000	-1.029339000	-1.264556000
1	-6.094974000	-0.784436000	-1.218022000
1	-4.575545000	-0.620814000	-0.352029000
6	1.469085000	-3.271019000	-3.093810000
6	0.767331000	-1.919300000	-3.114962000
1	2.257681000	-3.298989000	-3.860206000
1	0.734479000	-4.029149000	-3.385855000
1	1.446833000	-1.114069000	-2.777813000
1	-0.078503000	-1.933059000	-2.415744000
6	-0.446271000	-0.514206000	-4.615374000
1	0.043431000	0.144390000	-5.356780000
6	-1.840974000	-0.843557000	-5.111093000
1	-0.525065000	0.034173000	-3.665705000
1	-2.404713000	0.099363000	-5.205448000
1	-1.806179000	-1.343345000	-6.099780000
6	-4.737219000	-0.851535000	-3.756263000
1	-5.772500000	-1.231171000	-3.754064000
1	-4.704463000	0.017346000	-4.425124000
6	-3.827618000	-1.962977000	-4.297068000
1	-4.095127000	-2.139039000	-5.357238000
1	-3.988475000	-2.899598000	-3.750802000
8	-1.703614000	-4.369315000	-3.933045000
1	-1.730006000	-3.426736000	-4.188885000
1	-1.716853000	-4.274346000	-2.961223000
6	0.074728000	0.642231000	-0.775618000
8	-0.891825000	0.020936000	-1.203025000
8	0.790693000	1.313334000	-1.766505000
8	3.997382000	1.173442000	-2.184591000
1	4.211582000	0.166635000	-1.875860000
1	1.527864000	1.758284000	-1.322589000
1	4.244854000	1.768642000	-1.409874000
1	4.536199000	1.422187000	-2.958784000

[5]IM2 $_{H3O+}$:

6	-1.234677000	5.552217000	1.487261000
6	-1.737242000	5.433700000	0.179921000
6	-2.477879000	6.490369000	-0.367911000
6	-2.708130000	7.651163000	0.376992000
6	-2.203725000	7.764070000	1.676460000
6	-1.467212000	6.711315000	2.230845000
6	-1.479737000	4.167329000	-0.570187000
6	-0.150804000	3.933408000	-0.980429000
7	0.345536000	2.754082000	-1.494531000
6	1.692066000	2.928182000	-1.713625000
6	2.062678000	4.271171000	-1.325357000
6	0.925848000	4.891204000	-0.877382000
26	-0.713850000	0.993408000	-1.753309000
7	-2.462881000	1.988967000	-1.203535000
6	-3.679279000	1.388435000	-1.024056000
6	-4.598737000	2.335352000	-0.417734000
6	-3.899874000	3.492290000	-0.212495000
6	-2.548495000	3.259522000	-0.696800000
6	-3.992631000	0.047346000	-1.320069000
6	-5.302063000	-0.465675000	-0.814397000
6	-5.423341000	-0.785069000	0.553138000
6	-6.618915000	-1.304041000	1.058186000
6	-7.701460000	-1.513547000	0.200509000
6	-7.599682000	-1.199092000	-1.157430000
6	-6.402578000	-0.675977000	-1.653745000
6	2.589991000	1.946572000	-2.187646000
6	4.035362000	2.329886000	-2.244782000
6	4.475965000	3.204983000	-3.253099000
6	5.814350000	3.576968000	-3.373268000
6	6.750965000	3.069504000	-2.466278000
6	6.337958000	2.209956000	-1.450927000
6	4.990492000	1.838013000	-1.330948000
8	4.720507000	0.940728000	-0.335906000
6	3.545225000	1.022580000	0.463933000
6	3.862858000	0.760074000	1.939403000
7	4.509283000	-0.386284000	2.295962000
8	-4.348955000	-0.495854000	1.360647000
6	-3.943751000	-1.375340000	2.400629000
6	-3.204054000	-2.566976000	1.783734000
8	-3.652641000	-3.059314000	0.751830000
7	0.982073000	0.111300000	-2.610796000
6	2.246549000	0.643565000	-2.593561000
6	3.192782000	-0.360475000	-3.048251000
6	2.475100000	-1.489490000	-3.323206000
6	1.078193000	-1.184567000	-3.049429000
6	0.008949000	-2.099039000	-3.174185000
6	0.340431000	-3.468205000	-3.675108000
6	0.209802000	-4.591104000	-2.840660000
6	0.535889000	-5.867895000	-3.303764000
6	0.995455000	-6.043279000	-4.612858000
6	1.125664000	-4.933864000	-5.454223000
6	0.801594000	-3.656938000	-4.988541000
7	-1.829765000	-0.668488000	-2.306236000
6	-1.337141000	-1.842995000	-2.824782000
6	-2.410497000	-2.815349000	-2.878424000
6	-3.514020000	-2.230037000	-2.319652000
6	-3.143989000	-0.879941000	-1.958223000
8	3.499767000	1.596932000	2.764092000
1	-0.147223000	-4.458841000	-1.818097000
1	4.266230000	-0.216030000	-3.140440000
1	2.853807000	-2.443781000	-3.678276000
1	3.063256000	4.690005000	-1.386707000
1	0.826640000	5.909193000	-0.510315000
1	-4.260584000	4.418925000	0.226217000
1	-5.640207000	2.133817000	-0.179161000
1	-4.491821000	-2.673544000	-2.158897000
1	-2.328786000	-3.823552000	-3.274237000
1	3.737258000	3.586067000	-3.961727000
1	6.124824000	4.253821000	-4.171808000
1	7.804205000	3.348730000	-2.544062000
1	7.045598000	1.807296000	-0.723905000
1	-2.870359000	6.401717000	-1.383455000

1	-3.282443000	8.471242000	-0.060556000
1	-2.385229000	8.671506000	2.257260000
1	-1.075106000	6.791972000	3.247561000
1	-0.670911000	4.715345000	1.907601000
1	-6.306927000	-0.429919000	-2.713614000
1	-8.447070000	-1.359512000	-1.827031000
1	-8.632287000	-1.920045000	0.602669000
1	-6.709964000	-1.544517000	2.118078000
1	0.433414000	-6.727550000	-2.637388000
1	1.250740000	-7.041347000	-4.976449000
1	1.479079000	-5.062355000	-6.480054000
1	0.901457000	-2.792755000	-5.648828000
1	3.112342000	2.028178000	0.434050000
1	2.785612000	0.309048000	0.103458000
1	-3.331587000	-0.762950000	3.068764000
1	-4.800112000	-1.752521000	2.981909000
7	-2.088443000	-3.054522000	2.389336000
8	-0.321321000	-0.772561000	4.707762000
8	0.894499000	-3.873805000	2.086629000
8	2.437680000	-1.002459000	4.251474000
8	3.600143000	-3.245172000	1.864763000
6	-0.202757000	-4.758543000	2.245870000
1	-0.272228000	-4.981597000	3.319702000
6	-1.506165000	-4.217584000	1.696661000
1	0.000916000	-5.712897000	1.729051000
1	-2.243689000	-5.038225000	1.704888000
1	-1.379417000	-3.949649000	0.636754000
6	1.566390000	-3.962685000	0.844188000
1	1.955057000	-4.988492000	0.700814000
1	0.882574000	-3.736220000	0.008301000
6	2.705724000	-2.972208000	0.807474000
1	3.201176000	-3.066383000	-0.177581000
1	2.311428000	-1.944726000	0.888156000
6	4.925518000	-2.806022000	1.677424000
1	5.461393000	-3.065093000	2.602078000
1	5.410505000	-3.368337000	0.854220000
6	5.094216000	-1.325530000	1.340432000
1	6.180551000	-1.139786000	1.250666000
1	4.676590000	-1.129487000	0.349842000
6	-1.551772000	-2.508845000	3.639491000
6	-0.647764000	-1.295804000	3.434233000
1	-2.376424000	-2.241084000	4.315696000
1	-0.969950000	-3.279193000	4.157104000
1	-1.166951000	-0.527821000	2.833809000
1	0.253874000	-1.602020000	2.885998000
6	0.529516000	0.365523000	4.658481000
1	0.131998000	1.126195000	5.353064000
6	1.951070000	0.040548000	5.078271000
1	0.529475000	0.805654000	3.648183000
1	2.566073000	0.949846000	4.967768000
1	1.979111000	-0.283763000	6.137150000
6	4.807992000	-0.556156000	3.715806000
1	5.795709000	-1.031980000	3.821636000
1	4.870203000	0.444360000	4.161180000
6	3.777305000	-1.407574000	4.464655000
1	4.024021000	-1.390140000	5.543076000
1	3.817096000	-2.448731000	4.125870000
8	1.510042000	-3.614845000	4.753127000
1	1.571534000	-2.642097000	4.769184000
1	1.559363000	-3.773147000	3.790863000
6	-0.342178000	-0.222635000	-0.027657000
8	-0.084436000	-1.216161000	0.454948000
8	0.062198000	2.078517000	1.684604000
8	-2.604611000	1.567240000	2.075146000
1	-3.151284000	0.932502000	1.572022000
1	0.192986000	2.336317000	0.761668000
1	-0.900827000	1.887372000	1.749212000
1	-3.076295000	2.404405000	1.951337000

[2]CAT $_{H3O+,K+}$:

6	-3.665152000	-3.228626000	-1.768723000
6	-2.555427000	-3.261791000	-2.635540000
6	-2.508769000	-4.249882000	-3.632016000
6	-3.539500000	-5.187043000	-3.758981000
6	-4.633018000	-5.150598000	-2.888574000
6	-4.691394000	-4.167660000	-1.893192000
6	-1.452604000	-2.274949000	-2.448912000
6	-1.771885000	-0.900500000	-2.588981000
7	-0.990901000	0.144630000	-2.113122000
6	-1.757848000	1.284267000	-2.281091000
6	-2.983235000	0.969386000	-2.927463000
6	-2.990163000	-0.399495000	-3.122081000
26	0.951604000	0.065424000	-1.636888000
7	0.897524000	-1.928166000	-1.738275000
6	1.887516000	-2.802632000	-1.301517000
6	1.374516000	-4.159183000	-1.275244000
6	0.094363000	-4.112996000	-1.730913000
6	-0.212608000	-2.723767000	-2.020780000
6	3.175502000	-2.455389000	-0.937319000
6	4.100251000	-3.462182000	-0.342943000
6	4.420092000	-3.352307000	1.029982000
6	5.363744000	-4.196751000	1.621963000
6	5.987013000	-5.184543000	0.851520000
6	5.660456000	-5.332105000	-0.498896000
6	4.725484000	-4.471445000	-1.085758000
6	-1.409994000	2.569446000	-1.787725000
6	-2.468063000	3.615447000	-1.731194000
6	-2.387227000	4.766764000	-2.528445000
6	-3.351453000	5.777261000	-2.471548000
6	-4.426273000	5.644701000	-1.593011000
6	-4.540099000	4.507708000	-0.785693000
6	-3.577324000	3.493022000	-0.860657000
8	-3.640632000	2.362545000	-0.088747000
6	-4.864823000	2.041904000	0.544357000
6	-4.913923000	0.521182000	0.706952000
7	-5.360502000	-0.016324000	1.874502000
8	3.720181000	-2.426034000	1.748972000
6	4.378307000	-1.542468000	2.642604000
6	3.829829000	-0.126238000	2.435660000
8	4.618771000	0.804053000	2.298440000
7	0.983366000	2.046473000	-1.440245000
6	-0.121550000	2.888487000	-1.379011000
6	0.285277000	4.200508000	-0.908084000
6	1.639791000	4.176594000	-0.779279000
6	2.083995000	2.841172000	-1.123475000
6	3.408857000	2.430868000	-1.080659000
6	4.462583000	3.457708000	-0.834266000
6	5.249613000	3.418427000	0.332155000
6	6.214547000	4.401076000	0.569067000
6	6.414930000	5.435372000	-0.352187000
6	5.640176000	5.481862000	-1.515214000
6	4.672757000	4.501063000	-1.752340000
7	2.905752000	0.001611000	-1.209401000
6	3.791899000	1.070801000	-1.160628000
6	5.127087000	0.591349000	-1.028901000
6	5.050710000	-0.784689000	-0.919988000
6	3.671180000	-1.128977000	-1.027557000
8	-4.603886000	-0.168744000	-0.259465000
1	5.090344000	2.614380000	1.053731000
1	-0.387930000	5.032634000	-0.721204000
1	2.292286000	4.984505000	-0.458929000
1	-3.762183000	1.686865000	-3.173889000
1	-3.777454000	-1.007629000	-3.559992000
1	-0.601669000	-4.939398000	-1.846157000

1	1.941006000	-5.029336000	-0.953017000
1	5.865147000	-1.492380000	-0.785385000
1	6.016882000	1.212969000	-0.985207000
1	-1.540409000	4.858941000	-3.211767000
1	-3.260391000	6.659394000	-3.108778000
1	-5.185971000	6.426675000	-1.523888000
1	-5.374818000	4.432049000	-0.089778000
1	-1.655100000	-4.280021000	-4.313042000
1	-3.487995000	-5.947253000	-4.542519000
1	-5.437167000	-5.884133000	-2.985836000
1	-5.540350000	-4.133775000	-1.205179000
1	-3.717719000	-2.463995000	-0.990942000
1	4.488493000	-4.561775000	-2.148378000
1	6.141828000	-6.106858000	-1.099762000
1	6.722208000	-5.846123000	1.315811000
1	5.593305000	-4.093572000	2.684461000
1	6.811977000	4.362008000	1.483753000
1	7.170988000	6.201703000	-0.164066000
1	5.790553000	6.283274000	-2.242986000
1	4.069050000	4.537835000	-2.662004000
1	-4.974638000	2.615252000	1.472046000
1	-5.718456000	2.289337000	-0.109289000
1	4.247181000	-1.875102000	3.687672000
1	5.454102000	-1.488263000	2.427527000
7	2.475112000	0.026601000	2.378843000
19	-1.866547000	0.081856000	0.775830000
8	-0.499768000	-1.992498000	2.030002000
8	-0.310509000	1.773444000	2.619288000
8	-3.344862000	-2.248388000	1.746118000
8	-3.020535000	1.070310000	3.142308000
6	1.072074000	2.104152000	2.745490000
1	1.396495000	1.983010000	3.795838000
6	1.937474000	1.263259000	1.821245000
1	1.199790000	3.169167000	2.477878000
1	2.797047000	1.860522000	1.509013000
1	1.371075000	1.038146000	0.904291000
6	-1.107339000	2.494396000	3.544426000
1	-0.960781000	2.091743000	4.564599000
1	-0.810449000	3.560037000	3.557062000
6	-2.566819000	2.426240000	3.153832000
1	-3.155319000	3.024057000	3.867250000
1	-2.701751000	2.855117000	2.147907000
6	-4.155387000	0.718013000	3.929389000
1	-3.944882000	-0.287190000	4.315959000
1	-4.252450000	1.396293000	4.793038000
6	-5.460829000	0.722447000	3.128092000
1	-6.244227000	0.258247000	3.745446000
1	-5.793667000	1.749076000	2.938567000
6	1.520929000	-0.978983000	2.824022000
6	0.880179000	-1.801380000	1.702680000
1	1.995234000	-1.639684000	3.559348000
1	0.715838000	-0.457315000	3.359798000
1	1.371426000	-2.775795000	1.592152000
1	0.970644000	-1.283056000	0.735775000
6	-1.124069000	-3.104439000	1.402458000
1	-0.507790000	-4.009959000	1.538311000
6	-2.476298000	-3.333059000	2.027698000
1	-1.220459000	-2.932866000	0.317461000
1	-2.899059000	-4.271660000	1.622330000
1	-2.364374000	-3.456091000	3.121145000
6	-5.643332000	-1.452288000	1.881733000
1	-6.557565000	-1.614584000	2.473628000
1	-5.844627000	-1.746706000	0.844848000
6	-4.566727000	-2.363904000	2.451297000
1	-4.952579000	-3.397743000	2.372040000
1	-4.396960000	-2.169908000	3.526328000
8	-1.465838000	-0.959593000	4.439976000
1	-1.108688000	-1.464504000	3.685989000
1	-1.836150000	-0.175066000	4.001021000

^{3}CAT $_{H3O+,K+}$:

6	-3.647894000	-3.429704000	-1.762049000
6	-2.478697000	-3.516794000	-2.548559000
6	-2.366906000	-4.598201000	-3.441839000
6	-3.371623000	-5.567952000	-3.536135000
6	-4.518120000	-5.473217000	-2.742900000
6	-4.650042000	-4.396417000	-1.854762000
6	-1.403172000	-2.500617000	-2.397894000
6	-1.769185000	-1.120739000	-2.589890000
7	-1.051771000	-0.042398000	-2.074822000
6	-1.839343000	1.078573000	-2.313296000
6	-2.995319000	0.726002000	-3.026199000
6	-2.948781000	-0.668147000	-3.204518000
26	0.910340000	-0.034664000	-1.627151000
7	0.931136000	-2.040777000	-1.699629000
6	1.952026000	-2.864992000	-1.209994000
6	1.467554000	-4.234408000	-1.112956000
6	0.197054000	-4.252071000	-1.586490000
6	-0.155122000	-2.885514000	-1.958472000
6	3.216375000	-2.466426000	-0.856496000
6	4.169551000	-3.404872000	-0.205264000
6	4.530114000	-3.184921000	1.147280000
6	5.499733000	-3.968128000	1.779234000
6	6.115350000	-5.011586000	1.075929000
6	5.751606000	-5.271714000	-0.246870000
6	4.791421000	-4.467921000	-0.875181000
6	-1.537496000	2.396235000	-1.811222000
6	-2.630708000	3.399938000	-1.787518000
6	-2.551034000	4.563418000	-2.572699000
6	-3.545310000	5.546274000	-2.564795000
6	-4.667618000	5.372625000	-1.757213000
6	-4.790443000	4.221752000	-0.968521000
6	-3.791226000	3.240255000	-0.987216000
8	-3.867335000	2.106784000	-0.213392000
6	-5.090545000	1.848435000	0.453722000
6	-5.118679000	0.365748000	0.831031000
7	-5.304816000	0.000252000	2.131682000
8	3.833565000	-2.216688000	1.809432000
6	4.480174000	-1.314656000	2.687073000
6	3.924210000	0.092922000	2.446094000
8	4.710553000	1.025821000	2.304090000
7	0.879930000	1.960232000	-1.513028000
6	-0.259814000	2.766661000	-1.446247000
6	0.118019000	4.101735000	-0.996413000
6	1.472541000	4.132393000	-0.913796000
6	1.966693000	2.806068000	-1.249688000
6	3.297570000	2.450888000	-1.221951000
6	4.319054000	3.515696000	-1.020740000
6	5.166901000	3.511464000	0.106031000
6	6.097130000	4.534468000	0.304338000
6	6.210105000	5.583407000	-0.617345000
6	5.379263000	5.598472000	-1.741262000
6	4.448258000	4.573638000	-1.940015000
7	2.876383000	-0.012434000	-1.216386000
6	3.734881000	1.084588000	-1.261597000
6	5.074016000	0.653570000	-1.175236000
6	5.042442000	-0.737981000	-1.005817000
6	3.685262000	-1.117283000	-1.026047000
8	-5.014179000	-0.457580000	-0.073043000
1	5.077082000	2.702556000	0.833922000
1	-0.578592000	4.911331000	-0.793601000
1	2.100037000	4.971409000	-0.623009000
1	-3.779959000	1.415034000	-3.333641000
1	-3.691773000	-1.302714000	-3.683470000

1	-0.470529000	-5.106573000	-1.661618000
1	2.051538000	-5.071062000	-0.735547000
1	5.884804000	-1.415921000	-0.881788000
1	5.948288000	1.299569000	-1.189243000
1	-1.673611000	4.685064000	-3.211053000
1	-3.443001000	6.435516000	-3.191088000
1	-5.457630000	6.126742000	-1.729606000
1	-5.666106000	4.115063000	-0.330277000
1	-1.476919000	-4.673912000	-4.070907000
1	-3.259013000	-6.398017000	-4.238868000
1	-5.304374000	-6.228815000	-2.815851000
1	-5.539802000	-4.314010000	-1.224224000
1	-3.762196000	-2.597428000	-1.064441000
1	4.529473000	-4.646180000	-1.920872000
1	6.224897000	-6.088355000	-0.797239000
1	6.871058000	-5.625178000	1.572476000
1	5.754708000	-3.779197000	2.824195000
1	6.735917000	4.518746000	1.191938000
1	6.939974000	6.381546000	-0.459444000
1	5.458853000	6.408517000	-2.471292000
1	3.804847000	4.586015000	-2.822871000
1	-5.216693000	2.546070000	1.289787000
1	-5.941088000	1.985336000	-0.234614000
1	4.347985000	-1.624952000	3.739776000
1	5.557119000	-1.256372000	2.476870000
7	2.570645000	0.235613000	2.366971000
19	-1.837020000	0.170885000	0.770725000
8	-0.381428000	-1.833083000	2.042882000
8	-0.231756000	1.986717000	2.472041000
8	-3.212729000	-2.148579000	1.902260000
8	-2.921003000	1.303463000	3.100837000
6	1.149662000	2.321752000	2.606848000
1	1.451366000	2.258105000	3.669508000
6	2.032747000	1.438680000	1.740949000
1	1.281555000	3.370904000	2.284680000
1	2.891480000	2.024752000	1.407819000
1	1.482506000	1.162450000	0.828787000
6	-1.051310000	2.827259000	3.264277000
1	-0.904132000	2.600922000	4.337874000
1	-0.779784000	3.888386000	3.104299000
6	-2.506244000	2.654471000	2.889559000
1	-3.115299000	3.345625000	3.492982000
1	-2.656867000	2.907594000	1.827173000
6	-4.005150000	1.054414000	3.989535000
1	-3.757571000	0.122999000	4.514261000
1	-4.078369000	1.853458000	4.745672000
6	-5.344109000	0.921533000	3.262538000
1	-6.090007000	0.555378000	3.984842000
1	-5.697674000	1.904470000	2.934965000
6	1.614717000	-0.764892000	2.818210000
6	0.991106000	-1.607146000	1.704327000
1	2.082360000	-1.410517000	3.571480000
1	0.802065000	-0.234965000	3.334832000
1	1.506316000	-2.568038000	1.592051000
1	1.060384000	-1.093050000	0.733100000
6	-1.000614000	-2.917435000	1.363108000
1	-0.350313000	-3.807635000	1.396527000
6	-2.307374000	-3.232506000	2.043198000
1	-1.156496000	-2.674986000	0.297786000
1	-2.737853000	-4.148246000	1.595909000
1	-2.116787000	-3.433388000	3.113348000
6	-5.488106000	-1.426963000	2.400374000
1	-6.252939000	-1.530016000	3.185584000
1	-5.871191000	-1.882778000	1.479475000
6	-4.260095000	-2.203288000	2.855521000
1	-4.590365000	-3.248884000	2.997920000
1	-3.879982000	-1.846580000	3.828770000
8	-1.380288000	-0.718662000	4.402254000
1	-1.032155000	-1.249303000	3.661169000
1	-1.707472000	0.077941000	3.950119000

^{3}RC1 $_{H3O+,K+}$:

6	2.385704000	4.995257000	-1.734917000
6	1.149315000	4.803719000	-2.379389000
6	0.608753000	5.872554000	-3.116214000
6	1.286751000	7.089554000	-3.217675000
6	2.518738000	7.264136000	-2.577444000
6	3.065025000	6.212433000	-1.835055000
6	0.423411000	3.508214000	-2.259201000
6	1.050422000	2.346712000	-2.685131000
7	0.595141000	1.050007000	-2.499169000
6	1.626177000	0.217142000	-2.882875000
6	2.716546000	1.003738000	-3.430107000
6	2.356202000	2.311420000	-3.327508000
26	-1.186248000	0.495113000	-1.772946000
7	-1.684198000	2.426002000	-1.463327000
6	-2.750244000	2.848457000	-0.698806000
6	-2.571890000	4.215285000	-0.313136000
6	-1.377650000	4.638387000	-0.916417000
6	-0.839816000	3.521613000	-1.600241000
6	-3.844625000	2.038024000	-0.308197000
6	-4.751328000	2.507277000	0.776436000
6	-4.562651000	2.006645000	2.083691000
6	-5.394860000	2.387323000	3.138766000
6	-6.445549000	3.277439000	2.898999000
6	-6.646971000	3.797183000	1.617858000
6	-5.800125000	3.415253000	0.573533000
6	1.644180000	-1.166742000	-2.766375000
6	2.913923000	-1.911239000	-2.989990000
6	3.055995000	-2.763252000	-4.101110000
6	4.215071000	-3.508260000	-4.314600000
6	5.268234000	-3.425964000	-3.397932000
6	5.152445000	-2.592927000	-2.285579000
6	3.989637000	-1.837702000	-2.085494000
8	3.890142000	-1.036797000	-0.954648000
6	4.786721000	0.074561000	-0.942059000
6	4.849570000	0.748384000	0.426551000
7	5.311667000	0.032546000	1.499878000
8	-3.463499000	1.179479000	2.299427000
6	-3.595531000	0.048692000	3.184270000
6	-3.089762000	-1.213804000	2.465388000
8	-3.810361000	-1.696815000	1.596552000
7	-0.786480000	-1.400141000	-2.346965000
6	0.483472000	-1.925581000	-2.484115000
6	0.445620000	-3.350372000	-2.386760000
6	-0.882612000	-3.700418000	-2.227533000
6	-1.631101000	-2.484856000	-2.202715000
6	-3.031184000	-2.421490000	-1.990745000
6	-3.782987000	-3.708896000	-1.964617000
6	-4.365500000	-4.158771000	-0.765255000
6	-5.047446000	-5.378149000	-0.721540000
6	-5.156956000	-6.167085000	-1.871678000
6	-4.576913000	-5.730225000	-3.067708000
6	-3.891877000	-4.512897000	-3.111450000
7	-3.079103000	-0.020748000	-1.415183000
6	-3.687601000	-1.251024000	-1.651706000
6	-5.091209000	-1.177296000	-1.292530000
6	-5.291746000	0.037054000	-0.716477000
6	-4.031922000	0.750775000	-0.794097000
8	4.523969000	1.929066000	0.515006000
1	-4.265280000	-3.538621000	0.128474000
1	1.305722000	-4.011691000	-2.446931000
1	-1.300664000	-4.698021000	-2.122234000
1	3.621501000	0.589127000	-3.868513000

1	2.915169000	3.183903000	-3.654509000
1	-0.923973000	5.623869000	-0.860106000
1	-3.302953000	4.822174000	0.217944000
1	-6.208597000	0.443643000	-0.295490000
1	-5.812253000	-1.978334000	-1.431146000
1	2.226806000	-2.828638000	-4.808347000
1	4.296861000	-4.153045000	-5.192321000
1	6.178787000	-4.011179000	-3.543894000
1	5.969543000	-2.531522000	-1.564656000
1	-0.351863000	5.739008000	-3.618582000
1	0.853547000	7.904613000	-3.802864000
1	3.049111000	8.216254000	-2.655643000
1	4.023251000	6.341403000	-1.325461000
1	2.811851000	4.179045000	-1.146738000
1	-5.956607000	3.808729000	-0.433226000
1	-7.464511000	4.496624000	1.430748000
1	-7.098287000	3.572325000	3.723550000
1	-5.217040000	2.006004000	4.145426000
1	-5.491705000	-5.717183000	0.217973000
1	-5.690532000	-7.120150000	-1.836463000
1	-4.659786000	-6.339699000	-3.971155000
1	-3.436228000	-4.174455000	-4.044868000
1	5.802012000	-0.238733000	-1.238111000
1	4.435027000	0.841519000	-1.642938000
1	-3.102546000	0.269655000	4.138227000
1	-4.660840000	-0.128578000	3.375230000
7	-1.896669000	-1.767525000	2.806408000
19	2.079024000	-0.420247000	1.484464000
8	0.794540000	0.328580000	3.964833000
8	0.936809000	-3.113896000	2.069565000
8	3.444218000	1.166309000	3.478076000
8	3.804716000	-2.407337000	2.419131000
6	-0.297543000	-3.725661000	2.432429000
1	-0.310768000	-3.941160000	3.518136000
6	-1.516304000	-2.940429000	1.996009000
1	-0.366350000	-4.701861000	1.918327000
1	-2.366726000	-3.638413000	2.010909000
1	-1.379958000	-2.613577000	0.956266000
6	2.014906000	-4.023647000	2.196958000
1	2.178349000	-4.285696000	3.259220000
1	1.779364000	-4.958358000	1.653323000
6	3.291779000	-3.468226000	1.608217000
1	4.028863000	-4.286557000	1.565660000
1	3.121878000	-3.115030000	0.575718000
6	5.222582000	-2.277124000	2.470523000
1	5.469710000	-1.919646000	3.478721000
1	5.695198000	-3.266258000	2.353352000
6	5.793466000	-1.345535000	1.405056000
1	6.893546000	-1.360580000	1.502776000
1	5.541543000	-1.748992000	0.422966000
6	-1.019568000	-1.239138000	3.846912000
6	-0.570327000	0.201754000	3.598931000
1	-1.483855000	-1.341592000	4.840559000
1	-0.113123000	-1.851823000	3.865260000
1	-1.161562000	0.925746000	4.183007000
1	-0.681734000	0.420360000	2.529042000
6	1.219797000	1.672480000	4.153242000
1	0.618434000	2.153436000	4.945339000
6	2.668977000	1.652872000	4.558013000
1	1.084579000	2.249474000	3.221213000
1	2.990110000	2.673411000	4.838304000
1	2.788300000	1.002330000	5.443095000
6	5.692726000	0.793162000	2.694588000
1	6.675803000	0.428527000	3.033122000
1	5.800425000	1.840092000	2.386237000
6	4.734765000	0.741482000	3.873661000
1	5.144493000	1.416926000	4.646656000
1	4.659075000	-0.262502000	4.324527000
8	2.435764000	-1.831757000	4.870238000
1	1.865678000	-1.083329000	4.620544000
1	2.899668000	-2.069592000	4.047695000
6	-0.335121000	0.409472000	-0.053336000
8	-0.334056000	-0.737978000	0.407353000
8	0.195540000	1.422008000	0.521060000
8	-1.378839000	2.774168000	1.824742000
1	-2.116502000	2.129736000	2.062945000
1	-0.717835000	2.270302000	1.305518000
1	-1.787105000	3.426634000	1.153921000

^{3}IM1 $_{H3O+,K+}$:

6	2.478035000	4.654197000	-2.075214000
6	1.220846000	4.543947000	-2.697958000
6	0.756781000	5.621245000	-3.471509000
6	1.527096000	6.777516000	-3.621296000
6	2.774685000	6.876447000	-2.997165000
6	3.247355000	5.810547000	-2.224132000
6	0.400737000	3.311170000	-2.518214000
6	0.942606000	2.089349000	-2.932171000
7	0.417606000	0.833068000	-2.689966000
6	1.361643000	-0.067059000	-3.136566000
6	2.468893000	0.635633000	-3.752315000
6	2.205522000	1.966777000	-3.639192000
26	-1.324976000	0.377475000	-1.841090000
7	-1.697058000	2.385362000	-1.592639000
6	-2.706837000	2.894970000	-0.815438000
6	-2.501560000	4.305694000	-0.598070000
6	-1.340081000	4.637808000	-1.254904000
6	-0.837594000	3.426653000	-1.854429000
6	-3.778562000	2.151580000	-0.294753000
6	-4.604154000	2.726359000	0.806174000
6	-4.364449000	2.251870000	2.117124000
6	-5.127851000	2.707678000	3.195434000
6	-6.139706000	3.648540000	2.976070000
6	-6.379777000	4.141468000	1.691786000
6	-5.610623000	3.679743000	0.618249000
6	1.291391000	-1.459720000	-3.036873000
6	2.525979000	-2.258120000	-3.289645000
6	2.590937000	-3.139830000	-4.384113000
6	3.711726000	-3.933765000	-4.625663000
6	4.802824000	-3.870465000	-3.754084000
6	4.765945000	-3.004364000	-2.661321000
6	3.645779000	-2.195346000	-2.432854000
8	3.631642000	-1.358870000	-1.328103000
6	4.620841000	-0.332185000	-1.358989000
6	4.628355000	0.514653000	-0.084816000
7	5.153836000	-0.023159000	1.069043000
8	-3.314078000	1.377503000	2.273595000
6	-3.378965000	0.362706000	3.276505000
6	-2.779730000	-0.924086000	2.683625000
8	-3.405729000	-1.488542000	1.791146000
7	-1.112595000	-1.562254000	-2.488699000
6	0.104114000	-2.150170000	-2.737617000
6	-0.015066000	-3.586212000	-2.664546000
6	-1.327992000	-3.858218000	-2.385719000
6	-1.996285000	-2.585661000	-2.256261000
6	-3.331399000	-2.439574000	-1.830690000
6	-4.145434000	-3.676772000	-1.651760000
6	-4.549708000	-4.064489000	-0.360950000
6	-5.288353000	-5.235068000	-0.171866000
6	-5.634344000	-6.034865000	-1.266851000
6	-5.234149000	-5.658556000	-2.553063000
6	-4.492339000	-4.489113000	-2.743185000
7	-3.192380000	-0.025292000	-1.309456000
6	-3.876056000	-1.221032000	-1.422832000

6	-5.228288000	-1.070288000	-0.912797000
6	-5.319073000	0.184746000	-0.400614000
6	-4.047235000	0.832229000	-0.656816000
8	4.241491000	1.676600000	-0.141644000
1	-4.266769000	-3.436663000	0.487397000
1	0.795070000	-4.296826000	-2.802204000
1	-1.792608000	-4.830706000	-2.247015000
1	3.312684000	0.157799000	-4.244110000
1	2.800309000	2.797948000	-4.007094000
1	-0.865145000	5.613573000	-1.308559000
1	-3.160867000	4.959556000	-0.031669000
1	-6.171870000	0.659688000	0.078461000
1	-5.992262000	-1.842399000	-0.932037000
1	1.734034000	-3.188285000	-5.058794000
1	3.732049000	-4.601598000	-5.489462000
1	5.682772000	-4.495847000	-3.920126000
1	5.610638000	-2.958618000	-1.971398000
1	-0.214608000	5.544383000	-3.964840000
1	1.152673000	7.602951000	-4.231656000
1	3.376921000	7.780673000	-3.113353000
1	4.219795000	5.880876000	-1.730489000
1	2.849992000	3.823864000	-1.470048000
1	-5.802189000	4.045320000	-0.392989000
1	-7.167422000	4.878606000	1.522778000
1	-6.734413000	4.002041000	3.821428000
1	-4.926286000	2.346183000	4.204768000
1	-5.591864000	-5.527649000	0.836544000
1	-6.212851000	-6.949859000	-1.118009000
1	-5.502296000	-6.277108000	-3.413135000
1	-4.179737000	-4.196101000	-3.748071000
1	5.625857000	-0.757098000	-1.516669000
1	4.400762000	0.367674000	-2.174563000
1	-2.904449000	0.711443000	4.202944000
1	-4.429809000	0.125328000	3.497122000
7	-1.606501000	-1.416944000	3.167329000
19	2.190718000	-0.534086000	1.839555000
8	1.030513000	0.738384000	4.111317000
8	1.226056000	-2.923249000	2.910298000
8	3.613904000	1.450978000	3.138585000
8	4.118806000	-2.242415000	2.893468000
6	-0.059797000	-3.445788000	3.227488000
1	-0.199439000	-3.490954000	4.323982000
6	-1.172481000	-2.682018000	2.542292000
1	-0.115618000	-4.485621000	2.857597000
1	-2.050808000	-3.342958000	2.507267000
1	-0.875864000	-2.478138000	1.502463000
6	2.279426000	-3.756891000	3.367125000
1	2.455343000	-3.596730000	4.445705000
1	2.006739000	-4.817567000	3.218450000
6	3.551499000	-3.518342000	2.584718000
1	4.269972000	-4.310388000	2.856065000
1	3.347194000	-3.596893000	1.501490000
6	5.476605000	-2.074492000	2.487068000
1	5.979376000	-1.497550000	3.274358000
1	5.975966000	-3.055588000	2.442186000
6	5.641050000	-1.405943000	1.124389000
1	6.716573000	-1.442902000	0.870124000
1	5.097405000	-1.997585000	0.379288000
6	-0.839180000	-0.780445000	4.238031000
6	-0.376326000	0.646371000	3.918477000
1	-1.403382000	-0.805886000	5.183112000
1	0.061616000	-1.379025000	4.405619000
1	-0.873047000	1.387214000	4.568211000
1	-0.607450000	0.889028000	2.872515000
6	1.507188000	2.080524000	4.067759000
1	1.042534000	2.669059000	4.880064000
6	3.002126000	2.074410000	4.255086000
1	1.225547000	2.546474000	3.107925000
1	3.361126000	3.115282000	4.359148000
1	3.254748000	1.529988000	5.181949000
6	5.698280000	0.933249000	2.045085000
1	6.738693000	0.642231000	2.265018000
1	5.715596000	1.911159000	1.549488000
6	4.962541000	1.092066000	3.366266000
1	5.485977000	1.888721000	3.926318000
1	4.997548000	0.183111000	3.990415000
8	2.884660000	-1.125083000	5.284377000
1	2.207032000	-0.523433000	4.927503000
1	3.360809000	-1.467672000	4.508077000
6	-0.259156000	-0.008594000	-0.049881000
8	0.212576000	-1.124089000	0.184284000
8	0.369423000	0.993401000	0.693664000
8	-0.975309000	3.091661000	1.727673000
1	-1.758982000	2.554365000	1.948592000
1	-0.152126000	1.824955000	0.721028000
1	-1.316960000	3.759676000	1.109387000

^{3}RC2 $_{H3O+,K+}$:

6	2.118630000	5.429997000	-1.093948000
6	0.869235000	5.276712000	-1.721996000
6	0.224782000	6.417480000	-2.229894000
6	0.818738000	7.677639000	-2.124466000
6	2.063644000	7.817651000	-1.502655000
6	2.710549000	6.690302000	-0.985434000
6	0.232093000	3.933356000	-1.816047000
6	0.916794000	2.909049000	-2.474970000
7	0.574372000	1.562845000	-2.426863000
6	1.653965000	0.870321000	-2.936025000
6	2.660743000	1.800636000	-3.403613000
6	2.198066000	3.056597000	-3.138953000
26	-1.142154000	0.764066000	-1.781835000
7	-1.764703000	2.595315000	-1.188964000
6	-2.807061000	2.774094000	-0.310366000
6	-2.669946000	4.045864000	0.359688000
6	-1.545975000	4.648070000	-0.176507000
6	-0.988113000	3.730569000	-1.127358000
6	-3.867456000	1.872370000	-0.119604000
6	-4.870756000	2.111189000	0.959803000
6	-4.743563000	1.432206000	2.188583000
6	-5.682593000	1.579842000	3.209087000
6	-6.788002000	2.410149000	3.006283000
6	-6.932107000	3.105549000	1.803101000
6	-5.975663000	2.958132000	0.795407000
6	1.789585000	-0.519814000	-2.941444000
6	3.119853000	-1.111995000	-3.260183000
6	3.367938000	-1.708281000	-4.507091000
6	4.609723000	-2.268831000	-4.808604000
6	5.628563000	-2.249480000	-3.851096000
6	5.399682000	-1.673596000	-2.600704000
6	4.152577000	-1.108630000	-2.307606000
8	3.906198000	-0.585006000	-1.049644000
6	4.621707000	0.605830000	-0.735005000
6	4.622021000	0.929652000	0.759736000
7	5.105587000	0.005632000	1.657147000
8	-3.582929000	0.679662000	2.391339000
6	-3.638658000	-0.608843000	3.052438000
6	-3.037645000	-1.644742000	2.091316000
8	-3.650969000	-1.830514000	1.044603000
7	-0.588450000	-1.013968000	-2.511896000
6	0.720065000	-1.397703000	-2.704682000
6	0.807807000	-2.835038000	-2.764887000
6	-0.475245000	-3.310755000	-2.668005000
6	-1.338845000	-2.166562000	-2.521079000
6	-2.740664000	-2.225811000	-2.386073000

6	-3.406397000	-3.547873000	-2.552265000
6	-4.088629000	-4.132948000	-1.469371000
6	-4.690434000	-5.386232000	-1.606196000
6	-4.622900000	-6.071911000	-2.823887000
6	-3.945933000	-5.498378000	-3.905211000
6	-3.338873000	-4.247203000	-3.768992000
7	-2.975095000	0.071460000	-1.534315000
6	-3.499882000	-1.128943000	-1.977621000
6	-4.927621000	-1.165537000	-1.718131000
6	-5.225050000	-0.059786000	-0.984469000
6	-3.997202000	0.698333000	-0.860884000
8	4.237571000	2.040992000	1.108180000
1	-4.123211000	-3.592816000	-0.521119000
1	1.725146000	-3.404488000	-2.888916000
1	-0.804049000	-4.346416000	-2.682842000
1	3.592945000	1.517683000	-3.886931000
1	2.682624000	4.004042000	-3.358586000
1	-1.140487000	5.625910000	0.069373000
1	-3.388661000	4.477543000	1.054690000
1	-6.184764000	0.239950000	-0.570573000
1	-5.597817000	-1.964371000	-2.023453000
1	2.565575000	-1.719378000	-5.247767000
1	4.782265000	-2.720224000	-5.787828000
1	6.603199000	-2.690042000	-4.072048000
1	6.191470000	-1.667988000	-1.849683000
1	-0.746280000	6.308858000	-2.717848000
1	0.308031000	8.553479000	-2.531769000
1	2.527296000	8.803344000	-1.418466000
1	3.678472000	6.793226000	-0.489068000
1	2.616870000	4.552790000	-0.674764000
1	-6.086313000	3.490279000	-0.151563000
1	-7.790530000	3.762025000	1.647613000
1	-7.527557000	2.523712000	3.801547000
1	-5.545946000	1.064517000	4.160897000
1	-5.210936000	-5.832692000	-0.755227000
1	-5.095080000	-7.051590000	-2.929808000
1	-3.891687000	-6.026545000	-4.860266000
1	-2.811266000	-3.799926000	-4.614240000
1	5.668969000	0.533958000	-1.076906000
1	4.152623000	1.464471000	-1.234615000
1	-3.171581000	-0.517890000	4.037549000
1	-4.691817000	-0.881866000	3.187243000
7	-1.888428000	-2.305445000	2.382750000
19	1.926020000	-0.728163000	1.813138000
8	0.634877000	-0.787788000	4.323862000
8	1.035312000	-3.310241000	1.385767000
8	3.251327000	0.360960000	3.988971000
8	3.874139000	-2.711950000	2.016558000
6	-0.200876000	-4.012444000	1.470435000
1	-0.257718000	-4.575973000	2.420233000
6	-1.401951000	-3.120755000	1.255701000
1	-0.229011000	-4.760097000	0.657548000
1	-2.227314000	-3.773074000	0.936595000
1	-1.200436000	-2.447074000	0.408852000
6	2.129116000	-4.207647000	1.264473000
1	2.264812000	-4.774092000	2.203812000
1	1.922141000	-4.933069000	0.456018000
6	3.409143000	-3.487867000	0.909226000
1	4.164752000	-4.248719000	0.652376000
1	3.261037000	-2.846078000	0.022949000
6	5.277040000	-2.470695000	2.058732000
1	5.543017000	-2.360418000	3.117250000
1	5.820184000	-3.352945000	1.680125000
6	5.722354000	-1.257393000	1.246103000
1	6.820717000	-1.181356000	1.337933000
1	5.488245000	-1.441876000	0.197368000
6	-1.191115000	-2.241186000	3.666623000
6	-0.776923000	-0.840353000	4.142824000
1	-1.795353000	-2.736390000	4.442924000
1	-0.273129000	-2.832451000	3.579339000
1	-1.259940000	-0.597660000	5.105370000
1	-1.051244000	-0.087793000	3.394922000
6	1.054816000	0.426325000	4.936503000
1	0.517341000	0.570965000	5.890845000
6	2.533592000	0.362122000	5.211627000
1	0.816667000	1.283577000	4.279438000
1	2.825956000	1.233632000	5.826085000
1	2.758849000	-0.553426000	5.787640000
6	5.453236000	0.503361000	2.994723000
1	6.501774000	0.229877000	3.198054000
1	5.385176000	1.596686000	2.954088000
6	4.614208000	0.020319000	4.166730000
1	5.017476000	0.518822000	5.067334000
1	4.694641000	-1.066099000	4.337741000
8	2.425733000	-3.017305000	4.488560000
1	1.790977000	-2.279584000	4.475090000
1	2.942708000	-2.928260000	3.668517000
6	-0.303767000	0.569309000	0.209039000
8	-0.673717000	-0.035583000	1.210107000
8	0.868595000	1.305314000	0.430874000
8	-1.733959000	2.343240000	2.550451000
1	-2.443707000	1.556165000	2.472859000
1	1.109435000	1.680380000	-0.432930000
1	-1.853595000	2.959082000	1.762981000
1	-1.903259000	2.857206000	3.362934000

^{3}TS2 $_{H_3O^+,K^+}$:

6	2.023586000	5.519650000	-1.021672000
6	0.797843000	5.306448000	-1.678816000
6	0.143958000	6.411581000	-2.252363000
6	0.704307000	7.688978000	-2.183468000
6	1.927914000	7.886114000	-1.534339000
6	2.584700000	6.797102000	-0.952384000
6	0.192913000	3.947824000	-1.743306000
6	0.917665000	2.904602000	-2.320899000
7	0.575120000	1.565515000	-2.277959000
6	1.671921000	0.860868000	-2.732030000
6	2.700550000	1.787008000	-3.160384000
6	2.230418000	3.044733000	-2.929208000
26	-1.198981000	0.764566000	-1.726798000
7	-1.828376000	2.600160000	-1.168489000
6	-2.912613000	2.797281000	-0.334037000
6	-2.824864000	4.084327000	0.293615000
6	-1.682130000	4.688559000	-0.221935000
6	-1.069980000	3.753538000	-1.107014000
6	-3.962436000	1.879897000	-0.149916000
6	-4.933500000	2.104468000	0.959263000
6	-4.712295000	1.458106000	2.192822000
6	-5.593004000	1.617000000	3.263931000
6	-6.726953000	2.418592000	3.107609000
6	-6.963970000	3.073450000	1.896524000
6	-6.066488000	2.919364000	0.836710000
6	1.794582000	-0.524275000	-2.760641000
6	3.114635000	-1.126797000	-3.097284000
6	3.317110000	-1.755642000	-4.338043000
6	4.546720000	-2.319454000	-4.676938000
6	5.604430000	-2.270703000	-3.763375000
6	5.423617000	-1.663249000	-2.520767000
6	4.186303000	-1.096265000	-2.189588000
8	3.985540000	-0.548115000	-0.933510000
6	4.779939000	0.592293000	-0.618934000
6	4.787058000	0.876010000	0.882831000
7	5.281204000	-0.072955000	1.741009000
8	-3.540609000	0.713120000	2.335507000

6	-3.592731000	-0.562990000	3.014353000
6	-3.035362000	-1.635138000	2.065328000
8	-3.686336000	-1.865949000	1.050399000
7	-0.611347000	-1.000650000	-2.431448000
6	0.701931000	-1.400072000	-2.578525000
6	0.775711000	-2.827255000	-2.661348000
6	-0.518266000	-3.298156000	-2.617502000
6	-1.371284000	-2.153949000	-2.483400000
6	-2.776749000	-2.211754000	-2.383572000
6	-3.432580000	-3.539192000	-2.559022000
6	-4.072065000	-4.155229000	-1.468248000
6	-4.662426000	-5.413273000	-1.613947000
6	-4.624000000	-6.071498000	-2.847843000
6	-3.987945000	-5.466205000	-3.936827000
6	-3.392477000	-4.210307000	-3.791880000
7	-3.038115000	0.100020000	-1.570725000
6	-3.542652000	-1.124207000	-1.984473000
6	-4.967138000	-1.185934000	-1.709721000
6	-5.279352000	-0.084609000	-0.979448000
6	-4.071021000	0.705314000	-0.878292000
8	4.377767000	1.965483000	1.274003000
1	-4.086367000	-3.633674000	-0.509003000
1	1.691222000	-3.403940000	-2.763199000
1	-0.855264000	-4.330208000	-2.654242000
1	3.648701000	1.499223000	-3.608442000
1	2.720765000	3.989840000	-3.145142000
1	-1.305680000	5.683535000	-0.001003000
1	-3.581551000	4.526368000	0.939124000
1	-6.238672000	0.195422000	-0.551111000
1	-5.620654000	-2.003961000	-1.999050000
1	2.486165000	-1.785824000	-5.045713000
1	4.681013000	-2.793510000	-5.651411000
1	6.572008000	-2.711674000	-4.012494000
1	6.248241000	-1.635606000	-1.806919000
1	-0.809726000	6.260032000	-2.762872000
1	0.185233000	8.534058000	-2.642189000
1	2.366021000	8.885475000	-1.479521000
1	3.535714000	6.943436000	-0.434523000
1	2.530838000	4.674008000	-0.551413000
1	-6.244815000	3.423943000	-0.115065000
1	-7.846635000	3.704716000	1.776403000
1	-7.418037000	2.539402000	3.944419000
1	-5.390400000	1.129858000	4.218817000
1	-5.151711000	-5.884382000	-0.757727000
1	-5.086930000	-7.054840000	-2.960654000
1	-3.956025000	-5.973630000	-4.904075000
1	-2.893408000	-3.739169000	-4.641606000
1	5.817456000	0.460438000	-0.968801000
1	4.357200000	1.484380000	-1.100673000
1	-3.099805000	-0.471391000	3.987185000
1	-4.643379000	-0.827557000	3.184012000
7	-1.878931000	-2.289277000	2.344727000
19	2.071104000	-0.588078000	1.651047000
8	0.707671000	-0.662106000	4.161114000
8	0.995206000	-3.299203000	1.278863000
8	3.362079000	0.323036000	3.988553000
8	3.837789000	-2.689167000	1.880494000
6	-0.206024000	-4.037078000	1.488445000
1	-0.178742000	-4.531258000	2.477769000
6	-1.468089000	-3.228823000	1.284645000
1	-0.252271000	-4.845813000	0.736888000
1	-2.283801000	-3.954551000	1.142654000
1	-1.384750000	-2.665245000	0.346002000
6	2.095330000	-4.180191000	1.110521000
1	2.255113000	-4.774167000	2.029474000
1	1.881272000	-4.885359000	0.285304000
6	3.364405000	-3.440797000	0.758126000
1	4.122349000	-4.186655000	0.469519000
1	3.201445000	-2.776696000	-0.107539000
6	5.250577000	-2.567483000	2.018067000
1	5.457205000	-2.527431000	3.095116000
1	5.745887000	-3.471571000	1.627022000
6	5.837903000	-1.352644000	1.302271000
1	6.928496000	-1.358515000	1.474632000
1	5.672813000	-1.470088000	0.231322000
6	-1.111669000	-2.134305000	3.577877000
6	-0.689186000	-0.696376000	3.912369000
1	-1.654888000	-2.578344000	4.427033000
1	-0.188842000	-2.713187000	3.471018000
1	-1.215280000	-0.318420000	4.804463000
1	-0.920165000	-0.039433000	3.063234000
6	1.137874000	0.517379000	4.833701000
1	0.556107000	0.655276000	5.762059000
6	2.595430000	0.371872000	5.178959000
1	0.975292000	1.403819000	4.193981000
1	2.908712000	1.228086000	5.804564000
1	2.741359000	-0.552177000	5.766646000
6	5.609987000	0.363676000	3.102833000
1	6.629334000	0.014464000	3.333748000
1	5.613881000	1.460130000	3.095108000
6	4.693178000	-0.099994000	4.222724000
1	5.079743000	0.351291000	5.154667000
1	4.703175000	-1.193660000	4.364331000
8	2.407436000	-2.970278000	4.360135000
1	1.808813000	-2.203521000	4.352500000
1	2.895502000	-2.913581000	3.519552000
6	-0.435532000	0.503752000	0.051105000
8	-0.470703000	-0.489796000	0.735271000
8	0.271233000	1.579394000	0.688253000
8	-1.558692000	2.392826000	2.304977000
1	-2.278442000	1.664358000	2.344232000
1	0.448359000	2.283301000	0.034527000
1	-0.754516000	2.068143000	1.751408000
1	-1.954013000	3.140376000	1.757551000

^{3}IM2 $_{H3O+,K+}$:

6	1.096660000	6.526459000	-0.773286000
6	0.023866000	6.051293000	-1.545079000
6	-0.824164000	6.978261000	-2.171512000
6	-0.602502000	8.350970000	-2.032026000
6	0.468710000	8.814644000	-1.261841000
6	1.317537000	7.898698000	-0.632018000
6	-0.212236000	4.582366000	-1.685664000
6	0.709629000	3.831735000	-2.427915000
7	0.707003000	2.462499000	-2.583646000
6	1.885391000	2.142661000	-3.223634000
6	2.623382000	3.348679000	-3.528114000
6	1.884833000	4.394195000	-3.056652000
26	-0.629345000	1.160105000	-1.789048000
7	-1.710025000	2.707699000	-1.064696000
6	-2.865751000	2.624344000	-0.316546000
6	-3.199833000	3.918507000	0.226735000
6	-2.246007000	4.791822000	-0.218933000
6	-1.324873000	4.030497000	-1.034630000
6	-3.660912000	1.485494000	-0.166891000
6	-4.899030000	1.550427000	0.668807000
6	-4.828244000	1.294278000	2.052186000
6	-5.987402000	1.263746000	2.832612000
6	-7.231140000	1.487948000	2.236668000
6	-7.319108000	1.760779000	0.869330000
6	-6.154675000	1.793162000	0.097579000
6	2.359357000	0.845103000	-3.460092000
6	3.758481000	0.680549000	-3.953969000
6	4.095022000	1.070573000	-5.262395000

6	5.382812000	0.910503000	-5.771215000
6	6.374289000	0.345669000	-4.961518000
6	6.069134000	-0.043149000	-3.659410000
6	4.772918000	0.118695000	-3.147416000
8	4.593186000	-0.361477000	-1.879313000
6	3.712466000	0.247689000	-0.945025000
6	4.230887000	0.000655000	0.468618000
7	4.431364000	-1.296875000	0.873629000
8	-3.571991000	1.176078000	2.601287000
6	-3.275952000	0.219681000	3.602631000
6	-2.936456000	-1.133868000	2.967282000
8	-3.405829000	-1.393404000	1.868524000
7	0.362576000	-0.368351000	-2.631801000
6	1.624991000	-0.314655000	-3.186149000
6	2.111211000	-1.650059000	-3.441622000
6	1.129937000	-2.510938000	-3.041191000
6	0.029611000	-1.703471000	-2.563545000
6	-1.211734000	-2.217969000	-2.159065000
6	-1.456766000	-3.685172000	-2.249294000
6	-1.791143000	-4.413675000	-1.094197000
6	-2.048273000	-5.784632000	-1.166786000
6	-1.971428000	-6.450590000	-2.394087000
6	-1.638433000	-5.735847000	-3.549248000
6	-1.384634000	-4.364080000	-3.477800000
7	-2.143123000	-0.077108000	-1.334914000
6	-2.247268000	-1.411292000	-1.671534000
6	-3.585147000	-1.888789000	-1.395850000
6	-4.265001000	-0.858223000	-0.823664000
6	-3.358167000	0.261787000	-0.769883000
8	4.442103000	0.961785000	1.199256000
1	-1.832537000	-3.897197000	-0.133607000
1	3.075954000	-1.893448000	-3.877235000
1	1.142724000	-3.596301000	-3.078104000
1	3.582060000	3.386435000	-4.036524000
1	2.118629000	5.453655000	-3.110859000
1	-2.174962000	5.858318000	-0.024862000
1	-4.065488000	4.128197000	0.850426000
1	-5.284274000	-0.848950000	-0.450772000
1	-3.945544000	-2.892000000	-1.602962000
1	3.309061000	1.495324000	-5.890529000
1	5.609393000	1.217528000	-6.794164000
1	7.389398000	0.211521000	-5.342045000
1	6.825665000	-0.479630000	-3.004774000
1	-1.659582000	6.616360000	-2.774926000
1	-1.268110000	9.060711000	-2.528955000
1	0.641436000	9.887822000	-1.152140000
1	2.153956000	8.253548000	-0.025232000
1	1.755416000	5.809709000	-0.277971000
1	-6.211237000	1.991524000	-0.974960000
1	-8.289434000	1.945178000	0.403979000
1	-8.132869000	1.461586000	2.852505000
1	-5.923449000	1.079464000	3.905878000
1	-2.307931000	-6.335960000	-0.259855000
1	-2.172635000	-7.522821000	-2.450532000
1	-1.583603000	-6.246998000	-4.513238000
1	-1.142360000	-3.806254000	-4.384570000
1	3.682040000	1.335626000	-1.080051000
1	2.689987000	-0.145498000	-1.047829000
1	-2.458119000	0.642354000	4.190541000
1	-4.124466000	0.063954000	4.289155000
7	-2.199237000	-2.036987000	3.689879000
19	1.097945000	-1.409531000	1.992291000
8	0.711331000	-1.354769000	4.808104000
8	-0.292646000	-3.817494000	2.045679000
8	3.391719000	-1.225682000	3.601124000
8	2.538018000	-3.694814000	1.260769000
6	-1.081791000	-4.260573000	3.145098000
1	-0.461380000	-4.266793000	4.056739000
6	-2.346702000	-3.446099000	3.305504000
1	-1.407117000	-5.301070000	2.970800000
1	-2.961598000	-3.954433000	4.070349000
1	-2.906401000	-3.459186000	2.364294000
6	0.455583000	-4.878040000	1.463931000
1	0.955657000	-5.467799000	2.250652000
1	-0.217015000	-5.551356000	0.906617000
6	1.499196000	-4.340232000	0.517555000
1	1.917337000	-5.189054000	-0.047375000
1	1.050509000	-3.642088000	-0.212176000
6	3.836648000	-3.735293000	0.670803000
1	4.548758000	-4.010718000	1.460358000
1	3.875043000	-4.537894000	-0.082343000
6	4.250803000	-2.444682000	-0.021745000
1	5.192029000	-2.653518000	-0.560140000
1	3.500627000	-2.197870000	-0.781686000
6	-1.714387000	-1.758568000	5.044657000
6	-0.542971000	-0.797485000	5.192556000
1	-2.532469000	-1.366788000	5.672136000
1	-1.426216000	-2.716728000	5.497479000
1	-0.494043000	-0.520152000	6.260695000
1	-0.699654000	0.131538000	4.621179000
6	1.774872000	-0.488330000	5.212916000
1	1.673320000	-0.264234000	6.289457000
6	3.120678000	-1.124606000	4.988518000
1	1.707364000	0.469374000	4.663455000
1	3.884735000	-0.493725000	5.479764000
1	3.146450000	-2.121626000	5.460241000
6	5.258503000	-1.476680000	2.068601000
1	6.083230000	-2.170079000	1.832190000
1	5.692489000	-0.495907000	2.293900000
6	4.556510000	-1.985813000	3.321228000
1	5.281026000	-1.892382000	4.149244000
1	4.284588000	-3.050692000	3.256939000
8	2.009154000	-4.012501000	4.214588000
1	1.494130000	-3.221687000	4.447605000
1	2.238044000	-3.898769000	3.275984000
6	0.700384000	1.547190000	-0.072692000
8	1.340328000	2.239073000	0.561959000
8	-0.818782000	0.333735000	1.762842000
8	-1.377609000	2.839566000	2.589187000
1	-2.313398000	2.561480000	2.630382000
1	-1.517701000	0.153459000	1.115491000
1	-0.949481000	1.279429000	2.017848000
1	-1.376340000	3.552135000	1.931086000

^{4}CAT $_{H_3O^+,K^+}$:

6	3.763971000	3.571459000	-1.768273000
6	2.558997000	3.584890000	-2.496308000
6	2.355600000	4.608712000	-3.437883000
6	3.323091000	5.596688000	-3.643256000
6	4.513464000	5.576932000	-2.910077000
6	4.729062000	4.560134000	-1.972681000
6	1.521530000	2.543673000	-2.252058000
6	1.889758000	1.184954000	-2.423835000
7	1.143750000	0.097150000	-1.973000000
6	1.936223000	-1.016227000	-2.179843000
6	3.145282000	-0.646141000	-2.830802000
6	3.115583000	0.731233000	-2.981143000
26	-0.820401000	0.092814000	-1.592510000
7	-0.821004000	2.103506000	-1.564150000
6	-1.832608000	2.911444000	-1.074226000
6	-1.352543000	4.271958000	-0.943778000
6	-0.070512000	4.292024000	-1.408066000
6	0.269668000	2.938656000	-1.793581000
6	-3.127672000	2.501513000	-0.776905000

6	-4.102387000	3.428419000	-0.137583000
6	-4.548830000	3.141170000	1.175928000
6	-5.536960000	3.914443000	1.791365000
6	-6.081584000	5.008999000	1.109717000
6	-5.633997000	5.331050000	-0.173576000
6	-4.655187000	4.539979000	-0.786895000
6	1.618907000	-2.334175000	-1.740985000
6	2.711155000	-3.336272000	-1.659205000
6	2.642067000	-4.543818000	-2.383515000
6	3.632315000	-5.521927000	-2.295491000
6	4.736806000	-5.313888000	-1.465258000
6	4.843516000	-4.122461000	-0.745152000
6	3.852288000	-3.140812000	-0.847581000
8	3.958984000	-1.972465000	-0.107542000
6	5.187489000	-1.263660000	-0.277597000
6	5.190740000	0.028043000	0.533369000
7	5.054684000	-0.058202000	1.895303000
8	-3.918025000	2.113226000	1.809199000
6	-4.607039000	1.215333000	2.661388000
6	-4.075459000	-0.193507000	2.373060000
8	-4.870911000	-1.090143000	2.107660000
7	-0.811413000	-1.912969000	-1.570594000
6	0.307151000	-2.718086000	-1.443296000
6	-0.098165000	-4.053055000	-1.050538000
6	-1.463652000	-4.073476000	-1.049434000
6	-1.913532000	-2.742692000	-1.377067000
6	-3.250268000	-2.354501000	-1.402395000
6	-4.291402000	-3.419226000	-1.323679000
6	-5.183999000	-3.484619000	-0.236924000
6	-6.132610000	-4.507884000	-0.160492000
6	-6.212231000	-5.478284000	-1.165556000
6	-5.331404000	-5.421271000	-2.249972000
6	-4.379117000	-4.401125000	-2.326025000
7	-2.788498000	0.083668000	-1.265786000
6	-3.654884000	-1.001917000	-1.385721000
6	-5.005457000	-0.552958000	-1.295786000
6	-4.962392000	0.803745000	-1.041944000
6	-3.583778000	1.178597000	-1.021371000
8	5.335721000	1.095436000	-0.051274000
1	-5.122394000	-2.733942000	0.553994000
1	0.575906000	-4.872570000	-0.818562000
1	-2.114800000	-4.911188000	-0.813999000
1	3.923693000	-1.337080000	-3.145310000
1	3.877381000	1.367367000	-3.423690000
1	0.603268000	5.142930000	-1.460020000
1	-1.935051000	5.102849000	-0.553999000
1	-5.798017000	1.483389000	-0.893693000
1	-5.885172000	-1.184450000	-1.380243000
1	1.784966000	-4.699951000	-3.040798000
1	3.543890000	-6.441601000	-2.878011000
1	5.517533000	-6.072329000	-1.375598000
1	5.700315000	-3.962090000	-0.088903000
1	1.429282000	4.623070000	-4.016310000
1	3.147404000	6.382175000	-4.382541000
1	5.270376000	6.348831000	-3.069340000
1	5.655258000	4.536891000	-1.392610000
1	3.946193000	2.782538000	-1.036495000
1	-4.326345000	4.767723000	-1.803426000
1	-6.055522000	6.187241000	-0.704828000
1	-6.851894000	5.614696000	1.592972000
1	-5.863355000	3.675703000	2.805415000
1	-6.812334000	-4.550662000	0.694511000
1	-6.956559000	-6.275942000	-1.103054000
1	-5.386569000	-6.172388000	-3.041918000
1	-3.692202000	-4.357002000	-3.174075000
1	6.051041000	-1.881774000	0.017493000
1	5.304599000	-0.969560000	-1.327160000
1	-4.477870000	1.499219000	3.720822000
1	-5.681670000	1.192690000	2.433905000
7	-2.721529000	-0.368460000	2.367830000
19	1.763817000	-0.228676000	1.001535000
8	0.246107000	1.715508000	2.280400000
8	0.077972000	-2.132746000	2.408401000
8	3.051615000	2.044957000	2.156569000
8	2.786694000	-1.577037000	3.240861000
6	-1.304871000	-2.468737000	2.514863000
1	-1.616244000	-2.461676000	3.576352000
6	-2.177969000	-1.540277000	1.688952000
1	-1.437793000	-3.497912000	2.134991000
1	-3.035414000	-2.108081000	1.322403000
1	-1.613110000	-1.219211000	0.801793000
6	0.881600000	-3.049353000	3.129566000
1	0.715444000	-2.928297000	4.216804000
1	0.606500000	-4.086700000	2.860065000
6	2.345900000	-2.866555000	2.811842000
1	2.911076000	-3.650463000	3.342056000
1	2.531725000	-2.993153000	1.731580000
6	4.122068000	-1.499268000	3.726200000
1	4.146623000	-0.662488000	4.436448000
1	4.367673000	-2.413586000	4.292086000
6	5.165568000	-1.319503000	2.630886000
1	6.160385000	-1.376295000	3.108554000
1	5.089282000	-2.158577000	1.933983000
6	-1.767510000	0.584654000	2.917301000
6	-1.106533000	1.493880000	1.879683000
1	-2.248614000	1.182440000	3.700909000
1	-0.975061000	0.009419000	3.417180000
1	-1.628877000	2.453897000	1.802686000
1	-1.134476000	1.031088000	0.880741000
6	0.847477000	2.873668000	1.714547000
1	0.189802000	3.749181000	1.851692000
6	2.158517000	3.118315000	2.414985000
1	0.995554000	2.743588000	0.628575000
1	2.593019000	4.072390000	2.063910000
1	1.970193000	3.206710000	3.499251000
6	5.258014000	1.178934000	2.654566000
1	5.924517000	0.963440000	3.504959000
1	5.771199000	1.881612000	1.987573000
6	4.002168000	1.847921000	3.192888000
1	4.316633000	2.818362000	3.616507000
1	3.522579000	1.273993000	4.002847000
8	1.239518000	0.379309000	4.605023000
1	0.909760000	0.918876000	3.863766000
1	1.672319000	-0.377346000	4.170141000

[5]CAT $_{H3O+,K+}$:

6	3.811847000	3.589304000	-1.807054000
6	2.579926000	3.613608000	-2.495583000
6	2.356221000	4.673665000	-3.396892000
6	3.316657000	5.667723000	-3.602875000
6	4.533328000	5.627048000	-2.913084000
6	4.773635000	4.580767000	-2.014184000
6	1.551918000	2.569156000	-2.252057000
6	1.923868000	1.213070000	-2.396744000
7	1.177004000	0.121155000	-1.947684000
6	1.981327000	-1.000853000	-2.141327000
6	3.193977000	-0.622733000	-2.779884000
6	3.155752000	0.752981000	-2.946978000
26	-0.799789000	0.104629000	-1.596372000
7	-0.802306000	2.125817000	-1.554937000
6	-1.821363000	2.930653000	-1.046086000
6	-1.356135000	4.274561000	-0.914013000
6	-0.057759000	4.304307000	-1.388986000
6	0.282412000	2.969719000	-1.782246000

6	-3.123878000	2.495093000	-0.747815000
6	-4.100627000	3.420757000	-0.111135000
6	-4.559917000	3.149715000	1.202062000
6	-5.558741000	3.926477000	1.797994000
6	-6.102054000	5.012570000	1.102615000
6	-5.638623000	5.323824000	-0.178259000
6	-4.650670000	4.529316000	-0.771507000
6	1.664796000	-2.313086000	-1.717856000
6	2.755121000	-3.313946000	-1.638529000
6	2.693015000	-4.520222000	-2.369440000
6	3.684202000	-5.497662000	-2.288524000
6	4.789296000	-5.296683000	-1.455773000
6	4.889914000	-4.110581000	-0.725541000
6	3.896112000	-3.130298000	-0.821051000
8	3.999693000	-1.974519000	-0.058903000
6	5.221157000	-1.256639000	-0.218657000
6	5.218302000	0.026432000	0.605039000
7	5.039400000	-0.070917000	1.961220000
8	-3.934211000	2.140659000	1.874901000
6	-4.656149000	1.197723000	2.644797000
6	-4.118781000	-0.207888000	2.347559000
8	-4.916881000	-1.109309000	2.103858000
7	-0.787411000	-1.911878000	-1.586729000
6	0.340396000	-2.719637000	-1.438424000
6	-0.060936000	-4.040208000	-1.066058000
6	-1.445372000	-4.062028000	-1.075493000
6	-1.888477000	-2.745834000	-1.405258000
6	-3.244537000	-2.349989000	-1.450997000
6	-4.276632000	-3.419007000	-1.389846000
6	-5.202286000	-3.491196000	-0.327612000
6	-6.147583000	-4.519286000	-0.267911000
6	-6.192729000	-5.503357000	-1.261955000
6	-5.276995000	-5.449452000	-2.318950000
6	-4.333423000	-4.420963000	-2.378978000
7	-2.774044000	0.083756000	-1.270527000
6	-3.639664000	-1.002106000	-1.390344000
6	-4.993877000	-0.547878000	-1.280041000
6	-4.948573000	0.799768000	-0.992786000
6	-3.570881000	1.184272000	-0.988224000
8	5.400593000	1.097885000	0.037830000
1	-5.165166000	-2.737451000	0.461877000
1	0.609765000	-4.865067000	-0.840991000
1	-2.095613000	-4.903952000	-0.851361000
1	3.979076000	-1.310805000	-3.086344000
1	3.916552000	1.386632000	-3.395797000
1	0.610721000	5.160165000	-1.433271000
1	-1.940893000	5.103624000	-0.522146000
1	-5.785381000	1.473451000	-0.821157000
1	-5.875999000	-1.175929000	-1.368429000
1	1.835728000	-4.673910000	-3.026854000
1	3.597977000	-6.412057000	-2.880367000
1	5.571751000	-6.054208000	-1.372189000
1	5.744074000	-3.953028000	-0.064647000
1	1.411966000	4.709361000	-3.944787000
1	3.116883000	6.475146000	-4.312652000
1	5.286130000	6.402863000	-3.073816000
1	5.716691000	4.539740000	-1.461868000
1	4.013838000	2.785390000	-1.096652000
1	-4.309961000	4.749457000	-1.785837000
1	-6.056655000	6.173462000	-0.723286000
1	-6.880638000	5.619314000	1.571386000
1	-5.893007000	3.694578000	2.811469000
1	-6.850242000	-4.557891000	0.569295000
1	-6.932529000	-6.306362000	-1.212502000
1	-5.301716000	-6.209833000	-3.104311000
1	-3.622064000	-4.383143000	-3.207259000
1	6.089567000	-1.870359000	0.075027000

1	5.339918000	-0.951679000	-1.265099000
1	-4.584197000	1.434631000	3.721958000
1	-5.718640000	1.182142000	2.365128000
7	-2.766006000	-0.382649000	2.334061000
19	1.736007000	-0.262808000	0.963768000
8	0.218293000	1.684619000	2.260803000
8	0.023068000	-2.137163000	2.409335000
8	3.032819000	2.021971000	2.145332000
8	2.727955000	-1.591398000	3.241771000
6	-1.359645000	-2.487121000	2.479559000
1	-1.692099000	-2.502182000	3.534897000
6	-2.222425000	-1.552255000	1.651068000
1	-1.474319000	-3.509304000	2.076054000
1	-3.077031000	-2.114037000	1.268804000
1	-1.651306000	-1.227660000	0.769131000
6	0.824342000	-3.065035000	3.115685000
1	0.658131000	-2.962979000	4.205182000
1	0.550236000	-4.098230000	2.828886000
6	2.289278000	-2.877080000	2.800538000
1	2.856283000	-3.665760000	3.321751000
1	2.474327000	-2.992382000	1.718705000
6	4.051228000	-1.518545000	3.757951000
1	4.060468000	-0.683668000	4.471016000
1	4.281439000	-2.434902000	4.327335000
6	5.122583000	-1.337771000	2.689988000
1	6.104362000	-1.404337000	3.193360000
1	5.057830000	-2.170791000	1.984737000
6	-1.808084000	0.565603000	2.884700000
6	-1.127633000	1.449348000	1.841059000
1	-2.292744000	1.180448000	3.653018000
1	-1.028495000	-0.013150000	3.400792000
1	-1.649437000	2.405067000	1.727920000
1	-1.136472000	0.964770000	0.852338000
6	0.824454000	2.821557000	1.656946000
1	0.163457000	3.699672000	1.753217000
6	2.128168000	3.096265000	2.358812000
1	0.976764000	2.655318000	0.576677000
1	2.559536000	4.040902000	1.978911000
1	1.930744000	3.220663000	3.437879000
6	5.217573000	1.159942000	2.735005000
1	5.845190000	0.937298000	3.612800000
1	5.762498000	1.863301000	2.094364000
6	3.940748000	1.830764000	3.219614000
1	4.237268000	2.803185000	3.651976000
1	3.430255000	1.259023000	4.012047000
8	1.174703000	0.363802000	4.591500000
1	0.853744000	0.903960000	3.845713000
1	1.604490000	-0.394777000	4.156613000

^{5}**RC1** $_{\text{H3O+,K+}}$:

6	2.531755000	4.638524000	-2.050320000
6	1.292543000	4.486698000	-2.701553000
6	0.817539000	5.553172000	-3.486447000
6	1.558803000	6.728837000	-3.624778000
6	2.791059000	6.864637000	-2.975581000
6	3.274038000	5.814561000	-2.188224000
6	0.506777000	3.230255000	-2.545564000
6	1.120164000	2.030508000	-2.906809000
7	0.632960000	0.755338000	-2.681402000
6	1.593347000	-0.138494000	-3.095357000
6	2.704655000	0.597301000	-3.680717000
6	2.411863000	1.923359000	-3.576389000
26	-1.172021000	0.241114000	-1.833640000
7	-1.681830000	2.295232000	-1.771742000
6	-2.771504000	2.782630000	-1.091569000
6	-2.585460000	4.181060000	-0.829812000
6	-1.344842000	4.528176000	-1.390539000

6	-0.796070000	3.344834000	-1.953732000
6	-3.871080000	1.999620000	-0.626929000
6	-4.798295000	2.616193000	0.367674000
6	-4.601486000	2.347636000	1.739654000
6	-5.459397000	2.851936000	2.718295000
6	-6.547566000	3.641273000	2.333735000
6	-6.757085000	3.937991000	0.984826000
6	-5.883681000	3.430645000	0.017784000
6	1.529387000	-1.531048000	-3.000846000
6	2.791480000	-2.303690000	-3.178612000
6	2.942909000	-3.205814000	-4.247708000
6	4.094824000	-3.976048000	-4.406264000
6	5.131131000	-3.870396000	-3.472952000
6	5.006242000	-2.989105000	-2.398814000
6	3.853201000	-2.207392000	-2.253836000
8	3.746994000	-1.361651000	-1.158173000
6	4.680270000	-0.283850000	-1.167783000
6	4.595548000	0.580650000	0.090904000
7	5.113218000	0.090563000	1.266066000
8	-3.465373000	1.620545000	2.084688000
6	-3.514082000	0.653150000	3.145503000
6	-2.911705000	-0.665966000	2.629614000
8	-3.516455000	-1.255567000	1.741688000
7	-0.901579000	-1.749435000	-2.508665000
6	0.353002000	-2.281246000	-2.699604000
6	0.303896000	-3.703948000	-2.530956000
6	-1.007626000	-4.026236000	-2.226804000
6	-1.741981000	-2.797286000	-2.197258000
6	-3.104600000	-2.674814000	-1.788004000
6	-3.852739000	-3.941899000	-1.544660000
6	-4.315340000	-4.246683000	-0.250448000
6	-5.000026000	-5.438910000	-0.000152000
6	-5.232735000	-6.348689000	-1.037119000
6	-4.773789000	-6.058264000	-2.326668000
6	-4.087005000	-4.867130000	-2.576160000
7	-3.138803000	-0.227402000	-1.409280000
6	-3.737642000	-1.476639000	-1.463893000
6	-5.123622000	-1.354446000	-1.037683000
6	-5.315424000	-0.063972000	-0.652732000
6	-4.063720000	0.640483000	-0.888887000
8	4.145784000	1.719386000	0.006451000
1	-4.123479000	-3.534364000	0.555534000
1	1.146920000	-4.384674000	-2.616552000
1	-1.412816000	-5.012617000	-2.015698000
1	3.573537000	0.142445000	-4.151567000
1	3.004314000	2.761705000	-3.933119000
1	-0.873709000	5.507256000	-1.378512000
1	-3.313557000	4.844652000	-0.366803000
1	-6.224749000	0.393359000	-0.268924000
1	-5.848909000	-2.163890000	-1.028127000
1	2.126849000	-3.290526000	-4.968291000
1	4.183449000	-4.659112000	-5.253982000
1	6.034601000	-4.475678000	-3.575709000
1	5.804968000	-2.910887000	-1.658567000
1	-0.141004000	5.449636000	-3.999522000
1	1.175353000	7.541429000	-4.247030000
1	3.370885000	7.784582000	-3.082831000
1	4.232876000	5.912820000	-1.672774000
1	2.910337000	3.823114000	-1.429405000
1	-6.048796000	3.647105000	-1.039989000
1	-7.602927000	4.560074000	0.684413000
1	-7.222708000	4.034170000	3.097081000
1	-5.274883000	2.645391000	3.773708000
1	-5.349089000	-5.661474000	1.011527000
1	-5.768080000	-7.281063000	-0.841505000
1	-4.954452000	-6.761907000	-3.143254000
1	-3.729532000	-4.642971000	-3.583673000

1	5.712137000	-0.657651000	-1.277869000
1	4.456715000	0.390999000	-2.002823000
1	-3.045428000	1.071863000	4.043695000
1	-4.563466000	0.429122000	3.377705000
7	-1.764037000	-1.164100000	3.170967000
19	2.021061000	-0.304419000	1.862930000
8	0.855354000	0.927332000	4.154799000
8	1.027297000	-2.718763000	2.946539000
8	3.476355000	1.590106000	3.273507000
8	3.931295000	-2.063900000	3.000820000
6	-0.251459000	-3.220103000	3.317174000
1	-0.359019000	-3.227493000	4.418096000
6	-1.384405000	-2.487301000	2.635635000
1	-0.324741000	-4.272593000	2.988243000
1	-2.271800000	-3.136148000	2.696426000
1	-1.143424000	-2.372259000	1.570145000
6	2.073237000	-3.576956000	3.374175000
1	2.242352000	-3.464696000	4.460077000
1	1.792889000	-4.628332000	3.179081000
6	3.353600000	-3.313821000	2.616401000
1	4.058664000	-4.128085000	2.853569000
1	3.163498000	-3.333755000	1.527962000
6	5.324468000	-1.932002000	2.723898000
1	5.762528000	-1.355970000	3.549353000
1	5.803859000	-2.924258000	2.735687000
6	5.621469000	-1.278371000	1.377816000
1	6.717366000	-1.294861000	1.233172000
1	5.164985000	-1.885778000	0.589235000
6	-1.057297000	-0.548563000	4.293920000
6	-0.557599000	0.875424000	4.021315000
1	-1.678758000	-0.571451000	5.202605000
1	-0.174894000	-1.158350000	4.512156000
1	-1.009441000	1.599672000	4.720521000
1	-0.821351000	1.168953000	2.996373000
6	1.359194000	2.260355000	4.142191000
1	0.880345000	2.850185000	4.944002000
6	2.846603000	2.225538000	4.372017000
1	1.123778000	2.743194000	3.176292000
1	3.220882000	3.259804000	4.485945000
1	3.061377000	1.677072000	5.305936000
6	5.596786000	1.080714000	2.238567000
1	6.637514000	0.821716000	2.495299000
1	5.603418000	2.049848000	1.725183000
6	4.823181000	1.245144000	3.536306000
1	5.323439000	2.053745000	4.100137000
1	4.849387000	0.344651000	4.172049000
8	2.658205000	-0.977841000	5.362945000
1	2.000554000	-0.359580000	4.999663000
1	3.138208000	-1.322685000	4.589346000
6	-0.256017000	0.096786000	-0.055118000
8	0.042601000	-1.066548000	0.203448000
8	0.049128000	1.142667000	0.587645000
8	-1.348962000	2.977396000	1.408449000
1	-2.160969000	2.460812000	1.731016000
1	-0.773617000	2.303636000	0.989611000
1	-1.693455000	3.574362000	0.659864000

^{5}IM1 $_{H3O+,K+}$:

6	2.396010000	4.764491000	-1.869257000
6	1.149679000	4.669889000	-2.516709000
6	0.675978000	5.785467000	-3.228358000
6	1.424634000	6.963557000	-3.291576000
6	2.660428000	7.046864000	-2.642061000
6	3.143416000	5.942902000	-1.931475000
6	0.357052000	3.408879000	-2.430807000
6	0.969381000	2.230191000	-2.907904000
7	0.491378000	0.955568000	-2.743041000

6	1.431326000	0.092020000	-3.235710000
6	2.531845000	0.851348000	-3.805569000
6	2.244561000	2.172890000	-3.610403000
26	-1.273649000	0.374504000	-1.755464000
7	-1.770440000	2.415857000	-1.622059000
6	-2.832259000	2.854961000	-0.875083000
6	-2.664669000	4.265027000	-0.592748000
6	-1.479838000	4.648898000	-1.176321000
6	-0.915117000	3.473503000	-1.806947000
6	-3.907878000	2.057777000	-0.422317000
6	-4.767404000	2.593903000	0.676221000
6	-4.506079000	2.137318000	1.990072000
6	-5.291578000	2.560717000	3.065479000
6	-6.350834000	3.446771000	2.839779000
6	-6.615959000	3.918272000	1.552793000
6	-5.822196000	3.491233000	0.482304000
6	1.359708000	-1.314509000	-3.188722000
6	2.611062000	-2.081613000	-3.464404000
6	2.712257000	-2.900102000	-4.603230000
6	3.851875000	-3.661809000	-4.861912000
6	4.921922000	-3.630059000	-3.962774000
6	4.845842000	-2.829513000	-2.822778000
6	3.706827000	-2.053035000	-2.576258000
8	3.644909000	-1.285203000	-1.424735000
6	4.615777000	-0.242860000	-1.370389000
6	4.565356000	0.556928000	-0.065956000
7	5.090965000	-0.000954000	1.080077000
8	-3.414131000	1.314723000	2.148447000
6	-3.393331000	0.342843000	3.193601000
6	-2.743620000	-0.934634000	2.632684000
8	-3.322830000	-1.511209000	1.717427000
7	-1.042334000	-1.551470000	-2.610395000
6	0.201085000	-2.064335000	-2.885145000
6	0.150451000	-3.510991000	-2.808841000
6	-1.136789000	-3.847312000	-2.486916000
6	-1.875236000	-2.607265000	-2.345418000
6	-3.218993000	-2.526395000	-1.900321000
6	-3.949365000	-3.810391000	-1.688152000
6	-4.344054000	-4.180884000	-0.388952000
6	-5.010930000	-5.388433000	-0.168009000
6	-5.294976000	-6.243274000	-1.238802000
6	-4.905095000	-5.884249000	-2.533172000
6	-4.235104000	-4.677865000	-2.755175000
7	-3.274126000	-0.112093000	-1.416236000
6	-3.868965000	-1.342187000	-1.506687000
6	-5.231966000	-1.254905000	-0.998031000
6	-5.396175000	0.012299000	-0.523356000
6	-4.155460000	0.723521000	-0.789280000
8	4.147572000	1.708895000	-0.091353000
1	-4.109850000	-3.510538000	0.441543000
1	0.987798000	-4.184757000	-2.969402000
1	-1.540331000	-4.844500000	-2.332477000
1	3.390527000	0.425282000	-4.319797000
1	2.833535000	3.031113000	-3.922921000
1	-1.030857000	5.638386000	-1.160494000
1	-3.360291000	4.885586000	-0.032144000
1	-6.278808000	0.444074000	-0.056821000
1	-5.956410000	-2.065067000	-0.992377000
1	1.869434000	-2.926111000	-5.296952000
1	3.902863000	-4.281157000	-5.759907000
1	5.815669000	-4.231427000	-4.143373000
1	5.672308000	-2.811418000	-2.109802000
1	-0.284751000	5.721045000	-3.743707000
1	1.042869000	7.818575000	-3.854849000
1	3.245528000	7.968336000	-2.690495000
1	4.106792000	6.000262000	-1.418753000
1	2.775795000	3.904771000	-1.311910000
1	-6.029606000	3.842427000	-0.530937000
1	-7.440555000	4.612652000	1.379065000
1	-6.962875000	3.774448000	3.683161000
1	-5.072986000	2.217832000	4.077636000
1	-5.306610000	-5.666309000	0.846866000
1	-5.817451000	-7.187124000	-1.065063000
1	-5.125936000	-6.545142000	-3.375007000
1	-3.932871000	-4.397899000	-3.766879000
1	5.633583000	-0.645245000	-1.504574000
1	4.416716000	0.486269000	-2.166092000
1	-2.915328000	0.759743000	4.089122000
1	-4.422514000	0.057575000	3.458115000
7	-1.589674000	-1.417695000	3.172549000
19	2.158915000	-0.557014000	1.944473000
8	0.989238000	0.689722000	4.204109000
8	1.242042000	-2.931049000	2.987393000
8	3.572805000	1.421866000	3.208187000
8	4.125606000	-2.248006000	2.915464000
6	-0.043857000	-3.452874000	3.299984000
1	-0.193482000	-3.486768000	4.395403000
6	-1.151361000	-2.703361000	2.591467000
1	-0.095263000	-4.497115000	2.942024000
1	-2.029198000	-3.366562000	2.567375000
1	-0.849914000	-2.527729000	1.548412000
6	2.299240000	-3.763229000	3.439173000
1	2.487496000	-3.593749000	4.513944000
1	2.023544000	-4.824569000	3.302177000
6	3.561325000	-3.532414000	2.637627000
1	4.287029000	-4.316115000	2.914382000
1	3.343469000	-3.631065000	1.558809000
6	5.467427000	-2.070301000	2.461416000
1	5.997781000	-1.503185000	3.237905000
1	5.967128000	-3.049128000	2.384854000
6	5.586727000	-1.382000000	1.103012000
1	6.655791000	-1.410839000	0.820493000
1	5.027024000	-1.964903000	0.361974000
6	-0.910815000	-0.807423000	4.316781000
6	-0.428429000	0.630860000	4.088304000
1	-1.551088000	-0.858393000	5.211041000
1	-0.025726000	-1.409989000	4.544565000
1	-0.871501000	1.315490000	4.832871000
1	-0.711950000	0.976706000	3.085207000
6	1.475724000	2.029228000	4.175342000
1	1.024017000	2.607871000	5.001797000
6	2.973235000	2.021120000	4.344143000
1	1.184778000	2.510487000	3.225183000
1	3.329824000	3.061225000	4.463112000
1	3.238422000	1.459880000	5.257019000
6	5.640433000	0.942339000	2.067296000
1	6.687963000	0.659343000	2.263572000
1	5.638669000	1.929702000	1.590524000
6	4.928365000	1.070479000	3.404649000
1	5.455320000	1.861887000	3.968804000
1	4.983899000	0.151294000	4.011889000
8	2.908269000	-1.153338000	5.344046000
1	2.202587000	-0.578011000	4.998783000
1	3.383138000	-1.481082000	4.560745000
6	-0.226924000	-0.020863000	0.016523000
8	0.249982000	-1.143414000	0.216118000
8	0.331199000	0.949818000	0.842702000
8	-1.114168000	3.014471000	1.710073000
1	-1.915711000	2.478446000	1.867166000
1	-0.202100000	1.778236000	0.868854000
1	-1.408318000	3.695960000	1.081768000

^{5}RC2 $_{H3O+,K+}$:

6	2.238327000	5.236764000	-1.185494000

6	1.011810000	5.132687000	-1.867794000
6	0.442264000	6.298137000	-2.409741000
6	1.083551000	7.532471000	-2.280850000
6	2.303367000	7.623101000	-1.602869000
6	2.877613000	6.471437000	-1.054479000
6	0.320993000	3.817549000	-1.990126000
6	1.032554000	2.757281000	-2.593285000
7	0.644113000	1.438154000	-2.581469000
6	1.688294000	0.687673000	-3.051455000
6	2.761507000	1.574877000	-3.468469000
6	2.354531000	2.851362000	-3.195847000
26	-1.124046000	0.635432000	-1.807656000
7	-1.787857000	2.606181000	-1.459880000
6	-2.861886000	2.823607000	-0.635927000
6	-2.722654000	4.128864000	-0.013183000
6	-1.550718000	4.674296000	-0.498425000
6	-0.966072000	3.701743000	-1.392877000
6	-3.913374000	1.912578000	-0.383469000
6	-4.912339000	2.252047000	0.676563000
6	-4.787120000	1.675497000	1.956737000
6	-5.713407000	1.923200000	2.968434000
6	-6.807946000	2.751099000	2.703506000
6	-6.952097000	3.344190000	1.447183000
6	-6.005915000	3.098130000	0.448903000
6	1.741924000	-0.721712000	-3.079403000
6	3.062006000	-1.365202000	-3.353644000
6	3.308696000	-2.003090000	-4.580827000
6	4.517615000	-2.651289000	-4.835159000
6	5.505543000	-2.684517000	-3.846331000
6	5.280369000	-2.063440000	-2.617478000
6	4.069997000	-1.403051000	-2.373343000
8	3.852298000	-0.811200000	-1.137842000
6	4.661130000	0.339782000	-0.899038000
6	4.659824000	0.805247000	0.556721000
7	5.119543000	-0.036691000	1.541314000
8	-3.632549000	0.920461000	2.207229000
6	-3.682345000	-0.248668000	3.064982000
6	-3.070408000	-1.431934000	2.298668000
8	-3.667036000	-1.801994000	1.291881000
7	-0.645605000	-1.223934000	-2.641376000
6	0.655063000	-1.597802000	-2.867214000
6	0.734872000	-3.046762000	-2.902279000
6	-0.537203000	-3.518290000	-2.719746000
6	-1.399676000	-2.363038000	-2.552307000
6	-2.787995000	-2.416401000	-2.273058000
6	-3.440112000	-3.756967000	-2.280293000
6	-4.011167000	-4.264252000	-1.097935000
6	-4.601480000	-5.530296000	-1.084207000
6	-4.633867000	-6.306734000	-2.247709000
6	-4.069672000	-5.810114000	-3.427334000
6	-3.474633000	-4.545896000	-3.442517000
7	-3.063595000	-0.068996000	-1.579667000
6	-3.563002000	-1.311648000	-1.869059000
6	-4.978157000	-1.347347000	-1.528199000
6	-5.272670000	-0.157934000	-0.930659000
6	-4.056044000	0.636275000	-0.958650000
8	4.316396000	1.959114000	0.793088000
1	-3.973110000	-3.653797000	-0.193090000
1	1.640895000	-3.627997000	-3.051553000
1	-0.857043000	-4.556175000	-2.680360000
1	3.695093000	1.259191000	-3.928720000
1	2.898506000	3.772796000	-3.385549000
1	-1.127086000	5.643458000	-0.248323000
1	-3.450492000	4.603966000	0.642863000
1	-6.225919000	0.167768000	-0.520883000
1	-5.646103000	-2.187898000	-1.695858000
1	2.527269000	-1.981531000	-5.343316000
1	4.686495000	-3.133477000	-5.800242000
1	6.452156000	-3.198534000	-4.026721000
1	6.046237000	-2.094180000	-1.840459000
1	-0.507610000	6.228791000	-2.944211000
1	0.629832000	8.426686000	-2.714784000
1	2.804201000	8.588628000	-1.500316000
1	3.825991000	6.535068000	-0.515637000
1	2.684317000	4.343024000	-0.743216000
1	-6.115979000	3.550697000	-0.538604000
1	-7.802231000	3.997937000	1.242698000
1	-7.538583000	2.942545000	3.491960000
1	-5.578566000	1.490196000	3.960311000
1	-5.034842000	-5.915130000	-0.157621000
1	-5.096867000	-7.296384000	-2.235477000
1	-4.095102000	-6.408505000	-4.341291000
1	-3.037214000	-4.158478000	-4.365290000
1	5.706093000	0.148116000	-1.198559000
1	4.279874000	1.186266000	-1.484597000
1	-3.223653000	-0.000302000	4.027750000
1	-4.734508000	-0.505891000	3.233327000
7	-1.941373000	-2.041097000	2.738990000
19	2.008161000	-0.698508000	1.747979000
8	0.649705000	-0.140408000	4.228277000
8	0.971330000	-3.308675000	2.035786000
8	3.312507000	0.760765000	3.719170000
8	3.862897000	-2.644304000	2.429372000
6	-0.284342000	-3.939548000	2.272220000
1	-0.363656000	-4.252159000	3.330231000
6	-1.464185000	-3.100832000	1.833030000
1	-0.324630000	-4.864269000	1.668708000
1	-2.302837000	-3.792235000	1.664923000
1	-1.236066000	-2.636246000	0.862582000
6	2.042622000	-4.228059000	2.185798000
1	2.181057000	-4.485230000	3.251551000
1	1.807499000	-5.160542000	1.640058000
6	3.335213000	-3.692733000	1.612994000
1	4.057115000	-4.525655000	1.578597000
1	3.179432000	-3.338587000	0.577328000
6	5.266780000	-2.419676000	2.312714000
1	5.623892000	-2.126292000	3.308336000
1	5.775193000	-3.362703000	2.053736000
6	5.642765000	-1.377093000	1.263257000
1	6.746280000	-1.347062000	1.207977000
1	5.264353000	-1.709140000	0.293327000
6	-1.208730000	-1.662045000	3.946199000
6	-0.754384000	-0.197157000	4.007254000
1	-1.798251000	-1.917576000	4.840409000
1	-0.304121000	-2.276686000	3.993721000
1	-1.258433000	0.340927000	4.829103000
1	-0.975828000	0.296369000	3.052442000
6	1.105431000	1.175832000	4.519425000
1	0.541916000	1.588851000	5.375289000
6	2.569163000	1.122870000	4.869185000
1	0.936680000	1.835404000	3.648025000
1	2.892468000	2.112844000	5.240935000
1	2.724825000	0.385282000	5.676522000
6	5.534581000	0.594179000	2.801330000
1	6.546947000	0.235049000	3.048212000
1	5.590141000	1.672447000	2.611697000
6	4.641798000	0.372816000	4.012330000
1	5.053896000	0.992123000	4.829491000
1	4.643103000	-0.671930000	4.365761000
8	2.426812000	-2.291158000	4.922525000
1	1.796926000	-1.577669000	4.717920000
1	2.952764000	-2.409530000	4.112289000
6	-0.292107000	0.465393000	0.113767000
8	-0.505234000	-0.390683000	0.967200000

8	0.722427000	1.360184000	0.496037000
8	-1.752333000	2.538629000	2.224624000
1	-2.521576000	1.795410000	2.229834000
1	0.909818000	1.931866000	-0.265238000
1	-1.883463000	3.147590000	1.434402000
1	-1.798699000	3.077025000	3.037747000

^{5}TS2 $_{H_3O^+,K^+}$:

6	1.980654000	5.529668000	-0.882905000
6	0.792256000	5.353502000	-1.614426000
6	0.186185000	6.481011000	-2.195137000
6	0.756593000	7.748684000	-2.056493000
6	1.940595000	7.910814000	-1.330170000
6	2.549775000	6.797294000	-0.742310000
6	0.172491000	4.003986000	-1.753262000
6	0.948309000	2.978292000	-2.342471000
7	0.603763000	1.651182000	-2.383642000
6	1.691369000	0.942839000	-2.823144000
6	2.755749000	1.873713000	-3.159630000
6	2.295033000	3.129331000	-2.874881000
26	-1.160606000	0.767724000	-1.724065000
7	-1.884020000	2.690199000	-1.275225000
6	-2.971794000	2.841876000	-0.453033000
6	-2.910031000	4.148112000	0.178295000
6	-1.771583000	4.762532000	-0.295079000
6	-1.120941000	3.826161000	-1.188926000
6	-3.979377000	1.878968000	-0.234331000
6	-4.957591000	2.119352000	0.869772000
6	-4.743181000	1.507392000	2.123318000
6	-5.640299000	1.689948000	3.178165000
6	-6.781670000	2.472960000	2.987455000
6	-7.011633000	3.091470000	1.756309000
6	-6.098932000	2.917142000	0.713005000
6	1.785367000	-0.463053000	-2.900342000
6	3.120030000	-1.050047000	-3.223816000
6	3.352759000	-1.618100000	-4.487952000
6	4.585112000	-2.180650000	-4.819172000
6	5.614353000	-2.192027000	-3.873233000
6	5.402966000	-1.643116000	-2.608436000
6	4.164268000	-1.074932000	-2.283790000
8	3.945860000	-0.578158000	-1.007555000
6	4.742715000	0.550762000	-0.651607000
6	4.787409000	0.794816000	0.857263000
7	5.263772000	-0.186518000	1.688820000
8	-3.564676000	0.786893000	2.308259000
6	-3.599378000	-0.455480000	3.047760000
6	-3.064634000	-1.566526000	2.132083000
8	-3.725426000	-1.810723000	1.126445000
7	-0.597711000	-1.048282000	-2.559734000
6	0.721364000	-1.379173000	-2.744941000
6	0.840355000	-2.822312000	-2.826086000
6	-0.427075000	-3.332202000	-2.720636000
6	-1.326964000	-2.206341000	-2.555219000
6	-2.728432000	-2.297486000	-2.358463000
6	-3.351235000	-3.645827000	-2.479560000
6	-3.990805000	-4.228813000	-1.369488000
6	-4.551721000	-5.504626000	-1.465127000
6	-4.486165000	-6.215994000	-2.668061000
6	-3.853571000	-5.644183000	-3.776827000
6	-3.287738000	-4.370236000	-3.682077000
7	-3.072447000	0.000198000	-1.547026000
6	-3.541352000	-1.228609000	-1.934210000
6	-4.964660000	-1.306152000	-1.638652000
6	-5.290346000	-0.171847000	-0.954813000
6	-4.088242000	0.639504000	-0.894988000
8	4.435245000	1.894128000	1.274634000
1	-4.027236000	-3.669334000	-0.432658000
1	1.768044000	-3.374889000	-2.950365000
1	-0.720362000	-4.378581000	-2.731044000
1	3.721767000	1.601001000	-3.578167000
1	2.818216000	4.071672000	-3.012706000
1	-1.405193000	5.753449000	-0.039709000
1	-3.664874000	4.571498000	0.839026000
1	-6.255671000	0.107224000	-0.538808000
1	-5.616087000	-2.139498000	-1.887448000
1	2.542277000	-1.604420000	-5.219526000
1	4.741995000	-2.608361000	-5.811493000
1	6.583448000	-2.634139000	-4.114178000
1	6.205745000	-1.661629000	-1.869878000
1	-0.736351000	6.355977000	-2.766226000
1	0.275509000	8.612964000	-2.520588000
1	2.386130000	8.902300000	-1.220452000
1	3.469947000	6.916400000	-0.165384000
1	2.452682000	4.664976000	-0.411210000
1	-6.271093000	3.393043000	-0.254637000
1	-7.899054000	3.709862000	1.607697000
1	-7.482763000	2.609458000	3.813507000
1	-5.442898000	1.236283000	4.150458000
1	-5.038620000	-5.948202000	-0.593034000
1	-4.926049000	-7.213498000	-2.741366000
1	-3.802342000	-6.191381000	-4.721248000
1	-2.796289000	-3.924402000	-4.549553000
1	5.775077000	0.433975000	-1.021736000
1	4.313493000	1.459898000	-1.093344000
1	-3.079272000	-0.315163000	4.000392000
1	-4.644245000	-0.712960000	3.259551000
7	-1.915612000	-2.233294000	2.413096000
19	2.054315000	-0.672662000	1.642819000
8	0.709476000	-0.655976000	4.198247000
8	0.943361000	-3.316677000	1.320675000
8	3.385935000	0.254403000	3.935152000
8	3.807408000	-2.790746000	1.872321000
6	-0.281712000	-4.014046000	1.535087000
1	-0.265751000	-4.512704000	2.522293000
6	-1.516051000	-3.162679000	1.340091000
1	-0.358434000	-4.816992000	0.779995000
1	-2.349951000	-3.860502000	1.172683000
1	-1.406917000	-2.582926000	0.412609000
6	2.011945000	-4.234150000	1.142481000
1	2.167036000	-4.825334000	2.063975000
1	1.762227000	-4.938664000	0.327016000
6	3.299686000	-3.540717000	0.765032000
1	4.029094000	-4.316574000	0.481047000
1	3.150503000	-2.884478000	-0.110466000
6	5.224615000	-2.675445000	1.962051000
1	5.468499000	-2.627675000	3.030906000
1	5.702565000	-3.584522000	1.561405000
6	5.785380000	-1.468525000	1.215256000
1	6.884280000	-1.485223000	1.323339000
1	5.553441000	-1.582201000	0.155979000
6	-1.142497000	-2.100570000	3.645971000
6	-0.699707000	-0.677271000	4.015583000
1	-1.688994000	-2.555628000	4.487372000
1	-0.226680000	-2.688738000	3.526957000
1	-1.181094000	-0.340079000	4.949391000
1	-0.959583000	0.020684000	3.210315000
6	1.174962000	0.555517000	4.781901000
1	0.606613000	0.774164000	5.703274000
6	2.632131000	0.400967000	5.125444000
1	1.025082000	1.398356000	4.082055000
1	2.970906000	1.289621000	5.689459000
1	2.760322000	-0.484455000	5.773699000
6	5.632246000	0.215248000	3.051681000
1	6.634301000	-0.189580000	3.266289000

1	5.693736000	1.310021000	3.057056000
6	4.701095000	-0.213411000	4.174675000
1	5.102398000	0.228529000	5.104463000
1	4.671610000	-1.306100000	4.321130000
8	2.387532000	-2.991941000	4.380389000
1	1.810039000	-2.208842000	4.377997000
1	2.866202000	-2.957299000	3.533453000
6	-0.423548000	0.543518000	0.191466000
8	-0.481329000	-0.368536000	0.975375000
8	0.275938000	1.728096000	0.861387000
8	-1.570936000	2.501873000	2.320502000
1	-2.286033000	1.779964000	2.326576000
1	0.458031000	2.404871000	0.183739000
1	-0.715833000	2.181633000	1.748785000
1	-1.949850000	3.263963000	1.814572000

^{5}IM2 $_{H3O+,K+}$:

6	1.049045000	6.503914000	-0.643644000
6	0.024516000	6.054500000	-1.492427000
6	-0.764140000	7.001641000	-2.164041000
6	-0.531488000	8.369236000	-1.993487000
6	0.492011000	8.807270000	-1.147295000
6	1.281645000	7.870823000	-0.472108000
6	-0.225611000	4.589510000	-1.665211000
6	0.733757000	3.850928000	-2.390671000
7	0.721573000	2.491655000	-2.616311000
6	1.911959000	2.157990000	-3.219496000
6	2.686536000	3.363261000	-3.432344000
6	1.952178000	4.407629000	-2.939662000
26	-0.544188000	1.126025000	-1.648250000
7	-1.762720000	2.744134000	-1.066259000
6	-2.929778000	2.640563000	-0.354198000
6	-3.300188000	3.948197000	0.153377000
6	-2.341164000	4.827124000	-0.273971000
6	-1.379024000	4.059687000	-1.048968000
6	-3.682194000	1.465982000	-0.182133000
6	-4.922352000	1.519385000	0.653551000
6	-4.840237000	1.284031000	2.040430000
6	-5.994321000	1.239089000	2.826960000
6	-7.245260000	1.429676000	2.233791000
6	-7.345044000	1.682918000	0.863646000
6	-6.185067000	1.728795000	0.085485000
6	2.361782000	0.849272000	-3.505266000
6	3.766244000	0.695070000	-3.990044000
6	4.128088000	1.140685000	-5.273432000
6	5.421438000	0.984317000	-5.770150000
6	6.392440000	0.367936000	-4.973483000
6	6.063125000	-0.073332000	-3.693717000
6	4.762202000	0.085489000	-3.194434000
8	4.552561000	-0.438336000	-1.947668000
6	3.691254000	0.183812000	-1.002545000
6	4.226820000	-0.051255000	0.406845000
7	4.417549000	-1.344218000	0.827819000
8	-3.577611000	1.200893000	2.584849000
6	-3.257362000	0.273795000	3.605420000
6	-2.910969000	-1.091275000	2.997853000
8	-3.358351000	-1.365257000	1.893117000
7	0.399881000	-0.418801000	-2.694301000
6	1.636497000	-0.336965000	-3.278357000
6	2.110847000	-1.673307000	-3.587945000
6	1.140965000	-2.544609000	-3.178465000
6	0.053098000	-1.746126000	-2.637241000
6	-1.177752000	-2.253290000	-2.168373000
6	-1.425790000	-3.720275000	-2.266785000
6	-1.745070000	-4.457241000	-1.112101000
6	-2.003054000	-5.827647000	-1.189585000
6	-1.944222000	-6.485961000	-2.422014000
6	-1.630046000	-5.763047000	-3.577325000
6	-1.375075000	-4.391852000	-3.500770000
7	-2.139490000	-0.145523000	-1.262645000
6	-2.220993000	-1.470578000	-1.623764000
6	-3.560860000	-1.952294000	-1.357527000
6	-4.255191000	-0.920173000	-0.794002000
6	-3.358231000	0.209631000	-0.731833000
8	4.460488000	0.918244000	1.119831000
1	-1.777589000	-3.946464000	-0.147966000
1	3.059245000	-1.912175000	-4.061689000
1	1.158065000	-3.628718000	-3.244048000
1	3.668408000	3.408454000	-3.894830000
1	2.221949000	5.460094000	-2.933558000
1	-2.295507000	5.897986000	-0.095030000
1	-4.184408000	4.162722000	0.749431000
1	-5.283708000	-0.920562000	-0.445711000
1	-3.924430000	-2.952615000	-1.573791000
1	3.357896000	1.605760000	-5.892552000
1	5.668124000	1.334452000	-6.774470000
1	7.410926000	0.235764000	-5.345639000
1	6.804314000	-0.547672000	-3.047884000
1	-1.562216000	6.659828000	-2.826903000
1	-1.151002000	9.094903000	-2.525828000
1	0.673766000	9.876254000	-1.013743000
1	2.080597000	8.205550000	0.193785000
1	1.661462000	5.771057000	-0.113451000
1	-6.250445000	1.911418000	-0.989344000
1	-8.321160000	1.841152000	0.400750000
1	-8.143488000	1.392448000	2.854139000
1	-5.922256000	1.070155000	3.902235000
1	-2.250549000	-6.384785000	-0.282781000
1	-2.146306000	-7.557805000	-2.482458000
1	-1.591743000	-6.267274000	-4.545774000
1	-1.150227000	-3.828311000	-4.408343000
1	3.670091000	1.270683000	-1.144197000
1	2.663876000	-0.200854000	-1.092419000
1	-2.434678000	0.719385000	4.170092000
1	-4.092548000	0.126676000	4.309846000
7	-2.186967000	-1.984485000	3.743231000
19	1.065890000	-1.397924000	1.970758000
8	0.724605000	-1.290139000	4.802731000
8	-0.318718000	-3.806488000	2.101700000
8	3.387688000	-1.220628000	3.554190000
8	2.497663000	-3.705297000	1.266930000
6	-1.090726000	-4.225402000	3.222521000
1	-0.454771000	-4.218304000	4.123171000
6	-2.347757000	-3.400826000	3.388975000
1	-1.424850000	-5.266945000	3.072747000
1	-2.951728000	-3.888352000	4.175325000
1	-2.922910000	-3.429695000	2.457552000
6	0.417411000	-4.880629000	1.529559000
1	0.929767000	-5.454998000	2.319732000
1	-0.266014000	-5.564589000	0.998863000
6	1.445815000	-4.364449000	0.554922000
1	1.852531000	-5.225731000	0.000821000
1	0.986523000	-3.680611000	-0.181617000
6	3.789770000	-3.775642000	0.665312000
1	4.505940000	-4.050260000	1.451655000
1	3.808470000	-4.589513000	-0.076273000
6	4.216552000	-2.501681000	-0.049659000
1	5.152244000	-2.731058000	-0.589630000
1	3.466047000	-2.255864000	-0.809739000
6	-1.696844000	-1.679654000	5.090351000
6	-0.518430000	-0.723702000	5.208332000
1	-2.509364000	-1.267198000	5.711818000
1	-1.413778000	-2.629956000	5.562479000
1	-0.447951000	-0.437455000	6.272943000

1	-0.682292000	0.202349000	4.633424000
6	1.799460000	-0.418002000	5.164393000
1	1.715805000	-0.160628000	6.234944000
6	3.137921000	-1.069802000	4.940925000
1	1.728645000	0.522940000	4.587113000
1	3.912752000	-0.427262000	5.398999000
1	3.164660000	-2.050038000	5.446616000
6	5.248513000	-1.516443000	2.020719000
1	6.062856000	-2.224316000	1.791489000
1	5.696190000	-0.538003000	2.228629000
6	4.544468000	-1.996954000	3.283492000
1	5.271998000	-1.897546000	4.107956000
1	4.261887000	-3.059816000	3.237388000
8	2.000509000	-3.963771000	4.245958000
1	1.495941000	-3.164447000	4.472976000
1	2.217839000	-3.865744000	3.303016000
6	0.781961000	1.596566000	-0.048484000
8	1.443968000	2.216638000	0.634992000
8	-0.816131000	0.400729000	1.664442000
8	-1.412368000	2.894228000	2.501923000
1	-2.345815000	2.612841000	2.565450000
1	-1.598948000	0.148098000	1.149711000
1	-0.979384000	1.337024000	1.939403000
1	-1.428705000	3.604002000	1.840576000

^{3}RC1$_{phenol,K+}$:

6	3.941987000	3.130028000	-2.690584000
6	2.668277000	3.408843000	-3.221037000
6	2.529998000	4.515474000	-4.076843000
6	3.628530000	5.320451000	-4.394722000
6	4.887999000	5.032747000	-3.860132000
6	5.038917000	3.934535000	-3.004776000
6	1.487855000	2.576173000	-2.851627000
6	1.535909000	1.182045000	-3.116351000
7	0.608694000	0.259468000	-2.658212000
6	1.143969000	-0.985651000	-2.899955000
6	2.391712000	-0.858022000	-3.585692000
6	2.624642000	0.498760000	-3.742552000
26	-1.181689000	0.654280000	-1.853258000
7	-0.747487000	2.608519000	-1.774173000
6	-1.354343000	3.483200000	-0.888280000
6	-0.501700000	4.639642000	-0.688445000
6	0.582160000	4.481244000	-1.500777000
6	0.458091000	3.185075000	-2.138536000
6	-2.615355000	3.304332000	-0.326699000
6	-3.114257000	4.183581000	0.761872000
6	-3.401911000	3.618966000	2.036465000
6	-3.875087000	4.418895000	3.082639000
6	-4.072872000	5.790960000	2.883246000
6	-3.796961000	6.366024000	1.642719000
6	-3.324729000	5.560540000	0.599869000
6	0.528507000	-2.218637000	-2.545057000
6	1.330781000	-3.466754000	-2.637673000
6	0.983774000	-4.465961000	-3.569512000
6	1.706144000	-5.652360000	-3.693855000
6	2.818665000	-5.870931000	-2.877000000
6	3.190546000	-4.899681000	-1.946377000
6	2.459039000	-3.710561000	-1.826303000
8	2.818999000	-2.787384000	-0.856195000
6	4.128122000	-2.238500000	-0.972351000
6	4.791601000	-1.816147000	0.335237000
7	4.467769000	-2.431920000	1.529418000
8	-3.155330000	2.286281000	2.184734000
6	-3.773264000	1.547101000	3.231653000
6	-3.930102000	0.124648000	2.682782000
8	-4.956490000	-0.134170000	2.054768000
7	-1.716891000	-1.247570000	-2.228987000
6	-0.830720000	-2.308480000	-2.247487000
6	-1.541882000	-3.543154000	-1.974296000
6	-2.863813000	-3.223375000	-1.858041000
6	-2.972148000	-1.786728000	-1.997414000
6	-4.156917000	-1.078540000	-1.797499000
6	-5.428565000	-1.851930000	-1.713907000
6	-6.182142000	-1.877468000	-0.524294000
6	-7.353133000	-2.635469000	-0.442983000
6	-7.796650000	-3.378155000	-1.543886000
6	-7.055958000	-3.360065000	-2.729506000
6	-5.882365000	-2.604328000	-2.811427000
7	-3.047808000	1.087316000	-1.341447000
6	-4.184108000	0.309631000	-1.525886000
6	-5.344383000	1.060762000	-1.157891000
6	-4.896123000	2.265939000	-0.648691000
6	-3.473169000	2.254152000	-0.760247000
8	5.607370000	-0.908359000	0.254310000
1	-5.829483000	-1.309012000	0.338968000
1	-1.087053000	-4.526822000	-1.891662000
1	-3.694537000	-3.894781000	-1.655194000
1	3.004110000	-1.687011000	-3.934028000
1	3.475452000	0.974468000	-4.222983000
1	1.427581000	5.154297000	-1.616668000
1	-0.720723000	5.474926000	-0.028273000
1	-5.484736000	3.085088000	-0.240173000
1	-6.370215000	0.708519000	-1.226764000
1	0.125006000	-4.283874000	-4.218235000
1	1.407127000	-6.399293000	-4.432650000
1	3.397621000	-6.793544000	-2.957752000
1	4.056717000	-5.072001000	-1.305139000
1	1.546719000	4.742646000	-4.495252000
1	3.499590000	6.174528000	-5.064599000
1	5.747019000	5.662085000	-4.105811000
1	6.016422000	3.709303000	-2.570187000
1	4.047667000	2.286190000	-2.008895000
1	-3.128758000	6.000868000	-0.380084000
1	-3.955806000	7.434404000	1.480043000
1	-4.441511000	6.404766000	3.708632000
1	-4.073142000	3.982040000	4.062111000
1	-7.921158000	-2.653303000	0.491106000
1	-8.713488000	-3.969236000	-1.476781000
1	-7.393425000	-3.934739000	-3.595944000
1	-5.304483000	-2.591447000	-3.738434000
1	4.805594000	-2.944892000	-1.479867000
1	4.080492000	-1.323752000	-1.580025000
1	-3.190182000	1.618740000	4.163207000
1	-4.790199000	1.919056000	3.420988000
7	-2.913790000	-0.765546000	2.828773000
19	1.402505000	-1.363096000	1.323043000
8	0.502110000	0.175500000	3.655026000
8	-0.936386000	-3.253279000	2.119109000
8	3.266171000	-0.330205000	3.113561000
8	1.901141000	-3.816020000	2.543091000
6	-2.304527000	-3.225978000	2.510604000
1	-2.390918000	-3.351296000	3.607598000
6	-3.040272000	-1.995012000	2.025157000
1	-2.812782000	-4.093932000	2.051066000
1	-4.108514000	-2.244973000	2.004605000
1	-2.727760000	-1.778162000	0.993062000
6	-0.381128000	-4.542524000	2.275162000
1	-0.374653000	-4.840496000	3.341535000
1	-0.989136000	-5.286279000	1.724146000
6	1.025525000	-4.597830000	1.729661000
1	1.356542000	-5.649234000	1.732645000
1	1.048643000	-4.243431000	0.683533000
6	3.229447000	-4.305380000	2.681220000
1	3.588616000	-3.965939000	3.661340000

1	3.218712000	-5.408060000	2.705374000
6	4.169660000	-3.869844000	1.567548000
1	5.114759000	-4.428290000	1.702444000
1	3.743980000	-4.173632000	0.609362000
6	-1.770144000	-0.557748000	3.716557000
6	-0.827316000	0.594367000	3.352906000
1	-2.119316000	-0.452068000	4.756564000
1	-1.169495000	-1.471978000	3.691765000
1	-1.049613000	1.504259000	3.937410000
1	-0.912924000	0.830793000	2.287126000
6	1.463921000	1.214279000	3.514751000
1	1.183479000	2.078917000	4.145392000
6	2.812588000	0.710774000	3.966421000
1	1.490481000	1.551019000	2.467703000
1	3.531875000	1.549833000	3.953014000
1	2.726380000	0.341259000	5.003607000
6	5.096387000	-1.870052000	2.730829000
1	5.683322000	-2.653301000	3.239090000
1	5.798313000	-1.100641000	2.391614000
6	4.138560000	-1.252726000	3.743966000
1	4.763779000	-0.742252000	4.498118000
1	3.530984000	-2.003175000	4.276825000
8	1.026920000	-2.347149000	4.835149000
1	0.877088000	-1.451990000	4.477818000
1	1.240920000	-2.888427000	4.054721000
6	-0.401316000	0.402735000	-0.136546000
8	-0.974045000	-0.520445000	0.466382000
8	0.579319000	1.072459000	0.285780000
6	3.566760000	2.154623000	0.670177000
6	4.890623000	2.093140000	1.137915000
6	5.393897000	3.116640000	1.942848000
6	4.597264000	4.215089000	2.291908000
6	3.284029000	4.278022000	1.812765000
6	2.766899000	3.262186000	1.005231000
1	5.489529000	1.222027000	0.871922000
1	6.423340000	3.053073000	2.306811000
1	4.996555000	5.011580000	2.924081000
1	2.648484000	5.131276000	2.066387000
1	1.747549000	3.312864000	0.623171000
8	3.102535000	1.147264000	-0.098509000
1	2.105416000	1.206471000	-0.169688000

^{5}RC1$_{\text{phenol,K+}}$:

6	4.050134000	2.937767000	-2.533917000
6	2.810131000	3.287795000	-3.103617000
6	2.759123000	4.429502000	-3.924841000
6	3.901754000	5.198876000	-4.163837000
6	5.123839000	4.841074000	-3.586278000
6	5.191191000	3.706058000	-2.769015000
6	1.582042000	2.494408000	-2.817671000
6	1.642581000	1.082455000	-3.046413000
7	0.701968000	0.170997000	-2.607644000
6	1.183812000	-1.089577000	-2.867531000
6	2.444372000	-0.982653000	-3.538158000
6	2.723655000	0.372099000	-3.661856000
26	-1.162373000	0.600129000	-1.838849000
7	-0.759596000	2.655653000	-1.993223000
6	-1.419057000	3.539058000	-1.157787000
6	-0.555070000	4.683569000	-0.906974000
6	0.600563000	4.476815000	-1.602010000
6	0.497869000	3.170223000	-2.237166000
6	-2.698005000	3.354501000	-0.622439000
6	-3.200333000	4.275972000	0.433286000
6	-3.425594000	3.777077000	1.748533000
6	-3.885471000	4.625679000	2.762384000
6	-4.134145000	5.977068000	2.490857000
6	-3.919098000	6.486464000	1.210513000
6	-3.456612000	5.634254000	0.200699000
6	0.510577000	-2.311308000	-2.542666000
6	1.292769000	-3.573600000	-2.622680000
6	0.914515000	-4.582970000	-3.532225000
6	1.599300000	-5.793279000	-3.632792000
6	2.704454000	-6.029934000	-2.810918000
6	3.108028000	-5.049739000	-1.903206000
6	2.417831000	-3.833965000	-1.811072000
8	2.811253000	-2.901503000	-0.861455000
6	4.147420000	-2.424270000	-0.982174000
6	4.754743000	-1.880940000	0.308169000
7	4.470830000	-2.476033000	1.519777000
8	-3.131088000	2.463284000	1.968390000
6	-3.659453000	1.778360000	3.098406000
6	-3.859014000	0.326273000	2.645701000
8	-4.900018000	0.064084000	2.043861000
7	-1.771350000	-1.381608000	-2.266284000
6	-0.860202000	-2.414097000	-2.248076000
6	-1.547263000	-3.645663000	-1.883998000
6	-2.868690000	-3.336819000	-1.723639000
6	-3.006310000	-1.907589000	-1.939523000
6	-4.186754000	-1.185353000	-1.708340000
6	-5.440885000	-1.969229000	-1.521982000
6	-6.156008000	-1.909277000	-0.309781000
6	-7.310554000	-2.674717000	-0.127918000
6	-7.777414000	-3.510805000	-1.149457000
6	-7.075982000	-3.577920000	-2.357093000
6	-5.918838000	-2.814546000	-2.539465000
7	-3.133883000	1.039487000	-1.406240000
6	-4.245168000	0.223859000	-1.508826000
6	-5.415778000	0.989982000	-1.194393000
6	-4.983748000	2.252276000	-0.822079000
6	-3.558469000	2.255587000	-0.952519000
8	5.499811000	-0.915841000	0.198126000
1	-5.783057000	-1.268666000	0.491753000
1	-1.078373000	-4.618322000	-1.757157000
1	-3.673584000	-4.009552000	-1.437008000
1	3.037220000	-1.818209000	-3.905112000
1	3.593954000	0.823617000	-4.131448000
1	1.475744000	5.120768000	-1.629499000
1	-0.803315000	5.534956000	-0.277730000
1	-5.588090000	3.096238000	-0.493865000
1	-6.438192000	0.620812000	-1.214625000
1	0.057907000	-4.389745000	-4.180583000
1	1.275621000	-6.546069000	-4.355112000
1	3.252697000	-6.972735000	-2.869660000
1	3.966452000	-5.238248000	-1.256069000
1	1.807388000	4.710869000	-4.381121000
1	3.837131000	6.079759000	-4.807908000
1	6.017582000	5.442469000	-3.770317000
1	6.137709000	3.423560000	-2.300559000
1	4.097697000	2.066521000	-1.882157000
1	-3.299384000	6.021529000	-0.808485000
1	-4.114704000	7.538814000	0.992627000
1	-4.492198000	6.627177000	3.292837000
1	-4.032732000	4.244616000	3.773506000
1	-7.847170000	-2.624823000	0.823509000
1	-8.681465000	-4.107506000	-1.003962000
1	-7.432121000	-4.225009000	-3.162944000
1	-5.373417000	-2.867573000	-3.484478000
1	4.816193000	-3.213768000	-1.363886000
1	4.170182000	-1.588825000	-1.695672000
1	-3.007160000	1.905884000	3.975497000
1	-4.660525000	2.157553000	3.348599000
7	-2.878034000	-0.594460000	2.839367000
19	1.469461000	-1.260462000	1.284134000
8	0.548065000	0.245588000	3.605892000

8	-0.984501000	-3.134224000	2.152495000
8	3.309610000	-0.317443000	3.125669000
8	1.849202000	-3.725888000	2.483907000
6	-2.327886000	-3.086545000	2.615435000
1	-2.356650000	-3.179615000	3.718585000
6	-3.088079000	-1.870194000	2.130151000
1	-2.867737000	-3.962537000	2.210420000
1	-4.156642000	-2.109535000	2.212777000
1	-2.868228000	-1.720455000	1.063015000
6	-0.443656000	-4.434501000	2.255111000
1	-0.413306000	-4.766081000	3.311232000
1	-1.074375000	-5.153638000	1.696524000
6	0.948872000	-4.484079000	1.672960000
1	1.275273000	-5.535812000	1.636500000
1	0.948558000	-4.098947000	0.638335000
6	3.144236000	-4.277917000	2.685963000
1	3.479439000	-3.937406000	3.674187000
1	3.080412000	-5.378180000	2.728585000
6	4.148779000	-3.906001000	1.604996000
1	5.076815000	-4.469990000	1.812438000
1	3.767131000	-4.247335000	0.641317000
6	-1.746688000	-0.413359000	3.748091000
6	-0.780733000	0.735118000	3.432333000
1	-2.111416000	-0.325280000	4.784716000
1	-1.152648000	-1.331083000	3.712168000
1	-0.927673000	1.584199000	4.122949000
1	-0.916553000	1.096851000	2.406689000
6	1.541741000	1.262320000	3.564095000
1	1.268505000	2.087770000	4.247348000
6	2.861912000	0.690062000	4.019672000
1	1.607812000	1.673814000	2.546662000
1	3.606674000	1.505349000	4.073234000
1	2.735922000	0.268510000	5.032702000
6	5.113097000	-1.884124000	2.699967000
1	5.714116000	-2.657040000	3.207559000
1	5.802003000	-1.115250000	2.333595000
6	4.181469000	-1.257556000	3.730581000
1	4.830121000	-0.759536000	4.473574000
1	3.573899000	-2.000468000	4.273425000
8	1.035826000	-2.277548000	4.805655000
1	0.887172000	-1.379735000	4.455225000
1	1.210295000	-2.815734000	4.013139000
6	-0.498042000	0.523001000	0.004690000
8	-0.970026000	-0.486662000	0.534285000
8	0.333061000	1.351883000	0.429673000
6	3.481326000	2.207524000	0.731417000
6	4.809893000	2.079953000	1.165582000
6	5.426260000	3.142017000	1.829871000
6	4.736934000	4.337949000	2.069821000
6	3.414917000	4.459377000	1.628294000
6	2.784579000	3.405523000	0.961832000
1	5.328254000	1.140726000	0.973058000
1	6.460911000	3.032857000	2.166736000
1	5.225597000	5.164332000	2.590961000
1	2.862234000	5.387114000	1.800475000
1	1.758187000	3.498799000	0.605940000
8	2.902806000	1.163691000	0.092652000
1	1.933988000	1.344819000	-0.025383000

^{3}RC1 $_{phenol, 6W, K+}$:

6	6.203928000	1.054753000	-2.205627000
6	6.184281000	1.254307000	-0.814161000
6	7.408721000	1.402331000	-0.141477000
6	8.619776000	1.359884000	-0.838513000
6	8.626271000	1.166414000	-2.224094000
6	7.413638000	1.014180000	-2.904562000
6	4.893589000	1.272229000	-0.062919000
6	4.003592000	2.319753000	-0.261689000
7	2.792756000	2.503835000	0.384596000
6	2.187379000	3.596246000	-0.225202000
6	3.044828000	4.105703000	-1.277334000
6	4.180845000	3.355950000	-1.263990000
26	1.881461000	1.205293000	1.595826000
7	3.391038000	-0.076341000	1.392022000
6	3.423533000	-1.393923000	1.823269000
6	4.653425000	-2.006730000	1.452777000
6	5.389661000	-1.047527000	0.781319000
6	4.582416000	0.131039000	0.720032000
6	2.400001000	-2.026706000	2.563517000
6	2.415591000	-3.514632000	2.682059000
6	1.749528000	-4.282277000	1.697652000
6	1.687883000	-5.676606000	1.810614000
6	2.308134000	-6.315592000	2.890017000
6	2.984574000	-5.574988000	3.860432000
6	3.027898000	-4.179958000	3.748280000
6	0.982523000	4.163342000	0.149307000
6	0.361400000	5.224155000	-0.693564000
6	0.865743000	6.529251000	-0.783411000
6	0.232385000	7.501365000	-1.565288000
6	-0.924782000	7.174210000	-2.277530000
6	-1.443618000	5.877075000	-2.214828000
6	-0.804961000	4.915551000	-1.426574000
8	-1.251810000	3.623551000	-1.362151000
6	-2.618467000	3.356797000	-1.098709000
6	-3.283925000	2.767294000	-2.340073000
7	-4.426828000	2.029019000	-2.153912000
8	1.221428000	-3.578672000	0.658983000
6	0.352180000	-4.173347000	-0.272096000
6	-1.061542000	-4.291763000	0.300982000
8	-1.262557000	-4.024989000	1.488326000
7	0.563894000	2.644317000	2.076234000
6	0.282143000	3.768343000	1.320531000
6	-0.788391000	4.498492000	1.902452000
6	-1.175675000	3.801225000	3.041272000
6	-0.346780000	2.637360000	3.114403000
6	-0.580339000	1.544799000	3.996090000
6	-1.716983000	1.671063000	4.953899000
6	-2.892762000	0.922162000	4.763164000
6	-3.970382000	1.050642000	5.643582000
6	-3.897283000	1.936766000	6.723959000
6	-2.736469000	2.692991000	6.918398000
6	-1.657426000	2.560789000	6.039452000
7	1.129127000	0.036294000	3.036186000
6	0.074972000	0.326220000	3.883682000
6	-0.299098000	-0.871682000	4.617784000
6	0.466829000	-1.891263000	4.144361000
6	1.379167000	-1.320894000	3.175175000
8	-2.782672000	2.964641000	-3.443157000
1	-2.949012000	0.247934000	3.907147000
1	-1.192939000	5.431975000	1.518073000
1	-1.959140000	4.061782000	3.748303000
1	2.812584000	4.955511000	-1.914277000
1	5.058692000	3.468315000	-1.894563000
1	6.371374000	-1.156542000	0.328340000
1	4.927996000	-3.035729000	1.672090000
1	0.453467000	-2.937291000	4.440226000
1	-1.070836000	-0.918490000	5.380897000
1	1.762226000	6.783752000	-0.213580000
1	0.640773000	8.513292000	-1.613870000
1	-1.422619000	7.925540000	-2.895195000
1	-2.322650000	5.591779000	-2.794852000
1	7.405968000	1.551995000	0.940932000
1	9.562108000	1.481095000	-0.298060000
1	9.572400000	1.132507000	-2.769973000

1	7.408835000	0.854805000	-3.986053000
1	5.254696000	0.921005000	-2.725827000
1	3.538814000	-3.583111000	4.507644000
1	3.467038000	-6.074829000	4.703093000
1	2.253772000	-7.404317000	2.967068000
1	1.154050000	-6.272460000	1.070528000
1	-4.876260000	0.460575000	5.480861000
1	-4.741375000	2.039023000	7.410507000
1	-2.669347000	3.386656000	7.760472000
1	-0.751654000	3.152264000	6.193393000
1	-2.650069000	2.670148000	-0.243156000
1	-3.152264000	4.276805000	-0.805461000
1	0.338379000	-3.485384000	-1.128519000
1	0.718669000	-5.151287000	-0.627254000
7	-2.057924000	-4.659778000	-0.548493000
19	-2.429230000	-0.861781000	-1.275507000
8	-2.679318000	-3.213972000	-3.098002000
8	-4.379772000	-2.616101000	-0.417203000
8	-3.967173000	-0.509118000	-3.631453000
8	-5.736664000	-0.263922000	-0.547054000
6	-4.475325000	-4.000635000	-0.694754000
1	-4.386862000	-4.103487000	-1.784944000
6	-3.408183000	-4.842101000	-0.006767000
1	-5.467367000	-4.378849000	-0.390424000
1	-3.687837000	-5.905220000	-0.110029000
1	-3.366505000	-4.611405000	1.063310000
6	-5.096657000	-2.177185000	0.725056000
1	-6.112221000	-2.612452000	0.702254000
1	-4.595925000	-2.482064000	1.658830000
6	-5.186394000	-0.666264000	0.706591000
1	-5.847167000	-0.344115000	1.532596000
1	-4.201856000	-0.208133000	0.877967000
6	-6.222320000	1.061467000	-0.640463000
1	-6.968752000	1.059025000	-1.447501000
1	-6.753948000	1.338115000	0.287788000
6	-5.157988000	2.125303000	-0.878220000
1	-5.676974000	3.100653000	-0.818239000
1	-4.437256000	2.079495000	-0.052151000
6	-1.824247000	-5.163464000	-1.907655000
6	-1.613368000	-4.146471000	-3.024756000
1	-0.950523000	-5.833928000	-1.914169000
1	-2.689523000	-5.792935000	-2.157503000
1	-1.556679000	-4.725464000	-3.966712000
1	-0.665062000	-3.607578000	-2.901972000
6	-2.671607000	-2.459783000	-4.298351000
1	-2.598226000	-3.143898000	-5.164788000
6	-3.952939000	-1.671096000	-4.444810000
1	-1.795877000	-1.784295000	-4.337491000
1	-4.062067000	-1.378889000	-5.506587000
1	-4.805143000	-2.330078000	-4.193408000
6	-5.134801000	1.625057000	-3.369873000
1	-6.158655000	2.034894000	-3.356106000
1	-4.600090000	2.098056000	-4.201041000
6	-5.238364000	0.130408000	-3.650037000
1	-5.683024000	0.043674000	-4.659007000
1	-5.913236000	-0.392302000	-2.959070000
8	-7.085422000	-2.270042000	-1.925880000
1	-6.334619000	-2.785403000	-2.248502000
1	-6.646106000	-1.570161000	-1.401007000
6	0.649983000	0.513876000	0.318122000
8	-0.547531000	0.257293000	0.565623000
8	1.162198000	0.349924000	-0.840297000
6	3.456319000	-1.259420000	-2.654657000
6	4.161464000	-1.707669000	-3.782201000
6	4.614349000	-3.027391000	-3.842273000
6	4.368561000	-3.911250000	-2.784421000
6	3.668091000	-3.454104000	-1.662961000
6	3.214431000	-2.136528000	-1.589343000
1	4.344817000	-1.011355000	-4.603730000
1	5.163197000	-3.366861000	-4.724666000
1	4.722289000	-4.943657000	-2.833828000
1	3.472821000	-4.122435000	-0.821758000
1	2.680324000	-1.789768000	-0.707139000
8	3.015179000	0.027512000	-2.650240000
1	2.502365000	0.222614000	-1.819816000
8	-2.272620000	-1.480518000	1.782436000
1	-1.498001000	-0.905730000	1.638744000
1	-1.899942000	-2.381772000	1.814176000
8	0.750011000	-0.273126000	-4.404831000
1	0.151756000	0.369192000	-3.953425000
1	1.615817000	-0.063654000	-4.003873000
8	0.031541000	-1.731291000	-2.183602000
1	0.275006000	-1.395493000	-3.077104000
1	0.485238000	-1.087843000	-1.595333000
8	-0.715077000	1.145828000	-2.639630000
1	-0.877498000	2.104282000	-2.596577000
1	-0.034245000	0.966586000	-1.948409000
8	-2.776081000	1.555467000	1.479738000
1	-2.497277000	2.318278000	2.013618000
1	-1.918919000	1.126861000	1.261692000

^{5}RC1$_{phenol, 6W, K+}$:

6	6.359823000	0.581376000	-2.248818000
6	6.305455000	0.901151000	-0.880083000
6	7.511257000	1.027749000	-0.170508000
6	8.740556000	0.846801000	-0.811591000
6	8.782887000	0.530774000	-2.173379000
6	7.587579000	0.396970000	-2.888771000
6	4.993057000	1.055730000	-0.194528000
6	4.149498000	2.130027000	-0.580492000
7	2.921703000	2.418448000	-0.010304000
6	2.367177000	3.433940000	-0.772119000
6	3.250828000	3.787147000	-1.831001000
6	4.387028000	3.002290000	-1.682379000
26	1.942505000	1.341195000	1.366535000
7	3.363155000	-0.059353000	1.313545000
6	3.296761000	-1.326634000	1.864937000
6	4.502513000	-2.040502000	1.609345000
6	5.325106000	-1.190484000	0.892063000
6	4.594858000	0.021453000	0.682547000
6	2.214265000	-1.815482000	2.629575000
6	2.160247000	-3.270241000	2.948032000
6	1.454057000	-4.138856000	2.086431000
6	1.343399000	-5.501217000	2.390982000
6	1.951616000	-6.007099000	3.544579000
6	2.664622000	-5.164945000	4.399757000
6	2.757717000	-3.801928000	4.095443000
6	1.136701000	4.068194000	-0.473464000
6	0.503465000	4.968425000	-1.468562000
6	1.046594000	6.202443000	-1.856268000
6	0.381411000	7.034700000	-2.762451000
6	-0.843814000	6.634129000	-3.303077000
6	-1.400828000	5.401686000	-2.946367000
6	-0.730734000	4.578293000	-2.036699000
8	-1.199421000	3.346174000	-1.681661000
6	-2.555560000	3.175926000	-1.330416000
6	-3.311813000	2.465799000	-2.448263000
7	-4.509233000	1.886187000	-2.140915000
8	0.942274000	-3.562328000	0.965320000
6	0.071214000	-4.258683000	0.108440000
6	-1.342378000	-4.302759000	0.690449000
8	-1.535676000	-3.942899000	1.854737000
7	0.740906000	2.904803000	1.682662000
6	0.468386000	3.902142000	0.770065000

6	-0.525508000	4.779674000	1.283551000
6	-0.859431000	4.311315000	2.550573000
6	-0.089660000	3.126126000	2.769541000
6	-0.335600000	2.193066000	3.806492000
6	-1.323817000	2.577205000	4.850079000
6	-2.600567000	1.986831000	4.880799000
6	-3.536556000	2.363797000	5.846728000
6	-3.215210000	3.339628000	6.796980000
6	-1.952688000	3.940999000	6.769403000
6	-1.016899000	3.564496000	5.801493000
7	1.101033000	0.378220000	2.910947000
6	0.155155000	0.862293000	3.792876000
6	-0.302277000	-0.192641000	4.642211000
6	0.333030000	-1.351023000	4.227351000
6	1.224664000	-0.977722000	3.180218000
8	-2.825194000	2.436770000	-3.580653000
1	-2.853879000	1.250853000	4.116736000
1	-0.909191000	5.660094000	0.772893000
1	-1.578576000	4.731673000	3.249494000
1	3.067829000	4.554835000	-2.578180000
1	5.282479000	3.012975000	-2.297395000
1	6.316042000	-1.399815000	0.498307000
1	4.705010000	-3.059397000	1.929816000
1	0.231984000	-2.354543000	4.633242000
1	-1.029682000	-0.086018000	5.442026000
1	1.993469000	6.520856000	-1.415295000
1	0.817162000	7.997112000	-3.040048000
1	-1.367385000	7.275999000	-4.015673000
1	-2.334400000	5.057561000	-3.393141000
1	7.479632000	1.269418000	0.894473000
1	9.668992000	0.953420000	-0.244818000
1	9.743258000	0.386351000	-2.674403000
1	7.610948000	0.139093000	-3.950774000
1	5.422745000	0.462660000	-2.793814000
1	3.299197000	-3.125355000	4.760786000
1	3.137829000	-5.561217000	5.300689000
1	1.859165000	-7.071947000	3.772004000
1	0.780253000	-6.172521000	1.743175000
1	-4.525907000	1.898980000	5.853022000
1	-3.947592000	3.633598000	7.552851000
1	-1.694056000	4.705952000	7.505973000
1	-0.031653000	4.036191000	5.779389000
1	-2.571771000	2.602079000	-0.395025000
1	-3.041676000	4.145988000	-1.126669000
1	0.073030000	-3.673919000	-0.820894000
1	0.432170000	-5.275548000	-0.123367000
7	-2.348466000	-4.710531000	-0.127624000
19	-2.437270000	-1.005470000	-1.183989000
8	-2.930920000	-3.427905000	-2.760654000
8	-4.497278000	-2.513714000	-0.097035000
8	-4.190021000	-0.771360000	-3.413315000
8	-5.842044000	-0.149197000	-0.258909000
6	-4.717004000	-3.898048000	-0.288134000
1	-4.666509000	-4.067343000	-1.372066000
6	-3.705315000	-4.789933000	0.421407000
1	-5.729021000	-4.171533000	0.060815000
1	-4.055616000	-5.833904000	0.348949000
1	-3.641756000	-4.531187000	1.484122000
6	-5.064380000	-1.960229000	1.079000000
1	-6.083026000	-2.366303000	1.216612000
1	-4.460177000	-2.196922000	1.970087000
6	-5.130615000	-0.454380000	0.936934000
1	-5.662112000	-0.039894000	1.813906000
1	-4.123977000	-0.012899000	0.929515000
6	-6.256949000	1.189329000	-0.437330000
1	-7.061087000	1.159971000	-1.186133000
1	-6.699865000	1.581193000	0.496630000
6	-5.156343000	2.161159000	-0.846693000
1	-5.618372000	3.165960000	-0.866135000
1	-4.390168000	2.165852000	-0.060924000
6	-2.130265000	-5.314747000	-1.447874000
6	-1.892258000	-4.383998000	-2.633499000
1	-1.276809000	-6.009454000	-1.405462000
1	-3.015275000	-5.932174000	-1.654828000
1	-1.859600000	-5.026587000	-3.534165000
1	-0.926540000	-3.867695000	-2.554394000
6	-2.936599000	-2.763875000	-4.013092000
1	-2.896093000	-3.508552000	-4.830061000
6	-4.208793000	-1.964079000	-4.179337000
1	-2.050153000	-2.109292000	-4.118261000
1	-4.328073000	-1.715706000	-5.251168000
1	-5.065410000	-2.600545000	-3.887587000
6	-5.303894000	1.396886000	-3.270318000
1	-6.314296000	1.833459000	-3.220311000
1	-4.818414000	1.780715000	-4.174556000
6	-5.451455000	-0.114256000	-3.407061000
1	-5.965768000	-0.281591000	-4.371268000
1	-6.084685000	-0.552941000	-2.624231000
8	-7.385685000	-2.186089000	-1.335440000
1	-6.708253000	-2.749566000	-1.731091000
1	-6.856058000	-1.481328000	-0.909812000
6	0.660073000	0.599174000	0.124325000
8	-0.543461000	0.457037000	0.420245000
8	1.145737000	0.278476000	-1.005350000
6	3.472770000	-1.648243000	-2.496251000
6	4.255432000	-2.224890000	-3.508887000
6	4.667810000	-3.554611000	-3.400573000
6	4.303563000	-4.322661000	-2.287697000
6	3.526178000	-3.738648000	-1.281977000
6	3.112499000	-2.409454000	-1.377136000
1	4.530253000	-1.618946000	-4.375204000
1	5.278056000	-3.993663000	-4.194288000
1	4.625554000	-5.363341000	-2.205472000
1	3.238417000	-4.314651000	-0.399965000
1	2.519380000	-1.963643000	-0.582196000
8	3.073291000	-0.358380000	-2.659061000
1	2.518066000	-0.052238000	-1.891346000
8	-2.143425000	-1.264855000	1.744115000
1	-1.345636000	-0.731264000	1.575458000
1	-1.819379000	-2.162934000	1.940434000
8	0.839399000	-0.926873000	-4.406126000
1	0.204787000	-0.242677000	-4.084493000
1	1.681641000	-0.637830000	-4.005013000
8	0.003815000	-2.011905000	-2.015092000
1	0.274569000	-1.827533000	-2.943915000
1	0.440161000	-1.274343000	-1.535675000
8	-0.778214000	0.685525000	-2.983012000
1	-1.181820000	1.554064000	-3.168579000
1	-0.131943000	0.806396000	-2.254696000
8	-2.655369000	1.816436000	1.539122000
1	-2.308339000	2.654699000	1.887035000
1	-1.837443000	1.348772000	1.259144000

^{3}RC1$_{\text{acetate,K+}}$:

6	2.983247000	4.971835000	-1.914076000
6	1.685806000	4.799636000	-2.429436000
6	1.136508000	5.842317000	-3.198149000
6	1.854658000	7.016375000	-3.441297000
6	3.143040000	7.172287000	-2.920867000
6	3.703372000	6.143959000	-2.155245000
6	0.884609000	3.570286000	-2.160903000
6	1.405893000	2.309395000	-2.559549000
7	0.776192000	1.091383000	-2.355775000
6	1.670555000	0.115951000	-2.736118000

6	2.870883000	0.718675000	-3.226736000	1	-5.807343000	5.701315000	2.629449000
6	2.695004000	2.090518000	-3.139263000	1	-6.065082000	4.125807000	4.557106000
26	-1.077737000	0.793326000	-1.658155000	1	-5.031050000	1.876123000	4.464231000
7	-1.240118000	2.725781000	-1.195890000	1	-6.661011000	-4.672346000	-0.731624000
6	-2.121904000	3.238505000	-0.260984000	1	-6.999107000	-5.637005000	-3.008937000
6	-1.728729000	4.592481000	0.078378000	1	-5.689696000	-4.739042000	-4.935956000
6	-0.656918000	4.906160000	-0.702750000	1	-4.061921000	-2.891621000	-4.580634000
6	-0.307346000	3.717617000	-1.454964000	1	5.691357000	-1.347769000	-1.223088000
6	-3.259102000	2.575700000	0.202046000	1	4.612865000	0.064428000	-1.357857000
6	-4.027956000	3.041204000	1.382751000	1	-3.586907000	-0.150600000	4.180763000
6	-4.197527000	2.158394000	2.487981000	1	-5.144011000	-0.110735000	3.301031000
6	-4.925060000	2.553222000	3.615752000	7	-2.540683000	-2.004771000	2.450915000
6	-5.500554000	3.828832000	3.670009000	19	1.769366000	-1.198782000	1.354973000
6	-5.351071000	4.709310000	2.598485000	8	0.420352000	-0.379947000	3.774413000
6	-4.624361000	4.308356000	1.471612000	8	0.108352000	-3.697511000	1.575906000
6	1.408120000	-1.282104000	-2.720494000	8	3.243183000	0.029337000	3.500839000
6	2.519245000	-2.234048000	-2.991252000	8	2.978192000	-3.564808000	2.202120000
6	2.510196000	-3.008252000	-4.169819000	6	-1.242059000	-4.100210000	1.789886000
6	3.501727000	-3.946566000	-4.453381000	1	-1.373657000	-4.454912000	2.830472000
6	4.548102000	-4.139894000	-3.546971000	6	-2.249142000	-3.027554000	1.430318000
6	4.590452000	-3.382844000	-2.376091000	1	-1.451470000	-4.962990000	1.131029000
6	3.595555000	-2.434422000	-2.104166000	1	-3.199002000	-3.533533000	1.217828000
8	3.658046000	-1.718082000	-0.915816000	1	-1.922033000	-2.525418000	0.508143000
6	4.794108000	-0.864672000	-0.800008000	6	0.988238000	-4.803161000	1.626594000
6	5.118703000	-0.439254000	0.627920000	1	1.009736000	-5.238496000	2.644041000
7	5.043539000	-1.352552000	1.657171000	1	0.641532000	-5.592760000	0.932370000
8	-3.589723000	0.940137000	2.399920000	6	2.387985000	-4.412513000	1.215361000
6	-4.052101000	-0.154433000	3.181996000	1	2.985379000	-5.333532000	1.115416000
6	-3.759965000	-1.411154000	2.352657000	1	2.376676000	-3.912141000	0.230176000
8	-4.627521000	-1.797946000	1.570990000	6	4.387688000	-3.680812000	2.361960000
7	-1.036082000	-1.044544000	-2.467647000	1	4.604861000	-3.449267000	3.412870000
6	0.113590000	-1.794574000	-2.617973000	1	4.696012000	-4.725819000	2.191537000
6	-0.230142000	-3.201532000	-2.705881000	6	5.194285000	-2.793358000	1.423732000
6	-1.592546000	-3.281996000	-2.664542000	1	6.257842000	-3.069772000	1.546303000
6	-2.096370000	-1.937394000	-2.491930000	1	4.910235000	-3.022985000	0.394773000
6	-3.438493000	-1.641309000	-2.252147000	6	-1.560799000	-1.674531000	3.483131000
6	-4.441421000	-2.724384000	-2.461874000	6	-0.957769000	-0.266778000	3.423335000
6	-5.186498000	-3.237207000	-1.382446000	1	-1.989260000	-1.863740000	4.480432000
6	-6.097303000	-4.278085000	-1.581477000	1	-0.728740000	-2.377203000	3.376780000
6	-6.285113000	-4.823608000	-2.857079000	1	-1.448641000	0.416902000	4.136936000
6	-5.550103000	-4.321817000	-3.935334000	1	-1.055323000	0.148878000	2.415155000
6	-4.636029000	-3.282403000	-3.737350000	6	1.049791000	0.864619000	4.045273000
7	-3.001053000	0.576882000	-1.243073000	1	0.497218000	1.402570000	4.838190000
6	-3.864292000	-0.419774000	-1.684226000	6	2.451621000	0.601334000	4.533567000
6	-5.196775000	-0.120393000	-1.259494000	1	1.045891000	1.493953000	3.141530000
6	-5.120845000	1.011411000	-0.471042000	1	2.904498000	1.548782000	4.879772000
6	-3.751708000	1.419698000	-0.469422000	1	2.401847000	-0.086372000	5.395988000
8	5.458359000	0.724192000	0.799465000	6	5.438546000	-0.861003000	2.981804000
1	-5.032510000	-2.818227000	-0.385581000	1	6.217460000	-1.523135000	3.393981000
1	0.479805000	-4.019539000	-2.793797000	1	5.883710000	0.129157000	2.833465000
1	-2.206902000	-4.178006000	-2.705900000	6	4.320163000	-0.745754000	4.006902000
1	3.722530000	0.179381000	-3.635862000	1	4.757542000	-0.260522000	4.898131000
1	3.392279000	2.866293000	-3.443229000	1	3.925955000	-1.724444000	4.327646000
1	-0.096622000	5.837388000	-0.714327000	8	1.581621000	-2.848570000	4.599591000
1	-2.220786000	5.225563000	0.811570000	1	1.183486000	-1.988213000	4.372066000
1	-5.926963000	1.523659000	0.050207000	1	2.009470000	-3.147712000	3.777767000
1	-6.077799000	-0.715212000	-1.485042000	6	-0.312400000	0.375135000	0.032556000
1	1.693044000	-2.852494000	-4.876652000	8	-0.637143000	-0.780874000	0.353410000
1	3.459429000	-4.523225000	-5.380107000	8	0.465580000	1.120351000	0.693215000
1	5.329778000	-4.876405000	-3.745489000	6	3.108860000	2.678492000	0.895219000
1	5.403879000	-3.533269000	-1.663921000	1	3.245571000	1.896126000	1.657620000
1	0.131285000	5.722953000	-3.608335000	1	4.094095000	3.090870000	0.653977000
1	1.406462000	7.811193000	-4.042988000	1	2.661724000	2.198739000	0.014241000
1	3.706537000	8.089428000	-3.108926000	6	2.210000000	3.759201000	1.445902000
1	4.705574000	6.258142000	-1.734293000	8	2.617638000	4.851011000	1.800533000
1	3.422071000	4.182863000	-1.305242000	8	0.928821000	3.441633000	1.576065000
1	-4.535774000	4.983449000	0.618370000	1	0.699640000	2.559549000	1.126498000

^{5}**RC1** $_{acetate,K+}$:

6	2.245254000	5.356614000	-1.794180000
6	0.970099000	5.056483000	-2.310380000
6	0.293500000	6.072604000	-3.013737000
6	0.861162000	7.337609000	-3.186160000
6	2.125994000	7.618824000	-2.660102000
6	2.814569000	6.620022000	-1.962593000
6	0.326869000	3.727221000	-2.111318000
6	1.077757000	2.560341000	-2.468625000
7	0.675028000	1.263020000	-2.219167000
6	1.685850000	0.420417000	-2.615467000
6	2.770727000	1.201592000	-3.134433000
6	2.380979000	2.532556000	-3.065201000
26	-1.137694000	0.644162000	-1.425079000
7	-1.805337000	2.646665000	-1.386343000
6	-2.777505000	2.995956000	-0.461897000
6	-2.548311000	4.368111000	-0.035387000
6	-1.456796000	4.825578000	-0.711863000
6	-0.944143000	3.720066000	-1.510675000
6	-3.789046000	2.150619000	0.004884000
6	-4.642160000	2.523241000	1.161278000
6	-4.652771000	1.684261000	2.315126000
6	-5.457003000	1.994080000	3.417067000
6	-6.264295000	3.138450000	3.401202000
6	-6.265365000	3.977404000	2.286359000
6	-5.461583000	3.662621000	1.184872000
6	1.615212000	-1.009317000	-2.623268000
6	2.843218000	-1.773863000	-2.987931000
6	2.902320000	-2.450900000	-4.222821000
6	4.001106000	-3.223723000	-4.596917000
6	5.086962000	-3.346264000	-3.725009000
6	5.061234000	-2.683670000	-2.497738000
6	3.958339000	-1.897008000	-2.139106000
8	3.949154000	-1.271529000	-0.899450000
6	4.989068000	-0.318340000	-0.705531000
6	5.254550000	0.021276000	0.756976000
7	5.237915000	-0.971871000	1.713268000
8	-3.811257000	0.609076000	2.308891000
6	-4.081151000	-0.539675000	3.107319000
6	-3.625205000	-1.731746000	2.251663000
8	-4.457422000	-2.272965000	1.531316000
7	-0.834158000	-1.250443000	-2.319915000
6	0.432279000	-1.758639000	-2.493591000
6	0.354198000	-3.210850000	-2.572179000
6	-0.968831000	-3.546920000	-2.493479000
6	-1.716014000	-2.314843000	-2.321766000
6	-3.096343000	-2.244136000	-2.080456000
6	-3.898431000	-3.479699000	-2.301006000
6	-4.669567000	-4.043314000	-1.264795000
6	-5.393692000	-5.219582000	-1.474613000
6	-5.370699000	-5.855843000	-2.721211000
6	-4.613078000	-5.304417000	-3.759186000
6	-3.885848000	-4.129451000	-3.549654000
7	-3.109044000	0.048783000	-1.126049000
6	-3.756013000	-1.097023000	-1.559999000
6	-5.157536000	-0.970412000	-1.291035000
6	-5.336010000	0.236475000	-0.635857000
6	-4.047033000	0.850842000	-0.553170000
8	5.486906000	1.192963000	1.025793000
1	-4.682277000	-3.552213000	-0.290179000
1	1.200646000	-3.885837000	-2.671777000
1	-1.397588000	-4.546020000	-2.509241000
1	3.691771000	0.802837000	-3.555502000
1	2.945048000	3.397131000	-3.404960000
1	-0.990022000	5.803004000	-0.621983000
1	-3.143170000	4.908611000	0.696915000
1	-6.265262000	0.667487000	-0.266754000
1	-5.918907000	-1.705041000	-1.540546000
1	2.049076000	-2.356535000	-4.897431000
1	4.009774000	-3.729818000	-5.564879000
1	5.951739000	-3.957034000	-3.993460000
1	5.903228000	-2.783806000	-1.810197000
1	-0.692118000	5.857236000	-3.431824000
1	0.314097000	8.106044000	-3.738498000
1	2.571730000	8.607747000	-2.792103000
1	3.798450000	6.828240000	-1.534448000
1	2.789134000	4.594770000	-1.238917000
1	-5.482469000	4.300988000	0.299113000
1	-6.897119000	4.868359000	2.265038000
1	-6.886523000	3.368977000	4.269295000
1	-5.440502000	1.356094000	4.301964000
1	-5.977060000	-5.647520000	-0.654823000
1	-5.939668000	-6.774916000	-2.882437000
1	-4.591315000	-5.788318000	-4.739130000
1	-3.300146000	-3.698791000	-4.364685000
1	5.935725000	-0.671663000	-1.148132000
1	4.717317000	0.626894000	-1.197207000
1	-3.582859000	-0.458431000	4.086316000
1	-5.161326000	-0.659353000	3.268200000
7	-2.296591000	-2.051389000	2.244225000
19	2.044674000	-1.154445000	1.307954000
8	0.491740000	-0.328685000	3.665609000
8	0.528017000	-3.532658000	1.357197000
8	3.287950000	0.170343000	3.518076000
8	3.355262000	-3.396017000	2.136995000
6	-0.842566000	-3.939295000	1.329638000
1	-1.098508000	-4.471534000	2.265309000
6	-1.782711000	-2.776244000	1.073537000
1	-0.965038000	-4.656876000	0.498546000
1	-2.659658000	-3.164123000	0.549639000
1	-1.291775000	-2.061102000	0.399859000
6	1.401463000	-4.643490000	1.446426000
1	1.351296000	-5.093948000	2.455740000
1	1.099629000	-5.418283000	0.716894000
6	2.829021000	-4.259594000	1.127350000
1	3.428890000	-5.183808000	1.084352000
1	2.885212000	-3.776512000	0.134330000
6	4.773825000	-3.389797000	2.276308000
1	4.981824000	-3.201743000	3.337880000
1	5.172029000	-4.390874000	2.042176000
6	5.495843000	-2.381472000	1.388162000
1	6.578151000	-2.582528000	1.490122000
1	5.223621000	-2.566653000	0.346865000
6	-1.412654000	-1.755620000	3.368640000
6	-0.927190000	-0.309226000	3.516707000
1	-1.893497000	-2.087994000	4.300800000
1	-0.524547000	-2.388354000	3.257880000
1	-1.364690000	0.161018000	4.416209000
1	-1.210057000	0.294875000	2.648240000
6	1.053048000	0.933831000	3.996257000
1	0.456087000	1.417469000	4.791298000
6	2.449056000	0.719951000	4.524785000
1	1.030714000	1.596591000	3.115739000
1	2.860479000	1.682716000	4.880426000
1	2.395252000	0.032840000	5.387362000
6	5.561606000	-0.544916000	3.079095000
1	6.354655000	-1.193851000	3.483840000
1	5.961824000	0.472513000	3.008843000
6	4.393184000	-0.545615000	4.053619000
1	4.750172000	-0.064617000	4.981389000
1	4.055413000	-1.560870000	4.320215000
8	1.779160000	-2.763810000	4.471797000
1	1.360228000	-1.917261000	4.229814000

1	2.284593000	-3.035136000	3.685714000
6	-0.509632000	0.963594000	0.379677000
8	-0.149770000	-0.221607000	0.508057000
8	-0.439526000	1.934483000	1.137785000
6	2.663689000	2.657706000	0.762732000
1	2.713465000	1.936785000	1.593540000
1	3.679176000	2.754372000	0.366295000
1	1.983210000	2.265936000	-0.004234000
6	2.200113000	3.986938000	1.311771000
8	2.960462000	4.900508000	1.566214000
8	0.896548000	4.115533000	1.547011000
1	0.373722000	3.307374000	1.279016000

^{5}TS1$_{acetate,K+}$:

6	-0.664593000	6.336691000	0.873055000
6	0.356958000	5.683096000	1.584103000
6	1.170799000	6.444371000	2.441765000
6	0.964924000	7.817925000	2.598880000
6	-0.059991000	8.456763000	1.894034000
6	-0.868341000	7.711175000	1.029423000
6	0.615524000	4.217788000	1.445691000
6	-0.391359000	3.318916000	1.880406000
7	-0.292267000	1.933467000	1.879317000
6	-1.477462000	1.418542000	2.350795000
6	-2.376029000	2.507266000	2.636831000
6	-1.692608000	3.678369000	2.371361000
26	1.303535000	0.831847000	1.311381000
7	2.310921000	2.555866000	0.725000000
6	3.285174000	2.542237000	-0.245645000
6	3.438916000	3.888874000	-0.762248000
6	2.577289000	4.695632000	-0.059919000
6	1.821216000	3.845448000	0.827029000
6	4.011515000	1.381600000	-0.611594000
6	4.823966000	1.294511000	-1.843968000
6	4.634477000	0.186559000	-2.728514000
6	5.382590000	0.070821000	-3.904404000
6	6.336073000	1.041955000	-4.235877000
6	6.542745000	2.130583000	-3.388404000
6	5.795149000	2.242963000	-2.211681000
6	-1.712257000	0.048421000	2.647636000
6	-3.060712000	-0.360131000	3.136521000
6	-3.228368000	-0.696150000	4.495196000
6	-4.446164000	-1.139946000	5.007949000
6	-5.546120000	-1.269749000	4.154483000
6	-5.412824000	-0.941270000	2.805955000
6	-4.187190000	-0.480094000	2.304182000
8	-4.079330000	-0.182210000	0.952440000
6	-4.932557000	0.866163000	0.503266000
6	-5.218255000	0.855002000	-0.995014000
7	-5.406022000	-0.338634000	-1.659445000
8	3.666364000	-0.717627000	-2.394518000
6	3.695615000	-2.039719000	-2.919411000
6	3.069609000	-2.927040000	-1.832702000
8	3.819544000	-3.390343000	-0.977436000
7	0.642822000	-0.724668000	2.530368000
6	-0.697346000	-0.935576000	2.734960000
6	-0.906993000	-2.322747000	3.099211000
6	0.330465000	-2.919736000	3.140260000
6	1.297708000	-1.921049000	2.759943000
6	2.661763000	-2.167661000	2.505597000
6	3.226188000	-3.448787000	3.015799000
6	3.824806000	-4.381680000	2.147414000
6	4.322948000	-5.590986000	2.639848000
6	4.238559000	-5.891190000	4.003918000
6	3.646334000	-4.971672000	4.875562000
6	3.142937000	-3.764185000	4.384537000
7	3.035553000	-0.198885000	1.042286000
6	3.485064000	-1.323829000	1.731330000
6	4.856174000	-1.555733000	1.373690000
6	5.190068000	-0.621542000	0.417000000
6	4.037803000	0.217148000	0.230172000
8	-5.283678000	1.941354000	-1.555568000
1	3.880896000	-4.151060000	1.082243000
1	-1.870319000	-2.785507000	3.299029000
1	0.554753000	-3.959774000	3.365522000
1	-3.382401000	2.402037000	3.036768000
1	-2.052523000	4.695046000	2.507393000
1	2.414930000	5.762780000	-0.195284000
1	4.113007000	4.193556000	-1.558181000
1	6.142442000	-0.498890000	-0.095398000
1	5.484731000	-2.349236000	1.768951000
1	-2.362719000	-0.601878000	5.153997000
1	-4.536868000	-1.385861000	6.068311000
1	-6.505942000	-1.628648000	4.532499000
1	-6.268471000	-1.048063000	2.136833000
1	1.971018000	5.945404000	2.993376000
1	1.606162000	8.389710000	3.274606000
1	-0.223904000	9.530692000	2.013380000
1	-1.660781000	8.205939000	0.461368000
1	-1.272070000	5.775280000	0.161776000
1	5.992387000	3.071758000	-1.530258000
1	7.294220000	2.885739000	-3.630494000
1	6.911272000	0.938409000	-5.159012000
1	5.210623000	-0.767597000	-4.580604000
1	4.775585000	-6.308552000	1.950343000
1	4.630655000	-6.837196000	4.385597000
1	3.578037000	-5.193753000	5.943561000
1	2.682427000	-3.047020000	5.067315000
1	-5.902021000	0.837179000	1.028615000
1	-4.463628000	1.837161000	0.717419000
1	3.192863000	-2.085384000	-3.898258000
1	4.732054000	-2.385053000	-3.040968000
7	1.715280000	-3.085435000	-1.803193000
19	-2.260560000	-1.121852000	-1.153534000
8	-0.705972000	-1.160306000	-3.568400000
8	-1.300730000	-3.777159000	-0.685597000
8	-3.336167000	-0.112525000	-3.613700000
8	-4.071690000	-3.140825000	-1.463884000
6	-0.069593000	-4.500548000	-0.643549000
1	-0.004424000	-5.185785000	-1.510038000
6	1.146313000	-3.602309000	-0.546917000
1	-0.078034000	-5.130255000	0.264690000
1	1.940206000	-4.183862000	-0.066607000
1	0.915134000	-2.758147000	0.117563000
6	-2.408951000	-4.651549000	-0.569005000
1	-2.511048000	-5.267847000	-1.482054000
1	-2.255060000	-5.339308000	0.283786000
6	-3.696588000	-3.902335000	-0.314205000
1	-4.480796000	-4.645918000	-0.095972000
1	-3.597040000	-3.248562000	0.572016000
6	-5.463899000	-2.861700000	-1.598068000
1	-5.688685000	-2.891691000	-2.672428000
1	-6.048231000	-3.667327000	-1.124712000
6	-5.906290000	-1.541000000	-0.979105000
1	-7.011925000	-1.533203000	-0.997028000
1	-5.593432000	-1.518497000	0.067067000
6	0.872717000	-2.873397000	-2.979128000
6	0.691546000	-1.427040000	-3.457232000
1	1.247362000	-3.494725000	-3.807372000
1	-0.122823000	-3.264903000	-2.746390000
1	1.146859000	-1.288812000	-4.454526000
1	1.158928000	-0.718811000	-2.763966000
6	-1.006222000	0.090919000	-4.175106000
1	-0.333307000	0.266477000	-5.033988000

6	-2.423763000	0.051277000	-4.689378000
1	-0.842912000	0.905862000	-3.450672000
1	-2.642970000	0.985214000	-5.239592000
1	-2.517173000	-0.791211000	-5.397075000
6	-5.695424000	-0.227063000	-3.092937000
1	-6.605666000	-0.800907000	-3.329212000
1	-5.903405000	0.829256000	-3.294359000
6	-4.571471000	-0.691642000	-4.009355000
1	-4.836880000	-0.374835000	-5.033252000
1	-4.456828000	-1.788196000	-4.026461000
8	-2.471005000	-3.418530000	-3.863706000
1	-1.897289000	-2.631545000	-3.819124000
1	-3.014045000	-3.383248000	-3.056931000
6	0.606417000	0.412200000	-0.454927000
8	0.212517000	-0.748031000	-0.493623000
8	0.582220000	1.183367000	-1.492738000
6	-2.120759000	2.647613000	-1.168419000
1	-2.138985000	1.861235000	-1.938701000
1	-3.145489000	3.009098000	-1.039587000
1	-1.759824000	2.200163000	-0.232154000
6	-1.203168000	3.786428000	-1.612478000
8	-1.702887000	4.892899000	-1.870762000
8	0.051327000	3.531545000	-1.699267000
1	0.451681000	2.266170000	-1.430073000

^{5}IM1$_{acetate,K+}$:

6	-1.798629000	5.781413000	1.048354000
6	-0.548055000	5.472821000	1.610863000
6	0.204176000	6.516259000	2.179358000
6	-0.281310000	7.826346000	2.194930000
6	-1.530945000	8.118104000	1.638719000
6	-2.286054000	7.090317000	1.064150000
6	-0.008781000	4.081372000	1.599778000
6	-0.749656000	3.072826000	2.257243000
7	-0.403627000	1.747055000	2.313023000
6	-1.409293000	1.071869000	2.952881000
6	-2.450167000	2.013539000	3.330729000
6	-2.030380000	3.252685000	2.927523000
26	1.279237000	0.809519000	1.482819000
7	1.910052000	2.698850000	0.844040000
6	2.892778000	2.836081000	-0.104148000
6	2.798567000	4.154821000	-0.695293000
6	1.779126000	4.806271000	-0.053982000
6	1.193015000	3.865278000	0.879951000
6	3.879954000	1.864600000	-0.391976000
6	4.692366000	1.990265000	-1.636505000
6	4.492052000	1.026248000	-2.661952000
6	5.235117000	1.087703000	-3.846839000
6	6.181896000	2.103020000	-4.025529000
6	6.388986000	3.059313000	-3.030834000
6	5.642662000	2.994915000	-1.848437000
6	-1.394767000	-0.300416000	3.273541000
6	-2.663215000	-0.942146000	3.734143000
6	-2.783503000	-1.386239000	5.064368000
6	-3.913872000	-2.064954000	5.517770000
6	-4.959307000	-2.333738000	4.629135000
6	-4.868444000	-1.901744000	3.306518000
6	-3.739701000	-1.199995000	2.861947000
8	-3.696703000	-0.782974000	1.537854000
6	-4.634984000	0.252774000	1.256688000
6	-4.914650000	0.542260000	-0.215909000
7	-5.168441000	-0.479569000	-1.109808000
8	3.535617000	0.083211000	-2.434186000
6	3.537872000	-1.137996000	-3.156924000
6	2.878386000	-2.182955000	-2.243433000
8	3.478684000	-2.498197000	-1.218488000
7	1.019779000	-0.749000000	2.893046000
6	-0.248773000	-1.133913000	3.247525000
6	-0.236729000	-2.536517000	3.610859000
6	1.055404000	-2.972607000	3.473802000
6	1.831586000	-1.848082000	2.992693000
6	3.183175000	-1.939871000	2.564539000
6	3.897593000	-3.221247000	2.834507000
6	4.330302000	-4.027304000	1.764481000
6	4.980739000	-5.239692000	2.008280000
6	5.209977000	-5.667365000	3.320766000
6	4.780700000	-4.875051000	4.390573000
6	4.127533000	-3.663496000	4.148385000
7	3.253402000	0.152313000	1.273518000
6	3.842640000	-0.978585000	1.777106000
6	5.194756000	-1.087018000	1.243792000
6	5.350357000	-0.066455000	0.351429000
6	4.120520000	0.708653000	0.381221000
8	-4.984868000	1.723029000	-0.531067000
1	4.136019000	-3.692610000	0.742987000
1	-1.097697000	-3.120408000	3.925072000
1	1.435658000	-3.975848000	3.648436000
1	-3.353107000	1.765840000	3.885349000
1	-2.540682000	4.200922000	3.075209000
1	1.410636000	5.809379000	-0.250044000
1	3.431682000	4.539522000	-1.490891000
1	6.223241000	0.165012000	-0.255459000
1	5.918038000	-1.855260000	1.504721000
1	-1.956247000	-1.187848000	5.748780000
1	-3.975694000	-2.389532000	6.558695000
1	-5.844820000	-2.879878000	4.961708000
1	-5.678891000	-2.109730000	2.605288000
1	1.178519000	6.290004000	2.618158000
1	0.317183000	8.621373000	2.646752000
1	-1.912753000	9.141899000	1.649596000
1	-3.258079000	7.310508000	0.615545000
1	-2.374897000	4.988372000	0.574035000
1	5.810419000	3.727774000	-1.056297000
1	7.130858000	3.848831000	-3.167908000
1	6.755793000	2.140820000	-4.954525000
1	5.069929000	0.356706000	-4.639002000
1	5.305588000	-5.858204000	1.167572000
1	5.719240000	-6.615624000	3.509464000
1	4.957295000	-5.200368000	5.418877000
1	3.793762000	-3.045498000	4.985048000
1	-5.608811000	0.027474000	1.727006000
1	-4.270101000	1.201396000	1.673292000
1	3.064502000	-1.012979000	-4.141190000
1	4.570754000	-1.486974000	-3.317231000
7	1.681579000	-2.737352000	-2.574147000
19	-2.108050000	-1.338687000	-1.472594000
8	-0.873337000	-0.899561000	-4.062707000
8	-1.209482000	-4.000305000	-1.834480000
8	-3.505331000	0.104947000	-3.414779000
8	-4.060403000	-3.255606000	-1.993911000
6	0.053561000	-4.627876000	-2.016766000
1	0.159082000	-4.991760000	-3.056376000
6	1.203682000	-3.738680000	-1.601276000
1	0.095546000	-5.520612000	-1.366125000
1	2.055152000	-4.395635000	-1.370762000
1	0.933674000	-3.212730000	-0.673586000
6	-2.284246000	-4.907879000	-2.000516000
1	-2.471877000	-5.089784000	-3.073922000
1	-2.033340000	-5.876596000	-1.529981000
6	-3.537879000	-4.404671000	-1.324564000
1	-4.285664000	-5.215575000	-1.352969000
1	-3.328257000	-4.169778000	-0.264967000
6	-5.410380000	-2.936365000	-1.657491000
1	-5.931393000	-2.692749000	-2.592981000

1	-5.907225000	-3.827108000	-1.241889000
6	-5.558126000	-1.816816000	-0.633965000
1	-6.622867000	-1.803866000	-0.333510000
1	-4.964744000	-2.061927000	0.253332000
6	0.966083000	-2.450592000	-3.818223000
6	0.548110000	-0.987741000	-4.019826000
1	1.559477000	-2.796344000	-4.679192000
1	0.048785000	-3.048436000	-3.823184000
1	0.949190000	-0.603263000	-4.975293000
1	0.917851000	-0.358868000	-3.197407000
6	-1.324733000	0.413424000	-4.383669000
1	-0.834766000	0.755404000	-5.314764000
6	-2.813643000	0.387828000	-4.621627000
1	-1.046371000	1.113489000	-3.577790000
1	-3.132416000	1.368690000	-5.021163000
1	-3.044781000	-0.381657000	-5.378657000
6	-5.698704000	-0.051631000	-2.411839000
1	-6.689846000	-0.508350000	-2.568548000
1	-5.838142000	1.032233000	-2.346016000
6	-4.830085000	-0.352802000	-3.624479000
1	-5.289587000	0.171260000	-4.481959000
1	-4.803140000	-1.424788000	-3.880401000
8	-2.751471000	-2.976462000	-4.609884000
1	-2.070806000	-2.297645000	-4.443843000
1	-3.241517000	-3.052678000	-3.773295000
6	0.350917000	-0.184298000	-0.102291000
8	0.034527000	-1.379241000	0.066802000
8	-0.073551000	0.321183000	-1.288547000
6	-2.103711000	2.724954000	-1.095149000
1	-2.550063000	1.963947000	-1.753373000
1	-2.896970000	3.431487000	-0.823202000
1	-1.750525000	2.205478000	-0.193357000
6	-0.949229000	3.422658000	-1.820838000
8	-1.079965000	4.608990000	-2.166818000
8	0.080419000	2.698732000	-2.059946000
1	0.072861000	1.331328000	-1.469875000

[5]RC2 $_{acetate,K+}$:

6	4.159993000	2.733223000	-2.394905000
6	2.974205000	3.224988000	-2.973399000
6	3.022840000	4.441786000	-3.675092000
6	4.222432000	5.147134000	-3.798380000
6	5.394635000	4.647977000	-3.221197000
6	5.357968000	3.439002000	-2.519356000
6	1.695690000	2.478870000	-2.802993000
6	1.680572000	1.119102000	-3.190398000
7	0.691948000	0.220837000	-2.881837000
6	1.136592000	-1.029110000	-3.219040000
6	2.435209000	-0.914782000	-3.860492000
6	2.764246000	0.412772000	-3.859652000
26	-1.127999000	0.580578000	-1.936159000
7	-0.603775000	2.618971000	-1.872118000
6	-1.262865000	3.468551000	-1.016628000
6	-0.405911000	4.600613000	-0.728713000
6	0.753111000	4.420093000	-1.445631000
6	0.635179000	3.145399000	-2.131300000
6	-2.575207000	3.279726000	-0.519609000
6	-3.066498000	4.174659000	0.568731000
6	-3.296237000	3.614324000	1.854159000
6	-3.767637000	4.416132000	2.900526000
6	-4.011083000	5.776991000	2.682699000
6	-3.786348000	6.344260000	1.428053000
6	-3.316196000	5.539072000	0.384756000
6	0.459757000	-2.240343000	-2.962243000
6	1.253643000	-3.503438000	-3.022300000
6	0.994171000	-4.472456000	-4.007607000
6	1.682990000	-5.684809000	-4.044399000
6	2.647768000	-5.961005000	-3.071090000
6	2.923826000	-5.016900000	-2.081810000
6	2.243482000	-3.793484000	-2.059793000
8	2.533130000	-2.880811000	-1.054056000
6	3.841676000	-2.318117000	-1.160770000
6	4.200427000	-1.410290000	0.015910000
7	4.439752000	-1.974364000	1.247129000
8	-3.013951000	2.289264000	2.003310000
6	-3.542890000	1.569657000	3.107965000
6	-3.495912000	0.082668000	2.730097000
8	-4.348178000	-0.343693000	1.953019000
7	-1.795341000	-1.317790000	-2.509542000
6	-0.911893000	-2.361271000	-2.636620000
6	-1.619367000	-3.608884000	-2.421620000
6	-2.932048000	-3.287733000	-2.193526000
6	-3.027312000	-1.841321000	-2.224766000
6	-4.189520000	-1.104393000	-1.876559000
6	-5.452270000	-1.879084000	-1.696333000
6	-6.060208000	-1.966779000	-0.430317000
6	-7.231043000	-2.710375000	-0.259451000
6	-7.811549000	-3.375988000	-1.344568000
6	-7.210547000	-3.298451000	-2.605115000
6	-6.037952000	-2.558227000	-2.778095000
7	-3.093218000	1.070320000	-1.503673000
6	-4.203582000	0.267335000	-1.556548000
6	-5.350793000	1.015147000	-1.058741000
6	-4.879601000	2.217630000	-0.618549000
6	-3.451425000	2.237785000	-0.892392000
8	4.343267000	-0.209545000	-0.192156000
1	-5.593436000	-1.460157000	0.417036000
1	-1.178736000	-4.601923000	-2.444203000
1	-3.752564000	-3.970231000	-1.987055000
1	3.001113000	-1.740747000	-4.286080000
1	3.658282000	0.870900000	-4.274066000
1	1.621843000	5.073304000	-1.463560000
1	-0.652789000	5.435878000	-0.078476000
1	-5.442032000	3.028762000	-0.161562000
1	-6.374856000	0.652947000	-1.026252000
1	0.232495000	-4.254437000	-4.758862000
1	1.463834000	-6.413916000	-4.827268000
1	3.185069000	-6.911877000	-3.077110000
1	3.669673000	-5.230184000	-1.313744000
1	2.110586000	4.831498000	-4.131816000
1	4.242434000	6.089223000	-4.351676000
1	6.332033000	5.201211000	-3.315750000
1	6.265902000	3.047054000	-2.054522000
1	4.132623000	1.801978000	-1.825290000
1	-3.152960000	5.968663000	-0.606015000
1	-3.980992000	7.404895000	1.256617000
1	-4.378259000	6.391897000	3.507761000
1	-3.935111000	3.990630000	3.890102000
1	-7.689991000	-2.775881000	0.730424000
1	-8.727489000	-3.956054000	-1.207964000
1	-7.657217000	-3.814921000	-3.458333000
1	-5.569506000	-2.497767000	-3.763110000
1	4.602134000	-3.112328000	-1.246456000
1	3.895925000	-1.681595000	-2.052241000
1	-3.005367000	1.816999000	4.035320000
1	-4.606931000	1.815993000	3.248909000
7	-2.532036000	-0.716966000	3.255146000
19	1.424007000	-1.387752000	1.873670000
8	0.757015000	0.513372000	3.868762000
8	-0.373556000	-3.136786000	3.142083000
8	3.462696000	0.162985000	3.011621000
8	2.593300000	-3.482424000	3.196846000
6	-1.740432000	-3.126014000	3.546902000
1	-1.814892000	-2.982058000	4.641238000

6	-2.572561000	-2.113832000	2.787453000
1	-2.173630000	-4.118908000	3.328517000
1	-3.619861000	-2.442382000	2.850866000
1	-2.290569000	-2.133478000	1.725190000
6	0.337304000	-4.220678000	3.720419000
1	0.548938000	-4.015422000	4.785164000
1	-0.276559000	-5.138524000	3.666545000
6	1.622812000	-4.510648000	2.978534000
1	2.019491000	-5.468484000	3.355295000
1	1.419585000	-4.627358000	1.898531000
6	3.935801000	-3.838238000	2.867091000
1	4.584338000	-3.387275000	3.629703000
1	4.060460000	-4.929894000	2.947427000
6	4.367154000	-3.425581000	1.461839000
1	5.356718000	-3.883217000	1.278939000
1	3.661203000	-3.852603000	0.741435000
6	-1.496625000	-0.244738000	4.171229000
6	-0.598471000	0.875758000	3.628349000
1	-1.945481000	0.053044000	5.131870000
1	-0.841529000	-1.092500000	4.393762000
1	-0.803235000	1.832870000	4.138519000
1	-0.762530000	1.017941000	2.551075000
6	1.671296000	1.589470000	3.684267000
1	1.416013000	2.419218000	4.368797000
6	3.061553000	1.100314000	3.997173000
1	1.618695000	1.957908000	2.647124000
1	3.757068000	1.959424000	4.014792000
1	3.074294000	0.631930000	4.997130000
6	5.272553000	-1.192304000	2.173288000
1	6.098544000	-1.835084000	2.518701000
1	5.706716000	-0.370972000	1.591509000
6	4.587269000	-0.602258000	3.396290000
1	5.334605000	0.033908000	3.905156000
1	4.272096000	-1.372275000	4.121493000
8	1.776956000	-1.712160000	5.369808000
1	1.388854000	-0.951289000	4.900815000
1	2.117980000	-2.300298000	4.674001000
6	-0.437750000	0.105529000	-0.036414000
8	-0.856223000	-0.765953000	0.722972000
8	0.711486000	0.740132000	0.468326000
6	4.764338000	2.904938000	1.134074000
1	4.791274000	2.959563000	2.234322000
1	5.711303000	3.297373000	0.744670000
1	4.619967000	1.854056000	0.850925000
6	3.629399000	3.759551000	0.645132000
8	3.717979000	4.911089000	0.281124000
8	2.451614000	3.107343000	0.681054000
1	1.049534000	1.370372000	-0.185420000
1	1.752197000	3.703740000	0.338971000

[5]TS2 $_{\text{acetate,K+}}$:

6	1.319428000	5.736948000	0.787706000
6	0.285733000	5.726100000	-0.165112000
6	-0.330897000	6.941051000	-0.505075000
6	0.079536000	8.138781000	0.086841000
6	1.111601000	8.138672000	1.030562000
6	1.729603000	6.933374000	1.380500000
6	-0.154918000	4.437465000	-0.776474000
6	0.784473000	3.738687000	-1.567857000
7	0.613345000	2.475431000	-2.079726000
6	1.799317000	2.089466000	-2.652329000
6	2.752756000	3.174850000	-2.544394000
6	2.121123000	4.199203000	-1.888997000
26	-1.033407000	1.206982000	-1.840432000
7	-1.993804000	2.786927000	-0.864844000
6	-3.176106000	2.613252000	-0.192255000
6	-3.385628000	3.735915000	0.704166000
6	-2.323940000	4.579789000	0.537506000
6	-1.436793000	3.960610000	-0.431825000
6	-4.064533000	1.530766000	-0.352932000
6	-5.130135000	1.362424000	0.681741000
6	-4.884904000	0.425995000	1.718292000
6	-5.817381000	0.257469000	2.749089000
6	-7.006695000	0.995974000	2.737849000
6	-7.266909000	1.910568000	1.716735000
6	-6.321302000	2.090949000	0.699670000
6	2.075642000	0.822619000	-3.211940000
6	3.516786000	0.522541000	-3.456120000
6	4.051556000	0.448913000	-4.750312000
6	5.414015000	0.224177000	-4.959343000
6	6.264226000	0.067809000	-3.861254000
6	5.750905000	0.119190000	-2.563140000
6	4.382730000	0.337238000	-2.359705000
8	3.834335000	0.301200000	-1.094111000
6	4.308441000	1.234523000	-0.133421000
6	4.266978000	0.799993000	1.339834000
7	4.754303000	-0.423253000	1.742509000
8	-3.696545000	-0.227360000	1.647881000
6	-3.411268000	-1.362358000	2.433920000
6	-2.672278000	-2.371168000	1.542280000
8	-2.940521000	-2.412600000	0.352082000
7	-0.222369000	-0.101299000	-3.291570000
6	1.133884000	-0.185531000	-3.497164000
6	1.456750000	-1.498169000	-4.024023000
6	0.278652000	-2.187956000	-4.120165000
6	-0.772021000	-1.305971000	-3.639422000
6	-2.123022000	-1.687928000	-3.449727000
6	-2.505417000	-3.070336000	-3.862958000
6	-2.882244000	-4.007310000	-2.882047000
6	-3.211128000	-5.316270000	-3.242177000
6	-3.171516000	-5.709178000	-4.584634000
6	-2.799154000	-4.784724000	-5.565828000
6	-2.466125000	-3.475493000	-5.206826000
7	-2.851752000	0.235400000	-2.079803000
6	-3.091516000	-0.932825000	-2.755505000
6	-4.459441000	-1.354621000	-2.498880000
6	-4.979116000	-0.483518000	-1.584663000
6	-3.952094000	0.502389000	-1.311107000
8	3.890852000	1.643718000	2.146693000
1	-2.906958000	-3.694685000	-1.834963000
1	2.453742000	-1.848309000	-4.279478000
1	0.138205000	-3.209870000	-4.462154000
1	3.767633000	3.163084000	-2.934411000
1	2.528448000	5.175906000	-1.641297000
1	-2.139896000	5.521056000	1.048760000
1	-4.240237000	3.863710000	1.363533000
1	-5.969283000	-0.496490000	-1.134953000
1	-4.946002000	-2.219909000	-2.941098000
1	3.381020000	0.590517000	-5.600684000
1	5.811505000	0.179596000	-5.975428000
1	7.332304000	-0.105394000	-4.010476000
1	6.416911000	-0.020365000	-1.710475000
1	-1.135461000	6.942498000	-1.243842000
1	-0.407232000	9.076330000	-0.192613000
1	1.432192000	9.074871000	1.493711000
1	2.531468000	6.923801000	2.122787000
1	1.791673000	4.793209000	1.070410000
1	-6.506428000	2.809190000	-0.102342000
1	-8.194967000	2.485780000	1.711621000
1	-7.728132000	0.854458000	3.546136000
1	-5.618779000	-0.432228000	3.569725000
1	-3.495451000	-6.035059000	-2.469537000
1	-3.429331000	-6.733215000	-4.865375000
1	-2.769748000	-5.083079000	-6.616654000

1	-2.176264000	-2.754432000	-5.974445000
1	5.354351000	1.516234000	-0.348392000
1	3.698870000	2.146757000	-0.183279000
1	-2.832780000	-1.056937000	3.311782000
1	-4.331547000	-1.865603000	2.777181000
7	-1.780273000	-3.233197000	2.138516000
19	1.447310000	-1.176305000	2.108932000
8	0.402343000	-2.550508000	4.385634000
8	1.048405000	-3.696051000	1.090255000
8	3.057095000	-1.188204000	4.236249000
8	3.609046000	-3.048412000	1.051671000
6	0.149173000	-4.714488000	1.489409000
1	0.370478000	-4.947623000	2.541270000
6	-1.303404000	-4.331814000	1.288793000
1	0.339428000	-5.637901000	0.916450000
1	-1.923572000	-5.230701000	1.468294000
1	-1.462131000	-4.027375000	0.249613000
6	1.732852000	-3.904414000	-0.129895000
1	2.102529000	-4.944937000	-0.173103000
1	1.077282000	-3.729817000	-1.003087000
6	2.910949000	-2.949816000	-0.185755000
1	3.562509000	-3.235202000	-1.029509000
1	2.583770000	-1.911659000	-0.368887000
6	5.004222000	-2.819414000	1.022625000
1	5.393452000	-3.200313000	1.975973000
1	5.476870000	-3.412313000	0.218079000
6	5.385840000	-1.356974000	0.809477000
1	6.486278000	-1.281358000	0.885449000
1	5.100046000	-1.077095000	-0.203638000
6	-1.763158000	-3.475187000	3.589521000
6	-1.020267000	-2.512380000	4.506341000
1	-2.796890000	-3.517408000	3.971913000
1	-1.355524000	-4.483202000	3.744073000
1	-1.293291000	-2.803427000	5.538199000
1	-1.341836000	-1.473864000	4.350850000
6	0.987965000	-1.694585000	5.371276000
1	0.616964000	-1.984043000	6.371461000
6	2.493363000	-1.794889000	5.389453000
1	0.673828000	-0.652223000	5.183241000
1	2.857880000	-1.280070000	6.298621000
1	2.798725000	-2.852106000	5.450642000
6	5.146364000	-0.542852000	3.155855000
1	6.215380000	-0.816344000	3.179273000
1	5.040638000	0.453932000	3.599891000
6	4.418949000	-1.539378000	4.046980000
1	4.947563000	-1.514395000	5.018283000
1	4.470477000	-2.573656000	3.674583000
8	2.601955000	-4.348515000	3.502995000
1	1.793003000	-3.880979000	3.772584000
1	2.770803000	-4.011852000	2.603965000
6	-0.254129000	-0.051603000	-0.382980000
8	-0.179960000	-1.163346000	-0.041212000
8	1.013279000	0.943552000	0.584591000
6	-2.142033000	2.063385000	3.737700000
1	-1.947441000	3.124875000	3.942564000
1	-2.320690000	1.513077000	4.669917000
1	-3.046232000	2.008382000	3.112127000
6	-1.002778000	1.445374000	2.963439000
8	-0.645466000	0.278739000	3.153834000
8	-0.463789000	2.225772000	2.075177000
1	1.272986000	1.559899000	-0.115966000
1	0.222846000	1.670739000	1.427239000

^{5}IM2 $_{\text{acetate,K+}}$:

6	0.738269000	5.490940000	-1.768703000
6	-0.261610000	4.960241000	-2.602020000
6	-0.928248000	5.811875000	-3.495848000
6	-0.598245000	7.169106000	-3.562801000
6	0.400713000	7.689918000	-2.734337000
6	1.066460000	6.847285000	-1.837014000
6	-0.605500000	3.509042000	-2.510417000
6	0.372757000	2.585713000	-2.940981000
7	0.278835000	1.216492000	-2.877744000
6	1.463330000	0.693445000	-3.331928000
6	2.343172000	1.774697000	-3.716200000
6	1.661927000	2.943241000	-3.491168000
26	-1.252336000	0.126376000	-2.012177000
7	-2.289150000	1.897362000	-1.687368000
6	-3.455421000	1.991877000	-0.984325000
6	-3.779763000	3.390388000	-0.772725000
6	-2.789341000	4.120818000	-1.366390000
6	-1.838244000	3.172114000	-1.923128000
6	-4.233189000	0.907413000	-0.530538000
6	-5.214369000	1.183462000	0.559074000
6	-4.702765000	1.489313000	1.843357000
6	-5.569491000	1.630037000	2.932648000
6	-6.949164000	1.494049000	2.750187000
6	-7.470866000	1.224967000	1.482565000
6	-6.600198000	1.069375000	0.399092000
6	1.782124000	-0.679800000	-3.432828000
6	3.198888000	-1.020755000	-3.760920000
6	3.563562000	-1.548506000	-5.008382000
6	4.891973000	-1.863632000	-5.301691000
6	5.881044000	-1.661605000	-4.334728000
6	5.541242000	-1.147474000	-3.081598000
6	4.209791000	-0.823300000	-2.800956000
8	3.834630000	-0.372944000	-1.551545000
6	4.490275000	0.777030000	-1.028669000
6	4.594393000	0.926214000	0.496381000
7	4.950488000	-0.111926000	1.322824000
8	-3.357610000	1.699891000	1.940799000
6	-2.609987000	1.228702000	3.038116000
6	-2.418089000	-0.287034000	2.947160000
8	-2.948731000	-0.907772000	2.037406000
7	-0.430921000	-1.623046000	-2.859420000
6	0.884476000	-1.745575000	-3.233828000
6	1.207286000	-3.154036000	-3.387906000
6	0.067368000	-3.856104000	-3.112945000
6	-0.955262000	-2.884502000	-2.761583000
6	-2.243751000	-3.209505000	-2.286005000
6	-2.589521000	-4.664328000	-2.223871000
6	-2.610630000	-5.340622000	-0.992941000
6	-2.914985000	-6.702989000	-0.933745000
6	-3.201209000	-7.409855000	-2.106422000
6	-3.179204000	-6.746671000	-3.337315000
6	-2.874213000	-5.383618000	-3.394965000
7	-3.034082000	-0.928110000	-1.692916000
6	-3.183883000	-2.293649000	-1.768430000
6	-4.411188000	-2.670032000	-1.094750000
6	-4.932212000	-1.531666000	-0.544575000
6	-4.063133000	-0.436996000	-0.922400000
8	4.513869000	2.078357000	0.910342000
1	-2.379975000	-4.789203000	-0.078486000
1	2.178682000	-3.550981000	-3.671079000
1	-0.068065000	-4.934415000	-3.128659000
1	3.340721000	1.657404000	-4.132632000
1	2.005880000	3.956177000	-3.681714000
1	-2.692863000	5.202408000	-1.406850000
1	-4.657683000	3.756252000	-0.245022000
1	-5.824669000	-1.443544000	0.068021000
1	-4.806136000	-3.679806000	-1.022866000
1	2.784527000	-1.700834000	-5.758303000
1	5.154060000	-2.265711000	-6.282476000
1	6.922050000	-1.912962000	-4.549316000

1	6.308627000	-1.009756000	-2.318065000
1	-1.707029000	5.404415000	-4.144717000
1	-1.121848000	7.820833000	-4.266548000
1	0.658451000	8.750550000	-2.786462000
1	1.842126000	7.251208000	-1.181329000
1	1.223853000	4.818595000	-1.058399000
1	-6.996141000	0.840757000	-0.592917000
1	-8.548947000	1.130220000	1.336894000
1	-7.617316000	1.611113000	3.606752000
1	-5.170860000	1.857194000	3.922598000
1	-2.924486000	-7.215183000	0.031433000
1	-3.438936000	-8.475296000	-2.061020000
1	-3.401779000	-7.291951000	-4.257705000
1	-2.856717000	-4.866079000	-4.356858000
1	5.527043000	0.831541000	-1.400803000
1	3.961225000	1.677222000	-1.372042000
1	-1.625033000	1.711753000	2.944277000
1	-3.051212000	1.515071000	4.007510000
7	-1.605534000	-0.889321000	3.885315000
19	1.684506000	-0.052809000	2.475263000
8	1.031657000	0.434043000	5.135558000
8	0.746823000	-2.536328000	3.001158000
8	3.747252000	0.789980000	3.983104000
8	3.369102000	-2.483768000	2.265179000
6	-0.156915000	-2.944858000	4.017589000
1	0.281924000	-2.661600000	4.986769000
6	-1.541862000	-2.355349000	3.828452000
1	-0.257650000	-4.042738000	4.024926000
1	-2.204306000	-2.785060000	4.603184000
1	-1.940930000	-2.650578000	2.853100000
6	1.243933000	-3.556585000	2.153717000
1	1.597890000	-4.409605000	2.760319000
1	0.462223000	-3.918088000	1.461784000
6	2.404646000	-3.002101000	1.353211000
1	2.838363000	-3.818565000	0.750776000
1	2.069051000	-2.215309000	0.655543000
6	4.724331000	-2.554398000	1.854912000
1	5.325365000	-2.489864000	2.771569000
1	4.932868000	-3.539886000	1.401263000
6	5.139451000	-1.484219000	0.848311000
1	6.204945000	-1.661016000	0.607077000
1	4.565900000	-1.613253000	-0.072474000
6	-1.329811000	-0.273678000	5.192177000
6	-0.321226000	0.862744000	5.282218000
1	-2.269106000	0.107194000	5.628298000
1	-0.993341000	-1.074928000	5.863632000
1	-0.443606000	1.306082000	6.287878000
1	-0.516069000	1.654119000	4.544306000
6	1.923848000	1.526368000	5.370622000
1	1.727189000	1.949086000	6.372616000
6	3.365506000	1.083355000	5.317923000
1	1.737231000	2.317599000	4.622034000
1	3.993390000	1.901872000	5.717905000
1	3.507840000	0.199468000	5.961525000
6	5.648669000	0.264588000	2.562138000
1	6.597076000	-0.298639000	2.593477000
1	5.889807000	1.330682000	2.478555000
6	4.951294000	0.045406000	3.896517000
1	5.662770000	0.387621000	4.670691000
1	4.731040000	-1.012731000	4.102852000
8	2.686470000	-2.052271000	5.214759000
1	2.037312000	-1.328951000	5.251827000
1	2.722189000	-2.291366000	4.272459000
6	-0.743646000	-0.741398000	-0.149008000
8	-0.574578000	-1.650287000	0.512115000
8	1.602071000	0.344850000	-0.070112000
6	-0.658603000	4.207482000	1.478184000
1	-0.598409000	4.623033000	2.495099000
1	-1.691264000	3.857393000	1.319998000
1	-0.444856000	4.982496000	0.731276000
6	0.269203000	3.004773000	1.336350000
8	0.323202000	2.201002000	2.310112000
8	0.894859000	2.862795000	0.249215000
1	2.043095000	0.136277000	-0.905774000
1	1.364949000	1.322492000	-0.060157000

[3]RC1 acetate, 6W, K+:

6	-3.067310000	5.454147000	1.245680000
6	-1.663813000	5.406007000	1.369165000
6	-0.974236000	6.621543000	1.546622000
6	-1.656244000	7.838668000	1.599711000
6	-3.048576000	7.871945000	1.472309000
6	-3.748369000	6.674709000	1.295034000
6	-0.894397000	4.132026000	1.306465000
6	-1.282693000	3.039829000	2.093069000
7	-0.627901000	1.803895000	2.117655000
6	-1.405654000	0.961799000	2.865432000
6	-2.581018000	1.659445000	3.328933000
6	-2.470261000	2.957507000	2.902074000
26	1.176569000	1.394316000	1.429742000
7	1.169579000	3.083758000	0.381277000
6	1.920341000	3.292878000	-0.758574000
6	1.375061000	4.398421000	-1.495240000
6	0.331793000	4.905159000	-0.750093000
6	0.187390000	4.066011000	0.401985000
6	3.082760000	2.563786000	-1.092407000
6	3.729047000	2.716736000	-2.419054000
6	3.879501000	1.581554000	-3.262388000
6	4.475371000	1.704074000	-4.523078000
6	4.937974000	2.949695000	-4.964880000
6	4.808567000	4.074427000	-4.150770000
6	4.211255000	3.945317000	-2.891897000
6	-1.040318000	-0.324927000	3.323819000
6	-2.111962000	-1.168557000	3.910684000
6	-2.109495000	-1.554314000	5.266211000
6	-3.130335000	-2.327296000	5.819958000
6	-4.201405000	-2.738405000	5.021175000
6	-4.238210000	-2.368583000	3.675427000
6	-3.212776000	-1.590487000	3.126548000
8	-3.236215000	-1.282721000	1.778808000
6	-4.329087000	-0.506001000	1.317836000
6	-4.855122000	-0.768531000	-0.090927000
7	-4.770688000	-1.984595000	-0.718781000
8	3.392157000	0.399327000	-2.788809000
6	3.751221000	-0.811101000	-3.430667000
6	3.556391000	-1.944709000	-2.416519000
8	4.362341000	-2.047216000	-1.493279000
7	1.370849000	0.003877000	2.859237000
6	0.329874000	-0.683684000	3.443781000
6	0.850671000	-1.760418000	4.238694000
6	2.229929000	-1.693443000	4.158008000
6	2.544596000	-0.622451000	3.264148000
6	3.818639000	-0.415804000	2.696464000
6	4.935886000	-1.270944000	3.194538000
6	5.443486000	-2.301050000	2.383469000
6	6.469854000	-3.128508000	2.845912000
6	7.002471000	-2.940058000	4.126063000
6	6.500494000	-1.920871000	4.941679000
6	5.471978000	-1.094147000	4.479297000
7	3.089872000	1.173450000	0.948147000
6	4.053323000	0.406986000	1.598943000
6	5.306731000	0.515078000	0.889419000
6	5.072764000	1.279879000	-0.219241000
6	3.686343000	1.689673000	-0.166266000

8	-5.443244000	0.190987000	-0.598825000
1	5.017811000	-2.449220000	1.388600000
1	0.260388000	-2.486643000	4.788758000
1	2.953897000	-2.348087000	4.636940000
1	-3.351687000	1.231279000	3.965459000
1	-3.150800000	3.775292000	3.115770000
1	-0.310061000	5.745796000	-1.001432000
1	1.733383000	4.751626000	-2.457456000
1	5.776015000	1.569422000	-0.996792000
1	6.239719000	0.047526000	1.193278000
1	-1.293244000	-1.207317000	5.901438000
1	-3.095950000	-2.597002000	6.877991000
1	-5.006450000	-3.346777000	5.439309000
1	-5.064793000	-2.694138000	3.041597000
1	0.112007000	6.602727000	1.651248000
1	-1.096601000	8.766205000	1.743494000
1	-3.583595000	8.823758000	1.509637000
1	-4.835570000	6.686233000	1.184476000
1	-3.626830000	4.532377000	1.087821000
1	4.127379000	4.817925000	-2.241196000
1	5.177550000	5.046687000	-4.484982000
1	5.399796000	3.029907000	-5.951933000
1	4.568428000	0.837058000	-5.177006000
1	6.852747000	-3.927221000	2.205673000
1	7.805325000	-3.587153000	4.487613000
1	6.912156000	-1.767314000	5.942303000
1	5.077881000	-0.299626000	5.117136000
1	-5.195616000	-0.616958000	1.992225000
1	-4.043910000	0.555284000	1.318327000
1	3.189256000	-0.941359000	-4.367517000
1	4.825941000	-0.813255000	-3.674012000
7	2.530609000	-2.820930000	-2.574856000
19	-1.441567000	-2.060690000	-1.048809000
8	-0.661443000	-1.816519000	-3.827414000
8	0.158971000	-4.574995000	-1.355226000
8	-3.422758000	-1.523986000	-3.228098000
8	-2.842811000	-4.468024000	-1.225011000
6	1.459983000	-4.986696000	-1.751989000
1	1.440905000	-5.384439000	-2.784922000
6	2.496483000	-3.901264000	-1.574013000
1	1.778725000	-5.823316000	-1.102751000
1	3.483593000	-4.390848000	-1.581634000
1	2.377059000	-3.471483000	-0.571751000
6	-0.724474000	-5.682882000	-1.285063000
1	-0.967122000	-6.038267000	-2.302171000
1	-0.228591000	-6.513586000	-0.747545000
6	-1.986226000	-5.375016000	-0.514156000
1	-2.517231000	-6.332516000	-0.375979000
1	-1.740095000	-4.949783000	0.470894000
6	-4.193841000	-4.464408000	-0.762108000
1	-4.847937000	-4.608441000	-1.633123000
1	-4.355137000	-5.332117000	-0.104024000
6	-4.599405000	-3.235476000	0.043957000
1	-5.562071000	-3.493474000	0.524019000
1	-3.864861000	-3.070574000	0.837555000
6	1.539391000	-2.732928000	-3.642709000
6	0.703971000	-1.449496000	-3.624404000
1	2.010476000	-2.884694000	-4.627029000
1	0.837968000	-3.560921000	-3.508244000
1	1.007087000	-0.741532000	-4.410493000
1	0.812188000	-0.928082000	-2.659980000
6	-1.465961000	-0.806472000	-4.417002000
1	-1.097302000	-0.574711000	-5.433507000
6	-2.883285000	-1.317701000	-4.519750000
1	-1.390944000	0.121509000	-3.829570000
1	-3.494723000	-0.586784000	-5.082894000
1	-2.878720000	-2.263279000	-5.088850000
6	-5.449600000	-2.073071000	-2.017486000
1	-6.233842000	-2.847046000	-1.969669000
1	-5.950932000	-1.112851000	-2.169284000
6	-4.561309000	-2.363135000	-3.222931000
1	-5.182179000	-2.182680000	-4.119854000
1	-4.235068000	-3.414707000	-3.269315000
8	-1.874895000	-4.382810000	-3.967735000
1	-1.387957000	-3.538144000	-3.955566000
1	-2.286622000	-4.424754000	-3.086133000
6	0.797870000	0.237412000	-0.082115000
8	0.921627000	-0.997893000	0.051103000
8	0.408991000	0.790006000	-1.147788000
6	-3.613611000	1.658777000	-2.750022000
1	-3.823969000	2.733914000	-2.791823000
1	-4.508578000	1.076542000	-2.516865000
1	-3.239672000	1.367147000	-3.742247000
6	-2.530697000	1.364944000	-1.747379000
8	-2.524897000	0.410235000	-0.992695000
8	-1.515112000	2.228945000	-1.787257000
1	-0.703444000	1.785043000	-1.324185000
8	-3.052231000	4.575196000	-1.677727000
1	-2.939365000	5.234945000	-0.977432000
1	-2.264290000	4.003299000	-1.606463000
8	-0.954745000	-3.163880000	1.492550000
1	-1.069276000	-2.303511000	1.922227000
1	0.027360000	-3.266807000	1.469803000
8	-4.769899000	2.884799000	0.028314000
1	-4.984415000	1.982873000	-0.279138000
1	-4.272859000	3.326627000	-0.683451000
8	1.772176000	-3.050413000	1.434267000
1	2.012700000	-2.808602000	2.343707000
1	1.579355000	-2.176604000	1.000908000
8	0.689679000	1.846320000	-3.739990000
1	0.987261000	1.405795000	-2.922343000
1	-0.091165000	2.322038000	-3.420104000

[3]TS1 $_{\text{acetate, 6W, K+}}$:

6	-3.229496000	5.462800000	1.033725000
6	-1.829239000	5.426299000	1.192271000
6	-1.152040000	6.647434000	1.379778000
6	-1.843795000	7.859397000	1.410988000
6	-3.233225000	7.881221000	1.250480000
6	-3.919834000	6.678752000	1.061252000
6	-1.048679000	4.157050000	1.160931000
6	-1.433692000	3.082110000	1.969846000
7	-0.752659000	1.863919000	2.051871000
6	-1.527052000	1.028125000	2.810979000
6	-2.730635000	1.712235000	3.219680000
6	-2.638573000	2.997357000	2.753527000
26	1.064916000	1.471524000	1.404410000
7	1.014750000	3.082525000	0.262718000
6	1.789184000	3.285166000	-0.862602000
6	1.266021000	4.392809000	-1.608819000
6	0.217735000	4.913098000	-0.879348000
6	0.042561000	4.078302000	0.269063000
6	2.973452000	2.568924000	-1.152776000
6	3.654217000	2.705924000	-2.464870000
6	3.866773000	1.555161000	-3.270556000
6	4.511641000	1.657340000	-4.508445000
6	4.961986000	2.901643000	-4.964895000
6	4.771337000	4.043745000	-4.187442000
6	4.126425000	3.933714000	-2.950890000
6	-1.139510000	-0.229009000	3.323262000
6	-2.202784000	-1.084060000	3.909141000
6	-2.226813000	-1.430268000	5.274597000
6	-3.242295000	-2.214601000	5.822680000
6	-4.279055000	-2.677249000	5.007065000

6	-4.288586000	-2.347253000	3.650536000
6	-3.269442000	-1.557204000	3.106776000
8	-3.260584000	-1.287941000	1.752342000
6	-4.363349000	-0.564177000	1.231380000
6	-4.825928000	-0.876743000	-0.189540000
7	-4.681447000	-2.107081000	-0.779099000
8	3.382903000	0.372317000	-2.788023000
6	3.862471000	-0.847057000	-3.331398000
6	3.674676000	-1.937477000	-2.269532000
8	4.458642000	-1.974517000	-1.322974000
7	1.270975000	0.149352000	2.890323000
6	0.238803000	-0.543536000	3.485629000
6	0.775496000	-1.565769000	4.336838000
6	2.154821000	-1.460001000	4.277554000
6	2.454273000	-0.423308000	3.341394000
6	3.731832000	-0.206227000	2.781630000
6	4.860487000	-1.017079000	3.326960000
6	5.380685000	-2.084773000	2.574845000
6	6.417049000	-2.871886000	3.083202000
6	6.946128000	-2.605154000	4.350810000
6	6.430984000	-1.548158000	5.107971000
6	5.392690000	-0.761453000	4.599767000
7	2.992112000	1.295198000	0.961854000
6	3.960621000	0.573278000	1.653863000
6	5.216102000	0.662689000	0.943427000
6	4.976757000	1.361285000	-0.205248000
6	3.583930000	1.754964000	-0.180750000
8	-5.430458000	0.048022000	-0.739799000
1	4.957459000	-2.294146000	1.590034000
1	0.196726000	-2.282742000	4.910651000
1	2.889010000	-2.067794000	4.800713000
1	-3.507088000	1.286516000	3.850658000
1	-3.342760000	3.805388000	2.922876000
1	-0.404569000	5.764768000	-1.141291000
1	1.645630000	4.745040000	-2.563015000
1	5.679676000	1.616087000	-0.994814000
1	6.153594000	0.223809000	1.274668000
1	-1.438332000	-1.043096000	5.921553000
1	-3.230458000	-2.453323000	6.888585000
1	-5.078804000	-3.295544000	5.420850000
1	-5.087990000	-2.713404000	3.004081000
1	-0.068462000	6.636555000	1.510424000
1	-1.294744000	8.791748000	1.563980000
1	-3.775858000	8.829247000	1.271055000
1	-5.004144000	6.682283000	0.925189000
1	-3.779450000	4.536509000	0.866790000
1	4.000027000	4.820308000	-2.326849000
1	5.131283000	5.015615000	-4.532288000
1	5.462739000	2.967728000	-5.933744000
1	4.652443000	0.774768000	-5.132497000
1	6.810732000	-3.700214000	2.488789000
1	7.756663000	-3.220895000	4.748185000
1	6.839797000	-1.333794000	6.098514000
1	4.987993000	0.062520000	5.191999000
1	-5.250058000	-0.693572000	1.875791000
1	-4.117453000	0.506372000	1.215025000
1	3.374854000	-1.067994000	-4.292677000
1	4.948243000	-0.786119000	-3.504086000
7	2.677604000	-2.848887000	-2.413704000
19	-1.312248000	-2.091362000	-1.051371000
8	-0.481989000	-1.989612000	-3.828489000
8	0.325139000	-4.602410000	-1.180155000
8	-3.257821000	-1.732877000	-3.276317000
8	-2.679701000	-4.556974000	-1.137603000
6	1.644636000	-5.003247000	-1.520143000
1	1.664516000	-5.451021000	-2.532402000
6	2.648707000	-3.884546000	-1.367645000
1	1.964198000	-5.798962000	-0.821768000
1	3.648272000	-4.346860000	-1.328893000
1	2.492347000	-3.413961000	-0.389520000
6	-0.536323000	-5.724214000	-1.073561000
1	-0.744747000	-6.138252000	-2.075976000
1	-0.037870000	-6.513912000	-0.479553000
6	-1.824119000	-5.401926000	-0.353997000
1	-2.338229000	-6.361794000	-0.174361000
1	-1.613792000	-4.916053000	0.611008000
6	-4.045080000	-4.570514000	-0.719578000
1	-4.666082000	-4.758373000	-1.606134000
1	-4.208063000	-5.420744000	-0.039384000
6	-4.504202000	-3.326069000	0.030827000
1	-5.474029000	-3.592257000	0.492430000
1	-3.796661000	-3.116081000	0.838177000
6	1.727594000	-2.849855000	-3.522223000
6	0.870342000	-1.584152000	-3.623053000
1	2.239046000	-3.057468000	-4.475880000
1	1.033850000	-3.679264000	-3.359464000
1	1.182365000	-0.936518000	-4.457333000
1	0.946331000	-0.989404000	-2.699506000
6	-1.299749000	-1.015478000	-4.460666000
1	-0.920440000	-0.808223000	-5.478625000
6	-2.704432000	-1.558318000	-4.566274000
1	-1.253037000	-0.068789000	-3.901246000
1	-3.321072000	-0.857211000	-5.160810000
1	-2.673683000	-2.521591000	-5.104565000
6	-5.313062000	-2.253620000	-2.095376000
1	-6.093968000	-3.031525000	-2.044307000
1	-5.815381000	-1.303988000	-2.301140000
6	-4.387161000	-2.583989000	-3.261707000
1	-4.986634000	-2.449681000	-4.181031000
1	-4.047987000	-3.632386000	-3.255744000
8	-1.645346000	-4.582296000	-3.850148000
1	-1.178702000	-3.726809000	-3.877561000
1	-2.074761000	-4.583622000	-2.975788000
6	0.813954000	0.201270000	-0.027238000
8	1.107772000	-0.992636000	0.126762000
8	0.345552000	0.612827000	-1.153147000
6	-3.489170000	1.471384000	-2.876126000
1	-3.719622000	2.540043000	-2.958699000
1	-4.387080000	0.880910000	-2.674414000
1	-3.062727000	1.161464000	-3.841265000
6	-2.449042000	1.225721000	-1.806351000
8	-2.522654000	0.294841000	-1.011279000
8	-1.423040000	2.047815000	-1.820941000
1	-0.502873000	1.462879000	-1.311072000
8	-2.949378000	4.358694000	-1.824915000
1	-2.796974000	5.032604000	-1.147397000
1	-2.187121000	3.747010000	-1.747961000
8	-0.900008000	-3.055319000	1.540245000
1	-1.044907000	-2.188420000	1.947232000
1	0.079477000	-3.166371000	1.588635000
8	-4.820106000	2.792938000	-0.197879000
1	-4.976700000	1.868124000	-0.469498000
1	-4.227188000	3.193718000	-0.860342000
8	1.839198000	-3.007663000	1.643759000
1	2.034649000	-2.730637000	2.553950000
1	1.696089000	-2.150373000	1.167168000
8	0.644895000	1.764317000	-3.797504000
1	1.122972000	1.310784000	-3.084223000
1	-0.137123000	2.090288000	-3.319449000

[3]IM1 $_{\text{acetate, 6W, K+}}$:

6	-2.747956000	5.648695000	1.136926000
6	-1.348350000	5.516850000	1.232439000
6	-0.579042000	6.688580000	1.372639000

6	-1.182665000	7.946474000	1.417183000
6	-2.573032000	8.063547000	1.318424000
6	-3.349795000	6.910227000	1.177459000
6	-0.661812000	4.195295000	1.181227000
6	-1.079489000	3.157331000	2.025075000
7	-0.480195000	1.895312000	2.086249000
6	-1.278160000	1.114680000	2.877755000
6	-2.410342000	1.879998000	3.334327000
6	-2.251071000	3.156803000	2.856672000
26	1.282184000	1.391932000	1.380184000
7	1.257720000	2.953545000	0.191866000
6	1.994620000	3.094374000	-0.968401000
6	1.520448000	4.235265000	-1.698941000
6	0.554070000	4.837526000	-0.925448000
6	0.368357000	4.025807000	0.238265000
6	3.119514000	2.308086000	-1.295030000
6	3.744150000	2.404040000	-2.640720000
6	3.783257000	1.265373000	-3.485654000
6	4.366957000	1.336783000	-4.756147000
6	4.928168000	2.538367000	-5.202609000
6	4.908129000	3.668593000	-4.385233000
6	4.321095000	3.589194000	-3.118067000
6	-0.956588000	-0.172403000	3.369896000
6	-2.054497000	-0.953114000	3.992991000
6	-2.039262000	-1.322712000	5.352561000
6	-3.088873000	-2.028250000	5.941823000
6	-4.202686000	-2.382452000	5.175662000
6	-4.253410000	-2.025162000	3.826794000
6	-3.198671000	-1.316898000	3.239564000
8	-3.237068000	-1.021314000	1.891944000
6	-4.309402000	-0.213142000	1.431413000
6	-4.889560000	-0.497223000	0.046972000
7	-4.891743000	-1.744397000	-0.530276000
8	3.196594000	0.128142000	-3.006688000
6	3.527847000	-1.122476000	-3.592491000
6	3.335239000	-2.201580000	-2.520689000
8	4.178852000	-2.297135000	-1.631900000
7	1.454621000	0.065899000	2.855275000
6	0.404630000	-0.568459000	3.484847000
6	0.914090000	-1.626300000	4.311382000
6	2.293354000	-1.596549000	4.205684000
6	2.619617000	-0.575561000	3.260848000
6	3.885700000	-0.432374000	2.659003000
6	4.983320000	-1.307656000	3.169616000
6	5.351319000	-2.455768000	2.448272000
6	6.358731000	-3.299144000	2.923124000
6	7.009401000	-3.007094000	4.127069000
6	6.644701000	-1.869660000	4.854239000
6	5.635175000	-1.026407000	4.378999000
7	3.179196000	1.112359000	0.863299000
6	4.122204000	0.325710000	1.516576000
6	5.334964000	0.293162000	0.733418000
6	5.083672000	0.978435000	-0.423289000
6	3.732505000	1.485572000	-0.330690000
8	-5.447580000	0.467963000	-0.479144000
1	4.829053000	-2.683910000	1.516763000
1	0.316722000	-2.312277000	4.903532000
1	3.010795000	-2.244710000	4.703212000
1	-3.187143000	1.509797000	3.998842000
1	-2.892760000	4.010612000	3.048230000
1	-0.016758000	5.730239000	-1.166276000
1	1.881746000	4.551008000	-2.672821000
1	5.754235000	1.150014000	-1.261964000
1	6.251052000	-0.215362000	1.022851000
1	-1.190866000	-1.013716000	5.964591000
1	-3.042858000	-2.287355000	7.002000000
1	-5.031768000	-2.936418000	5.621564000

1	-5.116397000	-2.305680000	3.220920000
1	0.505971000	6.602925000	1.455292000
1	-0.563787000	8.839721000	1.531973000
1	-3.046849000	9.047516000	1.349455000
1	-4.436246000	6.988359000	1.089317000
1	-3.367330000	4.761330000	1.006646000
1	4.321124000	4.463936000	-2.464795000
1	5.353645000	4.606203000	-4.724702000
1	5.379704000	2.581660000	-6.196584000
1	4.373167000	0.465435000	-5.411060000
1	6.634246000	-4.190111000	2.353437000
1	7.797542000	-3.666359000	4.498762000
1	7.148565000	-1.636498000	5.795479000
1	5.347614000	-0.138913000	4.947493000
1	-5.158501000	-0.260876000	2.134704000
1	-3.974877000	0.831537000	1.381924000
1	2.950082000	-1.288818000	-4.513439000
1	4.597845000	-1.149819000	-3.849203000
7	2.265830000	-3.036889000	-2.591650000
19	-1.626011000	-1.952904000	-1.033611000
8	-0.903318000	-1.979658000	-3.842972000
8	-0.124473000	-4.589485000	-1.184971000
8	-3.610011000	-1.531568000	-3.109279000
8	-3.116993000	-4.337160000	-0.960964000
6	1.134337000	-5.097284000	-1.601105000
1	1.054842000	-5.559047000	-2.604103000
6	2.228957000	-4.058453000	-1.532980000
1	1.437022000	-5.905696000	-0.909836000
1	3.190796000	-4.595749000	-1.552410000
1	2.175673000	-3.565738000	-0.555423000
6	-1.055150000	-5.640066000	-0.981029000
1	-1.361301000	-6.070285000	-1.951262000
1	-0.575324000	-6.444920000	-0.392017000
6	-2.264584000	-5.198331000	-0.192483000
1	-2.825731000	-6.110705000	0.072920000
1	-1.951347000	-4.682856000	0.728643000
6	-4.452552000	-4.247660000	-0.465346000
1	-5.134484000	-4.398611000	-1.313070000
1	-4.637014000	-5.076740000	0.235513000
6	-4.775692000	-2.963393000	0.288487000
1	-5.739890000	-3.146706000	0.799760000
1	-4.015214000	-2.801949000	1.057609000
6	1.256649000	-2.985453000	-3.645396000
6	0.481477000	-1.665940000	-3.720091000
1	1.697739000	-3.239014000	-4.622684000
1	0.517866000	-3.762565000	-3.430960000
1	0.789392000	-1.052666000	-4.581894000
1	0.653764000	-1.067216000	-2.812668000
6	-1.689806000	-0.944566000	-4.417631000
1	-1.363595000	-0.757622000	-5.457731000
6	-3.134113000	-1.382751000	-4.432677000
1	-1.540330000	-0.009572000	-3.857409000
1	-3.734269000	-0.629305000	-4.976977000
1	-3.211902000	-2.339726000	-4.978063000
6	-5.622747000	-1.850990000	-1.798321000
1	-6.464349000	-2.555053000	-1.680773000
1	-6.050581000	-0.862011000	-1.987258000
6	-4.808742000	-2.275675000	-3.017153000
1	-5.449085000	-2.092410000	-3.899440000
1	-4.566753000	-3.350823000	-3.019460000
8	-2.256181000	-4.481126000	-3.731673000
1	-1.734045000	-3.661584000	-3.806977000
1	-2.627165000	-4.438648000	-2.832065000
6	0.954995000	0.081841000	0.015269000
8	1.344640000	-1.077042000	0.079492000
8	0.292849000	0.432360000	-1.077299000
6	-3.480383000	1.688708000	-2.775110000

1	-3.851325000	2.720108000	-2.714017000
1	-4.301770000	0.973911000	-2.676219000
1	-3.014810000	1.587675000	-3.767046000
6	-2.423618000	1.443613000	-1.706144000
8	-2.548762000	0.515998000	-0.895136000
8	-1.397306000	2.227180000	-1.723170000
1	-0.241841000	1.293691000	-1.061271000
8	-2.729714000	4.571232000	-1.770165000
1	-2.441029000	5.184555000	-1.080472000
1	-2.069262000	3.834762000	-1.748220000
8	-0.951443000	-2.858585000	1.481426000
1	-1.000109000	-2.012334000	1.951639000
1	0.004289000	-3.094219000	1.524450000
8	-4.606214000	3.158170000	-0.014931000
1	-4.816670000	2.238965000	-0.269931000
1	-4.013331000	3.519482000	-0.701005000
8	1.793837000	-3.188315000	1.608045000
1	1.998416000	-2.934687000	2.522950000
1	1.780277000	-2.324736000	1.129799000
8	0.470519000	1.724440000	-3.747117000
1	1.053975000	1.213377000	-3.166078000
1	-0.212485000	2.040686000	-3.120319000

^{3}RC2$_{acetate, 6W, K+}$:

6	-2.157373000	5.064596000	2.446299000
6	-0.777769000	4.801645000	2.561237000
6	0.045490000	5.828035000	3.066102000
6	-0.483271000	7.065683000	3.435985000
6	-1.855101000	7.311655000	3.313694000
6	-2.687059000	6.302365000	2.821531000
6	-0.176006000	3.486645000	2.187947000
6	-0.679496000	2.309751000	2.738078000
7	-0.170390000	1.031095000	2.551013000
6	-1.043895000	0.176478000	3.210485000
6	-2.115880000	0.935857000	3.819604000
6	-1.862693000	2.246536000	3.579481000
26	1.569226000	0.524947000	1.704094000
7	1.760858000	2.393501000	1.037084000
6	2.699427000	2.835474000	0.112190000
6	2.460501000	4.192018000	-0.219863000
6	1.362964000	4.602410000	0.527567000
6	0.946593000	3.484922000	1.304556000
6	3.803487000	2.082098000	-0.357851000
6	4.600390000	2.641438000	-1.483495000
6	4.052632000	2.709579000	-2.780523000
6	4.802197000	3.204279000	-3.852396000
6	6.117197000	3.629939000	-3.647352000
6	6.677023000	3.579799000	-2.368377000
6	5.915840000	3.095347000	-1.301669000
6	-0.866253000	-1.174080000	3.451051000
6	-2.015236000	-1.926321000	4.032073000
6	-1.973727000	-2.474721000	5.325326000
6	-3.078101000	-3.125288000	5.878007000
6	-4.258664000	-3.245687000	5.137938000
6	-4.327050000	-2.716848000	3.847946000
6	-3.217522000	-2.060914000	3.306818000
8	-3.255555000	-1.594573000	2.008090000
6	-4.180161000	-0.577039000	1.660999000
6	-4.744035000	-0.539006000	0.238454000
7	-4.829884000	-1.612575000	-0.604293000
8	2.736785000	2.343172000	-2.941026000
6	2.331720000	1.511515000	-4.011143000
6	2.373329000	0.040049000	-3.576500000
8	3.254582000	-0.319074000	-2.813742000
7	1.505491000	-1.220863000	2.705404000
6	0.384115000	-1.814849000	3.265016000
6	0.701161000	-3.123872000	3.724641000
6	2.049932000	-3.322313000	3.468050000
6	2.520841000	-2.154266000	2.796474000
6	3.810695000	-2.037717000	2.219399000
6	4.730558000	-3.209019000	2.325772000
6	4.417092000	-4.419739000	1.681496000
6	5.286420000	-5.510556000	1.751293000
6	6.485438000	-5.411058000	2.466525000
6	6.807349000	-4.213038000	3.111115000
6	5.935649000	-3.121784000	3.040995000
7	3.497707000	0.201436000	1.207257000
6	4.212243000	-0.955980000	1.448007000
6	5.419222000	-0.964286000	0.634320000
6	5.384754000	0.146068000	-0.145540000
6	4.187970000	0.883230000	0.214138000
8	-5.236627000	0.554214000	-0.059603000
1	3.482426000	-4.496063000	1.121483000
1	0.010393000	-3.811983000	4.202760000
1	2.648149000	-4.196421000	3.708245000
1	-2.914792000	0.513103000	4.422747000
1	-2.426887000	3.100831000	3.936153000
1	0.894997000	5.582728000	0.535177000
1	3.053870000	4.782089000	-0.914039000
1	6.097523000	0.451962000	-0.905740000
1	6.167823000	-1.751697000	0.642949000
1	-1.060001000	-2.361030000	5.911328000
1	-3.019772000	-3.531408000	6.890070000
1	-5.126218000	-3.757663000	5.559948000
1	-5.235150000	-2.821224000	3.251240000
1	1.115626000	5.643397000	3.176239000
1	0.179535000	7.840184000	3.829888000
1	-2.271395000	8.279276000	3.603319000
1	-3.762546000	6.476413000	2.729357000
1	-2.829724000	4.295884000	2.066246000
1	6.344158000	3.056298000	-0.297895000
1	7.700429000	3.921448000	-2.199418000
1	6.696219000	4.014206000	-4.490159000
1	4.356626000	3.267125000	-4.846683000
1	5.029602000	-6.441643000	1.239825000
1	7.165864000	-6.264337000	2.520259000
1	7.740469000	-4.126846000	3.673438000
1	6.188359000	-2.186005000	3.545020000
1	-5.067532000	-0.632020000	2.314148000
1	-3.714343000	0.405739000	1.815700000
1	1.337794000	1.857010000	-4.302729000
1	2.990727000	1.621370000	-4.887546000
7	1.449288000	-0.824723000	-4.122986000
19	-1.654133000	-1.013113000	-2.017780000
8	-1.676019000	-0.330247000	-4.841913000
8	-0.281162000	-3.242685000	-2.960659000
8	-4.212470000	-0.592505000	-3.350991000
8	-3.040839000	-3.710067000	-2.083936000
6	0.632260000	-3.252850000	-4.047770000
1	0.076557000	-3.140413000	-4.995562000
6	1.751333000	-2.245310000	-3.883203000
1	1.129315000	-4.237923000	-4.093197000
1	2.566835000	-2.555867000	-4.563301000
1	2.139030000	-2.315295000	-2.860624000
6	-0.866543000	-4.522529000	-2.768202000
1	-1.307979000	-4.878496000	-3.715022000
1	-0.086226000	-5.244018000	-2.460252000
6	-1.917812000	-4.504174000	-1.688685000
1	-2.243640000	-5.547984000	-1.535107000
1	-1.499774000	-4.120950000	-0.748543000
6	-4.225262000	-3.964702000	-1.333813000
1	-5.064538000	-4.002142000	-2.041048000
1	-4.162446000	-4.965688000	-0.877012000
6	-4.491490000	-2.987285000	-0.194376000

1	-5.330042000	-3.409069000	0.390390000
1	-3.612957000	-2.954898000	0.450059000
6	0.733856000	-0.451981000	-5.349581000
6	-0.515078000	0.407705000	-5.206007000
1	1.413614000	0.076937000	-6.036916000
1	0.450774000	-1.371233000	-5.877220000
1	-0.692937000	0.884067000	-6.187086000
1	-0.376919000	1.217348000	-4.470265000
6	-2.833920000	0.489192000	-5.007190000
1	-2.875943000	0.854245000	-6.049375000
6	-4.100566000	-0.271922000	-4.729900000
1	-2.758026000	1.371418000	-4.350023000
1	-4.954332000	0.359752000	-5.039554000
1	-4.116968000	-1.193666000	-5.333932000
6	-5.789835000	-1.464046000	-1.708443000
1	-6.559541000	-2.247969000	-1.607410000
1	-6.280688000	-0.496519000	-1.567247000
6	-5.254487000	-1.528380000	-3.129105000
1	-6.113798000	-1.298926000	-3.785608000
1	-4.899200000	-2.530526000	-3.411175000
8	-2.681478000	-3.096135000	-4.930028000
1	-2.201579000	-2.250087000	-4.928481000
1	-2.867805000	-3.273682000	-3.990277000
6	1.274565000	-0.369881000	0.006568000
8	1.627153000	-1.511593000	-0.215839000
8	0.651340000	0.282574000	-0.978543000
6	-3.460509000	2.527725000	-1.905926000
1	-4.190622000	3.299347000	-1.629573000
1	-3.963642000	1.608889000	-2.222346000
1	-2.849685000	2.933372000	-2.727954000
6	-2.546276000	2.240803000	-0.754465000
8	-2.232301000	1.138759000	-0.357900000
8	-2.045549000	3.359282000	-0.203620000
1	0.487947000	1.251197000	-0.866863000
8	-4.223534000	5.171045000	-0.150694000
1	-4.209045000	6.004390000	0.340540000
1	-3.294368000	4.879363000	-0.164871000
8	-1.243144000	-2.136346000	0.292723000
1	-1.765278000	-1.901197000	1.077211000
1	-0.554700000	-2.770963000	0.574971000
8	-4.761890000	2.884620000	1.445643000
1	-4.955003000	2.119617000	0.868516000
1	-4.721843000	3.662332000	0.857228000
8	0.881840000	-3.931488000	0.608729000
1	1.060904000	-3.974880000	1.563628000
1	1.321698000	-3.094218000	0.337112000
8	0.232368000	2.718407000	-1.834978000
1	1.172118000	2.778952000	-2.103021000
1	0.093836000	3.516860000	-1.302196000
1	-1.465197000	3.125998000	0.558619000

^{3}IM2$_{\text{acetate, 6W, K+}}$:

6	-6.338420000	-0.079080000	-2.295077000
6	-6.296008000	-0.121881000	-0.891806000
6	-7.474201000	-0.404361000	-0.182488000
6	-8.675936000	-0.629572000	-0.859775000
6	-8.712739000	-0.579873000	-2.256878000
6	-7.540799000	-0.307630000	-2.970067000
6	-5.016025000	0.105991000	-0.155574000
6	-4.372473000	-1.005806000	0.406833000
7	-3.216075000	-0.985517000	1.156523000
6	-2.870159000	-2.301127000	1.375960000
6	-3.819675000	-3.172898000	0.717186000
6	-4.769027000	-2.373310000	0.153607000
26	-2.099073000	0.598062000	1.612159000
7	-3.281271000	1.735948000	0.475902000
6	-2.965500000	2.998069000	0.038718000
6	-3.988661000	3.480792000	-0.861871000
6	-4.938557000	2.503365000	-0.939670000
6	-4.454262000	1.389311000	-0.151734000
6	-1.856832000	3.749486000	0.443201000
6	-1.444846000	4.924260000	-0.378034000
6	-0.651092000	4.677497000	-1.518793000
6	-0.146313000	5.737621000	-2.276638000
6	-0.448495000	7.053749000	-1.911730000
6	-1.251817000	7.314502000	-0.799945000
6	-1.741775000	6.248062000	-0.038783000
6	-1.799358000	-2.739926000	2.168482000
6	-1.504398000	-4.199333000	2.248797000
6	-2.443032000	-5.104155000	2.771111000
6	-2.170578000	-6.470296000	2.863041000
6	-0.934710000	-6.960247000	2.430966000
6	0.016554000	-6.080711000	1.911856000
6	-0.259061000	-4.712646000	1.823653000
8	0.753981000	-3.897432000	1.390150000
6	0.553512000	-3.078724000	0.237586000
6	1.345324000	-3.623330000	-0.952743000
7	2.708860000	-3.721937000	-0.858005000
8	-0.445332000	3.358987000	-1.846830000
6	0.747158000	2.955200000	-2.484362000
6	1.937132000	3.078969000	-1.529103000
8	1.718381000	3.330999000	-0.345287000
7	-1.037107000	-0.495903000	2.896428000
6	-1.008629000	-1.870269000	2.938199000
6	-0.097079000	-2.309374000	3.967613000
6	0.403029000	-1.185838000	4.565603000
6	-0.165323000	-0.051209000	3.871190000
6	0.192935000	1.283629000	4.107136000
6	1.190784000	1.587740000	5.175230000
6	2.512242000	1.116958000	5.080972000
6	3.450836000	1.429376000	6.067238000
6	3.083602000	2.217448000	7.162397000
6	1.772386000	2.693294000	7.264234000
6	0.833972000	2.382431000	6.276987000
7	-1.124744000	2.231955000	2.244241000
6	-0.242359000	2.342127000	3.295296000
6	0.339094000	3.667934000	3.303654000
6	-0.140860000	4.317321000	2.198813000
6	-1.072740000	3.422592000	1.558614000
8	0.736187000	-3.952976000	-1.974186000
1	2.806047000	0.512827000	4.221360000
1	0.105737000	-3.345983000	4.222205000
1	1.093581000	-1.130068000	5.401935000
1	-3.741758000	-4.253261000	0.668169000
1	-5.631637000	-2.671848000	-0.434919000
1	-5.864907000	2.512962000	-1.507226000
1	-3.985478000	4.457892000	-1.339569000
1	0.100625000	5.318863000	1.854135000
1	1.045564000	4.041624000	4.040633000
1	-3.398683000	-4.715287000	3.127678000
1	-2.918790000	-7.147875000	3.279673000
1	-0.709145000	-8.027040000	2.497076000
1	0.989829000	-6.434610000	1.566672000
1	-7.444522000	-0.444593000	0.908880000
1	-9.585831000	-0.844030000	-0.293973000
1	-9.651083000	-0.757384000	-2.787882000
1	-7.557367000	-0.279071000	-4.062547000
1	-5.425168000	0.103193000	-2.865287000
1	-2.355238000	6.437741000	0.844692000
1	-1.490448000	8.342420000	-0.519726000
1	-0.052759000	7.877707000	-2.509847000
1	0.476819000	5.548935000	-3.151262000
1	4.474569000	1.059109000	5.975366000
1	3.817726000	2.462028000	7.933563000

1	1.477459000	3.308694000	8.117460000
1	-0.189712000	2.754578000	6.357476000
1	-0.496165000	-3.070880000	-0.069806000
1	0.867917000	-2.057338000	0.491540000
1	0.603645000	1.890657000	-2.729603000
1	0.916584000	3.499036000	-3.426070000
7	3.196980000	2.868692000	-2.018221000
19	2.893129000	-0.474538000	-1.301906000
8	3.761418000	0.429253000	-3.898949000
8	4.946323000	1.050887000	-0.355377000
8	3.601289000	-2.430864000	-3.253960000
8	5.098051000	-1.825032000	-0.121953000
6	5.422584000	2.214406000	-1.008775000
1	5.868723000	1.939350000	-1.980825000
6	4.319730000	3.242843000	-1.141546000
1	6.221837000	2.685305000	-0.409506000
1	4.765023000	4.177040000	-1.525179000
1	3.910095000	3.447088000	-0.148342000
6	5.959235000	0.376625000	0.374071000
1	6.827812000	0.182360000	-0.277748000
1	6.295855000	1.005040000	1.220016000
6	5.434209000	-0.918550000	0.937241000
1	6.230357000	-1.360709000	1.560965000
1	4.546253000	-0.741300000	1.563643000
6	4.884428000	-3.166507000	0.316911000
1	5.442847000	-3.832188000	-0.355055000
1	5.321578000	-3.298708000	1.319019000
6	3.419714000	-3.574843000	0.423441000
1	3.392342000	-4.537571000	0.962675000
1	2.900808000	-2.838704000	1.045017000
6	3.507963000	2.833428000	-3.456985000
6	3.073941000	1.621865000	-4.261964000
1	3.083956000	3.722520000	-3.953338000
1	4.597710000	2.925895000	-3.546331000
1	3.295482000	1.860404000	-5.318733000
1	1.992757000	1.433294000	-4.190398000
6	3.400200000	-0.617114000	-4.807523000
1	3.683787000	-0.320951000	-5.834033000
6	4.091099000	-1.913687000	-4.480024000
1	2.309004000	-0.754448000	-4.762239000
1	3.877811000	-2.624339000	-5.301103000
1	5.183018000	-1.771233000	-4.425508000
6	3.389435000	-4.442369000	-1.938388000
1	3.989452000	-5.260526000	-1.507959000
1	2.603092000	-4.887709000	-2.556895000
6	4.287225000	-3.593790000	-2.832595000
1	4.559406000	-4.218708000	-3.702557000
1	5.229900000	-3.306323000	-2.338398000
8	6.238680000	-0.607767000	-2.614850000
1	5.486413000	-0.125789000	-2.999481000
1	5.862053000	-1.102587000	-1.866448000
6	-0.510293000	0.511978000	0.129658000
8	0.583660000	0.680729000	-0.119804000
8	-3.711066000	-0.176259000	-4.436909000
6	-0.452347000	-1.800394000	-4.318160000
1	-0.553536000	-2.870547000	-4.084123000
1	0.362336000	-1.665665000	-5.040537000
1	-1.396139000	-1.442885000	-4.759290000
6	-0.201144000	-1.029283000	-3.028509000
8	0.819046000	-0.313782000	-2.929285000
8	-1.086772000	-1.139388000	-2.114385000
1	-3.213902000	0.469343000	-3.891792000
8	-3.222983000	-2.480844000	-2.801203000
1	-2.930634000	-3.011706000	-3.555933000
1	-2.390382000	-1.987891000	-2.512603000
8	2.330686000	-0.701264000	1.738274000
1	1.767689000	-1.006115000	2.464653000

1	2.360603000	0.286478000	1.818200000
8	-1.930604000	-4.640775000	-1.368783000
1	-1.039771000	-4.377994000	-1.673619000
1	-2.501104000	-3.905555000	-1.656682000
8	2.650898000	1.978784000	1.835919000
1	2.220885000	2.452135000	2.563024000
1	2.301982000	2.435051000	1.036640000
8	-2.254101000	1.262216000	-2.605861000
1	-1.803716000	2.043906000	-2.246375000
1	-1.829527000	0.479060000	-2.194152000
1	-3.688348000	-0.977384000	-3.876980000

^{5}RC1 $_{\text{acetate, 6W, K+}}$:

6	-2.991236000	5.501196000	1.208562000
6	-1.581869000	5.463747000	1.270503000
6	-0.894328000	6.693249000	1.332657000
6	-1.580386000	7.909353000	1.325094000
6	-2.976826000	7.929994000	1.258771000
6	-3.675868000	6.719887000	1.200432000
6	-0.805856000	4.195855000	1.255074000
6	-1.179597000	3.118645000	2.107046000
7	-0.538659000	1.880290000	2.148620000
6	-1.304682000	1.062428000	2.945208000
6	-2.437708000	1.778045000	3.426290000
6	-2.331089000	3.073292000	2.943742000
26	1.254201000	1.416680000	1.438762000
7	1.233673000	3.115981000	0.359332000
6	1.951593000	3.244119000	-0.815242000
6	1.364376000	4.297334000	-1.618478000
6	0.360946000	4.854706000	-0.878952000
6	0.248625000	4.083070000	0.343780000
6	3.103553000	2.525174000	-1.127578000
6	3.736852000	2.648508000	-2.464964000
6	3.851156000	1.506623000	-3.303327000
6	4.425446000	1.616558000	-4.575842000
6	4.903516000	2.852335000	-5.028456000
6	4.810655000	3.982098000	-4.216769000
6	4.232634000	3.867186000	-2.947453000
6	-0.945390000	-0.242182000	3.404856000
6	-2.020147000	-1.061010000	4.024003000
6	-1.993744000	-1.417969000	5.386011000
6	-3.014865000	-2.165421000	5.974436000
6	-4.107375000	-2.573985000	5.204540000
6	-4.166885000	-2.229991000	3.852539000
6	-3.141159000	-1.478335000	3.269326000
8	-3.180165000	-1.193701000	1.918847000
6	-4.274179000	-0.418612000	1.456413000
6	-4.846569000	-0.713504000	0.071963000
7	-4.774614000	-1.936962000	-0.543332000
8	3.351895000	0.339084000	-2.814565000
6	3.627459000	-0.875553000	-3.488365000
6	3.428945000	-2.016849000	-2.484119000
8	4.230508000	-2.119556000	-1.556617000
7	1.451129000	0.049966000	2.906848000
6	0.396392000	-0.631445000	3.479756000
6	0.908357000	-1.762047000	4.229609000
6	2.272918000	-1.717413000	4.147351000
6	2.613542000	-0.614308000	3.274272000
6	3.877602000	-0.418419000	2.724144000
6	4.992803000	-1.286630000	3.200678000
6	5.511316000	-2.286415000	2.357791000
6	6.531020000	-3.131349000	2.800867000
6	7.049687000	-2.990557000	4.093444000
6	6.539180000	-2.001926000	4.939896000
6	5.516903000	-1.156870000	4.496201000
7	3.151559000	1.172291000	0.958097000
6	4.120535000	0.429088000	1.618032000

6	5.361878000	0.553409000	0.924777000
6	5.119759000	1.318818000	-0.203378000
6	3.743485000	1.691126000	-0.166511000
8	-5.462914000	0.234390000	-0.424788000
1	5.094512000	-2.401260000	1.354667000
1	0.303151000	-2.496840000	4.752224000
1	2.986617000	-2.404061000	4.595948000
1	-3.193999000	1.378579000	4.097696000
1	-3.003732000	3.900023000	3.146371000
1	-0.297405000	5.669912000	-1.167944000
1	1.682311000	4.567841000	-2.620871000
1	5.823780000	1.611089000	-0.979279000
1	6.300739000	0.099654000	1.231354000
1	-1.158434000	-1.071170000	5.996604000
1	-2.963033000	-2.416930000	7.036089000
1	-4.912836000	-3.161738000	5.650422000
1	-5.010079000	-2.555295000	3.240951000
1	0.195258000	6.686332000	1.397610000
1	-1.019968000	8.846126000	1.376712000
1	-3.515764000	8.880262000	1.252235000
1	-4.766914000	6.719892000	1.136413000
1	-3.553295000	4.570669000	1.134164000
1	4.170872000	4.742778000	-2.297970000
1	5.190455000	4.946438000	-4.561223000
1	5.348335000	2.920990000	-6.024161000
1	4.490027000	0.747544000	-5.230159000
1	6.918780000	-3.908009000	2.136922000
1	7.847734000	-3.651663000	4.439876000
1	6.938839000	-1.885940000	5.950382000
1	5.117086000	-0.384911000	5.157730000
1	-5.123309000	-0.493012000	2.156917000
1	-3.972268000	0.637138000	1.414768000
1	3.023061000	-0.961584000	-4.402879000
1	4.689841000	-0.921618000	-3.779574000
7	2.419575000	-2.906286000	-2.664062000
19	-1.488332000	-2.085550000	-1.090089000
8	-0.768443000	-1.902933000	-3.868125000
8	0.059459000	-4.633409000	-1.391425000
8	-3.504699000	-1.567865000	-3.118347000
8	-2.927621000	-4.478952000	-1.119421000
6	1.340634000	-5.064429000	-1.827341000
1	1.286009000	-5.459790000	-2.859892000
6	2.396610000	-3.994629000	-1.672479000
1	1.665637000	-5.907798000	-1.189897000
1	3.376497000	-4.498698000	-1.706033000
1	2.306179000	-3.564112000	-0.667384000
6	-0.833455000	-5.727281000	-1.261970000
1	-1.131255000	-6.095266000	-2.259723000
1	-0.324128000	-6.556816000	-0.735320000
6	-2.049351000	-5.384563000	-0.434793000
1	-2.587408000	-6.329819000	-0.247525000
1	-1.746344000	-4.942351000	0.526783000
6	-4.244710000	-4.429465000	-0.569397000
1	-4.958329000	-4.576248000	-1.391676000
1	-4.382832000	-5.278358000	0.117797000
6	-4.569136000	-3.174695000	0.234323000
1	-5.499478000	-3.406022000	0.786144000
1	-3.778355000	-3.006866000	0.971854000
6	1.443213000	-2.828655000	-3.747964000
6	0.611767000	-1.541122000	-3.769613000
1	1.930568000	-2.999043000	-4.721389000
1	0.737217000	-3.652850000	-3.612844000
1	0.870833000	-0.898550000	-4.625970000
1	0.774080000	-0.956286000	-2.851146000
6	-1.598222000	-0.885549000	-4.409825000
1	-1.284777000	-0.655868000	-5.445360000
6	-3.025184000	-1.380701000	-4.437085000
1	-1.482240000	0.042068000	-3.826911000
1	-3.654018000	-0.646335000	-4.975740000
1	-3.060796000	-2.331207000	-4.996311000
6	-5.498134000	-2.043043000	-1.816813000
1	-6.297646000	-2.797513000	-1.725491000
1	-5.984657000	-1.076241000	-1.973228000
6	-4.661771000	-2.379639000	-3.047804000
1	-5.312962000	-2.203948000	-3.923605000
1	-4.362138000	-3.439178000	-3.083525000
8	-2.052410000	-4.445265000	-3.912205000
1	-1.535834000	-3.618475000	-3.929241000
1	-2.433918000	-4.464133000	-3.016713000
6	0.748718000	0.136038000	-0.287550000
8	0.969232000	-1.101254000	-0.266382000
8	0.332904000	0.716205000	-1.343041000
6	-3.709247000	1.627401000	-2.676498000
1	-3.900875000	2.704366000	-2.750880000
1	-4.605738000	1.074742000	-2.384310000
1	-3.384362000	1.289721000	-3.671401000
6	-2.586889000	1.348772000	-1.711522000
8	-2.565305000	0.413495000	-0.930610000
8	-1.562798000	2.191734000	-1.819602000
1	-0.712841000	1.707256000	-1.422068000
8	-3.052857000	4.559143000	-1.693150000
1	-2.900186000	5.202498000	-0.985011000
1	-2.288178000	3.952976000	-1.648448000
8	-0.997045000	-3.090643000	1.493565000
1	-1.141367000	-2.217193000	1.884715000
1	-0.011246000	-3.171104000	1.469748000
8	-4.722513000	2.928297000	0.123025000
1	-4.968174000	2.025972000	-0.158926000
1	-4.253195000	3.348203000	-0.620124000
8	1.712068000	-2.991979000	1.340273000
1	2.021957000	-2.688465000	2.206685000
1	1.536433000	-2.156591000	0.815839000
8	0.599342000	1.833815000	-3.863819000
1	0.860486000	1.354065000	-3.053421000
1	-0.188928000	2.301005000	-3.549393000

[5]TS1 $_{\text{acetate, 6W, K+}}$:

6	-2.305991000	5.948054000	1.107442000
6	-0.916874000	5.681463000	1.111817000
6	-0.040351000	6.789945000	1.131377000
6	-0.522949000	8.097544000	1.159793000
6	-1.901482000	8.342523000	1.158947000
6	-2.785943000	7.260364000	1.128154000
6	-0.358425000	4.312777000	1.071839000
6	-0.879202000	3.293716000	1.919189000
7	-0.378281000	1.996715000	1.977477000
6	-1.225125000	1.279276000	2.789200000
6	-2.272073000	2.112121000	3.259364000
6	-2.027745000	3.386101000	2.755805000
26	1.375598000	1.377614000	1.305111000
7	1.460579000	2.923838000	0.079010000
6	2.159120000	2.947836000	-1.124191000
6	1.741783000	4.068945000	-1.891623000
6	0.836730000	4.775951000	-1.112783000
6	0.646815000	4.036090000	0.093317000
6	3.222383000	2.063551000	-1.422243000
6	3.831941000	2.056708000	-2.775414000
6	3.817252000	0.871680000	-3.554675000
6	4.398989000	0.851461000	-4.827726000
6	5.012843000	2.001150000	-5.336579000
6	5.046733000	3.173566000	-4.581351000
6	4.457251000	3.190323000	-3.313682000
6	-0.964993000	-0.030119000	3.313455000
6	-2.099055000	-0.731639000	3.958725000

6	-2.086891000	-1.049902000	5.332646000
6	-3.164757000	-1.677317000	5.955837000
6	-4.299776000	-2.008325000	5.210269000
6	-4.346589000	-1.698579000	3.849931000
6	-3.266652000	-1.060736000	3.228486000
8	-3.309223000	-0.803690000	1.873715000
6	-4.350523000	0.053204000	1.424051000
6	-4.955117000	-0.213333000	0.048321000
7	-5.037835000	-1.467407000	-0.504743000
8	3.180205000	-0.209171000	-3.016786000
6	3.443202000	-1.501020000	-3.548229000
6	3.187723000	-2.527681000	-2.438263000
8	4.033365000	-2.655329000	-1.555115000
7	1.459573000	0.066919000	2.819876000
6	0.366365000	-0.505036000	3.459854000
6	0.812710000	-1.555573000	4.313063000
6	2.201258000	-1.588907000	4.224355000
6	2.578993000	-0.607824000	3.260264000
6	3.870940000	-0.532781000	2.663161000
6	4.917852000	-1.434144000	3.218693000
6	5.293337000	-2.591725000	2.513709000
6	6.247035000	-3.466427000	3.039353000
6	6.839255000	-3.198750000	4.278735000
6	6.467316000	-2.053993000	4.990932000
6	5.510175000	-1.180445000	4.465903000
7	3.231634000	0.940181000	0.781557000
6	4.141104000	0.148707000	1.467391000
6	5.343939000	0.026290000	0.689420000
6	5.116283000	0.665907000	-0.508288000
6	3.804835000	1.235363000	-0.436702000
8	-5.451395000	0.777312000	-0.493501000
1	4.815897000	-2.803938000	1.554834000
1	0.181406000	-2.199987000	4.916443000
1	2.882697000	-2.254311000	4.748303000
1	-3.061074000	1.808989000	3.943450000
1	-2.609620000	4.280523000	2.948654000
1	0.316203000	5.690774000	-1.382477000
1	2.089861000	4.317506000	-2.889657000
1	5.793877000	0.767113000	-1.352900000
1	6.239590000	-0.504699000	1.000495000
1	-1.218342000	-0.759782000	5.924382000
1	-3.123000000	-1.895773000	7.025155000
1	-5.149519000	-2.506595000	5.682050000
1	-5.227899000	-1.960061000	3.262558000
1	1.036061000	6.609764000	1.136643000
1	0.181652000	8.932600000	1.186026000
1	-2.281000000	9.366768000	1.177118000
1	-3.864608000	7.435522000	1.109346000
1	-3.013902000	5.121593000	1.049805000
1	4.497824000	4.096976000	-2.707634000
1	5.534893000	4.069828000	-4.969841000
1	5.463319000	1.970811000	-6.331441000
1	4.364490000	-0.051839000	-5.436384000
1	6.525464000	-4.364272000	2.481846000
1	7.585953000	-3.882516000	4.689676000
1	6.924795000	-1.839217000	5.959907000
1	5.216281000	-0.288706000	5.024511000
1	-5.192312000	0.042754000	2.137298000
1	-3.969146000	1.081839000	1.373067000
1	2.859594000	-1.672535000	-4.464644000
1	4.510905000	-1.597213000	-3.797013000
7	2.061842000	-3.287019000	-2.474267000
19	-1.729084000	-1.923582000	-0.895911000
8	-1.043199000	-2.015911000	-3.724934000
8	-0.426934000	-4.637441000	-1.029763000
8	-3.724531000	-1.343641000	-3.070675000
8	-3.396678000	-4.166570000	-0.846759000

6	0.797127000	-5.238128000	-1.427821000
1	0.688266000	-5.718853000	-2.419086000
6	1.959317000	-4.272919000	-1.385832000
1	1.043735000	-6.046818000	-0.714910000
1	2.882395000	-4.874500000	-1.392993000
1	1.944096000	-3.753131000	-0.420708000
6	-1.433011000	-5.616844000	-0.825630000
1	-1.755817000	-6.036282000	-1.795151000
1	-1.019229000	-6.445925000	-0.220380000
6	-2.619477000	-5.081606000	-0.058877000
1	-3.249075000	-5.948691000	0.203965000
1	-2.282128000	-4.583024000	0.863099000
6	-4.737663000	-3.991534000	-0.388895000
1	-5.405148000	-4.118293000	-1.252114000
1	-4.987543000	-4.795527000	0.320628000
6	-5.006975000	-2.677152000	0.334645000
1	-5.991519000	-2.797620000	0.825326000
1	-4.257305000	-2.545330000	1.119433000
6	1.034404000	-3.174921000	-3.505134000
6	0.358675000	-1.802663000	-3.576613000
1	1.431472000	-3.471301000	-4.489175000
1	0.246099000	-3.892780000	-3.262830000
1	0.723473000	-1.202012000	-4.424066000
1	0.557819000	-1.228189000	-2.658135000
6	-1.739959000	-0.950147000	-4.354836000
1	-1.379278000	-0.825686000	-5.392745000
6	-3.210863000	-1.285440000	-4.386670000
1	-1.534542000	-0.007328000	-3.826531000
1	-3.745238000	-0.517240000	-4.978021000
1	-3.343893000	-2.256759000	-4.894762000
6	-5.762279000	-1.550652000	-1.777564000
1	-6.638469000	-2.210964000	-1.660323000
1	-6.138901000	-0.544201000	-1.982769000
6	-4.957062000	-2.029996000	-2.980780000
1	-5.579748000	-1.831073000	-3.872728000
1	-4.765246000	-3.115380000	-2.964250000
8	-2.541515000	-4.424635000	-3.601422000
1	-1.972142000	-3.637748000	-3.685420000
1	-2.914252000	-4.344326000	-2.704952000
6	0.912252000	0.028565000	-0.016909000
8	1.209618000	-1.157964000	0.121479000
8	0.292475000	0.438311000	-1.070321000
6	-3.390501000	1.896155000	-2.737150000
1	-3.494653000	2.986900000	-2.777686000
1	-4.348431000	1.410110000	-2.531712000
1	-3.022947000	1.574347000	-3.722959000
6	-2.369864000	1.492300000	-1.695820000
8	-2.572880000	0.582172000	-0.897779000
8	-1.230238000	2.141502000	-1.731795000
1	-0.388704000	1.401100000	-1.214079000
8	-2.412571000	4.647596000	-1.684936000
1	-2.113239000	5.315376000	-1.051677000
1	-1.734096000	3.940515000	-1.645658000
8	-1.088178000	-2.890859000	1.624439000
1	-1.084411000	-2.053537000	2.110501000
1	-0.136700000	-3.151469000	1.632061000
8	-4.432657000	3.420804000	0.017442000
1	-4.720661000	2.527360000	-0.250003000
1	-3.780534000	3.722579000	-0.642919000
8	1.648979000	-3.231543000	1.690953000
1	1.865077000	-2.942902000	2.594203000
1	1.627298000	-2.382826000	1.184262000
8	0.598503000	1.499585000	-3.834483000
1	1.093419000	0.985794000	-3.177225000
1	-0.056972000	1.948733000	-3.271090000

^{5}IM1$_{\text{acetate, 6W, K+}}$:

6	-2.236893000	5.927762000	1.198351000
6	-0.850072000	5.654621000	1.172671000
6	0.033842000	6.757107000	1.180172000
6	-0.440502000	8.067279000	1.225702000
6	-1.817354000	8.319379000	1.253969000
6	-2.708596000	7.242519000	1.235660000
6	-0.300947000	4.282800000	1.116088000
6	-0.800639000	3.265880000	1.973785000
7	-0.297207000	1.967650000	2.021332000
6	-1.128798000	1.249539000	2.847004000
6	-2.164502000	2.081638000	3.340799000
6	-1.931411000	3.356323000	2.834110000
26	1.441850000	1.353908000	1.317084000
7	1.487730000	2.886972000	0.079938000
6	2.167995000	2.918919000	-1.135808000
6	1.739601000	4.046346000	-1.887907000
6	0.850947000	4.749564000	-1.088636000
6	0.681177000	4.002719000	0.114484000
6	3.228675000	2.045550000	-1.456538000
6	3.806312000	2.057006000	-2.824453000
6	3.731567000	0.898283000	-3.636061000
6	4.278967000	0.897121000	-4.924577000
6	4.916657000	2.041506000	-5.415156000
6	5.007907000	3.189462000	-4.627507000
6	4.451971000	3.187250000	-3.344753000
6	-0.867316000	-0.068764000	3.350562000
6	-1.997584000	-0.771950000	3.998842000
6	-1.965254000	-1.122956000	5.364348000
6	-3.043307000	-1.744185000	5.993389000
6	-4.198593000	-2.034434000	5.262394000
6	-4.264919000	-1.692816000	3.910380000
6	-3.184189000	-1.063414000	3.281845000
8	-3.243227000	-0.778388000	1.933977000
6	-4.279294000	0.098835000	1.509895000
6	-4.929975000	-0.151564000	0.151002000
7	-5.055756000	-1.404741000	-0.401481000
8	3.072197000	-0.176104000	-3.111400000
6	3.301412000	-1.463829000	-3.669594000
6	3.070579000	-2.502829000	-2.566446000
8	3.941605000	-2.649596000	-1.711754000
7	1.547567000	0.022354000	2.809559000
6	0.464767000	-0.551372000	3.467391000
6	0.923222000	-1.617657000	4.292571000
6	2.309397000	-1.660724000	4.168871000
6	2.671551000	-0.668739000	3.211085000
6	3.952663000	-0.592931000	2.588798000
6	5.003511000	-1.509587000	3.111401000
6	5.334378000	-2.675398000	2.398198000
6	6.292379000	-3.563348000	2.892977000
6	6.932871000	-3.300262000	4.109039000
6	6.604976000	-2.147215000	4.829419000
6	5.643520000	-1.260334000	4.335465000
7	3.293974000	0.917828000	0.744796000
6	4.204643000	0.101433000	1.394966000
6	5.381884000	-0.036267000	0.582591000
6	5.132144000	0.620387000	-0.603195000
6	3.834920000	1.210601000	-0.487960000
8	-5.424950000	0.848623000	-0.370829000
1	4.818195000	-2.883986000	1.458886000
1	0.301893000	-2.265797000	4.902148000
1	2.998467000	-2.337867000	4.667134000
1	-2.939749000	1.776751000	4.039508000
1	-2.509595000	4.250356000	3.039473000
1	0.323515000	5.664464000	-1.343789000
1	2.070663000	4.298800000	-2.890684000
1	5.785501000	0.715368000	-1.467355000
1	6.274987000	-0.588489000	0.862402000

1	-1.081467000	-0.861501000	5.946676000
1	-2.985848000	-1.988399000	7.056353000
1	-5.049519000	-2.526093000	5.738947000
1	-5.162764000	-1.923442000	3.335615000
1	1.109113000	6.570866000	1.162601000
1	0.269120000	8.898269000	1.242206000
1	-2.190632000	9.345622000	1.285223000
1	-3.786373000	7.423900000	1.239826000
1	-2.950904000	5.105954000	1.152142000
1	4.533850000	4.074275000	-2.714080000
1	5.513525000	4.081334000	-5.003424000
1	5.339920000	2.027129000	-6.422220000
1	4.198755000	0.014015000	-5.558130000
1	6.536447000	-4.467512000	2.329677000
1	7.682997000	-3.994268000	4.495827000
1	7.100237000	-1.936384000	5.780489000
1	5.383932000	-0.361952000	4.900375000
1	-5.100388000	0.106766000	2.246841000
1	-3.876350000	1.117819000	1.447043000
1	2.686505000	-1.613525000	-4.568856000
1	4.359016000	-1.568781000	-3.954433000
7	1.936569000	-3.251441000	-2.577681000
19	-1.803014000	-1.868932000	-0.908895000
8	-1.189246000	-1.974536000	-3.751523000
8	-0.508093000	-4.598421000	-1.063597000
8	-3.844880000	-1.343212000	-3.014684000
8	-3.472995000	-4.120695000	-0.793421000
6	0.692233000	-5.208129000	-1.514288000
1	0.540462000	-5.676960000	-2.505608000
6	1.867182000	-4.257948000	-1.505658000
1	0.957171000	-6.027545000	-0.820371000
1	2.780997000	-4.871339000	-1.562878000
1	1.901709000	-3.755584000	-0.532127000
6	-1.510997000	-5.571309000	-0.817604000
1	-1.866924000	-5.998586000	-1.772037000
1	-1.080653000	-6.396458000	-0.218422000
6	-2.670269000	-5.024059000	-0.018772000
1	-3.291036000	-5.886586000	0.278181000
1	-2.300597000	-4.513226000	0.883934000
6	-4.795131000	-3.932526000	-0.289581000
1	-5.493432000	-4.054704000	-1.128553000
1	-5.027959000	-4.732439000	0.430526000
6	-5.024719000	-2.613351000	0.438423000
1	-5.997551000	-2.717275000	0.955871000
1	-4.251424000	-2.493882000	1.201582000
6	0.890942000	-3.135737000	-3.590200000
6	0.215829000	-1.761474000	-3.655376000
1	1.268376000	-3.436184000	-4.580762000
1	0.104284000	-3.849787000	-3.331764000
1	0.552248000	-1.172894000	-4.523267000
1	0.449692000	-1.176984000	-2.752314000
6	-1.915883000	-0.912977000	-4.356766000
1	-1.583509000	-0.777936000	-5.402665000
6	-3.381457000	-1.271902000	-4.349095000
1	-1.716590000	0.029706000	-3.825379000
1	-3.944565000	-0.505348000	-4.914794000
1	-3.520259000	-2.242832000	-4.856167000
6	-5.831606000	-1.472993000	-1.644893000
1	-6.727742000	-2.097660000	-1.486860000
1	-6.174655000	-0.453593000	-1.846015000
6	-5.092155000	-1.991817000	-2.874493000
1	-5.741618000	-1.781013000	-3.744148000
1	-4.932310000	-3.082139000	-2.854902000
8	-2.686421000	-4.386974000	-3.568767000
1	-2.119489000	-3.600922000	-3.672450000
1	-3.038255000	-4.299282000	-2.664535000
6	0.969355000	-0.001685000	0.035592000

8	1.308502000	-1.173297000	0.105813000
8	0.260986000	0.367694000	-1.017272000
6	-3.416224000	1.886451000	-2.701561000
1	-4.036137000	2.719493000	-2.343443000
1	-4.058257000	1.015314000	-2.861758000
1	-2.942504000	2.209204000	-3.639687000
6	-2.363692000	1.556210000	-1.653187000
8	-2.560671000	0.643275000	-0.840216000
8	-1.280259000	2.259387000	-1.667289000
1	-0.216281000	1.270037000	-1.018433000
8	-2.475127000	4.675625000	-1.645385000
1	-2.084972000	5.292334000	-1.010627000
1	-1.852701000	3.907804000	-1.666004000
8	-1.032658000	-2.835984000	1.565562000
1	-0.998884000	-2.012104000	2.073120000
1	-0.094427000	-3.136996000	1.568619000
8	-4.446380000	3.519297000	0.110373000
1	-4.687959000	2.602352000	-0.122100000
1	-3.781901000	3.816735000	-0.542965000
8	1.701028000	-3.300891000	1.636071000
1	1.921670000	-3.028974000	2.543431000
1	1.713952000	-2.448164000	1.141083000
8	0.430418000	1.574971000	-3.776947000
1	1.008736000	1.035096000	-3.217742000
1	-0.175552000	1.971150000	-3.117171000

^{5}RC2$_{\text{acetate, 6W, K+}}$:

6	-1.111141000	6.151469000	-0.302517000
6	0.192136000	5.711436000	0.004272000
6	1.208590000	6.686516000	0.091262000
6	0.934017000	8.040282000	-0.103846000
6	-0.367535000	8.459318000	-0.401088000
6	-1.385270000	7.506524000	-0.502135000
6	0.544989000	4.287724000	0.221016000
6	-0.159689000	3.505568000	1.187094000
7	0.219056000	2.235729000	1.611723000
6	-0.716303000	1.848768000	2.557233000
6	-1.706544000	2.848507000	2.703747000
6	-1.348561000	3.899705000	1.861612000
26	1.864157000	1.230341000	1.079074000
7	2.152607000	2.491297000	-0.414830000
6	2.942379000	2.265978000	-1.528200000
6	2.857980000	3.360247000	-2.420711000
6	2.010032000	4.295490000	-1.829418000
6	1.565473000	3.733526000	-0.597595000
6	3.812807000	1.155015000	-1.692735000
6	4.317115000	0.873047000	-3.056293000
6	3.409585000	0.467604000	-4.059439000
6	3.853261000	0.155284000	-5.346027000
6	5.213651000	0.246458000	-5.655890000
6	6.126482000	0.660930000	-4.683086000
6	5.674740000	0.971765000	-3.397689000
6	-0.585727000	0.739139000	3.442923000
6	-1.715978000	0.468440000	4.367267000
6	-1.572021000	0.613849000	5.761075000
6	-2.635262000	0.397238000	6.637163000
6	-3.883513000	0.018932000	6.133832000
6	-4.060717000	-0.124363000	4.756843000
6	-2.994513000	0.114192000	3.883735000
8	-3.149533000	-0.060639000	2.526181000
6	-4.186469000	0.655500000	1.878254000
6	-4.810626000	0.064483000	0.614730000
7	-4.985515000	-1.273259000	0.398166000
8	2.069979000	0.434774000	-3.739459000
6	1.296973000	-0.707782000	-4.037133000
6	1.660369000	-1.876383000	-3.116353000
8	2.641332000	-1.799693000	-2.401721000
7	1.754167000	0.206547000	2.807861000
6	0.646045000	0.060571000	3.623966000
6	0.951122000	-0.833989000	4.695577000
6	2.278050000	-1.204799000	4.546425000
6	2.747957000	-0.598122000	3.344191000
6	3.977339000	-0.916489000	2.719906000
6	4.863507000	-1.912028000	3.384819000
6	4.442219000	-3.244731000	3.543096000
6	5.284072000	-4.189511000	4.133910000
6	6.557555000	-3.817224000	4.578778000
6	6.986612000	-2.495432000	4.422488000
6	6.147282000	-1.551114000	3.824600000
7	3.664047000	0.428944000	0.657544000
6	4.348037000	-0.488592000	1.430927000
6	5.391201000	-1.097717000	0.654714000
6	5.291185000	-0.593678000	-0.621270000
6	4.228804000	0.366139000	-0.607026000
8	-5.285870000	0.911055000	-0.151473000
1	3.450870000	-3.533339000	3.187232000
1	0.268607000	-1.144099000	5.480152000
1	2.856870000	-1.857061000	5.193740000
1	-2.537844000	2.821570000	3.402237000
1	-1.860841000	4.850046000	1.755057000
1	1.703578000	5.259154000	-2.227287000
1	3.389728000	3.442648000	-3.365760000
1	5.890543000	-0.854791000	-1.488296000
1	6.088159000	-1.846225000	1.021755000
1	-0.605361000	0.931938000	6.153732000
1	-2.490601000	0.530336000	7.711379000
1	-4.721014000	-0.167674000	6.809522000
1	-5.028343000	-0.432219000	4.357342000
1	2.225854000	6.367514000	0.326349000
1	1.740367000	8.773033000	-0.019485000
1	-0.585509000	9.518584000	-0.555598000
1	-2.404339000	7.817280000	-0.745853000
1	-1.923025000	5.435766000	-0.412164000
1	6.383140000	1.301667000	-2.635508000
1	7.188244000	0.746735000	-4.923723000
1	5.553814000	0.002561000	-6.664844000
1	3.137170000	-0.153851000	-6.109325000
1	4.945886000	-5.222869000	4.243402000
1	7.214678000	-4.556336000	5.043247000
1	7.979761000	-2.197257000	4.767205000
1	6.484364000	-0.519938000	3.697380000
1	-5.036168000	0.805385000	2.565939000
1	-3.820721000	1.650646000	1.593762000
1	0.255924000	-0.409667000	-3.866714000
1	1.396978000	-1.002250000	-5.094562000
7	0.820192000	-2.979642000	-3.123064000
19	-1.796441000	-1.857622000	-1.191247000
8	-2.070765000	-2.568402000	-4.006577000
8	-0.699617000	-4.382388000	-0.813067000
8	-4.449653000	-1.784691000	-2.498822000
8	-3.460475000	-4.027720000	0.145271000
6	0.184382000	-5.006279000	-1.727361000
1	-0.399289000	-5.462954000	-2.547501000
6	1.257472000	-4.056076000	-2.218399000
1	0.715034000	-5.833779000	-1.220201000
1	2.038920000	-4.664053000	-2.711794000
1	1.722037000	-3.570678000	-1.353042000
6	-1.437937000	-5.345126000	-0.078206000
1	-1.965757000	-6.021753000	-0.773028000
1	-0.745954000	-5.957407000	0.531549000
6	-2.422384000	-4.701229000	0.864189000
1	-2.865011000	-5.509751000	1.472379000
1	-1.914575000	-3.988701000	1.528440000
6	-4.603555000	-3.723965000	0.941763000

1	-5.495563000	-4.013813000	0.370008000
1	-4.598032000	-4.356628000	1.843620000
6	-4.694474000	-2.282935000	1.432044000
1	-5.500233000	-2.266530000	2.189771000
1	-3.755191000	-2.023565000	1.923628000
6	0.183240000	-3.425942000	-4.372252000
6	-0.986616000	-2.630690000	-4.920755000
1	0.931153000	-3.500813000	-5.182586000
1	-0.172470000	-4.448011000	-4.198884000
1	-1.311664000	-3.142345000	-5.845693000
1	-0.701559000	-1.610420000	-5.215085000
6	-3.126317000	-1.751672000	-4.516178000
1	-3.230346000	-1.914333000	-5.602972000
6	-4.439449000	-2.116297000	-3.878196000
1	-2.879797000	-0.688384000	-4.355501000
1	-5.247687000	-1.573636000	-4.403794000
1	-4.610416000	-3.198227000	-4.007701000
6	-5.991062000	-1.614816000	-0.616932000
1	-6.802407000	-2.183396000	-0.131070000
1	-6.413425000	-0.667696000	-0.965475000
6	-5.532994000	-2.409912000	-1.827145000
1	-6.409473000	-2.468943000	-2.497462000
1	-5.249944000	-3.444361000	-1.583568000
8	-3.246822000	-4.865209000	-2.657566000
1	-2.760430000	-4.147634000	-3.100573000
1	-3.378115000	-4.553869000	-1.744062000
6	1.208876000	-0.313309000	0.094693000
8	1.441601000	-1.461960000	0.413103000
8	0.465593000	-0.104684000	-0.990305000
6	-3.350751000	1.578762000	-2.586991000
1	-4.026664000	2.431947000	-2.728549000
1	-3.904792000	0.634400000	-2.609002000
1	-2.598239000	1.593126000	-3.391556000
6	-2.612767000	1.690099000	-1.288995000
8	-2.288369000	0.761181000	-0.581641000
8	-2.299316000	2.957021000	-0.974976000
1	0.345287000	0.832642000	-1.298103000
8	-4.156508000	4.857901000	-1.728915000
1	-3.914091000	5.730899000	-2.066495000
1	-3.322736000	4.357597000	-1.672894000
8	-1.405669000	-1.845421000	1.421204000
1	-1.716987000	-1.038442000	1.860260000
1	-0.633029000	-2.184961000	1.917011000
8	-5.063319000	3.620680000	0.647055000
1	-5.193069000	2.720279000	0.291644000
1	-4.789458000	4.143524000	-0.129035000
8	0.872592000	-3.101367000	2.444018000
1	1.099790000	-2.655491000	3.277929000
1	1.272975000	-2.520455000	1.759389000
8	0.014478000	1.911758000	-2.602081000
1	0.843186000	1.684158000	-3.070638000
1	0.148523000	2.835662000	-2.333103000
1	-1.840277000	3.000755000	-0.102139000

UB3LYP data:

$^3CAT_{H3O+,K+}$:

6	2.618393000	5.223118000	-1.170136000
6	1.440087000	4.953331000	-1.895669000
6	0.906197000	5.995542000	-2.679838000
6	1.520347000	7.250970000	-2.738519000
6	2.690564000	7.497714000	-2.012154000
6	3.236666000	6.476196000	-1.226283000
6	0.769105000	3.619105000	-1.828416000
6	1.472804000	2.480891000	-2.289825000
7	1.022935000	1.158314000	-2.192913000
6	2.084182000	0.363518000	-2.631128000
6	3.175360000	1.188208000	-3.030162000
6	2.786770000	2.503865000	-2.842055000
26	-0.851116000	0.544590000	-1.765819000
7	-1.326374000	2.415542000	-1.196623000
6	-2.469862000	2.799603000	-0.498739000
6	-2.338045000	4.158450000	-0.059187000
6	-1.136797000	4.628097000	-0.541577000
6	-0.509374000	3.545702000	-1.243148000
6	-3.613469000	2.004450000	-0.310934000
6	-4.730466000	2.502568000	0.545979000
6	-4.998424000	1.880488000	1.795207000
6	-6.061283000	2.303117000	2.603744000
6	-6.877180000	3.362963000	2.187733000
6	-6.627784000	4.000822000	0.971382000
6	-5.565024000	3.566533000	0.168274000
6	2.096101000	-1.055278000	-2.618417000
6	3.394830000	-1.749713000	-2.895442000
6	3.879508000	-1.860112000	-4.209112000
6	5.101710000	-2.471924000	-4.507013000
6	5.867048000	-3.002737000	-3.467664000
6	5.410675000	-2.923410000	-2.147992000
6	4.188326000	-2.300563000	-1.858642000
8	3.664930000	-2.248609000	-0.585949000
6	4.472933000	-2.309484000	0.568827000
6	4.727967000	-0.877768000	1.081732000
7	5.036173000	-0.689315000	2.393597000
8	-4.139352000	0.886702000	2.173564000
6	-4.561977000	-0.152779000	3.040009000
6	-3.814046000	-1.429727000	2.633633000
8	-4.460633000	-2.409600000	2.269752000
7	-0.351643000	-1.342056000	-2.235495000
6	0.936945000	-1.833818000	-2.443135000
6	0.898298000	-3.259318000	-2.581410000
6	-0.424091000	-3.639183000	-2.517297000
6	-1.199535000	-2.450186000	-2.314821000
6	-2.602842000	-2.425149000	-2.191857000
6	-3.360626000	-3.689360000	-2.438180000
6	-4.112403000	-4.312330000	-1.419750000
6	-4.809092000	-5.501932000	-1.654868000
6	-4.774715000	-6.104280000	-2.917898000
6	-4.035700000	-5.501894000	-3.942020000
6	-3.340730000	-4.311916000	-3.703144000
7	-2.728871000	-0.060178000	-1.404921000
6	-3.314140000	-1.272042000	-1.776189000
6	-4.723767000	-1.212085000	-1.563468000
6	-4.992408000	0.015621000	-0.981099000
6	-3.755439000	0.712643000	-0.880573000
8	4.595849000	0.060210000	0.298083000
1	-4.148558000	-3.854194000	-0.428448000
1	1.766013000	-3.896831000	-2.741729000
1	-0.827230000	-4.646213000	-2.596439000
1	4.127985000	0.828315000	-3.412615000
1	3.368794000	3.398749000	-3.049223000
1	-0.712657000	5.620863000	-0.408675000
1	-3.075149000	4.704348000	0.525830000
1	-5.959695000	0.403783000	-0.666304000
1	-5.433585000	-2.002072000	-1.796818000
1	3.272277000	-1.441814000	-5.016063000
1	5.448827000	-2.535164000	-5.541229000
1	6.822828000	-3.491159000	-3.674358000
1	6.017309000	-3.361504000	-1.354631000
1	-0.005235000	5.811720000	-3.254506000
1	1.084956000	8.039009000	-3.359487000
1	3.172604000	8.477921000	-2.057089000
1	4.146604000	6.658044000	-0.647352000
1	3.048738000	4.434937000	-0.547211000
1	-5.380086000	4.052207000	-0.793271000
1	-7.261956000	4.827099000	0.640327000
1	-7.703858000	3.687673000	2.825060000
1	-6.244800000	1.822580000	3.566657000
1	-5.377991000	-5.964897000	-0.843503000
1	-5.319145000	-7.034273000	-3.102068000
1	-4.004252000	-5.957636000	-4.935770000
1	-2.771070000	-3.847436000	-4.512002000
1	3.914542000	-2.899362000	1.306513000
1	5.433375000	-2.819920000	0.403504000
1	-4.391577000	0.111666000	4.099701000
1	-5.630613000	-0.374040000	2.907097000
7	-2.447271000	-1.402634000	2.643083000
19	1.818295000	-0.106328000	0.420122000
8	0.245680000	1.002326000	2.584381000
8	0.386032000	-2.918952000	3.156738000
8	3.014887000	1.612951000	2.529038000
8	3.017340000	-2.124029000	4.141561000
6	-0.938295000	-3.405691000	2.998496000
1	-1.458213000	-3.459920000	3.974605000
6	-1.730512000	-2.524885000	2.035346000
1	-0.899631000	-4.435537000	2.592144000
1	-2.486224000	-3.144437000	1.540919000
1	-1.046072000	-2.148386000	1.256143000
6	1.129912000	-3.640596000	4.118614000
1	0.853565000	-3.320594000	5.142514000
1	0.915738000	-4.724157000	4.039105000
6	2.619179000	-3.468537000	3.906190000
1	3.144513000	-4.144282000	4.605092000
1	2.875882000	-3.780208000	2.877067000
6	4.371477000	-1.912218000	4.505254000
1	4.388758000	-0.995056000	5.111763000
1	4.731670000	-2.734321000	5.150180000
6	5.336856000	-1.780175000	3.322850000
1	6.346650000	-1.620821000	3.739855000
1	5.388493000	-2.727125000	2.773010000
6	-1.645640000	-0.336181000	3.241070000
6	-1.124970000	0.730916000	2.260693000
1	-2.213210000	0.138268000	4.051301000
1	-0.774970000	-0.808617000	3.715146000
1	-1.714833000	1.655210000	2.334316000
1	-1.209706000	0.383217000	1.216878000
6	0.711449000	2.320885000	2.317784000
1	-0.005271000	3.067286000	2.705383000
6	2.037969000	2.521975000	3.020220000
1	0.800689000	2.498558000	1.228921000
1	2.370045000	3.567100000	2.868035000
1	1.892456000	2.367783000	4.103964000
6	5.270147000	0.691461000	2.836548000
1	6.068612000	0.674279000	3.594683000
1	5.636904000	1.260982000	1.972509000
6	4.082312000	1.430168000	3.449481000
1	4.465702000	2.413684000	3.783708000
1	3.694025000	0.908627000	4.341726000
8	1.293494000	-0.095115000	5.074581000

1	0.946809000	0.290091000	4.250098000
1	1.838320000	-0.839812000	4.755117000

^{3}RC1$_{H3O+,K+}$:

6	1.223132000	1.393537000	-5.534236000
6	-0.024817000	0.750993000	-5.433097000
6	-0.830642000	0.684885000	-6.585632000
6	-0.400465000	1.231085000	-7.798511000
6	0.845933000	1.862171000	-7.883793000
6	1.655971000	1.941810000	-6.746464000
6	-0.494510000	0.162847000	-4.138883000
6	0.162852000	-0.942703000	-3.615880000
7	-0.142190000	-1.597093000	-2.432449000
6	0.797882000	-2.600223000	-2.300049000
6	1.711980000	-2.581129000	-3.428324000
6	1.305601000	-1.582764000	-4.252167000
26	-1.609431000	-1.145906000	-1.113474000
7	-2.183745000	0.387744000	-2.302370000
6	-3.103671000	1.360652000	-1.967132000
6	-3.081445000	2.414207000	-2.928646000
6	-2.126462000	2.057622000	-3.885127000
6	-1.576668000	0.815832000	-3.475506000
6	-3.937368000	1.352645000	-0.812253000
6	-4.719035000	2.591539000	-0.499762000
6	-4.166535000	3.615537000	0.300136000
6	-4.888008000	4.771663000	0.614821000
6	-6.194486000	4.922702000	0.142078000
6	-6.766006000	3.929144000	-0.656828000
6	-6.027521000	2.785074000	-0.974157000
6	0.847282000	-3.555098000	-1.292448000
6	2.030684000	-4.468738000	-1.201663000
6	1.948860000	-5.805277000	-1.616029000
6	3.024687000	-6.690764000	-1.484614000
6	4.210627000	-6.236483000	-0.907835000
6	4.328512000	-4.907917000	-0.483664000
6	3.256295000	-4.016634000	-0.647007000
8	3.303767000	-2.699648000	-0.289554000
6	4.479695000	-2.127626000	0.224535000
6	4.528837000	-0.647865000	-0.193036000
7	5.539855000	0.132006000	0.306807000
8	-2.850240000	3.471867000	0.732313000
6	-2.491239000	3.792323000	2.086421000
6	-1.823018000	2.547621000	2.694458000
8	-2.481605000	1.514989000	2.709721000
7	-1.344653000	-2.964375000	-0.267289000
6	-0.204651000	-3.741220000	-0.355524000
6	-0.251648000	-4.801669000	0.597476000
6	-1.452792000	-4.680067000	1.269232000
6	-2.108485000	-3.531203000	0.737596000
6	-3.324157000	-3.007033000	1.258625000
6	-4.049209000	-3.827568000	2.281067000
6	-4.110459000	-3.432847000	3.630162000
6	-4.786482000	-4.208616000	4.578128000
6	-5.413581000	-5.398769000	4.195451000
6	-5.357391000	-5.807543000	2.858120000
6	-4.679972000	-5.030880000	1.913332000
7	-3.270755000	-0.884522000	-0.016643000
6	-3.823450000	-1.759811000	0.911263000
6	-5.003958000	-1.159893000	1.510641000
6	-5.122745000	0.090020000	0.996617000
6	-4.059941000	0.247487000	0.018956000
8	3.724447000	-0.211124000	-1.008808000
1	-3.616613000	-2.507884000	3.938285000
1	0.516068000	-5.557354000	0.742047000
1	-1.833237000	-5.310545000	2.069080000
1	2.534047000	-3.278698000	-3.574332000
1	1.730025000	-1.294944000	-5.210707000

1	-1.840707000	2.616379000	-4.772449000
1	-3.738471000	3.281557000	-2.935129000
1	-5.883077000	0.835548000	1.217575000
1	-5.647999000	-1.642404000	2.241561000
1	1.008321000	-6.154703000	-2.049407000
1	2.930283000	-7.725667000	-1.821551000
1	5.058283000	-6.914107000	-0.777166000
1	5.260076000	-4.583830000	-0.020072000
1	-1.804176000	0.191314000	-6.527913000
1	-1.040266000	1.160205000	-8.682268000
1	1.183012000	2.290052000	-8.831488000
1	2.629301000	2.436940000	-6.800263000
1	1.858830000	1.465826000	-4.648115000
1	-6.472782000	2.005159000	-1.596740000
1	-7.784940000	4.043467000	-1.034032000
1	-6.757195000	5.825292000	0.391960000
1	-4.427814000	5.556558000	1.218139000
1	-4.819125000	-3.882597000	5.621216000
1	-5.942078000	-6.005678000	4.935185000
1	-5.846136000	-6.734974000	2.547643000
1	-4.641258000	-5.355949000	0.870504000
1	4.515903000	-2.263158000	1.317515000
1	5.387475000	-2.593502000	-0.195953000
1	-1.898561000	4.713669000	2.093745000
1	-3.403038000	3.966208000	2.674876000
7	-0.559566000	2.600921000	3.219984000
19	2.300921000	1.123201000	1.163203000
8	1.931762000	3.908156000	1.657873000
8	2.226346000	1.062341000	3.983927000
8	4.182030000	2.920255000	0.072899000
8	5.041341000	1.021695000	3.060501000
6	1.057879000	1.289233000	4.760564000
1	1.177426000	2.187281000	5.395030000
6	-0.197113000	1.338097000	3.914517000
1	0.924144000	0.438547000	5.454733000
1	-1.038488000	1.083988000	4.578135000
1	-0.158239000	0.529695000	3.167099000
6	3.369492000	0.839118000	4.799865000
1	3.731640000	1.797395000	5.212978000
1	3.097893000	0.180627000	5.645878000
6	4.478424000	0.148498000	4.036193000
1	5.255353000	-0.145984000	4.764440000
1	4.089136000	-0.772708000	3.564721000
6	6.353107000	0.704512000	2.613600000
1	6.829693000	1.658403000	2.350333000
1	6.945341000	0.266286000	3.436282000
6	6.399243000	-0.263673000	1.421533000
1	7.449645000	-0.326925000	1.085501000
1	6.122364000	-1.267507000	1.759789000
6	0.193639000	3.851764000	3.388086000
6	0.736709000	4.522313000	2.127570000
1	-0.422906000	4.594959000	3.918864000
1	1.046517000	3.653396000	4.047766000
1	0.950439000	5.577580000	2.380852000
1	-0.006465000	4.531588000	1.314163000
6	2.391715000	4.501357000	0.437557000
1	2.210046000	5.590211000	0.461765000
6	3.875947000	4.293490000	0.258552000
1	1.825258000	4.083825000	-0.414756000
1	4.199315000	4.877671000	-0.624684000
1	4.411041000	4.683770000	1.142424000
6	5.932959000	1.306973000	-0.485768000
1	7.030451000	1.277117000	-0.593586000
1	5.492414000	1.182918000	-1.483214000
6	5.580672000	2.701441000	0.017942000
1	6.041133000	3.409853000	-0.697910000
1	6.027432000	2.915729000	1.003731000

8	4.225913000	3.761732000	3.506260000
1	3.413071000	3.802759000	2.972912000
1	4.568929000	2.866295000	3.330029000
6	-0.295047000	-0.130311000	-0.048993000
8	0.165268000	-0.729042000	0.931611000
8	0.050488000	1.041973000	-0.411763000
8	-0.990899000	3.139248000	-1.061233000
1	-1.696013000	3.301456000	-0.347603000
1	-0.599045000	2.262295000	-0.866801000
1	-1.519366000	3.044520000	-1.909789000

^{3}IM1 $_{H_3O^+,K^+}$:

6	-0.344012000	0.578974000	-5.932617000
6	-1.314651000	-0.324941000	-5.464937000
6	-2.211648000	-0.882184000	-6.393670000
6	-2.138118000	-0.549703000	-7.750213000
6	-1.166231000	0.349128000	-8.202353000
6	-0.269499000	0.913084000	-7.288611000
6	-1.405087000	-0.680086000	-4.010607000
6	-0.416660000	-1.500780000	-3.453438000
7	-0.365534000	-1.957495000	-2.149115000
6	0.757340000	-2.757479000	-2.071719000
6	1.457468000	-2.758998000	-3.343228000
6	0.717862000	-2.010372000	-4.202683000
26	-1.570968000	-1.473791000	-0.631042000
7	-2.684145000	-0.329423000	-1.923157000
6	-3.690122000	0.529131000	-1.558100000
6	-4.155616000	1.264337000	-2.704632000
6	-3.403856000	0.844266000	-3.773728000
6	-2.468737000	-0.128108000	-3.264347000
6	-4.220208000	0.666026000	-0.257650000
6	-5.178448000	1.791417000	-0.005740000
6	-4.706523000	3.103173000	0.227264000
6	-5.602986000	4.151682000	0.474267000
6	-6.978050000	3.907291000	0.508079000
6	-7.465414000	2.618578000	0.275820000
6	-6.566731000	1.579869000	0.016877000
6	1.139266000	-3.543442000	-0.976562000
6	2.460559000	-4.252097000	-1.045696000
6	2.616824000	-5.433544000	-1.783197000
6	3.860628000	-6.061275000	-1.911816000
6	4.976564000	-5.492659000	-1.295842000
6	4.850570000	-4.320671000	-0.542486000
6	3.597946000	-3.707065000	-0.400676000
8	3.370778000	-2.610720000	0.382246000
6	4.403820000	-1.767097000	0.821912000
6	4.685739000	-0.668354000	-0.225854000
7	5.542394000	0.354755000	0.108866000
8	-3.352078000	3.337205000	0.123839000
6	-2.668659000	4.152838000	1.074359000
6	-1.895460000	3.230890000	2.031611000
8	-2.509453000	2.283892000	2.513415000
7	-0.860727000	-3.094153000	0.408869000
6	0.326954000	-3.733465000	0.158433000
6	0.627713000	-4.658199000	1.222088000
6	-0.396934000	-4.565220000	2.124769000
6	-1.304577000	-3.562877000	1.618011000
6	-2.410604000	-3.057018000	2.336031000
6	-2.762129000	-3.722219000	3.633099000
6	-2.525272000	-3.088368000	4.866305000
6	-2.847549000	-3.720078000	6.071623000
6	-3.413325000	-4.999530000	6.066357000
6	-3.652391000	-5.642685000	4.847300000
6	-3.328026000	-5.009965000	3.642999000
7	-2.984356000	-1.226675000	0.755991000
6	-3.156580000	-1.944042000	1.924413000
6	-4.240100000	-1.368120000	2.703500000
6	-4.690910000	-0.285773000	2.018344000
6	-3.933015000	-0.223474000	0.782822000
8	4.133823000	-0.727659000	-1.316560000
1	-2.077638000	-2.091495000	4.878503000
1	1.505407000	-5.299164000	1.269804000
1	-0.512661000	-5.108320000	3.059526000
1	2.377336000	-3.298621000	-3.551566000
1	0.913048000	-1.816565000	-5.254187000
1	-3.473177000	1.175773000	-4.806976000
1	-4.956278000	2.000740000	-2.701180000
1	-5.488841000	0.396629000	2.298259000
1	-4.599739000	-1.749371000	3.655594000
1	1.739142000	-5.859271000	-2.276159000
1	3.955169000	-6.980051000	-2.494991000
1	5.959637000	-5.960367000	-1.393074000
1	5.736700000	-3.899742000	-0.066883000
1	-2.971562000	-1.587257000	-6.047206000
1	-2.842183000	-0.997179000	-8.456683000
1	-1.108388000	0.609686000	-9.262263000
1	0.490864000	1.619712000	-7.631481000
1	0.355929000	1.026971000	-5.222743000
1	-6.941945000	0.570187000	-0.167186000
1	-8.539808000	2.421624000	0.291518000
1	-7.666764000	4.733250000	0.702079000
1	-5.226870000	5.165434000	0.625657000
1	-2.651968000	-3.211002000	7.019078000
1	-3.665781000	-5.493498000	7.008125000
1	-4.095893000	-6.641758000	4.832088000
1	-3.519572000	-5.517180000	2.694005000
1	4.048777000	-1.325685000	1.764332000
1	5.330796000	-2.312036000	1.060485000
1	-2.058742000	4.870315000	0.515271000
1	-3.378696000	4.726503000	1.689486000
7	-0.591769000	3.485483000	2.349610000
19	2.526262000	1.532051000	0.877620000
8	1.833118000	4.142910000	0.117146000
8	2.376087000	2.635489000	3.438274000
8	4.105480000	2.716342000	-1.092351000
8	5.090098000	2.258238000	2.429001000
6	1.183619000	2.972905000	4.135990000
1	1.166851000	4.049156000	4.391252000
6	-0.048889000	2.552507000	3.361608000
1	1.166829000	2.421147000	5.094030000
1	-0.854074000	2.385651000	4.090496000
1	0.134966000	1.569758000	2.893771000
6	3.553924000	2.896851000	4.189061000
1	3.851554000	3.953867000	4.071059000
1	3.367035000	2.709305000	5.262150000
6	4.682229000	1.980399000	3.769485000
1	5.530055000	2.148244000	4.457336000
1	4.365663000	0.926098000	3.867711000
6	6.362807000	1.734337000	2.058720000
1	6.843373000	2.481899000	1.412695000
1	7.003418000	1.628148000	2.950516000
6	6.305128000	0.373756000	1.361418000
1	7.348973000	0.064212000	1.166630000
1	5.884994000	-0.360878000	2.056919000
6	0.150389000	4.646228000	1.839896000
6	0.519549000	4.654479000	0.350288000
1	-0.422560000	5.562040000	2.051623000
1	1.079233000	4.744706000	2.414938000
1	0.489709000	5.701935000	-0.003804000
1	-0.197521000	4.076362000	-0.251776000
6	2.155607000	4.138144000	-1.276896000
1	1.840409000	5.094206000	-1.732894000
6	3.645010000	4.000615000	-1.490564000
1	1.601810000	3.327184000	-1.783467000

1	3.855416000	4.161667000	-2.565317000
1	4.171292000	4.784593000	-0.918112000
6	6.018239000	1.202448000	-0.995766000
1	7.120229000	1.235850000	-0.956653000
1	5.728542000	0.699137000	-1.926090000
6	5.521421000	2.644551000	-1.054399000
1	5.948768000	3.082782000	-1.976617000
1	5.893303000	3.252841000	-0.212048000
8	4.199127000	4.933275000	1.657422000
1	3.357107000	4.724979000	1.214542000
1	4.569200000	4.060873000	1.882777000
6	-0.192299000	-0.048823000	0.249585000
8	0.533044000	-0.325797000	1.212330000
8	0.063551000	1.223786000	-0.245263000
8	-1.448972000	2.762172000	-1.920302000
1	-2.208162000	2.917202000	-1.323176000
1	-0.556577000	1.501854000	-0.956571000
1	-1.864949000	2.420843000	-2.727688000

^{3}RC2$_{H3O+,K+}$:

6	0.843891000	6.077620000	0.106775000
6	-0.307508000	5.690329000	-0.603600000
6	-1.204358000	6.691172000	-1.021322000
6	-0.953483000	8.038500000	-0.745265000
6	0.196062000	8.409726000	-0.039815000
6	1.092859000	7.424468000	0.386941000
6	-0.588944000	4.250466000	-0.899547000
6	0.291726000	3.541193000	-1.724843000
7	0.216071000	2.184258000	-2.013597000
6	1.365531000	1.877671000	-2.712367000
6	2.168886000	3.068870000	-2.897023000
6	1.492964000	4.098627000	-2.317165000
26	-1.277879000	0.916502000	-1.577374000
7	-2.199801000	2.395007000	-0.521379000
6	-3.234183000	2.185382000	0.354024000
6	-3.400324000	3.341877000	1.203442000
6	-2.462288000	4.266380000	0.789204000
6	-1.715069000	3.657351000	-0.273465000
6	-4.053378000	1.035531000	0.395758000
6	-5.107355000	0.930498000	1.459208000
6	-4.856754000	0.284368000	2.686961000
6	-5.842712000	0.167368000	3.669974000
6	-7.120930000	0.676204000	3.429751000
6	-7.399111000	1.323207000	2.222912000
6	-6.397370000	1.451413000	1.258081000
6	1.704880000	0.616724000	-3.222219000
6	3.078470000	0.398602000	-3.780507000
6	3.287386000	0.392136000	-5.172204000
6	4.546721000	0.153414000	-5.724579000
6	5.632588000	-0.103777000	-4.881708000
6	5.451656000	-0.107715000	-3.498197000
6	4.188352000	0.150715000	-2.947123000
8	4.021006000	0.122559000	-1.570641000
6	4.734989000	1.142049000	-0.875868000
6	4.691963000	1.065136000	0.658959000
7	5.249051000	-0.006075000	1.327500000
8	-3.549355000	-0.155194000	2.947481000
6	-3.294977000	-1.438293000	3.576222000
6	-2.679268000	-2.376797000	2.526134000
8	-3.351143000	-2.598372000	1.522974000
7	-0.531662000	-0.396811000	-2.918443000
6	0.778989000	-0.438517000	-3.335128000
6	1.042514000	-1.686059000	-4.010227000
6	-0.138954000	-2.374699000	-4.037411000
6	-1.110252000	-1.567274000	-3.341767000
6	-2.436495000	-1.973536000	-3.075669000
6	-2.931585000	-3.223827000	-3.734307000
6	-3.214867000	-4.379274000	-2.982727000
6	-3.664797000	-5.546952000	-3.606222000
6	-3.841930000	-5.580548000	-4.993586000
6	-3.562794000	-4.439415000	-5.753030000
6	-3.108871000	-3.273330000	-5.129152000
7	-2.927526000	-0.186270000	-1.433612000
6	-3.270116000	-1.301066000	-2.173935000
6	-4.596076000	-1.758998000	-1.795125000
6	-5.005071000	-0.979007000	-0.762587000
6	-3.967111000	0.010907000	-0.549489000
8	4.274955000	2.050113000	1.257527000
1	-3.071978000	-4.361182000	-1.899572000
1	1.998495000	-1.990260000	-4.428165000
1	-0.327071000	-3.353969000	-4.470352000
1	3.109529000	3.110648000	-3.441486000
1	1.780063000	5.146303000	-2.286197000
1	-2.302345000	5.265697000	1.186079000
1	-4.191814000	3.486835000	1.937506000
1	-5.936666000	-1.038921000	-0.206067000
1	-5.128278000	-2.588426000	-2.253123000
1	2.434111000	0.583287000	-5.827329000
1	4.678259000	0.162119000	-6.809010000
1	6.621720000	-0.307512000	-5.298868000
1	6.294291000	-0.320578000	-2.836960000
1	-2.102307000	6.408304000	-1.576127000
1	-1.658795000	8.801235000	-1.085138000
1	0.391517000	9.462739000	0.178038000
1	1.990007000	7.704184000	0.945175000
1	1.543876000	5.312662000	0.451347000
1	-6.611990000	1.955944000	0.313212000
1	-8.394362000	1.730881000	2.032668000
1	-7.891718000	0.579045000	4.197532000
1	-5.612848000	-0.305339000	4.626401000
1	-3.874115000	-6.435525000	-3.004986000
1	-4.194798000	-6.492932000	-5.481022000
1	-3.700463000	-4.455091000	-6.837304000
1	-2.894959000	-2.384636000	-5.728049000
1	5.800040000	1.143198000	-1.170227000
1	4.308025000	2.124909000	-1.122893000
1	-2.701676000	-1.254114000	4.477523000
1	-4.250824000	-1.882785000	3.879522000
7	-1.454383000	-2.934655000	2.723699000
19	2.203368000	-0.990711000	1.777203000
8	1.192759000	-1.268768000	4.431534000
8	1.442170000	-3.786446000	1.474528000
8	3.721676000	-0.003625000	3.919055000
8	4.290008000	-2.895307000	1.604816000
6	0.274204000	-4.565392000	1.721685000
1	0.350486000	-5.082387000	2.697116000
6	-1.007696000	-3.767555000	1.586744000
1	0.217454000	-5.359925000	0.954931000
1	-1.812992000	-4.487184000	1.373681000
1	-0.927233000	-3.113723000	0.705489000
6	2.587884000	-4.604366000	1.281263000
1	2.898562000	-5.062568000	2.238119000
1	2.344389000	-5.424401000	0.579261000
6	3.740844000	-3.833611000	0.673892000
1	4.516280000	-4.566857000	0.392729000
1	3.411938000	-3.318941000	-0.247588000
6	5.641971000	-2.497682000	1.361952000
1	6.143908000	-2.458509000	2.338976000
1	6.158559000	-3.272261000	0.771453000
6	5.796136000	-1.169627000	0.617794000
1	6.879598000	-1.031075000	0.438246000
1	5.301775000	-1.245055000	-0.357272000
6	-0.652141000	-2.764526000	3.937991000
6	-0.222943000	-1.320610000	4.265529000

1	-1.172880000	-3.210019000	4.800319000
1	0.266586000	-3.347120000	3.811502000
1	-0.696052000	-0.975546000	5.201598000
1	-0.518890000	-0.642857000	3.447370000
6	1.641272000	-0.090746000	5.099395000
1	1.202910000	-0.042256000	6.113009000
6	3.146508000	-0.119545000	5.211534000
1	1.315415000	0.809532000	4.545217000
1	3.475030000	0.717467000	5.856277000
1	3.462673000	-1.063178000	5.690979000
6	5.782445000	0.285428000	2.671677000
1	6.840894000	-0.026376000	2.691016000
1	5.748961000	1.374863000	2.792356000
6	5.098337000	-0.339873000	3.881674000
1	5.616359000	0.062588000	4.772356000
1	5.206573000	-1.437601000	3.917492000
8	3.239394000	-3.405083000	4.348305000
1	2.513864000	-2.759807000	4.407425000
1	3.642617000	-3.248657000	3.476703000
6	-0.229197000	0.227981000	0.222339000
8	-0.379242000	-0.748470000	0.951733000
8	0.809467000	1.066222000	0.663130000
8	-1.955473000	1.739538000	3.164552000
1	-2.616838000	0.903364000	3.099952000
1	0.895771000	1.759634000	-0.011373000
1	-2.270188000	2.436249000	2.509572000
1	-1.976310000	2.122659000	4.062896000

^{3}TS2 $_{H3O+,K+}$:

6	1.728129000	5.837164000	-1.361342000
6	0.555252000	5.427992000	-2.023387000
6	-0.193014000	6.401428000	-2.713168000
6	0.223360000	7.734987000	-2.750530000
6	1.395114000	8.125379000	-2.092348000
6	2.144863000	7.171739000	-1.396364000
6	0.094856000	4.005938000	-1.990607000
6	0.888700000	3.008649000	-2.554724000
7	0.613961000	1.650572000	-2.535302000
6	1.732398000	1.017063000	-3.033432000
6	2.729952000	2.002819000	-3.403306000
6	2.195416000	3.227609000	-3.150181000
26	-1.110673000	0.724661000	-1.967681000
7	-1.783451000	2.504663000	-1.292404000
6	-2.864801000	2.653100000	-0.443936000
6	-2.873457000	3.969762000	0.117230000
6	-1.795018000	4.640922000	-0.446318000
6	-1.125745000	3.720587000	-1.302051000
6	-3.845595000	1.664655000	-0.191744000
6	-4.859961000	1.934801000	0.876333000
6	-4.600941000	1.572639000	2.215125000
6	-5.528091000	1.826394000	3.230270000
6	-6.750452000	2.426913000	2.915240000
6	-7.033856000	2.790701000	1.596926000
6	-6.089305000	2.551006000	0.594738000
6	1.874903000	-0.354278000	-3.241146000
6	3.223787000	-0.899372000	-3.592861000
6	3.559958000	-1.207209000	-4.924065000
6	4.811131000	-1.726696000	-5.261917000
6	5.757298000	-1.962352000	-4.259040000
6	5.446162000	-1.672518000	-2.929708000
6	4.191341000	-1.143161000	-2.597601000
8	3.887984000	-0.896257000	-1.265840000
6	4.527018000	0.254807000	-0.719520000
6	4.385410000	0.414012000	0.804322000
7	5.019461000	-0.462082000	1.649171000
8	-3.359481000	1.004818000	2.500931000
6	-3.232545000	-0.007970000	3.506353000
6	-2.550562000	-1.241547000	2.886392000
8	-2.864288000	-1.553474000	1.749541000
7	-0.529812000	-0.902353000	-2.945836000
6	0.771115000	-1.245373000	-3.251897000
6	0.826083000	-2.597348000	-3.714577000
6	-0.467393000	-3.069329000	-3.730470000
6	-1.298246000	-2.017255000	-3.230850000
6	-2.679004000	-2.159056000	-2.960939000
6	-3.354612000	-3.413870000	-3.424311000
6	-3.732012000	-4.420225000	-2.517449000
6	-4.353973000	-5.590443000	-2.964576000
6	-4.608472000	-5.775158000	-4.327557000
6	-4.234346000	-4.783337000	-5.240575000
6	-3.610549000	-3.614840000	-4.792572000
7	-2.915485000	-0.024342000	-1.731017000
6	-3.400798000	-1.229512000	-2.216406000
6	-4.755863000	-1.441212000	-1.739840000
6	-5.046262000	-0.423846000	-0.888991000
6	-3.905000000	0.468686000	-0.893730000
8	3.813058000	1.416296000	1.228330000
1	-3.529776000	-4.285045000	-1.452057000
1	1.726820000	-3.122797000	-4.021393000
1	-0.811693000	-4.058195000	-4.022179000
1	3.694804000	1.776708000	-3.851839000
1	2.642288000	4.199826000	-3.340868000
1	-1.502298000	5.674323000	-0.279455000
1	-3.639932000	4.375681000	0.774369000
1	-5.962513000	-0.261237000	-0.326633000
1	-5.387291000	-2.283777000	-2.009285000
1	2.817904000	-1.024615000	-5.705071000
1	5.046278000	-1.948843000	-6.305476000
1	6.737100000	-2.376742000	-4.508536000
1	6.176360000	-1.862157000	-2.139973000
1	-1.107355000	6.103662000	-3.232523000
1	-0.368761000	8.472091000	-3.299230000
1	1.720493000	9.168422000	-2.119952000
1	3.057182000	7.467583000	-0.871835000
1	2.312910000	5.101123000	-0.804339000
1	-6.303707000	2.837226000	-0.437620000
1	-7.985842000	3.264618000	1.347760000
1	-7.473987000	2.619728000	3.710655000
1	-5.298195000	1.570558000	4.265752000
1	-4.637507000	-6.362203000	-2.244219000
1	-5.095102000	-6.689245000	-4.677194000
1	-4.430184000	-4.918112000	-6.307539000
1	-3.321175000	-2.843172000	-5.510388000
1	5.606410000	0.246235000	-0.957142000
1	4.084972000	1.166072000	-1.148102000
1	-2.725885000	0.424982000	4.373936000
1	-4.230365000	-0.343037000	3.828175000
7	-1.692539000	-1.987522000	3.665335000
19	1.642924000	-0.660021000	2.376097000
8	0.787722000	-0.752101000	5.202515000
8	0.843692000	-3.392031000	2.424475000
8	3.498009000	0.000991000	4.336965000
8	3.690413000	-3.002687000	2.252719000
6	-0.088993000	-3.968086000	3.335656000
1	0.319954000	-3.898930000	4.358379000
6	-1.481612000	-3.376312000	3.214315000
1	-0.208144000	-5.042882000	3.115085000
1	-2.157008000	-4.034243000	3.792959000
1	-1.798430000	-3.419717000	2.166989000
6	1.741556000	-4.348347000	1.874601000
1	2.104470000	-5.029069000	2.665411000
1	1.228336000	-4.959374000	1.107898000
6	2.936356000	-3.671674000	1.240906000
1	3.552981000	-4.451563000	0.761306000

1	2.626684000	-2.962995000	0.451470000
6	5.098127000	-2.931845000	2.059666000
1	5.545669000	-2.879277000	3.061295000
1	5.468001000	-3.861235000	1.591909000
6	5.549527000	-1.747479000	1.196605000
1	6.655044000	-1.732516000	1.199109000
1	5.216465000	-1.915642000	0.168123000
6	-1.483001000	-1.724959000	5.099015000
6	-0.591208000	-0.553974000	5.497253000
1	-2.449306000	-1.580117000	5.611132000
1	-1.056688000	-2.636794000	5.538115000
1	-0.711208000	-0.427823000	6.589495000
1	-0.918419000	0.391060000	5.030605000
6	1.569663000	0.315343000	5.746708000
1	1.299955000	0.465832000	6.807792000
6	3.044642000	0.011882000	5.682177000
1	1.346341000	1.256246000	5.208607000
1	3.577868000	0.795444000	6.253698000
1	3.251379000	-0.959804000	6.163394000
6	5.496031000	0.069586000	2.935931000
1	6.570689000	-0.172662000	3.007173000
1	5.398703000	1.161724000	2.886005000
6	4.855784000	-0.397500000	4.236620000
1	5.442645000	0.075920000	5.046322000
1	4.929166000	-1.487172000	4.385237000
8	2.676588000	-3.158201000	5.031250000
1	1.983784000	-2.479637000	5.090990000
1	2.981689000	-3.118407000	4.106674000
6	-0.246225000	0.251293000	-0.265356000
8	-0.176986000	-0.821114000	0.275141000
8	0.355646000	1.323838000	0.478847000
8	-1.277844000	2.477065000	2.136080000
1	-2.085363000	1.858427000	2.293516000
1	0.576291000	2.031479000	-0.155284000
1	-0.597469000	1.990780000	1.540595000
1	-1.639506000	3.229628000	1.586460000

^{3}IM2 $_{H_3O^+,K^+}$:

6	0.715319000	6.740960000	-1.088815000
6	-0.208726000	6.099322000	-1.931864000
6	-1.016105000	6.887425000	-2.770087000
6	-0.899528000	8.281209000	-2.769159000
6	0.024323000	8.908719000	-1.927119000
6	0.830723000	8.134563000	-1.085957000
6	-0.344520000	4.604586000	-1.937182000
6	0.655520000	3.844805000	-2.569174000
7	0.689212000	2.471749000	-2.668221000
6	1.930369000	2.147429000	-3.173734000
6	2.688038000	3.353126000	-3.422948000
6	1.883637000	4.402767000	-3.089766000
26	-0.698063000	1.179592000	-1.963660000
7	-1.755005000	2.706005000	-1.181850000
6	-2.851775000	2.608888000	-0.352765000
6	-3.218178000	3.917515000	0.136549000
6	-2.355362000	4.808429000	-0.436303000
6	-1.436377000	4.043447000	-1.250143000
6	-3.581765000	1.437877000	-0.093623000
6	-4.773163000	1.499097000	0.820291000
6	-4.662469000	1.256250000	2.207649000
6	-5.799609000	1.270920000	3.025383000
6	-7.058808000	1.518290000	2.473484000
6	-7.188242000	1.771067000	1.105819000
6	-6.049272000	1.763342000	0.296719000
6	2.396739000	0.840353000	-3.412301000
6	3.827583000	0.632265000	-3.808399000
6	4.282633000	1.111253000	-5.053556000
6	5.589881000	0.913601000	-5.496789000
6	6.490893000	0.216425000	-4.684908000
6	6.072834000	-0.267743000	-3.447608000
6	4.755584000	-0.072641000	-3.000887000
8	4.468886000	-0.672693000	-1.799957000
6	3.629531000	-0.063433000	-0.826736000
6	4.213448000	-0.188983000	0.589648000
7	4.505605000	-1.429341000	1.111206000
8	-3.393830000	1.110165000	2.730559000
6	-3.109229000	0.208231000	3.789529000
6	-2.711801000	-1.167051000	3.227377000
8	-3.064110000	-1.453177000	2.090556000
7	0.267772000	-0.315555000	-2.876263000
6	1.566960000	-0.290667000	-3.336643000
6	1.951312000	-1.599696000	-3.808896000
6	0.863627000	-2.407998000	-3.655674000
6	-0.180654000	-1.602193000	-3.064271000
6	-1.444071000	-2.099435000	-2.696823000
6	-1.779401000	-3.514364000	-3.058288000
6	-1.891611000	-4.506488000	-2.067353000
6	-2.196817000	-5.827462000	-2.409074000
6	-2.397805000	-6.179083000	-3.748141000
6	-2.289665000	-5.201600000	-4.742882000
6	-1.981137000	-3.881300000	-4.400869000
7	-2.206369000	-0.060916000	-1.510107000
6	-2.379969000	-1.350072000	-1.966969000
6	-3.658487000	-1.863579000	-1.523736000
6	-4.222157000	-0.903465000	-0.741848000
6	-3.318485000	0.222964000	-0.745250000
8	4.373529000	0.842985000	1.233326000
1	-1.732137000	-4.238143000	-1.020130000
1	2.919288000	-1.857002000	-4.230913000
1	0.774924000	-3.459848000	-3.914181000
1	3.699365000	3.394440000	-3.816964000
1	2.113053000	5.462671000	-3.160743000
1	-2.337137000	5.888642000	-0.316149000
1	-4.050899000	4.128041000	0.803761000
1	-5.171045000	-0.941422000	-0.215168000
1	-4.060927000	-2.842736000	-1.768589000
1	3.570291000	1.635258000	-5.694862000
1	5.899533000	1.294140000	-6.472668000
1	7.520307000	0.051122000	-5.012138000
1	6.756445000	-0.811798000	-2.792671000
1	-1.738753000	6.401908000	-3.430812000
1	-1.533006000	8.878081000	-3.430354000
1	0.115108000	9.997868000	-1.925499000
1	1.552545000	8.616826000	-0.421782000
1	1.344629000	6.140688000	-0.427032000
1	-6.142710000	1.953366000	-0.775333000
1	-8.168824000	1.973435000	0.669369000
1	-7.937098000	1.526144000	3.123449000
1	-5.706288000	1.106912000	4.100030000
1	-2.275229000	-6.585020000	-1.625048000
1	-2.637528000	-7.211342000	-4.015435000
1	-2.448281000	-5.466235000	-5.791467000
1	-1.901109000	-3.121712000	-5.182434000
1	3.547668000	1.015264000	-1.000494000
1	2.617940000	-0.501144000	-0.868566000
1	-2.319309000	0.671965000	4.388329000
1	-3.976568000	0.061464000	4.453560000
7	-2.046915000	-2.054002000	4.039258000
19	1.255327000	-1.408468000	2.376917000
8	0.837540000	-1.369304000	5.197958000
8	-0.138470000	-3.856068000	2.420865000
8	3.541119000	-1.162489000	3.952885000
8	2.641673000	-3.816225000	1.627010000
6	-0.927052000	-4.300005000	3.522538000
1	-0.304644000	-4.313096000	4.433581000

6	-2.190948000	-3.478144000	3.693237000
1	-1.261401000	-5.338171000	3.350953000
1	-2.788388000	-3.967099000	4.483948000
1	-2.766558000	-3.518945000	2.762158000
6	0.504871000	-4.917755000	1.727042000
1	0.948901000	-5.631167000	2.443786000
1	-0.226726000	-5.469124000	1.106779000
6	1.598282000	-4.384542000	0.829075000
1	1.993552000	-5.225960000	0.235411000
1	1.196379000	-3.636282000	0.121010000
6	3.963723000	-3.899196000	1.094640000
1	4.636064000	-4.107377000	1.938372000
1	4.037808000	-4.762972000	0.413031000
6	4.422351000	-2.664063000	0.321658000
1	5.415692000	-2.905163000	-0.100561000
1	3.754220000	-2.502524000	-0.531122000
6	-1.611347000	-1.735753000	5.407118000
6	-0.414202000	-0.804567000	5.589359000
1	-2.441936000	-1.292164000	5.982647000
1	-1.381734000	-2.687985000	5.905073000
1	-0.374343000	-0.561808000	6.667164000
1	-0.553820000	0.148281000	5.050721000
6	1.916900000	-0.531910000	5.629111000
1	1.839195000	-0.368659000	6.719370000
6	3.265669000	-1.150126000	5.347611000
1	1.843296000	0.457286000	5.138546000
1	4.028072000	-0.546781000	5.876098000
1	3.304495000	-2.174775000	5.756207000
6	5.345230000	-1.440672000	2.318521000
1	6.216698000	-2.091830000	2.131905000
1	5.711466000	-0.415762000	2.451415000
6	4.716281000	-1.896502000	3.634561000
1	5.485254000	-1.732544000	4.412089000
1	4.480513000	-2.973012000	3.644470000
8	2.213351000	-4.141290000	4.594945000
1	1.674074000	-3.369604000	4.832185000
1	2.355528000	-4.061629000	3.635005000
6	0.672233000	1.515662000	-0.112864000
8	1.331571000	2.233348000	0.469251000
8	-0.574426000	0.450547000	2.042089000
8	-1.301312000	2.995145000	2.725635000
1	-2.210601000	2.646664000	2.786789000
1	-1.376183000	0.207536000	1.552938000
1	-0.713284000	1.402204000	2.266603000
1	-1.348733000	3.644739000	2.006793000

$^{5}CAT_{H3O+,K+}$:

6	2.607330000	5.254455000	-1.231047000
6	1.406352000	4.969905000	-1.914534000
6	0.831995000	6.011020000	-2.673530000
6	1.428439000	7.273376000	-2.750453000
6	2.622782000	7.532503000	-2.068089000
6	3.208784000	6.514670000	-1.306743000
6	0.753063000	3.629798000	-1.830387000
6	1.465349000	2.493718000	-2.273509000
7	1.024865000	1.169902000	-2.175189000
6	2.088604000	0.375937000	-2.612469000
6	3.179796000	1.207488000	-3.008442000
6	2.783529000	2.518432000	-2.825720000
26	-0.855167000	0.549934000	-1.764260000
7	-1.343081000	2.424441000	-1.191829000
6	-2.480663000	2.795916000	-0.478027000
6	-2.353249000	4.149970000	-0.035041000
6	-1.154240000	4.626837000	-0.528145000
6	-0.530433000	3.553206000	-1.238615000
6	-3.617682000	1.987042000	-0.281453000
6	-4.732544000	2.478896000	0.581598000
6	-4.997227000	1.848634000	1.827536000
6	-6.058341000	2.265386000	2.641448000
6	-6.875332000	3.327898000	2.234728000
6	-6.628732000	3.974251000	1.022136000
6	-5.567952000	3.545823000	0.213559000
6	2.103951000	-1.036208000	-2.610233000
6	3.402581000	-1.728175000	-2.894602000
6	3.878963000	-1.838815000	-4.211341000
6	5.098721000	-2.451733000	-4.517094000
6	5.869010000	-2.985258000	-3.482850000
6	5.420517000	-2.906521000	-2.160440000
6	4.201406000	-2.280929000	-1.863043000
8	3.687049000	-2.228013000	-0.586727000
6	4.503525000	-2.288057000	0.562324000
6	4.742967000	-0.856958000	1.084381000
7	5.047554000	-0.672873000	2.397798000
8	-4.136979000	0.852713000	2.197448000
6	-4.556732000	-0.193813000	3.056484000
6	-3.803579000	-1.465012000	2.641866000
8	-4.445876000	-2.446383000	2.274641000
7	-0.348876000	-1.337807000	-2.258190000
6	0.942422000	-1.824746000	-2.455316000
6	0.908146000	-3.244149000	-2.606498000
6	-0.419518000	-3.625663000	-2.552108000
6	-1.194764000	-2.442042000	-2.345231000
6	-2.604686000	-2.418030000	-2.213878000
6	-3.358568000	-3.679065000	-2.476670000
6	-4.127784000	-4.308631000	-1.473941000
6	-4.822792000	-5.495574000	-1.726384000
6	-4.768673000	-6.092924000	-2.991087000
6	-4.010221000	-5.486816000	-3.999299000
6	-3.318124000	-4.299206000	-3.743706000
7	-2.732028000	-0.066306000	-1.392212000
6	-3.312066000	-1.276245000	-1.768864000
6	-4.723055000	-1.221403000	-1.541395000
6	-4.991678000	-0.004397000	-0.942769000
6	-3.755305000	0.700111000	-0.849793000
8	4.603085000	0.083990000	0.305639000
1	-4.176213000	-3.858535000	-0.479460000
1	1.776839000	-3.879698000	-2.768222000
1	-0.820590000	-4.632864000	-2.638983000
1	4.133858000	0.851281000	-3.390350000
1	3.359967000	3.415026000	-3.040400000
1	-0.732157000	5.619969000	-0.391625000
1	-3.087008000	4.690688000	0.558561000
1	-5.957786000	0.377258000	-0.617351000
1	-5.433300000	-2.008739000	-1.781803000
1	3.267086000	-1.419461000	-5.014245000
1	5.439807000	-2.514225000	-5.553372000
1	6.822500000	-3.475539000	-3.695682000
1	6.030459000	-3.347540000	-1.371228000
1	-0.097451000	5.818702000	-3.215584000
1	0.960960000	8.057666000	-3.352698000
1	3.091442000	8.518380000	-2.127842000
1	4.136949000	6.705268000	-0.760314000
1	3.068604000	4.471937000	-0.623447000
1	-5.385511000	4.038208000	-0.745004000
1	-7.264037000	4.802471000	0.698145000
1	-7.700733000	3.647979000	2.876036000
1	-6.239531000	1.777821000	3.601272000
1	-5.404877000	-5.961550000	-0.926052000
1	-5.311461000	-7.021105000	-3.188694000
1	-3.962544000	-5.937828000	-4.994656000
1	-2.735023000	-3.832358000	-4.541525000
1	3.959650000	-2.893038000	1.298525000
1	5.470689000	-2.781641000	0.385549000
1	-4.387945000	0.063773000	4.118183000

1	-5.624540000	-0.417885000	2.921341000
7	-2.436830000	-1.430635000	2.648052000
19	1.827126000	-0.109369000	0.441757000
8	0.242972000	0.989440000	2.600343000
8	0.406373000	-2.934040000	3.145550000
8	3.008525000	1.613682000	2.547544000
8	3.035380000	-2.129558000	4.132132000
6	-0.915682000	-3.427353000	2.988214000
1	-1.432455000	-3.490111000	3.965488000
6	-1.715732000	-2.545744000	2.032261000
1	-0.872295000	-4.454631000	2.575892000
1	-2.469794000	-3.166778000	1.537044000
1	-1.035570000	-2.161596000	1.252978000
6	1.157222000	-3.657962000	4.100323000
1	0.880805000	-3.347762000	5.127226000
1	0.950083000	-4.742294000	4.012827000
6	2.644950000	-3.474518000	3.886363000
1	3.175710000	-4.152758000	4.578690000
1	2.901625000	-3.775977000	2.854202000
6	4.387365000	-1.912523000	4.500885000
1	4.397568000	-0.999654000	5.114004000
1	4.750753000	-2.737089000	5.140851000
6	5.355110000	-1.766090000	3.322052000
1	6.362273000	-1.600237000	3.742854000
1	5.416532000	-2.709576000	2.767385000
6	-1.639718000	-0.363889000	3.251506000
6	-1.126861000	0.713151000	2.277783000
1	-2.208061000	0.101963000	4.066177000
1	-0.765424000	-0.834693000	3.720521000
1	-1.721234000	1.633835000	2.359691000
1	-1.212351000	0.372282000	1.231850000
6	0.701748000	2.311590000	2.339397000
1	-0.018639000	3.052474000	2.730739000
6	2.027494000	2.516233000	3.042305000
1	0.789632000	2.494478000	1.251256000
1	2.354702000	3.563470000	2.894212000
1	1.882797000	2.357173000	4.125474000
6	5.270877000	0.707270000	2.848293000
1	6.069835000	0.692083000	3.605966000
1	5.632813000	1.284237000	1.987163000
6	4.078128000	1.433977000	3.466054000
1	4.454785000	2.418565000	3.804700000
1	3.694423000	0.905326000	4.356080000
8	1.300320000	-0.118337000	5.081780000
1	0.950358000	0.270995000	4.260657000
1	1.848770000	-0.857659000	4.756100000

^{5}RC1 $_{H_3O^+,K^+}$:

6	0.725570000	0.355787000	-5.853101000
6	-0.440608000	-0.379998000	-5.568441000
6	-1.286285000	-0.715871000	-6.643610000
6	-0.974355000	-0.339603000	-7.953345000
6	0.192351000	0.386729000	-8.218975000
6	1.041121000	0.733207000	-7.162772000
6	-0.780956000	-0.786190000	-4.167802000
6	0.056411000	-1.700076000	-3.517420000
7	-0.096963000	-2.177288000	-2.232282000
6	0.938738000	-3.057462000	-1.988162000
6	1.789223000	-3.130065000	-3.167459000
6	1.237940000	-2.321592000	-4.112348000
26	-1.459053000	-1.539126000	-0.801993000
7	-2.416216000	-0.405187000	-2.307722000
6	-3.449582000	0.476681000	-2.072855000
6	-3.657873000	1.283322000	-3.229691000
6	-2.694026000	0.896458000	-4.169623000
6	-1.933640000	-0.157731000	-3.575049000
6	-4.160494000	0.618630000	-0.832848000
6	-5.036082000	1.827727000	-0.705764000
6	-4.481407000	3.076899000	-0.347564000
6	-5.274402000	4.222767000	-0.232298000
6	-6.653491000	4.136334000	-0.447897000
6	-7.230132000	2.913546000	-0.797205000
6	-6.421261000	1.780185000	-0.930892000
6	1.129903000	-3.807547000	-0.826882000
6	2.424007000	-4.547736000	-0.667332000
6	2.523770000	-5.923647000	-0.905685000
6	3.717020000	-6.625152000	-0.693024000
6	4.831873000	-5.938085000	-0.213111000
6	4.765882000	-4.561847000	0.036984000
6	3.575972000	-3.859497000	-0.206722000
8	3.424807000	-2.514542000	-0.023082000
6	4.479529000	-1.707333000	0.431447000
6	4.386298000	-0.331341000	-0.255391000
7	5.344670000	0.598402000	0.065950000
8	-3.101226000	3.145945000	-0.164954000
6	-2.531517000	3.961708000	0.857880000
6	-1.735972000	3.081319000	1.841187000
8	-2.136402000	1.950189000	2.059938000
7	-0.982022000	-3.220393000	0.371568000
6	0.210926000	-3.902380000	0.266857000
6	0.397678000	-4.719930000	1.426535000
6	-0.702926000	-4.518120000	2.241486000
6	-1.542687000	-3.565213000	1.579789000
6	-2.723714000	-2.979975000	2.147353000
6	-3.199204000	-3.552165000	3.448151000
6	-3.142775000	-2.810127000	4.642933000
6	-3.588964000	-3.353159000	5.852665000
6	-4.098469000	-4.654954000	5.894628000
6	-4.156348000	-5.408788000	4.716765000
6	-3.709513000	-4.863243000	3.509686000
7	-3.130962000	-1.247868000	0.417821000
6	-3.421319000	-1.895695000	1.603930000
6	-4.587059000	-1.263069000	2.213872000
6	-4.949589000	-0.224491000	1.415215000
6	-4.052075000	-0.234299000	0.266580000
8	3.537665000	-0.121424000	-1.113562000
1	-2.737576000	-1.795386000	4.621484000
1	1.247289000	-5.372043000	1.615614000
1	-0.893865000	-4.968493000	3.212862000
1	2.673456000	-3.757192000	-3.264229000
1	1.589291000	-2.160273000	-5.128742000
1	-2.560979000	1.294474000	-5.172710000
1	-4.420160000	2.050922000	-3.347404000
1	-5.777391000	0.466044000	1.561513000
1	-5.064563000	-1.580463000	3.137856000
1	1.636550000	-6.453740000	-1.261772000
1	3.767762000	-7.698381000	-0.890749000
1	5.768529000	-6.468318000	-0.022384000
1	5.648456000	-4.055267000	0.427457000
1	-2.196992000	-1.287247000	-6.446501000
1	-1.643487000	-0.619423000	-8.771579000
1	0.437212000	0.681314000	-9.242752000
1	1.952579000	1.305129000	-7.356983000
1	1.390064000	0.638280000	-5.032593000
1	-6.867409000	0.822527000	-1.210108000
1	-8.306133000	2.840806000	-0.971813000
1	-7.269720000	5.033517000	-0.352722000
1	-4.821337000	5.184718000	0.014229000
1	-3.532904000	-2.756759000	6.767367000
1	-4.447118000	-5.080548000	6.839186000
1	-4.555440000	-6.426557000	4.736759000
1	-3.761407000	-5.457721000	2.594004000
1	4.443142000	-1.630161000	1.530772000
1	5.466307000	-2.118712000	0.163488000

1	-1.955512000	4.761249000	0.381419000
1	-3.325448000	4.436112000	1.456166000
7	-0.676773000	3.645165000	2.523504000
19	2.094793000	1.840071000	0.812784000
8	1.808290000	4.779533000	0.983058000
8	1.772979000	1.939614000	3.654803000
8	4.125926000	3.419788000	-0.356387000
8	4.410387000	1.582528000	2.630505000
6	1.030289000	2.949720000	4.329923000
1	1.578237000	3.906085000	4.260907000
6	-0.405326000	3.054099000	3.850520000
1	0.960040000	2.697380000	5.403616000
1	-0.941485000	3.657384000	4.607043000
1	-0.853935000	2.054889000	3.849092000
6	2.850838000	1.459994000	4.449616000
1	3.369605000	2.305839000	4.934974000
1	2.471157000	0.791951000	5.246456000
6	3.847613000	0.699564000	3.602495000
1	4.634384000	0.300199000	4.264310000
1	3.357711000	-0.157070000	3.105204000
6	5.806180000	1.499996000	2.367412000
1	6.113283000	2.516295000	2.088916000
1	6.358580000	1.231603000	3.284810000
6	6.183899000	0.501228000	1.256423000
1	7.236582000	0.686347000	0.982514000
1	6.151569000	-0.519428000	1.653345000
6	-0.252890000	5.038528000	2.318960000
6	0.507907000	5.359477000	1.036671000
1	-1.115820000	5.724873000	2.362942000
1	0.381839000	5.317241000	3.170393000
1	0.600391000	6.460571000	0.987371000
1	-0.052297000	5.045912000	0.139377000
6	2.496758000	5.207193000	-0.196891000
1	2.442496000	6.308467000	-0.269975000
6	3.955654000	4.820161000	-0.184825000
1	1.999536000	4.785728000	-1.090637000
1	4.439838000	5.359141000	-1.020989000
1	4.433864000	5.148524000	0.755254000
6	5.665879000	1.617278000	-0.942288000
1	6.728601000	1.492714000	-1.215928000
1	5.059371000	1.389387000	-1.827065000
6	5.483056000	3.085608000	-0.591370000
1	5.861752000	3.661682000	-1.458102000
1	6.105331000	3.383367000	0.271505000
8	3.818067000	4.447516000	3.103248000
1	3.085710000	4.645083000	2.493220000
1	3.996386000	3.502143000	2.951566000
6	-0.175570000	-0.099550000	-0.080396000
8	0.368188000	-0.450163000	0.963455000
8	0.007288000	0.950006000	-0.756555000
8	-1.350251000	2.578121000	-2.001102000
1	-2.071509000	2.817362000	-1.324602000
1	-0.826280000	1.862041000	-1.585069000
1	-1.842334000	2.195820000	-2.787043000

[5]IM1 $_{H3O+,K+}$:

6	-0.158487000	0.460719000	5.965355000
6	-1.220942000	1.186534000	5.396666000
6	-2.169254000	1.766859000	6.258542000
6	-2.057790000	1.628616000	7.645704000
6	-0.995721000	0.904429000	8.197552000
6	-0.046491000	0.320210000	7.352214000
6	-1.345340000	1.335231000	3.908929000
6	-0.391645000	2.140879000	3.247466000
7	-0.347579000	2.388731000	1.899634000
6	0.725528000	3.205351000	1.664539000
6	1.414983000	3.470423000	2.919204000
6	0.710266000	2.835265000	3.898981000
26	-1.485581000	1.430271000	0.381201000
7	-2.674520000	0.631867000	1.930006000
6	-3.686682000	-0.265478000	1.707836000
6	-4.091551000	-0.850670000	2.967502000
6	-3.304151000	-0.287395000	3.939749000
6	-2.400961000	0.629590000	3.273263000
6	-4.283448000	-0.562448000	0.456030000
6	-5.216881000	-1.736793000	0.405005000
6	-4.700431000	-3.048065000	0.279350000
6	-5.561615000	-4.151080000	0.212104000
6	-6.946094000	-3.962652000	0.250188000
6	-7.475785000	-2.676221000	0.374962000
6	-6.610513000	-1.580682000	0.456056000
6	1.083532000	3.748221000	0.409407000
6	2.405358000	4.457336000	0.332277000
6	2.568262000	5.757678000	0.828463000
6	3.815811000	6.391432000	0.836217000
6	4.928788000	5.708084000	0.344511000
6	4.796745000	4.413231000	-0.168759000
6	3.540931000	3.790395000	-0.192925000
8	3.307073000	2.564189000	-0.748052000
6	4.333251000	1.653003000	-1.046670000
6	4.589603000	0.718407000	0.156584000
7	5.453302000	-0.343020000	-0.008024000
8	-3.331891000	-3.202062000	0.301287000
6	-2.673341000	-4.163457000	-0.520827000
6	-1.849330000	-3.405733000	-1.576974000
8	-2.408536000	-2.493830000	-2.177371000
7	-0.891541000	3.003079000	-0.907385000
6	0.293308000	3.678644000	-0.763304000
6	0.611594000	4.359831000	-2.001176000
6	-0.398921000	4.081390000	-2.879341000
6	-1.332026000	3.209928000	-2.188646000
6	-2.469634000	2.611519000	-2.792999000
6	-2.804861000	3.033707000	-4.192358000
6	-2.672288000	2.143022000	-5.273506000
6	-2.985248000	2.543182000	-6.576356000
6	-3.439489000	3.843009000	-6.823659000
6	-3.575999000	4.739680000	-5.758579000
6	-3.260089000	4.338707000	-4.456798000
7	-3.112102000	1.112233000	-0.935262000
6	-3.286892000	1.626731000	-2.191248000
6	-4.429564000	0.976642000	-2.820323000
6	-4.892697000	0.046171000	-1.937468000
6	-4.073774000	0.157371000	-0.740082000
8	4.014236000	0.935514000	1.214079000
1	-2.311639000	1.128085000	-5.089392000
1	1.488084000	4.981099000	-2.173007000
1	-0.493261000	4.424278000	-3.906851000
1	2.301338000	4.088545000	3.038129000
1	0.916545000	2.837368000	4.966578000
1	-3.336269000	-0.484099000	5.008755000
1	-4.883264000	-1.584850000	3.101192000
1	-5.732547000	-0.631680000	-2.069833000
1	-4.824049000	1.201500000	-3.808321000
1	1.692618000	6.275815000	1.227665000
1	3.915132000	7.405131000	1.230831000
1	5.914673000	6.179876000	0.351908000
1	5.681641000	3.904022000	-0.551081000
1	-2.999249000	2.337596000	5.834489000
1	-2.803114000	2.091589000	8.297754000
1	-0.908422000	0.795423000	9.281581000
1	0.784847000	-0.251315000	7.773257000
1	0.583379000	-0.003095000	5.310478000
1	-7.018153000	-0.571566000	0.554687000
1	-8.556809000	-2.523901000	0.413199000

1	-7.607686000	-4.830710000	0.196115000
1	-5.153048000	-5.161160000	0.145445000
1	-2.870013000	1.836246000	-7.402197000
1	-3.685254000	4.155886000	-7.841672000
1	-3.933270000	5.756739000	-5.940270000
1	-3.373419000	5.043334000	-3.629142000
1	3.984304000	1.080190000	-1.918859000
1	5.269927000	2.146168000	-1.349414000
1	-2.102815000	-4.835774000	0.129661000
1	-3.401494000	-4.774185000	-1.076325000
7	-0.567730000	-3.781503000	-1.867494000
19	2.572455000	-1.727137000	-0.685811000
8	1.828335000	-4.199306000	0.424971000
8	2.419023000	-3.145577000	-3.041353000
8	4.062163000	-2.565191000	1.482622000
8	5.102139000	-2.585979000	-2.041297000
6	1.239766000	-3.595594000	-3.695390000
1	1.226759000	-4.698958000	-3.772632000
6	-0.005745000	-3.049566000	-3.026243000
1	1.239829000	-3.208420000	-4.731045000
1	-0.797740000	-3.015745000	-3.788476000
1	0.172980000	-1.999854000	-2.738228000
6	3.614368000	-3.494011000	-3.726266000
1	3.921672000	-4.519299000	-3.454841000
1	3.447735000	-3.460952000	-4.818461000
6	4.722093000	-2.509588000	-3.417336000
1	5.588299000	-2.760212000	-4.055200000
1	4.394456000	-1.485243000	-3.671817000
6	6.351401000	-1.978704000	-1.717730000
1	6.828904000	-2.612479000	-0.958292000
1	7.015395000	-1.986153000	-2.598550000
6	6.247761000	-0.531993000	-1.227539000
1	7.280796000	-0.174740000	-1.058305000
1	5.831439000	0.082605000	-2.033182000
6	0.117693000	-4.900003000	-1.206088000
6	0.508641000	-4.722866000	0.266368000
1	-0.507121000	-5.803706000	-1.279484000
1	1.029444000	-5.131679000	-1.769712000
1	0.471595000	-5.718001000	0.747980000
1	-0.198282000	-4.068144000	0.798512000
6	2.148483000	-4.013380000	1.807799000
1	1.853383000	-4.913034000	2.378142000
6	3.632671000	-3.813498000	2.010601000
1	1.574652000	-3.158481000	2.208305000
1	3.837786000	-3.853348000	3.097545000
1	4.185512000	-4.637972000	1.527052000
6	5.920354000	-1.004857000	1.221639000
1	7.023731000	-0.999529000	1.220448000
1	5.580930000	-0.383797000	2.059174000
6	5.474472000	-2.442335000	1.477744000
1	5.883837000	-2.723104000	2.467346000
1	5.900134000	-3.151940000	0.747379000
8	4.241900000	-5.175037000	-0.953468000
1	3.378221000	-4.927358000	-0.578029000
1	4.611078000	-4.332117000	-1.272071000
6	-0.115884000	-0.083818000	-0.232395000
8	0.592766000	0.056073000	-1.238502000
8	0.182855000	-1.243464000	0.466678000
8	-1.424397000	-2.468665000	2.275946000
1	-2.184569000	-2.666595000	1.692302000
1	-0.449402000	-1.429720000	1.199231000
1	-1.832439000	-2.026310000	3.037170000

^{5}RC2 $_{H_3O^+,K^+}$:

6	1.025423000	5.955486000	-0.566957000
6	-0.125899000	5.553211000	-1.270070000
6	-0.963014000	6.549231000	-1.807199000
6	-0.656167000	7.904188000	-1.651212000
6	0.491166000	8.289580000	-0.949862000
6	1.329984000	7.310382000	-0.406930000
6	-0.460835000	4.103905000	-1.438318000
6	0.450497000	3.295026000	-2.157909000
7	0.347978000	1.930839000	-2.313028000
6	1.473505000	1.495492000	-2.961744000
6	2.322446000	2.634841000	-3.257932000
6	1.682580000	3.745750000	-2.781971000
26	-1.178276000	0.651415000	-1.614223000
7	-2.179556000	2.374530000	-0.930561000
6	-3.277828000	2.290809000	-0.118154000
6	-3.462189000	3.564624000	0.558734000
6	-2.461214000	4.398487000	0.108571000
6	-1.655772000	3.635779000	-0.819089000
6	-4.116324000	1.157160000	0.040927000
6	-5.195832000	1.222144000	1.084182000
6	-4.967501000	0.784247000	2.405406000
6	-5.972300000	0.822756000	3.375084000
6	-7.247352000	1.277796000	3.031052000
6	-7.503886000	1.717064000	1.729883000
6	-6.483463000	1.692974000	0.776097000
6	1.755594000	0.155383000	-3.327769000
6	3.133568000	-0.157409000	-3.832540000
6	3.362801000	-0.345558000	-5.207954000
6	4.623454000	-0.681898000	-5.703879000
6	5.689425000	-0.855498000	-4.815872000
6	5.488655000	-0.677883000	-3.446425000
6	4.225303000	-0.320401000	-2.953756000
8	4.036625000	-0.163279000	-1.588065000
6	4.811948000	0.886840000	-1.013874000
6	4.703692000	1.046958000	0.511830000
7	5.197647000	0.072393000	1.357820000
8	-3.663453000	0.397772000	2.759510000
6	-3.418423000	-0.751726000	3.611091000
6	-2.778389000	-1.862902000	2.762409000
8	-3.401203000	-2.230223000	1.771110000
7	-0.476456000	-0.843965000	-2.900554000
6	0.827237000	-0.913125000	-3.322533000
6	1.089622000	-2.246801000	-3.837760000
6	-0.081517000	-2.943809000	-3.748624000
6	-1.061709000	-2.055812000	-3.147576000
6	-2.390309000	-2.422925000	-2.808066000
6	-2.864264000	-3.774149000	-3.248480000
6	-3.165038000	-4.778447000	-2.309344000
6	-3.600775000	-6.040465000	-2.723999000
6	-3.747004000	-6.322087000	-4.086556000
6	-3.451700000	-5.333364000	-5.031027000
6	-3.012390000	-4.072647000	-4.615685000
7	-2.976322000	-0.409560000	-1.496273000
6	-3.276396000	-1.629715000	-2.041025000
6	-4.618615000	-2.016244000	-1.631180000
6	-5.068065000	-1.052392000	-0.778363000
6	-4.030868000	-0.037534000	-0.706704000
8	4.303022000	2.123903000	0.937239000
1	-3.046494000	-4.566874000	-1.243928000
1	2.038184000	-2.601470000	-4.232590000
1	-0.257778000	-3.974683000	-4.045941000
1	3.267353000	2.596399000	-3.795804000
1	2.017094000	4.777703000	-2.853605000
1	-2.298275000	5.435206000	0.393223000
1	-4.292473000	3.829912000	1.211912000
1	-6.024822000	-1.016840000	-0.263121000
1	-5.142653000	-2.916489000	-1.942227000
1	2.523919000	-0.219812000	-5.896676000
1	4.770803000	-0.814294000	-6.778178000
1	6.679028000	-1.134629000	-5.185638000

1	6.317110000	-0.825562000	-2.750612000
1	-1.857564000	6.255508000	-2.361748000
1	-1.315676000	8.661576000	-2.082674000
1	0.730252000	9.348811000	-0.826008000
1	2.225130000	7.601706000	0.148586000
1	1.680147000	5.196574000	-0.131886000
1	-6.680777000	2.035432000	-0.242336000
1	-8.496506000	2.081821000	1.456762000
1	-8.032607000	1.302134000	3.789879000
1	-5.760392000	0.513293000	4.399922000
1	-3.823353000	-6.808138000	-1.978339000
1	-4.088845000	-7.308232000	-4.410920000
1	-3.566174000	-5.542366000	-6.097840000
1	-2.788427000	-3.303597000	-5.359000000
1	5.882928000	0.758648000	-1.251612000
1	4.480462000	1.852586000	-1.422054000
1	-2.842206000	-0.404224000	4.474219000
1	-4.378137000	-1.139697000	3.974703000
7	-1.591296000	-2.418896000	3.127564000
19	2.166579000	-0.781746000	1.955506000
8	1.073419000	-0.599007000	4.596784000
8	1.323461000	-3.546482000	2.127985000
8	3.653967000	0.498463000	3.912118000
8	4.195338000	-2.719122000	2.116216000
6	0.134068000	-4.237511000	2.501820000
1	0.184332000	-4.554746000	3.560710000
6	-1.128717000	-3.459683000	2.184535000
1	0.071114000	-5.166649000	1.906034000
1	-1.941787000	-4.195916000	2.086858000
1	-1.012858000	-2.986426000	1.198594000
6	2.448186000	-4.414603000	2.095981000
1	2.743487000	-4.699033000	3.122411000
1	2.185444000	-5.341626000	1.551948000
6	3.622948000	-3.796606000	1.367551000
1	4.379278000	-4.588322000	1.229449000
1	3.309325000	-3.449256000	0.366130000
6	5.549660000	-2.390226000	1.794639000
1	6.068129000	-2.203669000	2.745732000
1	6.044418000	-3.258937000	1.330097000
6	5.715094000	-1.202942000	0.843291000
1	6.798339000	-1.118686000	0.630529000
1	5.206030000	-1.424870000	-0.102226000
6	-0.853034000	-2.063623000	4.341990000
6	-0.343324000	-0.610742000	4.422482000
1	-1.457496000	-2.300983000	5.231539000
1	0.025589000	-2.714735000	4.400923000
1	-0.803472000	-0.088859000	5.279872000
1	-0.598728000	-0.067515000	3.497633000
6	1.566068000	0.652295000	5.073975000
1	1.127278000	0.875893000	6.063731000
6	3.069440000	0.593775000	5.202616000
1	1.274427000	1.465207000	4.382740000
1	3.419795000	1.508642000	5.716811000
1	3.355379000	-0.276154000	5.819736000
6	5.725796000	0.556916000	2.648333000
1	6.779247000	0.236425000	2.726435000
1	5.709759000	1.652297000	2.599822000
6	5.024520000	0.136800000	3.935013000
1	5.546565000	0.662548000	4.756371000
1	5.113599000	-0.943617000	4.140363000
8	3.112293000	-2.741088000	4.901593000
1	2.374316000	-2.111302000	4.831591000
1	3.531050000	-2.734002000	4.023475000
6	-0.206370000	0.257799000	0.238249000
8	-0.371563000	-0.622228000	1.079555000
8	0.808010000	1.161452000	0.600431000
8	-2.073789000	2.300523000	2.793061000
1	-2.747271000	1.466927000	2.814168000
1	0.932743000	1.757520000	-0.156548000
1	-2.352518000	2.907434000	2.042129000
1	-2.114739000	2.801686000	3.630207000

^{5}TS2 $_{H_3O^+,K^+}$:

6	1.644328000	5.923980000	-1.111918000
6	0.511906000	5.547037000	-1.857603000
6	-0.170872000	6.540341000	-2.584100000
6	0.268444000	7.867545000	-2.570998000
6	1.396571000	8.227844000	-1.826531000
6	2.081936000	7.251606000	-1.095553000
6	0.030325000	4.128521000	-1.877170000
6	0.885346000	3.145184000	-2.437557000
7	0.629081000	1.798697000	-2.474001000
6	1.737980000	1.172217000	-2.980687000
6	2.739713000	2.171754000	-3.309897000
6	2.206292000	3.389853000	-2.996123000
26	-1.017812000	0.771355000	-1.673772000
7	-1.883498000	2.659300000	-1.267083000
6	-2.992627000	2.779803000	-0.470026000
6	-3.061773000	4.136293000	0.044551000
6	-1.983555000	4.811825000	-0.473352000
6	-1.235395000	3.866744000	-1.280453000
6	-3.935297000	1.757876000	-0.203295000
6	-4.991435000	2.023981000	0.830000000
6	-4.795083000	1.635191000	2.174490000
6	-5.778469000	1.866980000	3.142162000
6	-6.987403000	2.465670000	2.778803000
6	-7.204845000	2.856997000	1.455556000
6	-6.208745000	2.641374000	0.499588000
6	1.882589000	-0.220416000	-3.189223000
6	3.227411000	-0.734817000	-3.617061000
6	3.500945000	-0.967676000	-4.976981000
6	4.723816000	-1.493127000	-5.398584000
6	5.701785000	-1.814042000	-4.451834000
6	5.453498000	-1.595054000	-3.096170000
6	4.230354000	-1.051594000	-2.679217000
8	3.999524000	-0.852340000	-1.324617000
6	4.782065000	0.194922000	-0.756989000
6	4.728746000	0.332329000	0.774000000
7	5.157197000	-0.686133000	1.598318000
8	-3.557792000	1.103090000	2.536032000
6	-3.467550000	-0.005474000	3.455309000
6	-2.995360000	-1.247093000	2.677352000
8	-3.616971000	-1.537034000	1.661024000
7	-0.449562000	-0.936871000	-2.730040000
6	0.847469000	-1.182805000	-3.102392000
6	0.975707000	-2.577660000	-3.482034000
6	-0.266556000	-3.136512000	-3.366181000
6	-1.158490000	-2.096611000	-2.887327000
6	-2.534884000	-2.273826000	-2.586229000
6	-3.147330000	-3.600767000	-2.913805000
6	-3.618536000	-4.450343000	-1.895302000
6	-4.183573000	-5.691766000	-2.201806000
6	-4.291674000	-6.106341000	-3.533592000
6	-3.828685000	-5.271439000	-4.556078000
6	-3.259843000	-4.031801000	-4.248658000
7	-2.926903000	-0.080949000	-1.512929000
6	-3.351552000	-1.305511000	-1.957729000
6	-4.751504000	-1.481982000	-1.600901000
6	-5.115194000	-0.391747000	-0.868164000
6	-3.962440000	0.489702000	-0.827166000
8	4.422320000	1.432570000	1.221828000
1	-3.531717000	-4.133889000	-0.853160000
1	1.889048000	-3.064740000	-3.814371000
1	-0.546634000	-4.166272000	-3.573940000

1	3.711677000	1.970695000	-3.755308000
1	2.666869000	4.364829000	-3.133951000
1	-1.723047000	5.854771000	-0.309798000
1	-3.857698000	4.543286000	0.666252000
1	-6.083744000	-0.185365000	-0.418959000
1	-5.369729000	-2.337105000	-1.862376000
1	2.728931000	-0.728641000	-5.712458000
1	4.909169000	-1.658724000	-6.462374000
1	6.657831000	-2.239973000	-4.765572000
1	6.209291000	-1.851120000	-2.350462000
1	-1.049825000	6.265042000	-3.172176000
1	-0.272326000	8.622413000	-3.147623000
1	1.739403000	9.265526000	-1.814651000
1	2.960125000	7.524533000	-0.504760000
1	2.178850000	5.169226000	-0.530005000
1	-6.372702000	2.945414000	-0.536973000
1	-8.145037000	3.332202000	1.167093000
1	-7.751263000	2.638935000	3.540374000
1	-5.597473000	1.595792000	4.183719000
1	-4.537782000	-6.339075000	-1.395367000
1	-4.734725000	-7.076207000	-3.773578000
1	-3.912824000	-5.584383000	-5.600020000
1	-2.905432000	-3.382656000	-5.053044000
1	5.846340000	0.075899000	-1.031077000
1	4.435197000	1.165463000	-1.141136000
1	-2.831748000	0.306678000	4.290177000
1	-4.462462000	-0.239612000	3.857882000
7	-1.940772000	-1.993681000	3.115479000
19	1.996733000	-1.046652000	2.053461000
8	0.901164000	-0.703207000	4.754040000
8	0.773310000	-3.615313000	2.277805000
8	3.635018000	-0.081855000	4.114280000
8	3.734358000	-3.266392000	2.309099000
6	-0.530222000	-4.099808000	2.590771000
1	-0.582694000	-4.418980000	3.648681000
6	-1.630206000	-3.126161000	2.215430000
1	-0.719218000	-5.005670000	1.986446000
1	-2.551780000	-3.717963000	2.097394000
1	-1.402623000	-2.713141000	1.221261000
6	1.739390000	-4.659814000	2.262704000
1	1.965229000	-4.986809000	3.293474000
1	1.334999000	-5.529314000	1.711371000
6	3.009333000	-4.241143000	1.552297000
1	3.631294000	-5.142254000	1.414909000
1	2.766983000	-3.847893000	0.547641000
6	5.133252000	-3.167933000	2.026407000
1	5.649120000	-3.074780000	2.992537000
1	5.489714000	-4.103948000	1.566033000
6	5.515839000	-2.016549000	1.092829000
1	6.609417000	-2.083892000	0.936671000
1	5.029048000	-2.159805000	0.121952000
6	-1.251849000	-1.783320000	4.391672000
6	-0.486720000	-0.454148000	4.539188000
1	-1.960129000	-1.903508000	5.226459000
1	-0.517382000	-2.587354000	4.509485000
1	-0.878005000	0.120903000	5.396706000
1	-0.602301000	0.156950000	3.629750000
6	1.591937000	0.449406000	5.233357000
1	1.177328000	0.756249000	6.211104000
6	3.059052000	0.136769000	5.392607000
1	1.458919000	1.292101000	4.529145000
1	3.552022000	0.989888000	5.895964000
1	3.184640000	-0.756628000	6.029501000
6	5.723524000	-0.271559000	2.895627000
1	6.724856000	-0.726145000	2.986641000
1	5.848631000	0.817022000	2.850238000
6	4.952110000	-0.601980000	4.168303000
1	5.513901000	-0.138199000	5.000413000
1	4.910231000	-1.684585000	4.376070000
8	2.586065000	-3.130238000	5.059882000
1	1.952153000	-2.397849000	4.971621000
1	3.032600000	-3.175893000	4.196644000
6	-0.282593000	0.582154000	0.296175000
8	-0.349270000	-0.291403000	1.126228000
8	0.261192000	1.851586000	0.946235000
8	-1.536152000	2.782207000	2.370703000
1	-2.297306000	2.114701000	2.446603000
1	0.450879000	2.498480000	0.242882000
1	-0.709135000	2.366101000	1.791683000
1	-1.888145000	3.538285000	1.846460000

^{5}IM2 $_{H3O+,K+}$:

6	0.149903000	6.778454000	-0.571967000
6	-0.146609000	6.118700000	-1.778290000
6	-0.260543000	6.885957000	-2.951721000
6	-0.088262000	8.273144000	-2.919402000
6	0.205567000	8.917387000	-1.713032000
6	0.325561000	8.165239000	-0.539656000
6	-0.330410000	4.630607000	-1.824986000
6	0.678364000	3.881288000	-2.483759000
7	0.686529000	2.520635000	-2.690396000
6	1.924732000	2.179555000	-3.188788000
6	2.721079000	3.379620000	-3.327745000
6	1.945310000	4.429469000	-2.917988000
26	-0.597128000	1.150120000	-1.750415000
7	-1.786425000	2.760244000	-1.095928000
6	-2.928345000	2.645918000	-0.347008000
6	-3.346546000	3.965834000	0.090190000
6	-2.455302000	4.859631000	-0.434837000
6	-1.462119000	4.090096000	-1.167591000
6	-3.639357000	1.449098000	-0.105225000
6	-4.845907000	1.498144000	0.790783000
6	-4.737218000	1.239221000	2.176280000
6	-5.876369000	1.227406000	2.990002000
6	-7.136166000	1.468316000	2.435140000
6	-7.263431000	1.739412000	1.070930000
6	-6.121598000	1.754972000	0.264959000
6	2.366279000	0.864446000	-3.490328000
6	3.793297000	0.672481000	-3.909803000
6	4.240022000	1.197880000	-5.138698000
6	5.542752000	1.010280000	-5.600181000
6	6.446801000	0.278443000	-4.822982000
6	6.038611000	-0.246638000	-3.598635000
6	4.726983000	-0.060914000	-3.133308000
8	4.447750000	-0.688884000	-1.943933000
6	3.633538000	-0.080145000	-0.947840000
6	4.254834000	-0.202043000	0.452504000
7	4.497713000	-1.445164000	0.993075000
8	-3.465175000	1.104466000	2.694871000
6	-3.161525000	0.222343000	3.764597000
6	-2.771229000	-1.162682000	3.220534000
8	-3.163424000	-1.476464000	2.104247000
7	0.309530000	-0.365305000	-2.872660000
6	1.564102000	-0.296751000	-3.416585000
6	1.939231000	-1.601288000	-3.929534000
6	0.885498000	-2.436703000	-3.695382000
6	-0.138462000	-1.651646000	-3.025523000
6	-1.382504000	-2.152960000	-2.575293000
6	-1.704289000	-3.584576000	-2.882252000
6	-1.819063000	-4.532585000	-1.848659000
6	-2.113706000	-5.869557000	-2.132058000
6	-2.303522000	-6.282697000	-3.455072000
6	-2.196257000	-5.349695000	-4.491792000
6	-1.897935000	-4.013437000	-4.207886000

7	-2.204831000	-0.119437000	-1.395630000
6	-2.342226000	-1.413136000	-1.843287000
6	-3.638933000	-1.917451000	-1.439913000
6	-4.245814000	-0.932995000	-0.714887000
6	-3.346947000	0.199257000	-0.700856000
8	4.486639000	0.834352000	1.066254000
1	-1.671389000	-4.215822000	-0.813395000
1	2.875823000	-1.840566000	-4.427537000
1	0.809099000	-3.489958000	-3.952491000
1	3.745486000	3.423704000	-3.687790000
1	2.226040000	5.478965000	-2.882876000
1	-2.477481000	5.942472000	-0.343278000
1	-4.227889000	4.183323000	0.689740000
1	-5.224118000	-0.964884000	-0.243733000
1	-4.036872000	-2.900235000	-1.678158000
1	3.524688000	1.750601000	-5.752200000
1	5.846708000	1.425877000	-6.563494000
1	7.472217000	0.119905000	-5.165810000
1	6.726844000	-0.814056000	-2.968792000
1	-0.492728000	6.388847000	-3.896859000
1	-0.186068000	8.852439000	-3.841143000
1	0.342075000	10.001440000	-1.687561000
1	0.561144000	8.658984000	0.406630000
1	0.253757000	6.196277000	0.346937000
1	-6.213215000	1.956499000	-0.805169000
1	-8.244914000	1.936380000	0.634007000
1	-8.017336000	1.455875000	3.081139000
1	-5.786577000	1.047284000	4.062347000
1	-2.193195000	-6.591055000	-1.314806000
1	-2.535250000	-7.327404000	-3.677177000
1	-2.348783000	-5.661614000	-5.528221000
1	-1.821685000	-3.289275000	-5.022614000
1	3.549088000	0.998098000	-1.120368000
1	2.622175000	-0.519086000	-0.966113000
1	-2.363058000	0.700423000	4.340880000
1	-4.015806000	0.086278000	4.447634000
7	-2.065993000	-2.024827000	4.024789000
19	1.209754000	-1.413804000	2.264747000
8	0.845560000	-1.296669000	5.099514000
8	-0.172072000	-3.872597000	2.411444000
8	3.536264000	-1.135083000	3.814835000
8	2.609906000	-3.819435000	1.566798000
6	-0.940662000	-4.278571000	3.542090000
1	-0.301728000	-4.261298000	4.441503000
6	-2.204867000	-3.457117000	3.709965000
1	-1.275948000	-5.322422000	3.410352000
1	-2.789313000	-3.932472000	4.518565000
1	-2.793002000	-3.520746000	2.788136000
6	0.496587000	-4.958399000	1.780323000
1	0.968911000	-5.609267000	2.537197000
1	-0.226347000	-5.570984000	1.209185000
6	1.562482000	-4.460120000	0.830866000
1	1.964121000	-5.331554000	0.286857000
1	1.132987000	-3.769219000	0.082310000
6	3.921384000	-3.909751000	1.007447000
1	4.613032000	-4.115440000	1.836420000
1	3.977589000	-4.777202000	0.329449000
6	4.369454000	-2.683497000	0.214824000
1	5.344959000	-2.941657000	-0.238105000
1	3.675348000	-2.517092000	-0.616564000
6	-1.595385000	-1.675376000	5.373439000
6	-0.401949000	-0.730735000	5.502867000
1	-2.413848000	-1.226739000	5.961986000
1	-1.344716000	-2.615085000	5.884826000
1	-0.339936000	-0.457414000	6.572221000
1	-0.561351000	0.205888000	4.941293000
6	1.927298000	-0.438293000	5.482023000
1	1.862736000	-0.231687000	6.565704000
6	3.274812000	-1.063468000	5.210477000
1	1.842915000	0.530366000	4.953828000
1	4.040873000	-0.436439000	5.704933000
1	3.320805000	-2.069441000	5.662350000
6	5.344609000	-1.470048000	2.194150000
1	6.195574000	-2.149247000	2.012739000
1	5.742505000	-0.455419000	2.312107000
6	4.704241000	-1.889916000	3.516759000
1	5.471748000	-1.720113000	4.294185000
1	4.455323000	-2.962960000	3.547762000
8	2.212576000	-4.072346000	4.569266000
1	1.681988000	-3.290433000	4.793350000
1	2.357488000	-4.009155000	3.608862000
6	0.757554000	1.539050000	-0.104242000
8	1.451979000	2.140393000	0.562632000
8	-0.663867000	0.409040000	1.844065000
8	-1.351378000	2.935663000	2.628986000
1	-2.270595000	2.616026000	2.700698000
1	-1.495773000	0.130837000	1.428672000
1	-0.822407000	1.349057000	2.105304000
1	-1.394096000	3.622658000	1.945769000

UPBE0 data:

$^{3}CAT_{H3O+,K+}$:

6	3.471829000	3.882265000	-2.302857000
6	2.183122000	3.774305000	-2.857327000
6	1.792630000	4.753224000	-3.788069000
6	2.649106000	5.792643000	-4.150731000
6	3.924601000	5.880769000	-3.591316000
6	4.330945000	4.919226000	-2.663818000
6	1.260922000	2.679399000	-2.463442000
6	1.684958000	1.338330000	-2.625018000
7	1.015948000	0.227588000	-2.121343000
6	1.859692000	-0.850520000	-2.337365000
6	3.023347000	-0.430026000	-3.025031000
6	2.905123000	0.934269000	-3.227145000
26	-0.919427000	0.133126000	-1.638503000
7	-0.988368000	2.129884000	-1.564727000
6	-1.987317000	2.888376000	-0.975478000
6	-1.558546000	4.245791000	-0.846396000
6	-0.315641000	4.331700000	-1.426984000
6	0.033929000	3.016373000	-1.867762000
6	-3.261286000	2.415883000	-0.636779000
6	-4.208134000	3.268638000	0.126910000
6	-4.526653000	2.914315000	1.462583000
6	-5.458085000	3.644347000	2.203129000
6	-6.079454000	4.757745000	1.630884000
6	-5.766158000	5.141880000	0.329836000
6	-4.840282000	4.396763000	-0.406582000
6	1.605633000	-2.180059000	-1.914891000
6	2.713054000	-3.168400000	-1.986623000
6	2.677771000	-4.215296000	-2.918029000
6	3.689394000	-5.171849000	-3.010165000
6	4.776823000	-5.095266000	-2.145984000
6	4.851396000	-4.065964000	-1.205666000
6	3.838642000	-3.104069000	-1.131540000
8	3.866482000	-2.085511000	-0.225255000
6	5.037360000	-1.866126000	0.520722000
6	5.028522000	-0.398888000	0.943220000
7	5.342097000	-0.054226000	2.217031000
8	-3.839306000	1.864860000	1.974941000
6	-4.359103000	1.068107000	3.010531000
6	-3.820407000	-0.343657000	2.779351000
8	-4.609140000	-1.267031000	2.621081000
7	-0.834347000	-1.857182000	-1.626114000
6	0.316115000	-2.629906000	-1.583747000
6	-0.020566000	-3.987706000	-1.285439000
6	-1.392351000	-4.060524000	-1.235480000
6	-1.891667000	-2.740157000	-1.456800000
6	-3.253993000	-2.400832000	-1.458597000
6	-4.253672000	-3.499659000	-1.421056000
6	-5.134543000	-3.656322000	-0.335175000
6	-6.057356000	-4.701654000	-0.300898000
6	-6.125007000	-5.619296000	-1.351143000
6	-5.256822000	-5.481542000	-2.434987000
6	-4.335225000	-4.434913000	-2.467212000
7	-2.867359000	0.039392000	-1.251069000
6	-3.700198000	-1.061012000	-1.385640000
6	-5.058401000	-0.658818000	-1.268378000
6	-5.048617000	0.690405000	-0.959144000
6	-3.691860000	1.100824000	-0.945737000
8	4.779889000	0.435550000	0.083044000
1	-5.081772000	-2.950032000	0.497992000
1	0.692315000	-4.800481000	-1.159508000
1	-2.004957000	-4.940577000	-1.048783000
1	3.840975000	-1.081882000	-3.329973000
1	3.613544000	1.595024000	-3.722942000
1	0.313651000	5.214502000	-1.523430000
1	-2.140902000	5.048131000	-0.396249000
1	-5.903070000	1.340240000	-0.773314000
1	-5.922951000	-1.312463000	-1.365701000
1	1.820162000	-4.266194000	-3.593493000
1	3.625717000	-5.969301000	-3.754311000
1	5.577888000	-5.837258000	-2.191250000
1	5.700460000	-4.034964000	-0.522197000
1	0.797859000	4.688008000	-4.236559000
1	2.319487000	6.536091000	-4.881814000
1	4.597712000	6.693887000	-3.875179000
1	5.324143000	4.981944000	-2.210431000
1	3.795285000	3.140318000	-1.568061000
1	-4.611493000	4.679791000	-1.437483000
1	-6.249602000	6.012486000	-0.120046000
1	-6.806451000	5.326976000	2.215871000
1	-5.685837000	3.360582000	3.232990000
1	-6.725378000	-4.805434000	0.558770000
1	-6.848869000	-6.437963000	-1.323743000
1	-5.300895000	-6.191137000	-3.265877000
1	-3.661747000	-4.330334000	-3.321989000
1	5.110385000	-2.592026000	1.341044000
1	5.937129000	-1.977766000	-0.109873000
1	-4.095240000	1.468290000	4.006615000
1	-5.454786000	0.989891000	2.949771000
7	-2.471000000	-0.489820000	2.675061000
19	1.899549000	-0.220486000	0.741963000
8	0.425400000	1.650329000	2.323218000
8	0.337909000	-2.191440000	2.717083000
8	3.219470000	2.106989000	2.170075000
8	3.070818000	-1.476724000	3.213612000
6	-1.017461000	-2.542373000	2.920182000
1	-1.284411000	-2.449060000	3.990968000
6	-1.956896000	-1.708810000	2.069149000
1	-1.149792000	-3.606410000	2.646297000
1	-2.835611000	-2.318520000	1.831478000
1	-1.468431000	-1.471429000	1.106901000
6	1.201478000	-2.985716000	3.493193000
1	1.104424000	-2.727542000	4.565926000
1	0.940259000	-4.056779000	3.387064000
6	2.630596000	-2.814723000	3.043125000
1	3.267101000	-3.509152000	3.615193000
1	2.716003000	-3.082433000	1.975855000
6	4.169213000	-1.228556000	4.072173000
1	3.910033000	-0.335997000	4.659368000
1	4.305869000	-2.059944000	4.783768000
6	5.472108000	-0.999825000	3.310696000
1	6.220436000	-0.613414000	4.019520000
1	5.877015000	-1.949387000	2.941044000
6	-1.512459000	0.511336000	3.099009000
6	-0.930904000	1.388832000	1.991819000
1	-1.956711000	1.135554000	3.885285000
1	-0.673997000	-0.016382000	3.577476000
1	-1.482894000	2.334144000	1.899224000
1	-0.998930000	0.890502000	1.007706000
6	0.968523000	2.844320000	1.800532000
1	0.288774000	3.692339000	1.998132000
6	2.283458000	3.112391000	2.481336000
1	1.089206000	2.778478000	0.702250000
1	2.658631000	4.103990000	2.162912000
1	2.116726000	3.156539000	3.574274000
6	5.512025000	1.366781000	2.491473000
1	6.336310000	1.475788000	3.213264000
1	5.815430000	1.842432000	1.549711000
6	4.318151000	2.119840000	3.050208000
1	4.661245000	3.158784000	3.217660000
1	4.005840000	1.723723000	4.034097000
8	1.506137000	0.455130000	4.636033000

1	1.122231000	1.006341000	3.932973000
1	1.867676000	-0.288872000	4.128270000

^{3}RC1$_{H3O+, K+}$

6	1.679553000	4.062450000	-3.779712000
6	0.461352000	3.472331000	-4.151844000
6	-0.237613000	4.022513000	-5.238078000
6	0.268700000	5.118205000	-5.935475000
6	1.484024000	5.691247000	-5.556970000
6	2.187244000	5.159402000	-4.476029000
6	-0.083911000	2.303413000	-3.409450000
6	0.629489000	1.116053000	-3.398954000
7	0.288877000	-0.039887000	-2.727358000
6	1.322832000	-0.917964000	-2.938974000
6	2.319693000	-0.319296000	-3.799915000
6	1.881776000	0.925949000	-4.104114000
26	-1.357753000	-0.371961000	-1.641032000
7	-1.962308000	1.513337000	-1.974360000
6	-3.003343000	2.142211000	-1.342301000
6	-3.000552000	3.533522000	-1.642276000
6	-1.924563000	3.743843000	-2.506116000
6	-1.292713000	2.492325000	-2.681726000
6	-3.937409000	1.519160000	-0.473393000
6	-4.853043000	2.380406000	0.325565000
6	-4.491426000	2.793879000	1.622315000
6	-5.341625000	3.584462000	2.396356000
6	-6.583294000	3.964889000	1.891127000
6	-6.964300000	3.574052000	0.608736000
6	-6.099292000	2.797037000	-0.160414000
6	1.398070000	-2.227538000	-2.485250000
6	2.658455000	-2.996509000	-2.670072000
6	2.699845000	-4.084825000	-3.558020000
6	3.845689000	-4.854608000	-3.732726000
6	4.992424000	-4.556290000	-2.996760000
6	4.983122000	-3.482917000	-2.111642000
6	3.834731000	-2.699429000	-1.954838000
8	3.846977000	-1.645953000	-1.066378000
6	4.812946000	-0.657886000	-1.368436000
6	4.810987000	0.523076000	-0.399768000
7	5.360217000	0.365826000	0.846411000
8	-3.234010000	2.455942000	2.085933000
6	-3.058999000	2.001350000	3.428763000
6	-2.600877000	0.542853000	3.374947000
8	-3.373956000	-0.271454000	2.890553000
7	-0.954391000	-2.343439000	-1.724178000
6	0.283560000	-2.902950000	-1.930605000
6	0.274351000	-4.281371000	-1.576917000
6	-1.010564000	-4.575011000	-1.176481000
6	-1.749256000	-3.363147000	-1.258359000
6	-3.087350000	-3.236500000	-0.805418000
6	-3.818056000	-4.487835000	-0.460687000
6	-4.172797000	-4.785934000	0.863789000
6	-4.851849000	-5.963887000	1.176202000
6	-5.186329000	-6.869184000	0.169024000
6	-4.833446000	-6.589415000	-1.152180000
6	-4.153297000	-5.412486000	-1.461935000
7	-3.132550000	-0.771796000	-0.835703000
6	-3.695004000	-2.011935000	-0.592818000
6	-5.006612000	-1.852922000	-0.000151000
6	-5.194064000	-0.526551000	0.195048000
6	-4.037468000	0.142666000	-0.359311000
8	4.421013000	1.609932000	-0.800339000
1	-3.903975000	-4.084464000	1.657906000
1	1.124595000	-4.957123000	-1.628803000
1	-1.400839000	-5.529968000	-0.831306000
1	3.210165000	-0.825219000	-4.168682000
1	2.349221000	1.657076000	-4.759625000
1	-1.606261000	4.683694000	-2.950895000
1	-3.749988000	4.253531000	-1.317036000
1	-6.053748000	-0.023961000	0.633177000
1	-5.683705000	-2.667117000	0.248349000
1	1.797538000	-4.317930000	-4.128426000
1	3.842838000	-5.687072000	-4.440141000
1	5.897085000	-5.158790000	-3.108029000
1	5.876257000	-3.253348000	-1.526116000
1	-1.187757000	3.575591000	-5.541929000
1	-0.287912000	5.524771000	-6.783903000
1	1.881006000	6.550765000	-6.102931000
1	3.137126000	5.603962000	-4.167735000
1	2.231865000	3.653256000	-2.929511000
1	-6.392988000	2.483806000	-1.165463000
1	-7.934323000	3.874316000	0.206131000
1	-7.246544000	4.581488000	2.502241000
1	-5.029477000	3.914975000	3.389279000
1	-5.116389000	-6.177362000	2.215139000
1	-5.718510000	-7.792098000	0.412946000
1	-5.092272000	-7.291717000	-1.948879000
1	-3.880122000	-5.197490000	-2.498300000
1	5.830543000	-1.085228000	-1.392217000
1	4.599522000	-0.215089000	-2.351572000
1	-2.388977000	2.694173000	3.951075000
1	-4.024946000	2.003423000	3.951566000
7	-1.372978000	0.189250000	3.833377000
19	2.247643000	0.372537000	1.673417000
8	1.471743000	2.328793000	3.683615000
8	1.346321000	-1.526683000	3.756557000
8	3.982075000	2.530436000	2.397847000
8	4.222617000	-1.092404000	3.230274000
6	0.120747000	-1.791870000	4.408985000
1	0.148718000	-1.439731000	5.458602000
6	-1.060667000	-1.234677000	3.655619000
1	-0.026520000	-2.886847000	4.452981000
1	-1.950799000	-1.799649000	3.968177000
1	-0.916281000	-1.437797000	2.581467000
6	2.407932000	-2.269000000	4.310365000
1	2.723307000	-1.834100000	5.276731000
1	2.078388000	-3.307501000	4.501975000
6	3.582495000	-2.351547000	3.370779000
1	4.294082000	-3.081166000	3.794083000
1	3.253306000	-2.733857000	2.387233000
6	5.579256000	-1.144538000	2.823522000
1	6.123629000	-0.386183000	3.404305000
1	6.016431000	-2.119744000	3.094443000
6	5.796105000	-0.936281000	1.332543000
1	6.876244000	-1.078680000	1.139312000
1	5.251125000	-1.714627000	0.783337000
6	-0.442193000	1.108513000	4.469880000
6	0.069036000	2.230057000	3.563574000
1	-0.873712000	1.517710000	5.396649000
1	0.436167000	0.529020000	4.777464000
1	-0.381173000	3.202983000	3.826947000
1	-0.192690000	1.999042000	2.516083000
6	1.994919000	3.550989000	3.199918000
1	1.615068000	4.394555000	3.804346000
6	3.492537000	3.503688000	3.290163000
1	1.680855000	3.716332000	2.152415000
1	3.904047000	4.500629000	3.044944000
1	3.788193000	3.260476000	4.326861000
6	5.965366000	1.557627000	1.439725000
1	7.008745000	1.312060000	1.701971000
1	5.987706000	2.321625000	0.651752000
6	5.316084000	2.173278000	2.661894000
1	5.914471000	3.070344000	2.908376000
1	5.350576000	1.514932000	3.547490000

8	3.335920000	0.776135000	5.291861000
1	2.668982000	1.296174000	4.816358000
1	3.678433000	0.159205000	4.625022000
6	-0.273345000	0.085484000	-0.086905000
8	0.097848000	-0.907089000	0.541431000
8	0.043950000	1.284284000	0.187474000
8	-1.249677000	3.301254000	0.580096000
1	-1.983087000	2.998046000	1.200207000
1	-0.743053000	2.498566000	0.336327000
1	-1.736842000	3.595778000	-0.251907000

^{3}IM1 $_{H3O+, K+}$

6	-0.229638000	1.826040000	-5.597973000
6	-1.216778000	0.858773000	-5.360073000
6	-2.103016000	0.537023000	-6.397997000
6	-2.001650000	1.160727000	-7.641376000
6	-1.012915000	2.118945000	-7.866927000
6	-0.127468000	2.450347000	-6.840866000
6	-1.337454000	0.196777000	-4.029147000
6	-0.352132000	-0.705721000	-3.630056000
7	-0.330226000	-1.432780000	-2.460246000
6	0.796386000	-2.218023000	-2.532220000
6	1.529980000	-1.935601000	-3.746299000
6	0.804224000	-1.024477000	-4.439124000
26	-1.566617000	-1.306070000	-0.923776000
7	-2.654426000	0.081076000	-1.953786000
6	-3.666544000	0.836461000	-1.436639000
6	-4.128058000	1.786560000	-2.404466000
6	-3.360088000	1.603765000	-3.525918000
6	-2.426521000	0.558863000	-3.213309000
6	-4.207830000	0.700162000	-0.144709000
6	-5.165652000	1.741855000	0.324571000
6	-4.694778000	2.985803000	0.789380000
6	-5.588292000	3.957492000	1.247880000
6	-6.957399000	3.699628000	1.261020000
6	-7.443135000	2.477954000	0.798517000
6	-6.547777000	1.517112000	0.331527000
6	1.158402000	-3.227407000	-1.639588000
6	2.476308000	-3.899850000	-1.838808000
6	2.651415000	-4.880852000	-2.818347000
6	3.898466000	-5.457755000	-3.061190000
6	4.996044000	-5.038533000	-2.314258000
6	4.849317000	-4.069740000	-1.321258000
6	3.592984000	-3.510996000	-1.067476000
8	3.345406000	-2.625981000	-0.069666000
6	4.365975000	-1.907244000	0.551645000
6	4.704732000	-0.651021000	-0.262263000
7	5.534901000	0.282617000	0.295864000
8	-3.353564000	3.243110000	0.704083000
6	-2.659763000	3.857035000	1.773909000
6	-1.925012000	2.765181000	2.552771000
8	-2.560181000	1.760034000	2.839378000
7	-0.866149000	-3.098562000	-0.243653000
6	0.323455000	-3.665530000	-0.598708000
6	0.607918000	-4.799107000	0.233518000
6	-0.434795000	-4.907545000	1.110566000
6	-1.329508000	-3.821281000	0.816001000
6	-2.443179000	-3.479810000	1.607204000
6	-2.811234000	-4.401420000	2.720317000
6	-2.619507000	-4.035480000	4.060725000
6	-2.956632000	-4.908696000	5.094490000
6	-3.490348000	-6.165127000	4.805857000
6	-3.682774000	-6.542667000	3.476588000
6	-3.344331000	-5.668899000	2.443651000
7	-2.984251000	-1.359308000	0.449087000
6	-3.172497000	-2.302179000	1.433611000
6	-4.255250000	-1.901617000	2.307845000
6	-4.691051000	-0.696469000	1.868805000
6	-3.925677000	-0.383627000	0.683047000
8	4.215488000	-0.510717000	-1.370566000
1	-2.193878000	-3.055613000	4.291971000
1	1.490202000	-5.431655000	0.154511000
1	-0.570673000	-5.641183000	1.902306000
1	2.463392000	-2.411320000	-4.037370000
1	1.019842000	-0.600921000	-5.417284000
1	-3.415735000	2.142179000	-4.469779000
1	-4.936628000	2.499697000	-2.254451000
1	-5.485360000	-0.079073000	2.280855000
1	-4.624075000	-2.478021000	3.152987000
1	1.784134000	-5.184322000	-3.410538000
1	4.010761000	-6.220651000	-3.834567000
1	5.984383000	-5.467698000	-2.496898000
1	5.724977000	-3.761540000	-0.748693000
1	-2.877043000	-0.215539000	-6.226478000
1	-2.698488000	0.893585000	-8.439943000
1	-0.933292000	2.607870000	-8.841052000
1	0.646971000	3.203576000	-7.006754000
1	0.461252000	2.093354000	-4.794068000
1	-6.919772000	0.556637000	-0.034036000
1	-8.516048000	2.273461000	0.796793000
1	-7.645543000	4.467917000	1.621691000
1	-5.213430000	4.927168000	1.582610000
1	-2.796508000	-4.606381000	6.132596000
1	-3.754627000	-6.849566000	5.615812000
1	-4.101987000	-7.524110000	3.240525000
1	-3.499423000	-5.967793000	1.403718000
1	3.981315000	-1.638756000	1.546392000
1	5.274188000	-2.508233000	0.723045000
1	-2.022795000	4.639740000	1.347309000
1	-3.353952000	4.343009000	2.477253000
7	-0.628637000	2.931784000	2.923914000
19	2.500627000	1.277379000	1.191197000
8	1.829638000	3.959591000	0.903883000
8	2.300542000	1.900614000	3.875443000
8	4.116175000	2.787928000	-0.482972000
8	5.026153000	1.718039000	2.895542000
6	1.096232000	2.073910000	4.591308000
1	1.054038000	3.074720000	5.061710000
6	-0.103515000	1.814414000	3.715519000
1	1.063620000	1.337596000	5.415223000
1	-0.926445000	1.484838000	4.364943000
1	0.108736000	0.956247000	3.052566000
6	3.446691000	2.006668000	4.689829000
1	3.746274000	3.065080000	4.791794000
1	3.231833000	1.613484000	5.699963000
6	4.584015000	1.190670000	4.134231000
1	5.410024000	1.217787000	4.866986000
1	4.266649000	0.137981000	4.020156000
6	6.295622000	1.261190000	2.471929000
1	6.799031000	2.105647000	1.980751000
1	6.914363000	0.992459000	3.344798000
6	6.247020000	0.055763000	1.545467000
1	7.291907000	-0.237111000	1.333261000
1	5.790729000	-0.785123000	2.080777000
6	0.130479000	4.146010000	2.649810000
6	0.522211000	4.419593000	1.200033000
1	-0.431746000	5.015607000	3.023068000
1	1.051787000	4.121723000	3.246388000
1	0.494303000	5.513039000	1.035720000
1	-0.185905000	3.957515000	0.494506000
6	2.165613000	4.194423000	-0.454675000
1	1.846344000	5.211056000	-0.747254000
6	3.652406000	4.107779000	-0.663210000
1	1.627698000	3.478047000	-1.102346000

1	3.879768000	4.455242000	-1.688808000
1	4.158165000	4.787243000	0.046055000
6	6.041818000	1.320080000	-0.597586000
1	7.140316000	1.354084000	-0.506329000
1	5.798992000	0.999785000	-1.618750000
6	5.520624000	2.732868000	-0.402541000
1	5.973528000	3.344431000	-1.205884000
1	5.847871000	3.176019000	0.554385000
8	4.142837000	4.456902000	2.590256000
1	3.316370000	4.329538000	2.096497000
1	4.506213000	3.559941000	2.673609000
6	-0.220001000	-0.100670000	0.237819000
8	0.488706000	-0.565537000	1.134419000
8	0.042544000	1.226333000	0.000703000
8	-1.430919000	3.038822000	-1.359565000
1	-2.208986000	3.075562000	-0.772133000
1	-0.567640000	1.637972000	-0.650537000
1	-1.817775000	2.839307000	-2.224200000

^{3}**RC2** $_{H3O+, K+}$

6	0.922370000	6.099071000	0.140890000
6	-0.277469000	5.714213000	-0.476407000
6	-1.222357000	6.706208000	-0.781199000
6	-0.968666000	8.045483000	-0.490136000
6	0.230999000	8.416017000	0.119006000
6	1.174375000	7.438468000	0.435797000
6	-0.560220000	4.287196000	-0.787481000
6	0.263959000	3.598753000	-1.661448000
7	0.174089000	2.240706000	-1.948208000
6	1.310006000	1.928893000	-2.666463000
6	2.103920000	3.115963000	-2.874837000
6	1.441261000	4.147966000	-2.291919000
26	-1.319247000	0.998618000	-1.513843000
7	-2.143407000	2.410634000	-0.375178000
6	-3.132709000	2.181165000	0.543584000
6	-3.226240000	3.283362000	1.449848000
6	-2.300804000	4.222985000	1.013036000
6	-1.637693000	3.662996000	-0.103352000
6	-3.994346000	1.062308000	0.553910000
6	-4.999590000	0.918508000	1.645672000
6	-4.719967000	0.154431000	2.792500000
6	-5.662222000	-0.016853000	3.804907000
6	-6.922785000	0.561682000	3.675205000
6	-7.226653000	1.329207000	2.552156000
6	-6.268148000	1.507187000	1.556843000
6	1.644125000	0.670393000	-3.147904000
6	2.997539000	0.436358000	-3.724795000
6	3.191683000	0.439175000	-5.114331000
6	4.441040000	0.193606000	-5.677058000
6	5.529320000	-0.078314000	-4.847509000
6	5.360947000	-0.093790000	-3.466226000
6	4.106568000	0.166772000	-2.905831000
8	3.951561000	0.124527000	-1.538015000
6	4.602078000	1.178796000	-0.858158000
6	4.636402000	1.074730000	0.666083000
7	5.220857000	-0.002578000	1.284686000
8	-3.433274000	-0.350063000	2.935581000
6	-3.205801000	-1.669337000	3.453103000
6	-2.589859000	-2.516015000	2.337862000
8	-3.254299000	-2.661823000	1.321637000
7	-0.606129000	-0.302356000	-2.826527000
6	0.703310000	-0.377514000	-3.230904000
6	0.942986000	-1.614622000	-3.900399000
6	-0.261179000	-2.272954000	-3.950757000
6	-1.209954000	-1.452501000	-3.276629000
6	-2.557879000	-1.810316000	-3.059630000
6	-3.077038000	-3.026257000	-3.743484000
6	-3.440089000	-4.166214000	-3.010408000
6	-3.913848000	-5.307464000	-3.656434000
6	-4.032941000	-5.327970000	-5.046389000
6	-3.671320000	-4.201763000	-5.786690000
6	-3.193507000	-3.062212000	-5.140984000
7	-2.976124000	-0.054385000	-1.385333000
6	-3.369017000	-1.126345000	-2.166773000
6	-4.699225000	-1.544594000	-1.793124000
6	-5.055839000	-0.811805000	-0.710102000
6	-3.980469000	0.118781000	-0.463885000
8	4.240632000	2.042370000	1.298547000
1	-3.339148000	-4.155191000	-1.921998000
1	1.897432000	-1.933760000	-4.312444000
1	-0.473894000	-3.242110000	-4.396566000
1	3.034995000	3.149290000	-3.437489000
1	1.727773000	5.196984000	-2.273036000
1	-2.103393000	5.204229000	1.438679000
1	-3.990422000	3.411179000	2.216692000
1	-5.975783000	-0.866228000	-0.132511000
1	-5.269552000	-2.328385000	-2.286350000
1	2.333022000	0.644075000	-5.758661000
1	4.564328000	0.209682000	-6.762274000
1	6.513122000	-0.285371000	-5.275215000
1	6.206971000	-0.316830000	-2.811758000
1	-2.161919000	6.420683000	-1.261045000
1	-1.712478000	8.805106000	-0.743424000
1	0.429005000	9.465804000	0.349172000
1	2.112545000	7.718482000	0.921438000
1	1.658805000	5.335034000	0.402743000
1	-6.501672000	2.104261000	0.672027000
1	-8.210884000	1.791482000	2.450702000
1	-7.661825000	0.422552000	4.467331000
1	-5.410018000	-0.587730000	4.700663000
1	-4.187381000	-6.187827000	-3.069389000
1	-4.405399000	-6.221918000	-5.552753000
1	-3.763536000	-4.208811000	-6.875695000
1	-2.912968000	-2.181473000	-5.724211000
1	5.651722000	1.273565000	-1.194099000
1	4.091455000	2.131070000	-1.066305000
1	-2.624664000	-1.581953000	4.377655000
1	-4.170563000	-2.131313000	3.700062000
7	-1.367294000	-3.078930000	2.492108000
19	2.207216000	-1.049052000	1.674569000
8	1.230765000	-1.507290000	4.303491000
8	1.515355000	-3.789757000	1.207385000
8	3.703389000	-0.161309000	3.849529000
8	4.337348000	-2.893811000	1.435566000
6	0.364147000	-4.594300000	1.376155000
1	0.436654000	-5.190560000	2.305961000
6	-0.919140000	-3.806049000	1.300926000
1	0.321932000	-5.323429000	0.546282000
1	-1.717037000	-4.513160000	1.026657000
1	-0.846283000	-3.084925000	0.471399000
6	2.663383000	-4.577432000	0.978830000
1	2.966005000	-5.095978000	1.907148000
1	2.438316000	-5.351424000	0.221203000
6	3.813264000	-3.764151000	0.443935000
1	4.596919000	-4.473191000	0.125807000
1	3.494846000	-3.195260000	-0.449080000
6	5.671915000	-2.463588000	1.221806000
1	6.168349000	-2.451568000	2.202496000
1	6.210397000	-3.204088000	0.607437000
6	5.798100000	-1.112180000	0.534673000
1	6.877517000	-0.936653000	0.366622000
1	5.310689000	-1.161011000	-0.445910000
6	-0.568582000	-2.996163000	3.705159000
6	-0.172977000	-1.582137000	4.143917000

1	-1.069329000	-3.528368000	4.529278000
1	0.364577000	-3.542996000	3.525325000
1	-0.648867000	-1.320176000	5.105488000
1	-0.492313000	-0.852850000	3.379967000
6	1.646266000	-0.364699000	5.027968000
1	1.214220000	-0.382360000	6.044962000
6	3.145213000	-0.358535000	5.128274000
1	1.291845000	0.555776000	4.526995000
1	3.457167000	0.448848000	5.816675000
1	3.487596000	-1.318903000	5.554571000
6	5.743502000	0.243224000	2.629337000
1	6.811556000	-0.033504000	2.636475000
1	5.674606000	1.325240000	2.797477000
6	5.079192000	-0.453619000	3.800717000
1	5.585108000	-0.079258000	4.710078000
1	5.220107000	-1.548603000	3.787366000
8	3.288209000	-3.567235000	4.094136000
1	2.558692000	-2.935704000	4.194339000
1	3.690092000	-3.348765000	3.238217000
6	-0.237726000	0.259372000	0.227433000
8	-0.433558000	-0.688796000	0.970413000
8	0.844173000	1.032722000	0.566372000
8	-1.803456000	1.518318000	3.177359000
1	-2.451220000	0.694127000	3.100608000
1	0.916821000	1.705283000	-0.132427000
1	-2.160103000	2.239919000	2.557269000
1	-1.809636000	1.860975000	4.088336000

^{3}T**S2** $_{H3O+, K+}$

6	1.792850000	5.684287000	-1.119747000
6	0.619494000	5.363474000	-1.819293000
6	-0.055721000	6.386461000	-2.502086000
6	0.433967000	7.691794000	-2.494069000
6	1.603421000	7.997659000	-1.797554000
6	2.280011000	6.990349000	-1.108705000
6	0.081076000	3.975439000	-1.827014000
6	0.859620000	2.946180000	-2.377439000
7	0.547190000	1.608788000	-2.363251000
6	1.656393000	0.953950000	-2.823838000
6	2.681075000	1.900928000	-3.192476000
6	2.177255000	3.138480000	-2.941114000
26	-1.160760000	0.757001000	-1.784458000
7	-1.849001000	2.569677000	-1.192796000
6	-2.910019000	2.738208000	-0.349480000
6	-2.898378000	4.067833000	0.205619000
6	-1.819987000	4.707089000	-0.349881000
6	-1.163260000	3.752100000	-1.199763000
6	-3.889656000	1.771157000	-0.076705000
6	-4.883932000	2.020540000	1.004995000
6	-4.681806000	1.457615000	2.280577000
6	-5.605309000	1.656912000	3.307070000
6	-6.755820000	2.405944000	3.067386000
6	-6.973559000	2.975719000	1.814558000
6	-6.037750000	2.785788000	0.799406000
6	1.789059000	-0.433707000	-2.961803000
6	3.121369000	-1.006525000	-3.305269000
6	3.368624000	-1.478717000	-4.603672000
6	4.589120000	-2.047041000	-4.955280000
6	5.593099000	-2.167616000	-3.994917000
6	5.370741000	-1.711025000	-2.699580000
6	4.148050000	-1.127144000	-2.353379000
8	3.938880000	-0.700077000	-1.059019000
6	4.765897000	0.379220000	-0.664049000
6	4.783320000	0.644776000	0.839659000
7	5.244293000	-0.310375000	1.706230000
8	-3.499977000	0.777778000	2.507121000
6	-3.502391000	-0.401224000	3.320647000

6	-2.966712000	-1.564270000	2.480761000
8	-3.607219000	-1.868374000	1.483877000
7	-0.599407000	-0.946496000	-2.667455000
6	0.699178000	-1.313684000	-2.899292000
6	0.773708000	-2.722403000	-3.185837000
6	-0.507533000	-3.188195000	-3.174458000
6	-1.352092000	-2.071877000	-2.838318000
6	-2.742374000	-2.169888000	-2.639206000
6	-3.410823000	-3.449668000	-3.000852000
6	-3.992276000	-4.262770000	-2.015561000
6	-4.600951000	-5.469548000	-2.356959000
6	-4.640020000	-5.883730000	-3.688889000
6	-4.062529000	-5.085713000	-4.676910000
6	-3.449942000	-3.880709000	-4.335303000
7	-2.973825000	0.011384000	-1.526651000
6	-3.486367000	-1.159847000	-2.021586000
6	-4.883019000	-1.281467000	-1.663982000
6	-5.168386000	-0.238186000	-0.845587000
6	-3.973115000	0.566410000	-0.777584000
8	4.456850000	1.759942000	1.220319000
1	-3.953006000	-3.944751000	-0.970760000
1	1.684890000	-3.280734000	-3.387262000
1	-0.849170000	-4.205476000	-3.352117000
1	3.644258000	1.645119000	-3.629908000
1	2.651259000	4.100515000	-3.120563000
1	-1.495544000	5.731451000	-0.180155000
1	-3.654824000	4.487504000	0.868082000
1	-6.110694000	-0.001455000	-0.356114000
1	-5.546213000	-2.082975000	-1.980548000
1	2.575341000	-1.385780000	-5.349411000
1	4.754555000	-2.398429000	-5.976068000
1	6.552883000	-2.622783000	-4.249969000
1	6.154580000	-1.812556000	-1.945704000
1	-0.970007000	6.150084000	-3.052394000
1	-0.101146000	8.474212000	-3.037940000
1	1.986311000	9.021120000	-1.789480000
1	3.191926000	7.223008000	-0.553193000
1	2.319864000	4.900947000	-0.568862000
1	-6.202930000	3.223882000	-0.187919000
1	-7.870969000	3.568816000	1.626234000
1	-7.476305000	2.554781000	3.874868000
1	-5.420862000	1.242055000	4.299899000
1	-5.043170000	-6.092734000	-1.575600000
1	-5.118373000	-6.829185000	-3.956344000
1	-4.090455000	-5.401669000	-5.722762000
1	-3.001623000	-3.256909000	-5.112789000
1	5.808502000	0.220230000	-0.992585000
1	4.401383000	1.313230000	-1.116173000
1	-2.969208000	-0.194971000	4.256083000
1	-4.538015000	-0.666803000	3.572656000
7	-1.838429000	-2.225717000	2.840214000
19	2.079671000	-0.781864000	1.829745000
8	0.838128000	-0.577520000	4.440171000
8	0.964799000	-3.501325000	1.867664000
8	3.504574000	0.290571000	4.027539000
8	3.849014000	-2.927630000	2.212477000
6	-0.259560000	-4.134817000	2.187415000
1	-0.244502000	-4.503272000	3.231142000
6	-1.468833000	-3.282518000	1.893258000
1	-0.367264000	-5.030663000	1.548524000
1	-2.326962000	-3.967394000	1.802121000
1	-1.336074000	-2.817614000	0.905017000
6	2.014109000	-4.441122000	1.808632000
1	2.197521000	-4.879085000	2.807722000
1	1.735973000	-5.268689000	1.128595000
6	3.289074000	-3.839268000	1.278779000
1	3.996235000	-4.666901000	1.099942000

1	3.108592000	-3.342449000	0.307504000
6	5.255932000	-2.767417000	2.158902000
1	5.602831000	-2.626525000	3.192029000
1	5.726830000	-3.694361000	1.791161000
6	5.719278000	-1.618283000	1.274145000
1	6.824650000	-1.633986000	1.268482000
1	5.378441000	-1.799329000	0.249662000
6	-1.082084000	-1.982274000	4.058131000
6	-0.560786000	-0.555046000	4.244740000
1	-1.661147000	-2.295780000	4.941558000
1	-0.199962000	-2.632898000	4.033538000
1	-1.032645000	-0.071393000	5.117879000
1	-0.789157000	0.042773000	3.348462000
6	1.345321000	0.637190000	4.959386000
1	0.823551000	0.895982000	5.898283000
6	2.811537000	0.478022000	5.238866000
1	1.177844000	1.461609000	4.241360000
1	3.179571000	1.380180000	5.762083000
1	2.964238000	-0.387428000	5.909541000
6	5.685381000	0.162007000	3.017564000
1	6.693490000	-0.243831000	3.203909000
1	5.766583000	1.254229000	2.947933000
6	4.820237000	-0.169713000	4.217742000
1	5.284585000	0.333290000	5.086078000
1	4.798359000	-1.249429000	4.448071000
8	2.510714000	-2.922311000	4.808649000
1	1.927373000	-2.149744000	4.742655000
1	2.979670000	-2.949640000	3.959267000
6	-0.387112000	0.421884000	0.170240000
8	-0.428164000	-0.500237000	0.940738000
8	0.277271000	1.602449000	0.862479000
8	-1.459352000	2.474431000	2.366654000
1	-2.194083000	1.788680000	2.435274000
1	0.468071000	2.269764000	0.181741000
1	-0.648095000	2.096163000	1.745616000
1	-1.840661000	3.234730000	1.870924000

^{3}**IM2** $_{H3O+,\ K+}$

6	0.955349000	6.693594000	-1.145188000
6	-0.118780000	6.099397000	-1.823281000
6	-1.058993000	6.924929000	-2.457997000
6	-0.916566000	8.310779000	-2.430658000
6	0.156366000	8.892779000	-1.754027000
6	1.088673000	8.080985000	-1.108038000
6	-0.278979000	4.620193000	-1.861713000
6	0.705917000	3.875810000	-2.523966000
7	0.749688000	2.501375000	-2.618312000
6	1.940326000	2.172876000	-3.200289000
6	2.674410000	3.380052000	-3.512935000
6	1.895637000	4.428690000	-3.129468000
26	-0.596588000	1.154661000	-1.730871000
7	-1.800895000	2.781543000	-1.161398000
6	-2.875486000	2.682077000	-0.327213000
6	-3.188417000	3.960159000	0.223008000
6	-2.268784000	4.848851000	-0.304893000
6	-1.412675000	4.094060000	-1.158606000
6	-3.621657000	1.481380000	-0.101682000
6	-4.818589000	1.550241000	0.785571000
6	-4.713001000	1.256846000	2.159143000
6	-5.847181000	1.264767000	2.973144000
6	-7.095237000	1.559084000	2.427043000
6	-7.215969000	1.867403000	1.073706000
6	-6.079125000	1.866991000	0.267141000
6	2.404106000	0.855344000	-3.418738000
6	3.814753000	0.663975000	-3.850156000
6	4.213461000	1.115969000	-5.120828000
6	5.499458000	0.917136000	-5.609660000
6	6.434423000	0.248782000	-4.817915000
6	6.072368000	-0.203458000	-3.555579000
6	4.776431000	-0.007825000	-3.060826000
8	4.555616000	-0.551745000	-1.833325000
6	3.679207000	0.034206000	-0.895132000
6	4.228852000	-0.112918000	0.522606000
7	4.467581000	-1.365715000	1.023727000
8	-3.452880000	1.072627000	2.662629000
6	-3.180906000	0.148992000	3.689775000
6	-2.765689000	-1.194499000	3.089447000
8	-3.111708000	-1.446378000	1.946486000
7	0.338828000	-0.367210000	-2.798804000
6	1.622346000	-0.333776000	-3.261543000
6	2.057234000	-1.648418000	-3.597859000
6	1.000864000	-2.492413000	-3.319053000
6	-0.057132000	-1.676189000	-2.826235000
6	-1.344591000	-2.154984000	-2.412156000
6	-1.644424000	-3.585479000	-2.675619000
6	-1.927724000	-4.457327000	-1.612247000
6	-2.202957000	-5.802809000	-1.851039000
6	-2.210569000	-6.294986000	-3.156313000
6	-1.934111000	-5.436191000	-4.221388000
6	-1.644395000	-4.094310000	-3.983707000
7	-2.171183000	-0.107415000	-1.303946000
6	-2.324113000	-1.399362000	-1.761253000
6	-3.661370000	-1.858413000	-1.457390000
6	-4.283396000	-0.857570000	-0.781777000
6	-3.345881000	0.237751000	-0.701159000
8	4.419684000	0.900752000	1.177667000
1	-1.915207000	-4.072987000	-0.589071000
1	3.027547000	-1.915657000	-4.010253000
1	0.968003000	-3.572206000	-3.444799000
1	3.655096000	3.425013000	-3.979561000
1	2.118199000	5.487608000	-3.237205000
1	-2.198744000	5.917578000	-0.114981000
1	-4.008558000	4.176738000	0.905178000
1	-5.296128000	-0.845599000	-0.386646000
1	-4.073950000	-2.825376000	-1.735549000
1	3.468747000	1.614944000	-5.745754000
1	5.766651000	1.273825000	-6.606594000
1	7.451381000	0.082322000	-5.181036000
1	6.787444000	-0.720706000	-2.912510000
1	-1.901408000	6.472250000	-2.986656000
1	-1.649569000	8.940098000	-2.941312000
1	0.264196000	9.979860000	-1.728423000
1	1.926642000	8.529326000	-0.568729000
1	1.683582000	6.060632000	-0.632332000
1	-6.163042000	2.099855000	-0.797367000
1	-8.191233000	2.107637000	0.645050000
1	-7.975351000	1.560948000	3.074351000
1	-5.760854000	1.060239000	4.041903000
1	-2.411749000	-6.469903000	-1.011000000
1	-2.431285000	-7.348535000	-3.344306000
1	-1.944867000	-5.813789000	-5.246738000
1	-1.429982000	-3.425460000	-4.820541000
1	3.595543000	1.116346000	-1.058648000
1	2.672102000	-0.411751000	-0.969685000
1	-2.411261000	0.597378000	4.326937000
1	-4.059748000	-0.034611000	4.330074000
7	-2.098362000	-2.093906000	3.871827000
19	1.161935000	-1.400590000	2.209289000
8	0.753962000	-1.403791000	5.031820000
8	-0.182050000	-3.849927000	2.232734000
8	3.444079000	-1.168474000	3.812116000
8	2.613558000	-3.760087000	1.488472000
6	-0.971186000	-4.309045000	3.313492000
1	-0.356265000	-4.339178000	4.230008000

6	-2.232065000	-3.498108000	3.488658000
1	-1.303750000	-5.344404000	3.121894000
1	-2.839663000	-4.009752000	4.256403000
1	-2.797764000	-3.514170000	2.549803000
6	0.509109000	-4.895534000	1.582924000
1	0.966240000	-5.573897000	2.325298000
1	-0.188904000	-5.491056000	0.965376000
6	1.595006000	-4.350996000	0.694694000
1	2.009861000	-5.191786000	0.113643000
1	1.184623000	-3.620776000	-0.027999000
6	3.925086000	-3.816606000	0.955439000
1	4.607541000	-4.034627000	1.789094000
1	4.007435000	-4.665972000	0.257266000
6	4.364281000	-2.569676000	0.207889000
1	5.344862000	-2.800608000	-0.247398000
1	3.672795000	-2.385775000	-0.623454000
6	-1.670428000	-1.809185000	5.238027000
6	-0.493338000	-0.870379000	5.437688000
1	-2.506345000	-1.398624000	5.830018000
1	-1.423306000	-2.770869000	5.709181000
1	-0.454293000	-0.649518000	6.520183000
1	-0.649112000	0.093966000	4.922857000
6	1.808334000	-0.553271000	5.460725000
1	1.722455000	-0.382404000	6.549001000
6	3.160431000	-1.151605000	5.193747000
1	1.724413000	0.432307000	4.964227000
1	3.910741000	-0.538350000	5.727376000
1	3.205919000	-2.173613000	5.609735000
6	5.278798000	-1.424090000	2.234524000
1	6.146309000	-2.080808000	2.049232000
1	5.656535000	-0.407289000	2.398817000
6	4.616145000	-1.896483000	3.518144000
1	5.364954000	-1.748280000	4.317831000
1	4.379126000	-2.973263000	3.507434000
8	2.127664000	-4.105237000	4.413952000
1	1.595645000	-3.331914000	4.652283000
1	2.301173000	-4.005291000	3.463918000
6	0.667383000	1.564098000	-0.133072000
8	1.397470000	2.109217000	0.541633000
8	-0.698963000	0.427604000	1.822125000
8	-1.354443000	2.937586000	2.625864000
1	-2.257967000	2.604617000	2.759752000
1	-1.526973000	0.152988000	1.401929000
1	-0.859102000	1.361007000	2.097275000
1	-1.463459000	3.568560000	1.897946000

^{5}CAT$_{H_3O^+, K^+}$

6	3.455111000	3.907256000	-2.377416000
6	2.149298000	3.788578000	-2.890666000
6	1.723364000	4.770366000	-3.804851000
6	2.560384000	5.815854000	-4.192210000
6	3.853976000	5.911396000	-3.675767000
6	4.295611000	4.950074000	-2.764496000
6	1.247164000	2.688103000	-2.473526000
6	1.683442000	1.353716000	-2.611174000
7	1.021817000	0.240121000	-2.107411000
6	1.869505000	-0.837212000	-2.319287000
6	3.038033000	-0.408480000	-3.002778000
6	2.911357000	0.949813000	-3.209564000
26	-0.919812000	0.135807000	-1.640339000
7	-1.002470000	2.139133000	-1.566865000
6	-1.997468000	2.888180000	-0.960545000
6	-1.576502000	4.243264000	-0.831746000
6	-0.334438000	4.334285000	-1.424378000
6	0.014224000	3.025627000	-1.871497000
6	-3.267827000	2.401583000	-0.608148000
6	-4.213380000	3.251461000	0.160424000
6	-4.528913000	2.892583000	1.495725000
6	-5.458698000	3.620278000	2.240848000
6	-6.080527000	4.736024000	1.674138000
6	-5.769438000	5.125075000	0.373815000
6	-4.845458000	4.382585000	-0.367194000
6	1.620087000	-2.163187000	-1.909308000
6	2.728977000	-3.149194000	-1.985801000
6	2.694292000	-4.195335000	-2.918499000
6	3.707903000	-5.149089000	-3.014386000
6	4.796968000	-5.071979000	-2.151891000
6	4.870673000	-4.044494000	-1.209699000
6	3.855714000	-3.085061000	-1.131899000
8	3.883058000	-2.068419000	-0.223322000
6	5.053643000	-1.849040000	0.522897000
6	5.038763000	-0.383611000	0.951473000
7	5.344917000	-0.042514000	2.228077000
8	-3.840965000	1.841364000	2.003413000
6	-4.359167000	1.038315000	3.034552000
6	-3.815395000	-0.370750000	2.798712000
8	-4.600236000	-1.298032000	2.645026000
7	-0.828241000	-1.861859000	-1.647631000
6	0.325391000	-2.626990000	-1.589924000
6	-0.005379000	-3.981663000	-1.298066000
6	-1.382949000	-4.058054000	-1.257938000
6	-1.882551000	-2.743213000	-1.481371000
6	-3.252392000	-2.400960000	-1.478926000
6	-4.245770000	-3.503877000	-1.461761000
6	-5.146179000	-3.673378000	-0.392948000
6	-6.064730000	-4.723013000	-0.381203000
6	-6.108130000	-5.636465000	-1.436119000
6	-5.218519000	-5.489261000	-2.501918000
6	-4.302470000	-4.438102000	-2.512094000
7	-2.869429000	0.034373000	-1.242847000
6	-3.696604000	-1.067880000	-1.373851000
6	-5.058666000	-0.667450000	-1.231742000
6	-5.049782000	0.672011000	-0.902904000
6	-3.690575000	1.091359000	-0.910216000
8	4.789710000	0.453247000	0.093727000
1	-5.110053000	-2.974449000	0.447063000
1	0.708805000	-4.792660000	-1.169679000
1	-1.993836000	-4.939898000	-1.074125000
1	3.858775000	-1.056884000	-3.305942000
1	3.613780000	1.610576000	-3.713565000
1	0.292158000	5.219068000	-1.519945000
1	-2.157743000	5.042366000	-0.374961000
1	-5.903586000	1.316815000	-0.698869000
1	-5.923155000	-1.320456000	-1.332296000
1	1.835430000	-4.247361000	-3.592227000
1	3.645002000	-5.944829000	-3.760452000
1	5.599686000	-5.811968000	-2.200279000
1	5.720887000	-4.012589000	-0.527686000
1	0.715254000	4.700313000	-4.221659000
1	2.201793000	6.558530000	-4.910428000
1	4.512106000	6.729534000	-3.979500000
1	5.302154000	5.018328000	-2.342261000
1	3.806411000	3.169450000	-1.651411000
1	-4.618573000	4.669539000	-1.397380000
1	-6.253654000	5.997400000	-0.071853000
1	-6.806315000	5.303237000	2.262546000
1	-5.684738000	3.332265000	3.269909000
1	-6.747482000	-4.834176000	0.465932000
1	-6.828143000	-6.458874000	-1.426379000
1	-5.242626000	-6.195217000	-3.336790000
1	-3.613643000	-4.326957000	-3.353667000
1	5.129520000	-2.578335000	1.339995000
1	5.953438000	-1.954720000	-0.108693000
1	-4.097642000	1.434470000	4.032936000

1	-5.454546000	0.956727000	2.972450000
7	-2.465608000	-0.510326000	2.687324000
19	1.912362000	-0.213695000	0.754653000
8	0.420998000	1.642803000	2.331536000
8	0.347681000	-2.203018000	2.726906000
8	3.211514000	2.109119000	2.181158000
8	3.079612000	-1.480199000	3.215229000
6	-1.005976000	-2.559020000	2.931432000
1	-1.272503000	-2.467230000	4.002444000
6	-1.948516000	-1.727790000	2.081199000
1	-1.135088000	-3.623281000	2.656863000
1	-2.825724000	-2.339632000	1.843068000
1	-1.459297000	-1.489293000	1.119692000
6	1.216403000	-2.996833000	3.497572000
1	1.122356000	-2.742499000	4.571497000
1	0.958505000	-4.068454000	3.388958000
6	2.643185000	-2.819197000	3.042458000
1	3.284803000	-3.513326000	3.609101000
1	2.725021000	-3.082618000	1.973868000
6	4.175249000	-1.230001000	4.076945000
1	3.910982000	-0.341160000	4.667460000
1	4.314895000	-2.063666000	4.785224000
6	5.477924000	-0.992043000	3.317990000
1	6.223847000	-0.605382000	4.029213000
1	5.887212000	-1.938211000	2.944586000
6	-1.509835000	0.494044000	3.110057000
6	-0.934808000	1.376210000	2.003082000
1	-1.954306000	1.115254000	3.898590000
1	-0.667941000	-0.031083000	3.585284000
1	-1.490889000	2.319359000	1.913719000
1	-1.003475000	0.879162000	1.018409000
6	0.958733000	2.838697000	1.807373000
1	0.275165000	3.683865000	2.003717000
6	2.272346000	3.112922000	2.488408000
1	1.079414000	2.772263000	0.709037000
1	2.644935000	4.104628000	2.167426000
1	2.104288000	3.159803000	3.580992000
6	5.506527000	1.378286000	2.508776000
1	6.327388000	1.488651000	3.234253000
1	5.811170000	1.859094000	1.570051000
6	4.306867000	2.123507000	3.065709000
1	4.644656000	3.163290000	3.238229000
1	3.992051000	1.722231000	4.046630000
8	1.511172000	0.446899000	4.640275000
1	1.124508000	0.999102000	3.939540000
1	1.871112000	-0.296456000	4.130387000

^{5}**RC1** $_{H_3O^+, K^+}$

6	0.406820000	3.412322000	-4.602208000
6	-0.671718000	2.531192000	-4.778417000
6	-1.506465000	2.724168000	-5.891036000
6	-1.266409000	3.753975000	-6.799711000
6	-0.185316000	4.617397000	-6.613798000
6	0.650585000	4.442512000	-5.510897000
6	-0.932425000	1.430613000	-3.808627000
6	0.005745000	0.402610000	-3.700850000
7	-0.066042000	-0.682163000	-2.865077000
6	1.053135000	-1.449589000	-3.087111000
6	1.874947000	-0.810784000	-4.099554000
6	1.221683000	0.310545000	-4.495799000
26	-1.435262000	-1.008398000	-1.353308000
7	-2.517961000	0.649690000	-2.052446000
6	-3.630289000	1.167461000	-1.437128000
6	-3.985165000	2.397393000	-2.045983000
6	-3.018302000	2.652345000	-3.024456000
6	-2.123473000	1.541271000	-3.014349000
6	-4.297256000	0.595235000	-0.304796000
6	-5.288288000	1.473999000	0.375194000
6	-4.865251000	2.544948000	1.187004000
6	-5.783555000	3.383850000	1.817723000
6	-7.150831000	3.154731000	1.672343000
6	-7.596912000	2.100053000	0.879105000
6	-6.669559000	1.282284000	0.234719000
6	1.352442000	-2.667027000	-2.477064000
6	2.704492000	-3.243197000	-2.742485000
6	2.989351000	-3.919659000	-3.931440000
6	4.271574000	-4.389815000	-4.221043000
6	5.294751000	-4.175398000	-3.302477000
6	5.039149000	-3.514538000	-2.099932000
6	3.749132000	-3.061736000	-1.807915000
8	3.401879000	-2.477869000	-0.631046000
6	4.349947000	-1.924743000	0.227958000
6	4.635544000	-0.465976000	-0.156718000
7	5.429651000	0.282283000	0.667776000
8	-3.509757000	2.796514000	1.313219000
6	-2.936664000	3.026328000	2.595035000
6	-2.165227000	1.772544000	3.007587000
8	-2.727103000	0.697997000	2.865539000
7	-0.744417000	-2.974663000	-1.177903000
6	0.486902000	-3.393098000	-1.609139000
6	0.779141000	-4.676536000	-1.061255000
6	-0.298666000	-5.025418000	-0.272236000
6	-1.229573000	-3.942320000	-0.343218000
6	-2.429143000	-3.842874000	0.429097000
6	-2.795439000	-5.032891000	1.249457000
6	-2.764358000	-4.991813000	2.651950000
6	-3.106431000	-6.110884000	3.411691000
6	-3.482532000	-7.297922000	2.782926000
6	-3.512467000	-7.356643000	1.388632000
6	-3.170609000	-6.236668000	0.631894000
7	-3.064171000	-1.530927000	-0.175718000
6	-3.240911000	-2.709091000	0.508930000
6	-4.423314000	-2.595728000	1.349280000
6	-4.909382000	-1.339125000	1.192318000
6	-4.070732000	-0.680294000	0.205780000
8	4.135862000	-0.005699000	-1.170101000
1	-2.460147000	-4.067936000	3.150814000
1	1.684649000	-5.252575000	-1.245166000
1	-0.423050000	-5.933475000	0.314444000
1	2.820406000	-1.195991000	-4.475671000
1	1.527741000	1.018750000	-5.262969000
1	-2.975926000	3.501594000	-3.703411000
1	-4.832260000	3.025815000	-1.776457000
1	-5.779647000	-0.898701000	1.674459000
1	-4.825086000	-3.385048000	1.981305000
1	2.179235000	-4.064259000	-4.651123000
1	4.467959000	-4.911173000	-5.160556000
1	6.308675000	-4.525203000	-3.512382000
1	5.859201000	-3.363086000	-1.396912000
1	-2.351383000	2.048000000	-6.046105000
1	-1.925491000	3.879694000	-7.662809000
1	0.003740000	5.424459000	-7.326127000
1	1.496824000	5.116170000	-5.352154000
1	1.059761000	3.285967000	-3.734441000
1	-7.014122000	0.461077000	-0.398648000
1	-8.666580000	1.917882000	0.752979000
1	-7.863887000	3.814392000	2.172163000
1	-5.430486000	4.224795000	2.418618000
1	-3.072959000	-6.055464000	4.502973000
1	-3.750082000	-8.175373000	3.377065000
1	-3.808602000	-8.280897000	0.885476000
1	-3.199830000	-6.287879000	-0.459655000
1	3.914978000	-1.982321000	1.237033000
1	5.288915000	-2.500054000	0.263933000

1	-2.353884000	3.952464000	2.542796000
1	-3.725584000	3.174658000	3.347286000
7	-0.920503000	1.865937000	3.549677000
19	2.287071000	0.899137000	1.570156000
8	1.540334000	3.502100000	2.173975000
8	1.969512000	0.650098000	4.325203000
8	3.936819000	2.886051000	0.623869000
8	4.763630000	0.865549000	3.523827000
6	0.734022000	0.569570000	5.001503000
1	0.644600000	1.368350000	5.761847000
6	-0.426556000	0.572029000	4.042119000
1	0.688792000	-0.389032000	5.550167000
1	-1.272056000	0.084107000	4.549129000
1	-0.190786000	-0.081320000	3.184636000
6	3.070656000	0.516379000	5.198161000
1	3.318201000	1.493043000	5.650569000
1	2.821275000	-0.185555000	6.014185000
6	4.269080000	-0.049433000	4.484917000
1	5.047812000	-0.251892000	5.241654000
1	4.000655000	-1.011009000	4.010331000
6	6.072205000	0.594078000	3.063479000
1	6.558161000	1.561803000	2.877190000
1	6.658908000	0.095066000	3.853536000
6	6.122237000	-0.278432000	1.818163000
1	7.187512000	-0.443395000	1.572742000
1	5.701691000	-1.262532000	2.055778000
6	-0.246024000	3.134113000	3.804601000
6	0.256773000	3.917617000	2.599455000
1	-0.909673000	3.797816000	4.380362000
1	0.612648000	2.945161000	4.461435000
1	0.305822000	4.983710000	2.888185000
1	-0.444073000	3.843542000	1.752348000
6	1.949748000	4.190106000	0.998574000
1	1.624597000	5.243871000	1.050614000
6	3.447274000	4.187376000	0.861453000
1	1.469127000	3.734191000	0.113287000
1	3.716686000	4.855657000	0.021863000
1	3.897006000	4.598377000	1.782780000
6	5.908040000	1.555297000	0.136660000
1	7.003362000	1.592817000	0.259486000
1	5.690334000	1.548527000	-0.938948000
6	5.340161000	2.831514000	0.731524000
1	5.796966000	3.666094000	0.166577000
1	5.629288000	2.969911000	1.787782000
8	3.781961000	3.519282000	4.072490000
1	2.976482000	3.519544000	3.531502000
1	4.187795000	2.657634000	3.878968000
6	-0.266236000	-0.036494000	0.047964000
8	0.267062000	-0.835725000	0.806924000
8	-0.133058000	1.207666000	-0.027123000
8	-1.652423000	3.092824000	-0.461023000
1	-2.440113000	2.961204000	0.164648000
1	-1.075079000	2.305334000	-0.377759000
1	-2.050569000	3.099661000	-1.384129000

^{5}IM1 $_{H_3O^+, K^+}$

6	-0.219002000	0.860889000	-5.880953000
6	-1.284350000	0.046375000	-5.469689000
6	-2.255983000	-0.311837000	-6.415881000
6	-2.163309000	0.128189000	-7.735902000
6	-1.097799000	0.936825000	-8.132697000
6	-0.126262000	1.302481000	-7.200520000
6	-1.389258000	-0.425486000	-4.057973000
6	-0.425908000	-1.345739000	-3.599806000
7	-0.370879000	-1.887431000	-2.348988000
6	0.706762000	-2.718709000	-2.312486000
6	1.388807000	-2.689696000	-3.592285000
6	0.671244000	-1.860082000	-4.398081000
26	-1.492571000	-1.302320000	-0.652230000
7	-2.690220000	-0.188445000	-1.968376000
6	-3.698521000	0.624515000	-1.544741000
6	-4.127052000	1.468158000	-2.632533000
6	-3.353604000	1.139363000	-3.713714000
6	-2.439751000	0.109928000	-3.274646000
6	-4.283751000	0.627620000	-0.257688000
6	-5.210208000	1.752725000	0.063301000
6	-4.684219000	3.015513000	0.407159000
6	-5.535783000	4.072274000	0.738663000
6	-6.916504000	3.881528000	0.746087000
6	-7.454765000	2.642570000	0.404716000
6	-6.600558000	1.594796000	0.062890000
6	1.077490000	-3.525909000	-1.217792000
6	2.396836000	-4.220795000	-1.309511000
6	2.574405000	-5.347771000	-2.116222000
6	3.825367000	-5.945710000	-2.273542000
6	4.924758000	-5.399524000	-1.617322000
6	4.776294000	-4.282245000	-0.794941000
6	3.516881000	-3.700121000	-0.622894000
8	3.268129000	-2.665594000	0.218676000
6	4.284363000	-1.852195000	0.719066000
6	4.584541000	-0.710381000	-0.261568000
7	5.433092000	0.288725000	0.140429000
8	-3.329458000	3.182236000	0.332841000
6	-2.624135000	3.956704000	1.282362000
6	-1.815864000	3.004705000	2.166940000
8	-2.379501000	1.998314000	2.571358000
7	-0.884805000	-3.117094000	0.237178000
6	0.299054000	-3.726766000	-0.058438000
6	0.639569000	-4.659726000	0.989122000
6	-0.361471000	-4.594531000	1.913403000
6	-1.305790000	-3.606377000	1.437780000
6	-2.438673000	-3.172099000	2.167150000
6	-2.749349000	-3.889360000	3.437381000
6	-2.647943000	-3.242047000	4.678134000
6	-2.936643000	-3.918525000	5.862850000
6	-3.333732000	-5.255641000	5.828863000
6	-3.437876000	-5.911682000	4.601924000
6	-3.146900000	-5.234912000	3.417986000
7	-3.105515000	-1.310116000	0.701485000
6	-3.267188000	-2.089591000	1.805663000
6	-4.403252000	-1.611134000	2.572488000
6	-4.875457000	-0.508776000	1.928818000
6	-4.066250000	-0.344323000	0.737166000
8	4.054738000	-0.715661000	-1.359577000
1	-2.328758000	-2.197239000	4.711499000
1	1.526219000	-5.290967000	1.008694000
1	-0.443673000	-5.154717000	2.842411000
1	2.281732000	-3.255389000	-3.848971000
1	0.865848000	-1.618427000	-5.440852000
1	-3.400501000	1.564378000	-4.714326000
1	-4.925888000	2.206409000	-2.586782000
1	-5.715088000	0.119837000	2.217462000
1	-4.788518000	-2.059521000	3.485832000
1	1.705393000	-5.753602000	-2.640629000
1	3.938608000	-6.824928000	-2.911396000
1	5.915978000	-5.843942000	-1.736899000
1	5.654000000	-3.877210000	-0.289935000
1	-3.089736000	-0.949595000	-6.111043000
1	-2.927549000	-0.166136000	-8.459736000
1	-1.025065000	1.282199000	-9.166964000
1	0.709372000	1.939851000	-7.500681000
1	0.540654000	1.155750000	-5.152379000
1	-7.013327000	0.619576000	-0.207439000
1	-8.536388000	2.491138000	0.399827000

1	-7.571731000	4.716058000	1.007074000
1	-5.121640000	5.054038000	0.977630000
1	-2.846089000	-3.397613000	6.819466000
1	-3.560713000	-5.785761000	6.757168000
1	-3.751424000	-6.958072000	4.564461000
1	-3.235265000	-5.751981000	2.459049000
1	3.913954000	-1.461209000	1.678148000
1	5.207380000	-2.408870000	0.948871000
1	-2.036191000	4.706821000	0.740788000
1	-3.312309000	4.497303000	1.951226000
7	-0.540906000	3.309153000	2.527927000
19	2.570801000	1.520557000	1.024506000
8	1.852466000	4.134242000	0.381571000
8	2.420134000	2.502800000	3.568749000
8	4.073099000	2.732018000	-0.914027000
8	5.091268000	2.100352000	2.534985000
6	1.236094000	2.778651000	4.284487000
1	1.192803000	3.841433000	4.589627000
6	0.016083000	2.362073000	3.501970000
1	1.236555000	2.184926000	5.216928000
1	-0.784744000	2.151006000	4.225477000
1	0.220250000	1.399097000	3.001976000
6	3.594703000	2.706375000	4.322096000
1	3.917223000	3.760135000	4.249252000
1	3.408369000	2.477500000	5.386826000
6	4.694657000	1.786050000	3.859756000
1	5.552385000	1.908653000	4.544619000
1	4.353798000	0.736439000	3.925247000
6	6.322720000	1.534164000	2.128948000
1	6.835987000	2.280613000	1.506707000
1	6.968706000	1.360760000	3.006012000
6	6.190183000	0.214448000	1.383289000
1	7.215063000	-0.146857000	1.177730000
1	5.727621000	-0.521931000	2.050887000
6	0.148085000	4.520083000	2.097603000
6	0.545008000	4.622295000	0.628105000
1	-0.470086000	5.397913000	2.341979000
1	1.058529000	4.634554000	2.700008000
1	0.512819000	5.690283000	0.341778000
1	-0.163421000	4.082895000	-0.020607000
6	2.167138000	4.195723000	-1.001217000
1	1.869372000	5.179752000	-1.406843000
6	3.645117000	4.038285000	-1.232444000
1	1.595771000	3.423099000	-1.547239000
1	3.852094000	4.257805000	-2.296950000
1	4.192084000	4.776139000	-0.618961000
6	5.931463000	1.177599000	-0.906974000
1	7.033982000	1.174953000	-0.872204000
1	5.625059000	0.734159000	-1.862781000
6	5.476147000	2.626406000	-0.885863000
1	5.913661000	3.101727000	-1.784414000
1	5.867189000	3.180766000	-0.014642000
8	4.249912000	4.800070000	1.894838000
1	3.387643000	4.628672000	1.482285000
1	4.605467000	3.914427000	2.074669000
6	-0.120801000	0.036277000	0.256515000
8	0.590508000	-0.316042000	1.203648000
8	0.178400000	1.301707000	-0.184537000
8	-1.423947000	2.829240000	-1.709020000
1	-2.204219000	2.894838000	-1.126605000
1	-0.455495000	1.633923000	-0.860184000
1	-1.798626000	2.529508000	-2.549546000

^{5}**RC2** $_{H3O+, K+}$

6	1.379545000	5.658241000	-1.286120000
6	0.186851000	5.276562000	-1.920726000
6	-0.594916000	6.269536000	-2.530967000
6	-0.193251000	7.604389000	-2.512296000
6	0.994322000	7.970830000	-1.878398000
6	1.778474000	6.993663000	-1.264155000
6	-0.246004000	3.852293000	-1.937653000
6	0.606090000	2.914230000	-2.558243000
7	0.423460000	1.556722000	-2.545932000
6	1.517350000	0.985151000	-3.124054000
6	2.426267000	2.022267000	-3.558841000
6	1.853536000	3.216006000	-3.227249000
26	-1.162362000	0.469529000	-1.707191000
7	-2.058008000	2.309070000	-1.245004000
6	-3.148760000	2.386246000	-0.434150000
6	-3.258486000	3.733361000	0.088224000
6	-2.210841000	4.445512000	-0.447281000
6	-1.461947000	3.534034000	-1.276155000
6	-4.041239000	1.330954000	-0.134677000
6	-5.098943000	1.580511000	0.891713000
6	-4.874295000	1.284255000	2.247953000
6	-5.859600000	1.489255000	3.211421000
6	-7.108354000	1.972636000	2.827091000
6	-7.358468000	2.274558000	1.489983000
6	-6.357301000	2.084828000	0.539793000
6	1.729083000	-0.400482000	-3.303723000
6	3.087791000	-0.844635000	-3.731987000
6	3.325759000	-1.250986000	-5.053243000
6	4.575414000	-1.708365000	-5.462156000
6	5.617254000	-1.783310000	-4.537818000
6	5.404840000	-1.389351000	-3.220017000
6	4.153506000	-0.913097000	-2.817098000
8	3.952517000	-0.545283000	-1.504915000
6	4.707214000	0.584049000	-1.111230000
6	4.656601000	0.958873000	0.370595000
7	5.125793000	0.095452000	1.331334000
8	-3.599781000	0.866933000	2.623544000
6	-3.412528000	-0.162205000	3.609903000
6	-2.819508000	-1.390775000	2.918175000
8	-3.456020000	-1.860872000	1.985822000
7	-0.541367000	-1.213948000	-2.767997000
6	0.750642000	-1.406620000	-3.162255000
6	0.950170000	-2.804682000	-3.484462000
6	-0.251759000	-3.422539000	-3.308543000
6	-1.181230000	-2.412543000	-2.846795000
6	-2.520513000	-2.663114000	-2.464809000
6	-3.054340000	-4.034112000	-2.701072000
6	-3.414586000	-4.860940000	-1.625790000
6	-3.905483000	-6.146626000	-1.848646000
6	-4.047165000	-6.627793000	-3.150703000
6	-3.691982000	-5.815818000	-4.228262000
6	-3.197707000	-4.531309000	-4.005222000
7	-3.000096000	-0.470212000	-1.452660000
6	-3.361528000	-1.730546000	-1.821004000
6	-4.712730000	-1.994131000	-1.363973000
6	-5.106681000	-0.905496000	-0.649504000
6	-4.022320000	0.050827000	-0.719020000
8	4.324260000	2.102040000	0.644151000
1	-3.296895000	-4.490082000	-0.604240000
1	1.883337000	-3.254330000	-3.816272000
1	-0.481972000	-4.475277000	-3.458069000
1	3.364474000	1.860447000	-4.086793000
1	2.242696000	4.212270000	-3.425756000
1	-1.979646000	5.495766000	-0.281462000
1	-4.067267000	4.116772000	0.710319000
1	-6.055705000	-0.750290000	-0.140759000
1	-5.280801000	-2.902545000	-1.552022000
1	2.502032000	-1.199025000	-5.769572000
1	4.733617000	-2.011664000	-6.499466000
1	6.599294000	-2.155141000	-4.839438000

1	6.214134000	-1.459527000	-2.489530000
1	-1.522164000	5.986349000	-3.035538000
1	-0.811427000	8.362409000	-2.999748000
1	1.308133000	9.017444000	-1.862072000
1	2.706442000	7.272786000	-0.758858000
1	1.991343000	4.897668000	-0.794003000
1	-6.547549000	2.318221000	-0.510496000
1	-8.333167000	2.661386000	1.185142000
1	-7.880244000	2.127738000	3.584047000
1	-5.651302000	1.287096000	4.263748000
1	-4.174904000	-6.778049000	-0.998244000
1	-4.433145000	-7.635130000	-3.325377000
1	-3.803101000	-6.183036000	-5.251627000
1	-2.926063000	-3.896772000	-4.852669000
1	5.775298000	0.451312000	-1.364986000
1	4.339617000	1.477430000	-1.637251000
1	-2.831263000	0.260902000	4.436235000
1	-4.390596000	-0.465289000	4.005542000
7	-1.656202000	-1.932385000	3.350759000
19	2.107324000	-0.588339000	1.999810000
8	1.027396000	0.014107000	4.570385000
8	1.200332000	-3.253193000	2.557583000
8	3.605700000	0.928551000	3.753312000
8	4.075210000	-2.517556000	2.476853000
6	-0.008583000	-3.856698000	2.975215000
1	0.006706000	-4.052453000	4.064709000
6	-1.230605000	-3.082423000	2.546851000
1	-0.091745000	-4.845846000	2.488375000
1	-2.070720000	-3.793545000	2.519420000
1	-1.083533000	-2.732098000	1.513489000
6	2.290454000	-4.140168000	2.676421000
1	2.561023000	-4.279639000	3.739226000
1	2.011058000	-5.130394000	2.269658000
6	3.489927000	-3.672139000	1.893117000
1	4.224243000	-4.496078000	1.892764000
1	3.203439000	-3.477238000	0.843239000
6	5.422469000	-2.268292000	2.107975000
1	5.954696000	-1.953264000	3.017237000
1	5.898810000	-3.205397000	1.776068000
6	5.600985000	-1.243107000	0.997223000
1	6.682107000	-1.217211000	0.761404000
1	5.073524000	-1.584313000	0.097626000
6	-0.892432000	-1.446476000	4.489475000
6	-0.376035000	-0.006846000	4.385134000
1	-1.469479000	-1.580018000	5.418202000
1	-0.011779000	-2.090626000	4.595526000
1	-0.839376000	0.631088000	5.158852000
1	-0.618634000	0.404372000	3.390330000
6	1.529344000	1.307671000	4.848358000
1	1.087185000	1.693144000	5.785132000
6	3.023502000	1.242186000	4.997460000
1	1.258174000	2.006760000	4.034732000
1	3.391963000	2.218687000	5.363284000
1	3.284414000	0.476761000	5.750268000
6	5.672734000	0.727646000	2.533706000
1	6.711420000	0.377893000	2.661928000
1	5.704022000	1.805457000	2.331010000
6	4.949912000	0.524302000	3.851046000
1	5.486027000	1.140843000	4.596312000
1	4.994550000	-0.518653000	4.210741000
8	2.960530000	-2.097418000	5.185514000
1	2.261187000	-1.445815000	5.019023000
1	3.387117000	-2.230580000	4.324395000
6	-0.211755000	0.303860000	0.177241000
8	-0.423172000	-0.419599000	1.144947000
8	0.837948000	1.190097000	0.391405000
8	-1.890126000	2.640501000	2.379034000
1	-2.607396000	1.866290000	2.503823000
1	0.983252000	1.649132000	-0.450476000
1	-2.157452000	3.180501000	1.576471000
1	-1.888136000	3.225100000	3.157813000

^{5}**TS2** $_{H_3O^+, K^+}$

6	1.900879000	5.532716000	-0.806109000
6	0.729032000	5.328535000	-1.551311000
6	0.111135000	6.438612000	-2.146911000
6	0.652443000	7.715658000	-2.006270000
6	1.818077000	7.905621000	-1.263528000
6	2.439692000	6.810341000	-0.662821000
6	0.144831000	3.966289000	-1.699250000
6	0.932271000	2.978113000	-2.331000000
7	0.605047000	1.655957000	-2.434790000
6	1.677859000	1.002827000	-2.962574000
6	2.720718000	1.959948000	-3.262281000
6	2.253793000	3.185006000	-2.886921000
26	-1.136027000	0.710984000	-1.795201000
7	-1.855975000	2.587515000	-1.211763000
6	-2.965218000	2.726064000	-0.430809000
6	-2.964384000	4.041416000	0.169705000
6	-1.832874000	4.676476000	-0.276422000
6	-1.137228000	3.744488000	-1.132375000
6	-3.952307000	1.739923000	-0.210200000
6	-4.963532000	1.997218000	0.858005000
6	-4.742611000	1.507518000	2.160814000
6	-5.676064000	1.722430000	3.174842000
6	-6.855774000	2.410730000	2.896449000
6	-7.094623000	2.903164000	1.615393000
6	-6.148279000	2.699618000	0.612177000
6	1.767188000	-0.385509000	-3.204111000
6	3.103544000	-0.946172000	-3.561403000
6	3.378087000	-1.352671000	-4.875823000
6	4.598545000	-1.924337000	-5.224094000
6	5.572899000	-2.116641000	-4.245057000
6	5.323931000	-1.723567000	-2.933496000
6	4.104259000	-1.132813000	-2.590379000
8	3.873094000	-0.762747000	-1.282041000
6	4.677357000	0.317895000	-0.845893000
6	4.630826000	0.599517000	0.655818000
7	5.123974000	-0.315601000	1.548768000
8	-3.540820000	0.876399000	2.417767000
6	-3.491462000	-0.237885000	3.312533000
6	-2.936724000	-1.442190000	2.545037000
8	-3.542268000	-1.782356000	1.538744000
7	-0.599700000	-0.993077000	-2.840059000
6	0.688138000	-1.295433000	-3.176952000
6	0.767379000	-2.689759000	-3.552852000
6	-0.497458000	-3.193236000	-3.466205000
6	-1.348084000	-2.118950000	-3.004950000
6	-2.723485000	-2.254420000	-2.697979000
6	-3.368314000	-3.559935000	-3.015795000
6	-3.834321000	-4.398122000	-1.991224000
6	-4.427641000	-5.624083000	-2.288902000
6	-4.567495000	-6.032147000	-3.615901000
6	-4.107786000	-5.207649000	-4.643212000
6	-3.510970000	-3.983113000	-4.345553000
7	-3.043175000	-0.070387000	-1.608321000
6	-3.502075000	-1.276176000	-2.047017000
6	-4.885341000	-1.437205000	-1.641997000
6	-5.201394000	-0.354718000	-0.880997000
6	-4.035225000	0.501162000	-0.872819000
8	4.248714000	1.704101000	1.016042000
1	-3.719967000	-4.082969000	-0.950742000
1	1.669410000	-3.217911000	-3.853339000
1	-0.819250000	-4.211223000	-3.675049000

1	3.674203000	1.729488000	-3.734443000
1	2.758076000	4.143600000	-2.986535000
1	-1.506379000	5.686105000	-0.036008000
1	-3.743278000	4.449824000	0.812646000
1	-6.146613000	-0.138371000	-0.387240000
1	-5.524544000	-2.281578000	-1.890161000
1	2.606938000	-1.207530000	-5.636386000
1	4.786067000	-2.224381000	-6.257420000
1	6.530038000	-2.578177000	-4.498763000
1	6.082012000	-1.880670000	-2.162790000
1	-0.798556000	6.293694000	-2.735191000
1	0.161137000	8.567339000	-2.483344000
1	2.241306000	8.906896000	-1.151816000
1	3.349061000	6.950983000	-0.073244000
1	2.386139000	4.679145000	-0.325296000
1	-6.327833000	3.080037000	-0.396353000
1	-8.015349000	3.447768000	1.395654000
1	-7.582994000	2.572214000	3.695435000
1	-5.478248000	1.368126000	4.188251000
1	-4.779310000	-6.266413000	-1.477607000
1	-5.033681000	-6.992605000	-3.849184000
1	-4.216458000	-5.517815000	-5.685541000
1	-3.156349000	-3.338349000	-5.153753000
1	5.735514000	0.158043000	-1.121463000
1	4.334631000	1.250125000	-1.317691000
1	-2.948209000	0.053039000	4.219410000
1	-4.511763000	-0.518574000	3.609226000
7	-1.840615000	-2.110783000	2.984661000
19	2.049073000	-0.787123000	1.907820000
8	0.841593000	-0.484701000	4.524508000
8	0.928495000	-3.493920000	2.088454000
8	3.499189000	0.368735000	3.961856000
8	3.826181000	-2.922122000	2.289173000
6	-0.290046000	-4.092281000	2.484493000
1	-0.253376000	-4.377542000	3.553325000
6	-1.503341000	-3.264042000	2.143360000
1	-0.416059000	-5.036765000	1.923542000
1	-2.366639000	-3.950071000	2.143804000
1	-1.397269000	-2.901985000	1.110991000
6	1.967750000	-4.446081000	2.066172000
1	2.178333000	-4.811969000	3.088562000
1	1.661458000	-5.318669000	1.458354000
6	3.231388000	-3.903306000	1.452964000
1	3.925614000	-4.752702000	1.333999000
1	3.030900000	-3.490436000	0.447281000
6	5.221849000	-2.740404000	2.122094000
1	5.640985000	-2.536457000	3.117249000
1	5.685073000	-3.677872000	1.772026000
6	5.594939000	-1.634065000	1.145576000
1	6.697059000	-1.634993000	1.055520000
1	5.177111000	-1.869551000	0.159622000
6	-1.143081000	-1.833806000	4.230302000
6	-0.562736000	-0.424786000	4.378353000
1	-1.785675000	-2.070813000	5.093186000
1	-0.293979000	-2.524319000	4.294504000
1	-0.990233000	0.077335000	5.264179000
1	-0.803913000	0.184551000	3.493149000
6	1.391443000	0.729655000	5.001755000
1	0.922969000	1.003911000	5.964176000
6	2.869677000	0.563290000	5.206513000
1	1.192781000	1.547677000	4.284251000
1	3.266465000	1.467421000	5.704627000
1	3.055400000	-0.299174000	5.872360000
6	5.616004000	0.219151000	2.819319000
1	6.640805000	-0.159937000	2.969286000
1	5.673298000	1.308692000	2.700357000
6	4.829314000	-0.072158000	4.082750000
1	5.342493000	0.470388000	4.898457000
1	4.836786000	-1.141572000	4.358133000
8	2.557770000	-2.808128000	4.947905000
1	1.952298000	-2.055637000	4.858321000
1	3.009948000	-2.866170000	4.091421000
6	-0.376471000	0.294437000	0.156804000
8	-0.413247000	-0.639862000	0.902491000
8	0.314888000	1.476580000	0.868650000
8	-1.435319000	2.482256000	2.261653000
1	-2.220463000	1.854805000	2.315784000
1	0.537040000	2.129572000	0.182621000
1	-0.620622000	2.038436000	1.667500000
1	-1.755673000	3.270932000	1.766562000

^{5}IM2 $_{H3O+, K+}$

6	0.817076000	6.615147000	-0.806665000
6	-0.114782000	6.071032000	-1.701249000
6	-0.875287000	6.940072000	-2.495028000
6	-0.704954000	8.321218000	-2.399582000
6	0.226525000	8.852681000	-1.507421000
6	0.986578000	7.996007000	-0.710509000
6	-0.305027000	4.592032000	-1.802431000
6	0.697306000	3.853653000	-2.469080000
7	0.706128000	2.500199000	-2.672146000
6	1.928398000	2.166394000	-3.188573000
6	2.713903000	3.365017000	-3.356989000
6	1.940489000	4.411004000	-2.942717000
26	-0.596690000	1.132357000	-1.772104000
7	-1.788559000	2.732073000	-1.137698000
6	-2.915608000	2.617308000	-0.379089000
6	-3.305824000	3.923852000	0.104493000
6	-2.398974000	4.814558000	-0.392560000
6	-1.443378000	4.050638000	-1.168786000
6	-3.632362000	1.427232000	-0.148508000
6	-4.832454000	1.475227000	0.742187000
6	-4.708114000	1.253936000	2.127903000
6	-5.839040000	1.230619000	2.945988000
6	-7.103635000	1.425743000	2.393488000
6	-7.245077000	1.662052000	1.027804000
6	-6.111579000	1.687887000	0.216759000
6	2.371648000	0.854759000	-3.481609000
6	3.791114000	0.665206000	-3.899295000
6	4.234781000	1.181212000	-5.128373000
6	5.533209000	0.988707000	-5.588201000
6	6.432646000	0.260218000	-4.808776000
6	6.025625000	-0.255222000	-3.584294000
6	4.718952000	-0.062355000	-3.119166000
8	4.441010000	-0.670346000	-1.930987000
6	3.605811000	-0.063338000	-0.967278000
6	4.194903000	-0.182235000	0.437973000
7	4.432475000	-1.421786000	0.970360000
8	-3.438792000	1.165490000	2.636416000
6	-3.112996000	0.297250000	3.694905000
6	-2.749234000	-1.083960000	3.149670000
8	-3.131257000	-1.380190000	2.029062000
7	0.329403000	-0.378493000	-2.857075000
6	1.581287000	-0.308183000	-3.390235000
6	1.980099000	-1.614053000	-3.865793000
6	0.940016000	-2.457082000	-3.614447000
6	-0.095295000	-1.668898000	-2.978106000
6	-1.333692000	-2.175032000	-2.529657000
6	-1.628194000	-3.612590000	-2.792784000
6	-1.775242000	-4.514619000	-1.727945000
6	-2.044116000	-5.861644000	-1.966309000
6	-2.175091000	-6.329216000	-3.274369000
6	-2.035627000	-5.441271000	-4.341418000
6	-1.763059000	-4.094728000	-4.102944000

7	-2.188368000	-0.138808000	-1.405442000
6	-2.310301000	-1.432444000	-1.831458000
6	-3.606110000	-1.940751000	-1.446112000
6	-4.229720000	-0.951368000	-0.748051000
6	-3.336466000	0.179577000	-0.736377000
8	4.413762000	0.847990000	1.058413000
1	-1.671159000	-4.151068000	-0.702197000
1	2.925166000	-1.850381000	-4.350354000
1	0.879616000	-3.519326000	-3.839705000
1	3.728115000	3.410463000	-3.746690000
1	2.204510000	5.466145000	-2.936585000
1	-2.381257000	5.893267000	-0.252011000
1	-4.173749000	4.133699000	0.727380000
1	-5.215556000	-0.978471000	-0.290764000
1	-3.991612000	-2.930646000	-1.678645000
1	3.517449000	1.729593000	-5.744069000
1	5.837750000	1.397335000	-6.554083000
1	7.457216000	0.096222000	-5.151173000
1	6.712516000	-0.820396000	-2.950843000
1	-1.604299000	6.526481000	-3.196647000
1	-1.303291000	8.985458000	-3.028229000
1	0.359659000	9.934736000	-1.432445000
1	1.715539000	8.404529000	-0.006146000
1	1.410096000	5.945879000	-0.177928000
1	-6.211377000	1.861741000	-0.857622000
1	-8.233617000	1.824358000	0.592970000
1	-7.980943000	1.405510000	3.044329000
1	-5.738791000	1.077037000	4.021964000
1	-2.149330000	-6.549885000	-1.123799000
1	-2.387171000	-7.384633000	-3.462039000
1	-2.143554000	-5.797693000	-5.368872000
1	-1.662122000	-3.402128000	-4.942337000
1	3.524186000	1.015875000	-1.143884000
1	2.592721000	-0.500025000	-1.006237000
1	-2.292650000	0.771126000	4.244595000
1	-3.945206000	0.172862000	4.407819000
7	-2.076089000	-1.960150000	3.953369000
19	1.154074000	-1.369795000	2.218123000
8	0.797451000	-1.285061000	5.050275000
8	-0.198790000	-3.813562000	2.370146000
8	3.466603000	-1.133150000	3.772197000
8	2.575072000	-3.782887000	1.549091000
6	-0.985261000	-4.211545000	3.476749000
1	-0.363733000	-4.210633000	4.389318000
6	-2.233146000	-3.375920000	3.624606000
1	-1.334288000	-5.249411000	3.334189000
1	-2.846558000	-3.848527000	4.412506000
1	-2.800977000	-3.418579000	2.687975000
6	0.468470000	-4.899894000	1.763821000
1	0.938069000	-5.539450000	2.532380000
1	-0.249095000	-5.523526000	1.198276000
6	1.535481000	-4.416850000	0.819075000
1	1.932578000	-5.295017000	0.282514000
1	1.111364000	-3.731105000	0.062130000
6	3.870649000	-3.868500000	0.981097000
1	4.574961000	-4.073422000	1.800053000
1	3.924079000	-4.735395000	0.302161000
6	4.302394000	-2.645973000	0.190118000
1	5.271053000	-2.897613000	-0.279708000
1	3.594702000	-2.478237000	-0.630807000
6	-1.632018000	-1.632555000	5.304793000
6	-0.431087000	-0.716908000	5.465835000
1	-2.454156000	-1.177154000	5.883087000
1	-1.408183000	-2.581469000	5.812072000
1	-0.370318000	-0.476060000	6.543108000
1	-0.574381000	0.241847000	4.936869000
6	1.875838000	-0.444217000	5.437315000
1	1.815535000	-0.246340000	6.522764000
6	3.212359000	-1.071883000	5.158505000
1	1.798435000	0.530851000	4.919590000
1	3.982805000	-0.454957000	5.658107000
1	3.251872000	-2.082015000	5.602813000
6	5.267152000	-1.453259000	2.166185000
1	6.124523000	-2.123775000	1.983513000
1	5.657695000	-0.435976000	2.293035000
6	4.625210000	-1.881729000	3.475550000
1	5.391165000	-1.719261000	4.255945000
1	4.377895000	-2.955951000	3.499324000
8	2.137385000	-4.025931000	4.496684000
1	1.611032000	-3.243501000	4.716916000
1	2.303328000	-3.952678000	3.542909000
6	0.742474000	1.559677000	-0.141083000
8	1.418673000	2.176829000	0.525807000
8	-0.707131000	0.444683000	1.716855000
8	-1.325706000	2.965749000	2.520660000
1	-2.249512000	2.674281000	2.608791000
1	-1.552595000	0.162981000	1.337186000
1	-0.858652000	1.380332000	1.988361000
1	-1.365839000	3.649610000	1.837659000
